# On-Resin Photochemical Decarboxylative Arylation of Peptides

**DOI:** 10.1021/acs.orglett.3c03070

**Published:** 2023-10-11

**Authors:** Sunit Pal, Joseph Openy, Adrian Krzyzanowski, Anaïs Noisier, Peter ‘t Hart

**Affiliations:** † Chemical Genomics Centre, Max Planck Institute of Molecular Physiology, 44227 Dortmund, Germany; ‡ Department of Chemical Biology, Max Planck Institute of Molecular Physiology, 44227 Dortmund, Germany; § Medicinal Chemistry, Research and Early Development Cardiovascular, Renal and Metabolism BioPharmaceutical R&D, AstraZeneca, 431 83 Gothenburg, Sweden

## Abstract

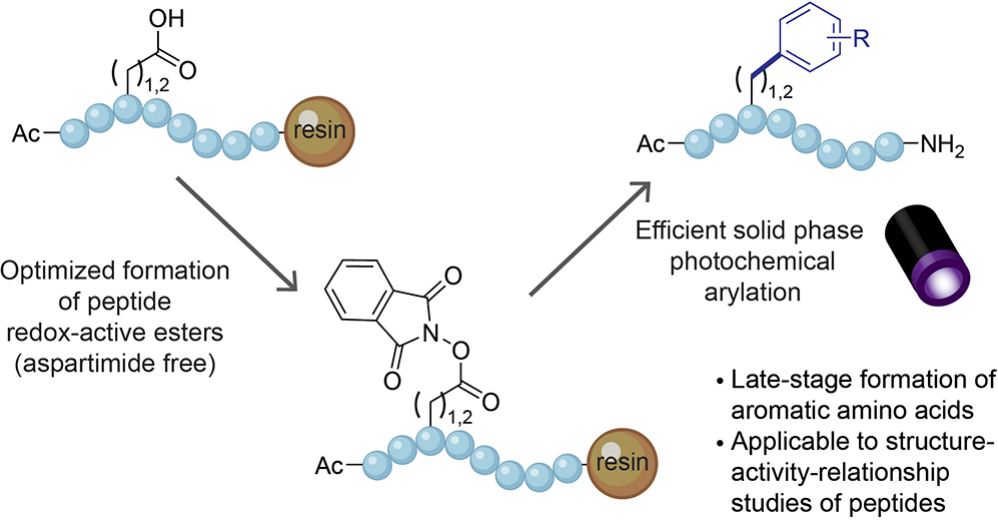

Here we describe the application of photochemical decarboxylative arylation as a late-stage functionalization reaction for peptides. The reaction uses redox-active esters of aspartic acid and glutamic acid on the solid phase to provide analogues of aromatic amino acids. By using aryl bromides as arylation reagents, a wide variety of amino acids can be accessed without having to synthesize them individually in solution. The reaction is compatible with proteinogenic amino acids and was used to perform a structure–activity relationship study of a PRMT5 binding peptide.

The increasing interest in peptides as a modality in drug discovery creates a demand for optimized synthetic methods to prepare unnatural amino acids (UAAs).^1,2^ UAAs can enhance the affinity of a peptide for its target by making new interactions that cannot be achieved with proteinogenic amino acids as well as provide proteolytic stability.^3−8^ Traditionally, UAAs suitably protected for Fmoc solid-phase peptide synthesis (SPPS) are prepared via multistep synthetic routes which often require thorough optimization to produce high optical purity and yield.^9^ Instead, by leveraging the existing chirality of natural amino acids, late-stage functionalization (LSF) approaches allow the rapid generation of an array of modified peptides in their enantiomerically pure form. In the past decade, several protocols have been developed for the LSF of peptides.^10−15^ While solution-phase LSF was quite successful for short peptides, only exquisitely selective methods can be employed with more complex peptides. Furthermore, peptides longer than five or six amino acids face solubility problems in organic solvents.^16,17^ Therefore, methodologies to perform LSF on the solid phase open up the possibility to modify larger peptides. In addition, site selectivity is easily achieved via use of orthogonally protected amino acids, which after deprotection are readily converted to the desired functionality. While cumbersome in the solution phase, these steps are rendered straightforward on the solid phase, as the removal of reagents and high-boiling solvents only requires simple filtration.

One class of organic transformations that can be exploited for peptide LSF are decarboxylative C_sp^3^_–C_sp^2^_ cross-coupling (DCC) reactions of redox-active esters (RAEs) (Figure 1).^18−20^ In the context of peptides, DCC of aspartic acid (Asp) or glutamic acid (Glu) provides straightforward access to phenylalanine (Phe) and homophenylalanine (hPhe) analogues.^21^ Optimization of these residues is often beneficial for peptide affinity and stability, as illustrated by the clinically approved degarelix as well as the tricyclic peptidic proprotein convertase subtilisin-like/kexin type 9 (PCSK9) inhibitor currently in clinical trials.^6,7^ The Baran group reported a C_sp^3^_–C_sp^2^_ DCC reaction using nickel catalysis and organozinc reagents (Figure 1).^22^ Later, the Molander group reported electron donor–acceptor (EDA) formation between a RAE and the inexpensive Hantzsch ester (HE) which could undergo photoinduced radical-mediated DCC to functionalize single amino acids.^23^ More recently, the Baran group demonstrated the use of electrochemistry on RAE of Asp and Glu to yield aromatic amino acids.^24^ We hypothesized that the photochemical method from the Molander group would be most applicable to derivatize peptides directly on the solid phase. However, a limitation of the activation of Asp as a RAE is that it will rapidly undergo aspartimide formation with the neighboring amino acid in the peptide sequence. Here, we first optimized the RAE-forming reaction conditions to mitigate aspartimide- or pyroglutamate (from Glu)-forming side reactions.^25^ Thereafter, the key solid-phase photochemistry reaction conditions were screened, and improved conditions were identified which enabled the functionalization of peptides in good yields. The substrate scope was also investigated by reacting a broad selection of aryl bromides (Ar–Br) with a resin-bound model peptide. In addition, we tested the compatibility of all canonical amino acids as well as various commonly used Fmoc SPPS linker-resins with the photochemistry conditions. Finally, we modified biologically relevant peptides, including a protein arginine methyltransferase 5 (PRMT5) binding peptide, to demonstrate the broad applicability of the current methodology.

**Figure 1:**
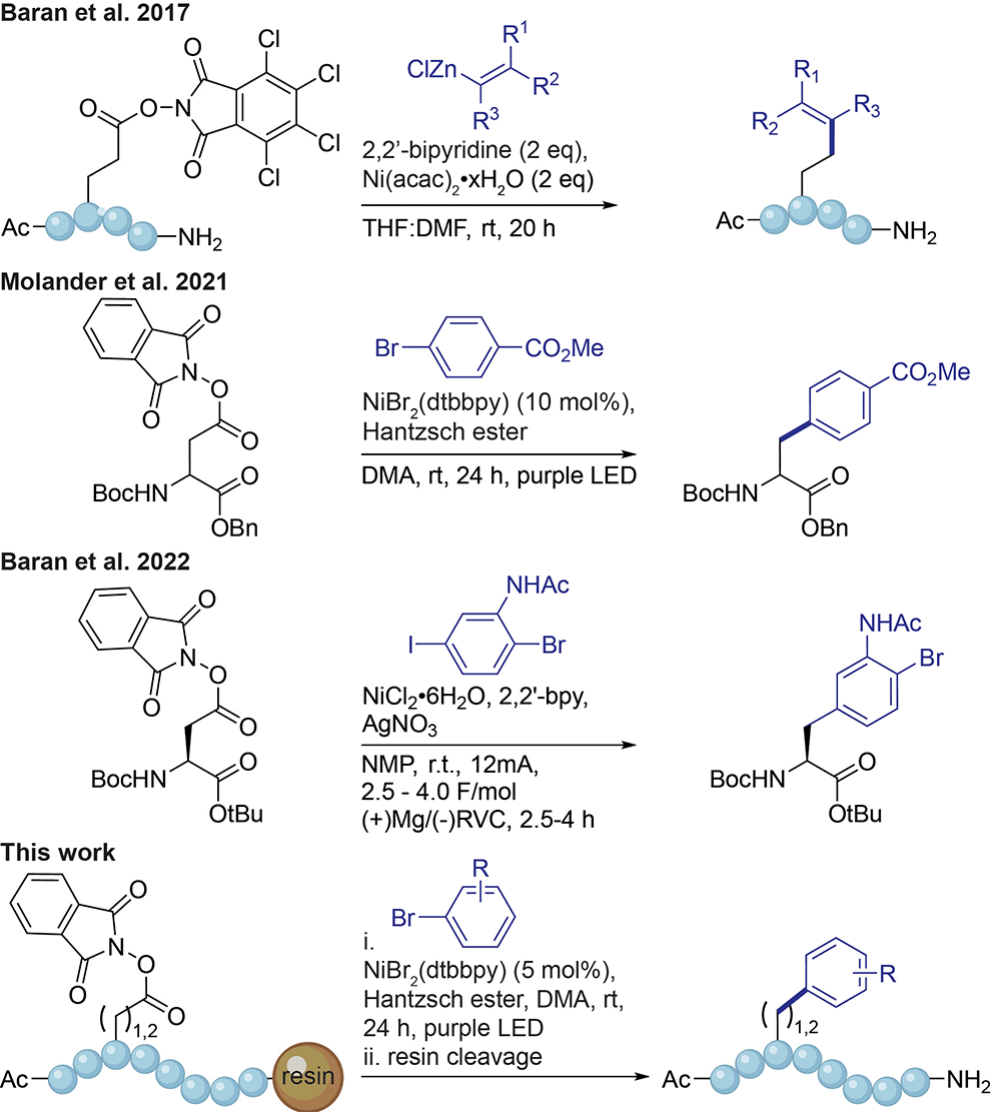
Previously reported and current decarboxylative strategies of redox-active esters to functionalize amino acids and peptides.

Initially, we designed a resin-bound model tripeptide to test the photochemical LSF. The tripeptide contains the Asp to be modified at the *N*-terminus and a tyrosine (Tyr) residue at the *C*-terminus for easy detection during HPLC analysis and purification. Briefly, the *N*-terminally acetylated tripeptide was synthesized on a Rink amide polystyrene (PS) resin, followed by removal of the allyl side-chain protecting group of Asp by treatment with Pd(PPh_3_)_4_ and phenylsilane (Scheme S1). Next, *N*-hydroxypthalimide (NHPI) was to be coupled to the free acid to obtain the RAE, but this was complicated by aspartimide formation.^26,27^ Solid-phase activation of Asp and Glu side-chain carboxylic acids with NHPI has previously been performed using excesses of carbodiimide or hexafluorophosphate azabenzotriazole tetramethyl uronium (HATU) and various bases.^28,29^ However, in these reports, proline was always used as the neighboring amino acid, eliminating the possibility for aspartimide formation but restricting the applicability to peptide sequences with an Asp-Pro pair. Instead, we looked for a general protocol that avoids this side reaction, irrespective of the nature of the next amino acid. Since basic conditions drive this side reaction, we hypothesized that the equivalents of base used could be fine-tuned. Therefore, we used NHPI (8 equiv), HATU (4 equiv), and varying amounts of diisopropylethylamine (DIPEA). Interestingly, although 8 equiv led to substantial aspartimide formation (Figure S2), we could not observe any when using 6 equiv, while 4 equiv led to incomplete conversion. Note that isolation of the activated ester was not possible because it is unstable during prolonged exposure to HPLC buffers during preparative purification.

With reliable conditions for the formation of the RAE in hand, we tested the EDA-complex-mediated DCC reaction on the resin-bound peptide with 4-bromobenzonitrile by adapting the protocol by Molander et al.^23^ The reduced mass transfer effect of the solid support prompted us to use slight excess amounts of Ar–Br and HE. We observed satisfactory conversion of the starting material to **1** by HPLC, and the highest yields were obtained when using 4 equiv of Ar–Br and HE with 5 mol % Ni catalyst (Table 1, entries 1–4, and Figure S3). The yields for the reaction were calculated over three steps (including RAE formation). These are similar to or higher than those of previously reported cross-coupling reactions directly on peptides on the solid phase.^22,28,30^ Interestingly, the cross-coupling reaction was catalytic, and a reduced yield was observed at higher catalyst loadings, unlike other reports where an equivalent amount of nickel catalyst or more was required to successfully convert the resin-bound peptide.^22,28^ Lowering the amount of either Ar–Br or HE reduced the overall yield of the reaction (entries 5 and 6). Performing the reaction using NiBr_2·_3H_2_O or removing the catalyst altogether led to a complete loss of conversion (entries 7 and 8). The light source played a key role in EDA-driven radical generation since only purple LEDs could promote the reaction, while the use of blue LED light or absence of light did not yield any product (entries 9 and 10). To investigate the compatibility with alternative linker-resins, we tested Rink amide poly(ethylene glycol) (PEG) resin as well as Wang PS to provide a free C-terminus (entries 11 and 12). Both alternatives were less efficient but still afforded the desired products.

**Table 1:**
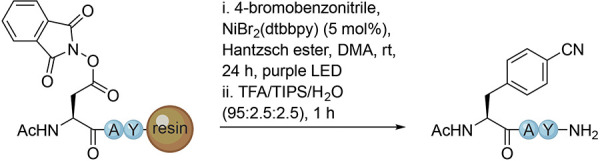
Optimization of On-Resin Decarboxylative Photochemistry

entry	catalyst loading (mol %)	equiv. of Ar–Br	equiv. of HE	yield (%)^a^
1	40	4	4	26
2	20	4	4	31
3	10	4	4	41
4	5	4	4	44
5	5	2	4	29
6	5	4	2	26
7	5 (NiBr_2_·3H_2_O)	4	4	NR
8	no cat.	4	4	NR
9	5 (no LED)	4	4	NR
10	5 (blue LED)	4	4	NR
11	5 (Rink PEG resin)	4	4	17
12^b^	5 (Wang PS resin)	4	4	27

a NMR yields calculated over three steps starting with RAE formation. NR: no reaction.

b Provides peptide **1-OH**.

Next, a variety of aryl bromides with different substituents were tested using the optimized conditions to explore the scope of the methodology (Figure 2). Substrates with electron-withdrawing groups at the *para* position (**1**, **2**, and **6**) provided good yields of 38–49%, and substitutions at the *ortho* and *meta* positions were tolerated as well (**3** and **4**). Additionally, the RAE underwent cross-coupling with aryl bromides containing electron-donating groups or completely unactivated bromobenzene to obtain **5**, **9**, and **14**–**16** in moderate to good yields. Disubstitution of the phenyl ring was possible and provided molecule **7**. However, a reduced yield was obtained, possibly due to steric encumbrance during in situ formation of the activated Ni complex as previously described.^23^ Polycyclic aromatic bromides could also be used efficiently to obtain peptides **8** and **10**–**13**. These results demonstrated that the electronics at the C–Br bond of the aryl bromide reactant do not extensively influence the Ni-catalyzed cross-coupling reaction. To further explore the reaction, variants of the tripeptide were prepared containing Glu replacing Asp as well as the d enantiomers of Asp and Glu. These starting materials provide access to analogues of d-Phe and both l and d analogues of hPhe, all of which are often not commercially available or very costly but can be valuable in a SAR study.^31−33^ A subset of aryl bromides was tested and found to successfully afford the functionalized peptides **17**–**34** in good yields (Figure 2). Surprisingly, d-Glu performed better in various cases, suggesting that it was the least affected by steric occlusion in the context of this model peptide. Additionally, during the DCC reaction, no epimerization was observed after analysis of the crude NMR spectra of molecules **1** and **17** (Figure S4).

**Figure 2:**
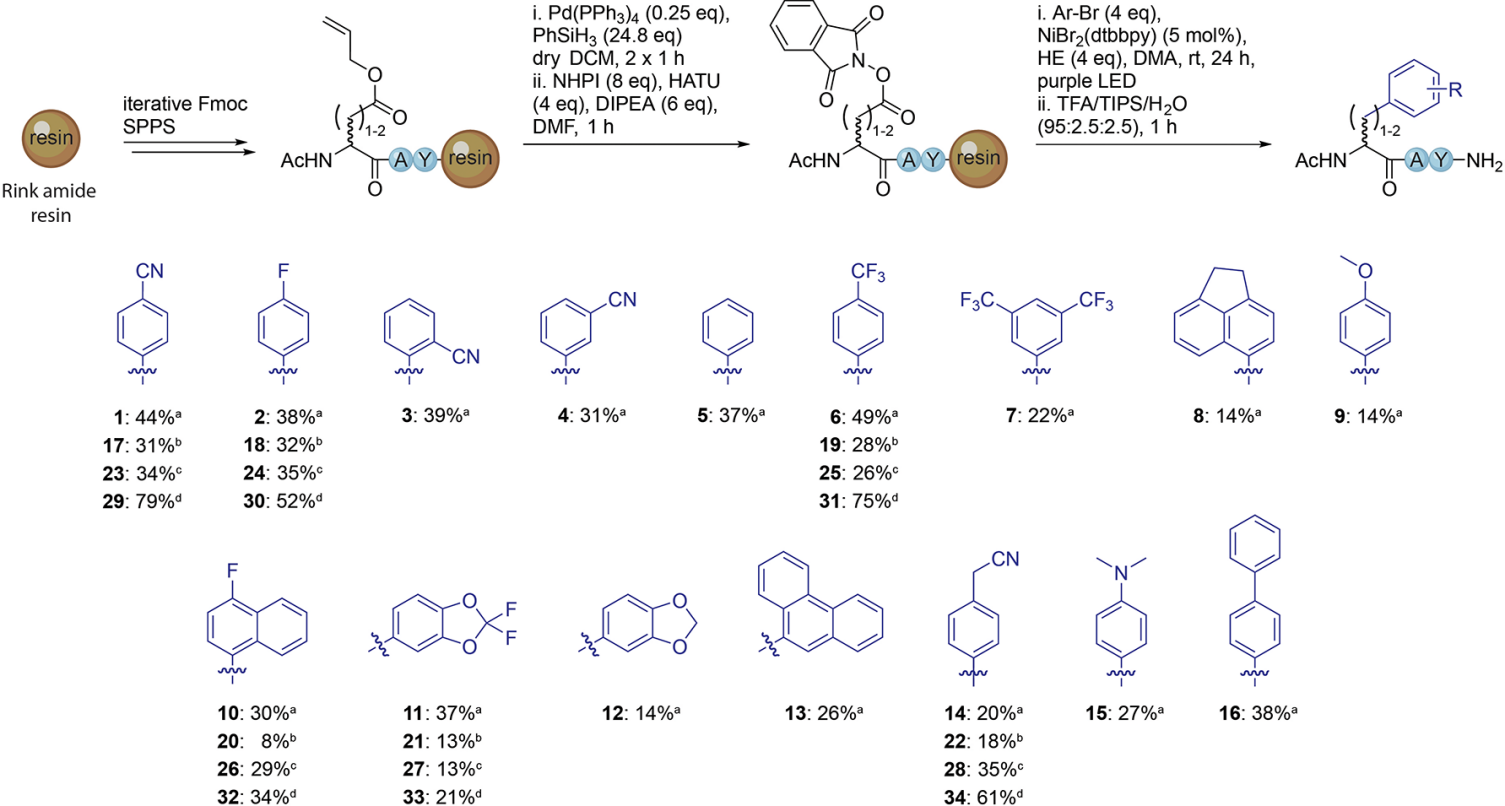
Scope of the decarboxylative photochemical reaction of the resin-bound peptides for the C_sp^3^_–C_sp^2^_ cross-coupling reaction. Yields were determined by ^1^H NMR calculated over three steps starting with RAE formation. ^a^From l-Asp. ^b^From d-Asp. ^c^From l-Glu. ^d^From d-Glu.

Various proteinogenic amino acids (e.g., Cys, Trp, Met, and His) can undergo photochemical diversification due to their reactive side-chain functionalities.^13^ To probe whether our method was compatible with these, we synthesized model tetrapeptides placing one of these amino acids at the *N*-terminus (Figure 3A). Note that from this point, yields for all peptides were calculated over all synthetic steps after loading of the first amino acid. After RAE formation and photochemical DCC, the desired modified peptides **35**–**38** were obtained in all cases. These results indicated the compatibility with these amino acids but also that the amino acid to be modified does not need to be *N*-terminal.

**Figure 3:**
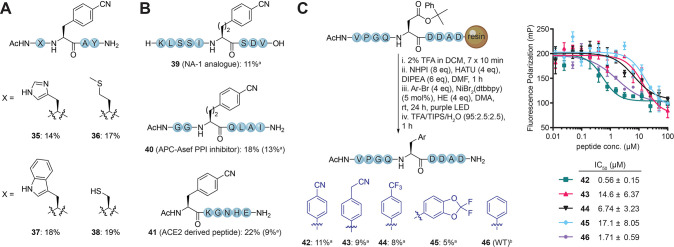
(A) Compatibility of sensitive amino acids with the photochemical decarboxylative arylation reaction. Yields were determined by ^1^H NMR over 12 steps. (B) Modification of biologically active peptides. Yields were determined by ^1^H NMR over all steps after the first amino acid loading. (C) SAR study of a PRMT5 binding peptide and evaluation via competitive fluorescence polarization assay. ^a^Isolated yield. ^b^Peptide prepared using regular SPPS.

To demonstrate the applicability of the methodology, we modified three biologically active peptides. The NA-1 peptide **39** was synthesized on Wang resin, and the *N*-terminal Fmoc group was maintained (Figure 3B) until peptide cleavage.^34^ The on-resin photochemistry was found to be compatible with the Fmoc group, which allows further extension of a peptide after the decarboxylative arylation step. The APC-Asef inhibitor peptide **40** and ACE2-derived peptide **41** were synthesized on Rink amide resin prior to LSF, and both underwent decarboxylative arylation in good yields (Figure 3B).^35,36^

Next, we used the method to perform a small SAR study on a peptide that we previously identified as an inhibitor of PRMT5 protein–protein interactions.^37^ The peptide is derived from the PRMT5 binding protein RioK1 and contains a Phe that is important for high-affinity binding. We prepared a peptide with an Asp residue installed in its place, which was protected using the 2-phenylisopropyl group to avoid aspartimide formation during peptide extension that occurred while using the allyl group.^38^ The batch was split, and each portion was reacted with a different Ar–Br to rapidly generate peptides with diverse Phe analogues (Figure 3C). The prepared peptides were then tested in a competitive fluorescence polarization assay against a tracer peptide binding to the PRMT5/MEP50 complex.^37^ Satisfyingly, peptide **42** showed a 3-fold improvement in IC_50_ in comparison to the wild-type peptide **46**.

We demonstrated that EDA-driven decarboxylative Ni-catalyzed C_sp^3^_–C_sp^2^_ cross-coupling reactions can be translated to solid-phase peptide chemistry. First, we refined the protocol for the activation of the side-chain carboxylic group with NHPI, which was previously plagued by undesired aspartimide/pyroglutamate formation. Thus, we provide a methodology which alleviates the restrictions in neighboring amino acids, making it suitable for any peptide sequence. Next, we optimized the conditions for the cross-coupling using a simple photochemical setup, making it applicable in general peptide chemistry laboratories and providing yields typical for peptide synthesis. The method widely expands access to diverse Phe and hPhe analogues as well as their enantiomers using low-cost aryl bromides. By avoiding multistep solution-phase amino acid synthesis, it saves on large amounts of reagents and solvents, providing a greener alternative. Furthermore, the approach does not require any directing groups and is independent of the peptide sequence, making it applicable not only to biologically active peptides but also to other areas of peptide chemistry such as materials science.

## Data Availability

The data underlying this study are available in the published article and its Supporting Information.

## Supporting Information Available

General experimental procedures, supplemental figures supporting the conclusions, chromatographic purity analysis, and spectroscopic data for corresponding products (PDF)
