# Supplementary material for: On-Resin Photochemical Decarboxylative Arylation of Peptides
Source: Org Lett. 2023 Oct 11;26(14):2795–9. doi: 10.1021/acs.orglett.3c03070 (PMC11019635; doi:10.1021/acs.orglett.3c03070)
Supplement: Supplementary file 1 — ol3c03070_si_001.pdf [file ol3c03070_si_001.pdf]

Supporting Information

On-Resin Photochemical Decarboxylative Arylation of Peptides

Sunit Pal,[a] Joseph Openy,[a] Adrian Krzyzanowski,[b,†] Anaïs Noisier,[c] Peter ‘t Hart,*,[a]

[a]Chemical Genomics Centre, Max Planck Institute of Molecular Physiology, 44227 Dortmund, Germany

[b]Department of Chemical Biology, Max Planck Institute of Molecular Physiology, 44227, Dortmund Germany

[c]Medicinal Chemistry, Research and Early Development Cardiovascular, Renal and Metabolism BioPharmaceutical R&D, AstraZeneca, 431 83 Gothenburg, Sweden

Table of Contents

Table of Contents	2
Experimental Procedures	3
Results and Discussion	6
References	86

Experimental Procedures

1.1. Reagents

All solvents and reagents were of analytical grade and obtained from commercial sources unless stated otherwise. Anhydrous DMA was purchased from Acros and sparged with argon prior to use in moisture and air-sensitive reactions. All moisture-sensitive reactions were carried out in flamed dried 2 mL vials under an argon atmosphere.

Manual solid phase peptide synthesis was carried out on a promega® vacuum manifold while automatic solid phase synthesis was carried out using a Syro I® peptide synthesizer.

Analytical UHPLC was performed using 2.1 mm x 150 mm, 2.7 µm Zorbax Eclipse C18 Rapid Resolution columns equipped on either an Agilent 1290 or Agilent 1260 Infinity system. Analytical LCMS was performed using an Agilent 1260 Infinity system equipped with a 2.1 mm x 50 mm, 1.8 µm InfinityLab Poroshell 120 EC-C18 column. Preparatory HPLC purification was carried out using 125 mm x 21 mm, 5 µm, Macherey-Nagel C18 Gravity columns (Macherey-Nagel GmbH & Co. KG, Germany) on a Büchi Pure C-850 Flash Prep for tripeptides or an Infinity II LC-MS system (Agilent Technologies, USA) for longer peptides. High-resolution mass spectra were recorded on an LTQ Orbitrap in tandem with an HPLC-System fitted with a 50 mm x 1 mm, 1.9 µm Hypersyl GOLD using an electrospray ionization method.

Nuclear magnetic resonance (NMR) spectra were recorded using Bruker DRX700 spectrometers and chemical shifts are reported with reference to deuterated solvent peaks.

1.2. Purification methods

Peptides were purified by preparative HPLC on a Büchi Pure C-850 Flash Prep system, eluting with a binary mixture of MeCN and H_2O with both containing 0.1% TFA using a gradient of 0% - 15% MeCN over 60 min for peptides **1 – 38** and 0% - 50% MeCN over 60 min for peptides **39 - 46**.

2. General synthetic methods

a. General method for linear peptide synthesis

Solid phase peptide synthesis was carried out using the Fmoc strategy on Rink Amide AM resin (loading ca. 0.7 mmol/g, 100 – 200 mesh) unless stated otherwise. Fmoc-protected amino acids (4 eq) were coupled using PyBOP (4 eq) and DIPEA (8 eq) in DMF (14 ml / gram of resin) for 30 minutes at room temperature. Deprotection of the Fmoc protecting group was carried out using a 20% solution of piperidine in DMF (14 ml / gram of resin) for 5 minutes followed by addition of fresh reagents and further reaction for 10 minutes. Linear peptides were N-terminally acetylated using DIPEA (10 eq) and Ac_2O (10 eq) in DMF (14 ml / gram of resin) over 30 minutes. In between steps the resin was washed 4 x with DMF (14 ml / gram of resin).

b. General method for Allyl deprotection

After synthesizing the allyl-protected tripeptide (**P1**), the resin-bound peptide was washed with dry DCM (3 times). Then the resin (500 µmol) was treated with a solution of $Pd(PPh_3)_4$ (0.25 eq, 144.4 mg, 125 µmol) in 10 mL dry DCM (0.05 M) followed by adding of phenylsilane (24.8 eq, 1.34 ml, 12400 µmol) and shaken for 1 h. Afterward, the liquid was discarded and the resin was again treated with fresh reagent solution to obtain the fully side chain deprotected peptide (**P2**). The complete removal of the allyl group was checked by cleavage of a small portion of resin and LC-MS analysis. Next, the peptidyl resin was washed with DCM (10 mL X 4 times), DMF (10 mL X 4 times), 0.5% diethyldithiocarbamate in DMF (10 mL X 5 times, each time 5 mins), 11.5% pyridine hydrochloride in 5% MeOH/ DCM (10 mL X 5 times, each time 5 mins), DCM (10 mL X 4 times) and DMF (10 mL X 4 times). (**Note: The rigorous washing is necessary to complete removal of residual catalyst which might interfere later in the Ni-catalyzed photochemical reaction**).

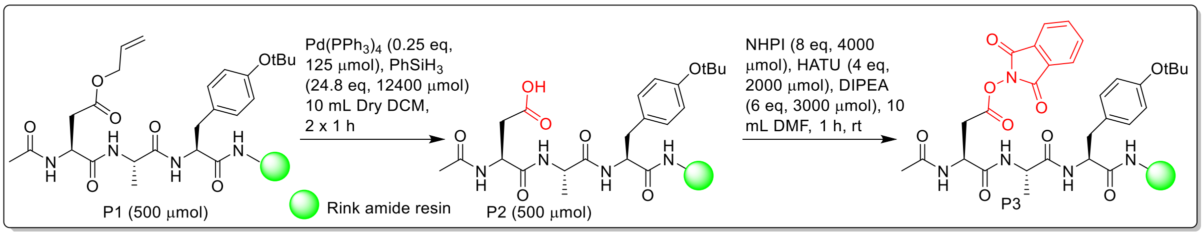

Scheme S1. Generation of activated ester on-resin.

c. General method for N-hydroxyphtalimide-activated peptides

The activation of the side chain carboxylic acid group was carried out by suspending 500 μmol of the resin-bound peptide in the solution of N-hydroxypthalimide (NHPI, 8 eq, 652.5 mg, 4000 μmol), HATU (4 eq, 758.5 mg, 2000 μmol) and DIPEA (6 eq, 0.522 ml, 3000 μmol) in 10 mL DMF (0.05 M) and shaken for 1 h at room temperature. The liquid was discarded, and the resin was washed with DMF (10 mL X 3 times), DCM (10 mL X 3 times) and diethyl ether (10 mL X 3 times) to obtain peptide **P3**. Then the resin was dried under high vaccum before it was used for the photochemical reaction. (**<u>To keep the activated ester intact, the dry resin must be stored at 4°C</u>**).

d. General method for decarboxylative Ni-catalyzed Csp^3-Csp^2 coupling reaction

To a 2 mL oven-dried borosilicate glass vial equipped with a magnetic stir bar and cap was added activated resin (30 μmol, 1 eq), $NiBr_2$(dtbbpy) (5 mol%, 1.50 μmol), HE (4 eq, 30.4 mg, 120 μmol), aryl bromide (4 eq, 120 μmol). The vial was evacuated three times with an inlet needle and then purged with Argon. The vial was charged with dry, degassed DMA (0.1 M, 300 μl). The vial was irradiated with Kessil purple lamp (model No – PR160L-390nm) for 24 h at room temperature. The distance between the vial and the lamp was kept at approx. 3 cm (see figure S1). The room temperature was maintained via a fan. After completion, the resin was washed with DCM (2 mL X 3 times), DMF (2 mL X 3 times), DCM (2 mL X 3 times) and the peptide was cleaved from the resin using 2 mL cleavage cocktail (95:2.5:2.5, TFA:TIPS:water, 1 h). Next, the TFA was removed followed by washing with DCM (2 mL X 3 times). Then, the crude peptide was dried under reduced pressure and submitted for NMR (in DMSO-d$_6$). An equimolar amount of 1,3,5-trimethoxybenzene was added to each NMR sample prior to determining the NMR yield. For compounds 1-34 the yields of the peptide after allyl deprotection were measured in the same way and used for the yield calculation of the final product.

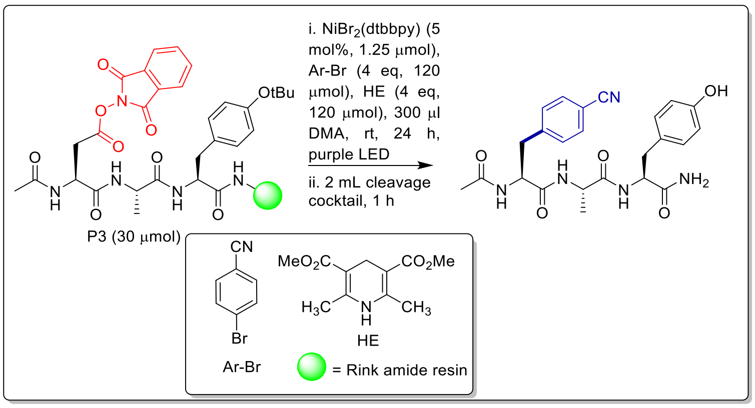

Scheme S2. On-resin photochemistry.

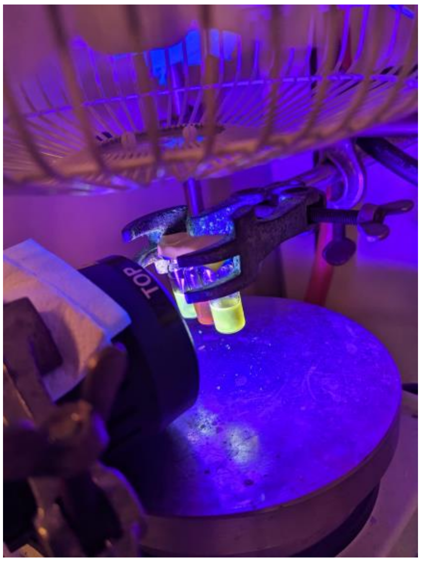

Figure S1. Photochemistry set-up.

e. General method for cleavage and deprotection of short peptides

Peptidyl resin was cleaved with TFA/TIPS/H_2O (95/2.5/2.5 v/v) for 1 h. The solution was collected and excess TFA was removed under a stream of argon followed by co-evaporation with $CHCl_3$ 3 times. TFA/DODT/TIPS/H_2O (90/2.5/2.5/5 v/v) cocktail was used for sulphur-containing peptides.[1]

f. General method for synthesis of $NiBr_2$(dtbbpy)

The synthesis of the Ni-catalyst was synthesized based on a previously reported protocol.[2] Briefly, a 50 mL, round bottom flask was equipped with a teflon-coated magnetic stirbar, $NiBr_2.3H_2O$ (1.0 eq, 0.46 g, 1.69 mmol), 4,4'-di-tert-butyl-2,2'-dipyridine (dtbbpy; 1.10 eq, 0.50 g, 1.86 mmol), dry ethanol (5 mL, 0.34 M) and a condenser with a rubber septum and nitrogen inlet. The reaction was heated to reflux overnight. The reaction was then cooled to room temperature and the solvent was reduced in vacuo to ~10% of its original volume. A 1:4 mixture of THF:diethyl ether (20 mL) was added to the residue and the mixture was stirred for 3 h under nitrogen. The mixture was then filtered and the collected light green solid was dried under vacuum overnight and stored in the desiccator. Yield: 0.7 g, 40 % Elemental Analysis: Calculated: C, 44.40; H, 4.97; N, 5.75. Found: C,44.4; H, 5.1; N, 5.5.

g. Method for synthesis of PRMT5 binding peptides for photochemistry

All peptides were synthesized following the *general method for linear peptide synthesis* mentioned above with a 2-phenylisopropyl protecting group on aspartic acid instead of allyl protecting group. Then the resin (1 eq, 200 μmol) was treated with 3 mL (0.067 M) solution of 2% TFA/DCM (v/v) and shaken for 10 min. Afterward, the liquid was discarded, and the resin was again treated with 2% TFA/DCM solution and the process was repeated five more times to obtain the completely deprotected free carboxylic acid on the aspartic acid. Next, the peptidyl resin was washed with DCM (5 mL X 4 times), 1% DIPEA in DMF (5 mL X 2 times), DMF (5 mL X 4 times). The activation of the side chain carboxylic acid group was and arylation were performed according to general methods c and d described above. Peptide cleavage and deprotection was performed according to general method e.

h. Competitive Fluorescence Polarization assay

Evaluation of peptide **42**-**46** was performed similar to our previously described protocol.[3] As tracer, we used peptide 50 from the same report. In brief, the peptides to be tested were serially diluted in buffer (50 mM HEPES, 250 mM NaCl, 1mM TCEP, and 0.01% (v/v) Tween 20, pH 8.0) in a black 384 wells plate in a total volume of 10 μl per well. Next, a mixture of PRMT5:MEP50 complex and tracer peptide 50 in the same buffer was added so that a final protein concentration of 200 nM and final tracer concentration of 1 nM was reached. The mixture was incubated for 1 hour and read on a Tecan Spark plate reader in fluorescence polarization mode set to an excitation wavelength of 485 nm and emission wavelength of 535 nm. Data was analyzed using Graphpad Prism version 9.2.0 and curves were fitted using nonlinear regression (log(inhibitor) vs. response – variable slope (four parameters)).

Results and Discussion

Base screening for the synthesis of activated ester on resin

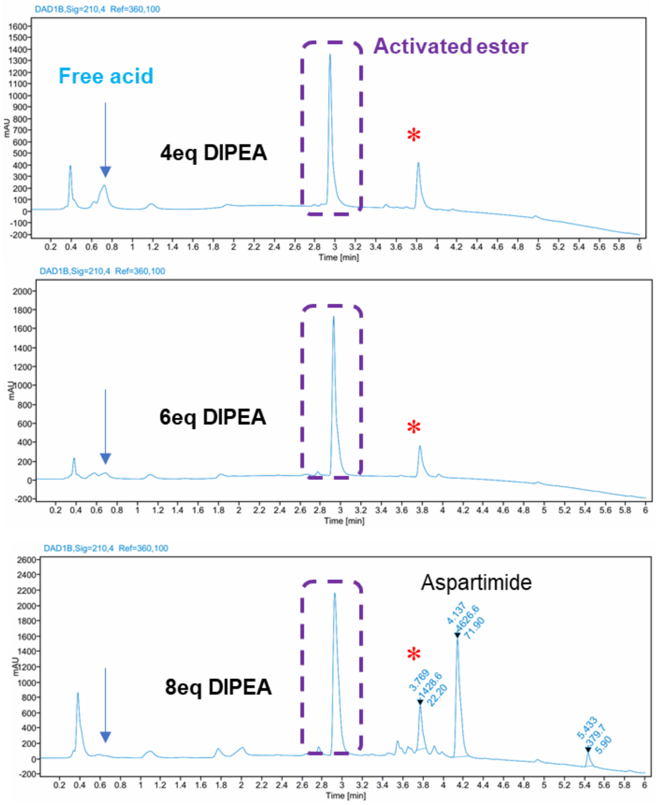

Figure S2. HPLC profiles of activated ester at different equivalents of base. *indicated unidentified peak appeared during the synthesis.

Reaction analysis of optimal reaction conditions for photochemical decarboxylative arylation

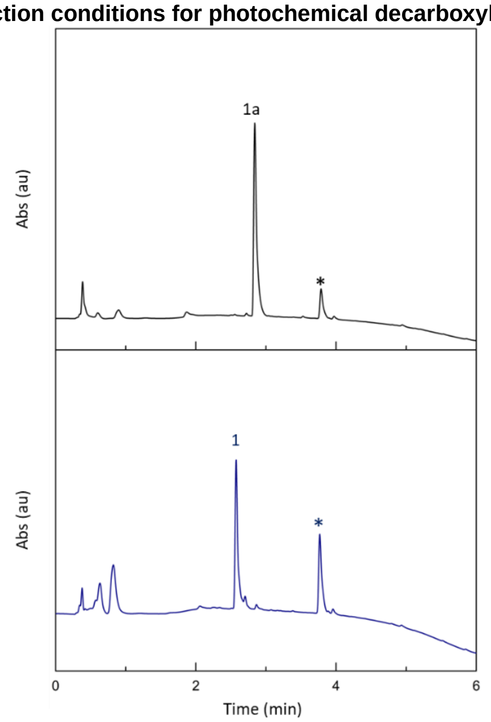

Figure S3. HPLC profiles of conversion of activated ester 1a to final product 1. *indicated unidentified peak appeared during the synthesis.

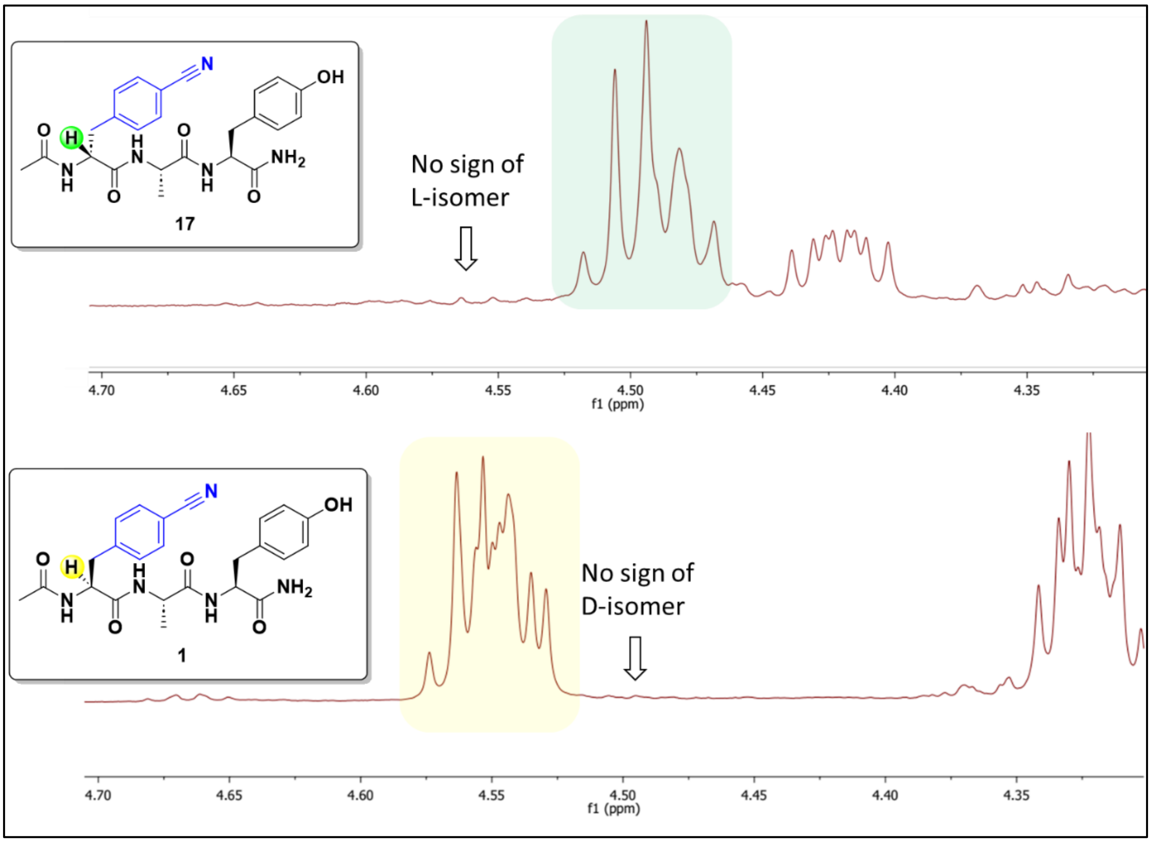

Figure S4. Stacked NMR spectra of the H$^{\alpha}$-regions of the modified D- and L-Asp amino acids of molecules **1** and **17.**

Synthesis of modified peptides

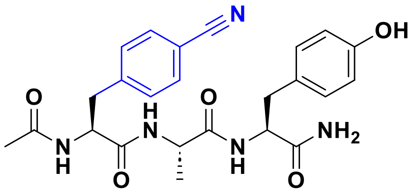

1

The modified peptide was synthesized based on the general protocols **2a-d** with NMR yield 44% (isolated yield – 4.6 mg, 33%), white amorphous solid.

^{1}H NMR (700 MHz, DMSO-d$_6$) δ: 8.24 (d, *J*=7.2 Hz, 1H), 8.14 (d, *J*=8.5 Hz, 1H), 7.72 (dd, *J*=8.4, 2.5 Hz, 3H), 7.43 (d, *J*=8.0 Hz, 2H), 7.32 (d, *J*=2.2 Hz, 1H), 7.06 (d, *J*=2.1 Hz, 1H), 6.99 (d, *J*=8.3 Hz, 2H), 6.62 (d, *J*=8.3 Hz, 2H), 4.54 (ddd, *J*=10.3, 8.4, 4.0 Hz, 1H), 4.32 (td, *J*=8.2, 5.2 Hz, 1H), 4.21 (p, *J*=7.1 Hz, 1H), 3.04 (dd, *J*=13.9 Hz, 4.0, 1H), 2.88 (dd, *J*=13.9, 5.2 Hz, 1H), 2.76 (dd, *J*=14.0, 10.5 Hz, 1H), 2.72 (dd, *J*=13.9, 8.3 Hz, 1H), 1.73 (s, 3H), 1.18 (d, *J*=7.1 Hz, 3H). **^{13}C NMR (176 MHz, DMSO-d$_6$) δ:** 172.8, 171.6, 170.8, 169.2, 155.8, 144.2, 131.9, 130.3, 130.1, 127.7, 119.0, 114.8, 109.1, 53.9, 53.2, 48.5, 22.4, 18.0; **HRMS ESI m/z** calculated for $C_{24}H_{28}N_5O_5$ [M+H]$^+$: 466.2090, measured: 466.2085 (error = -1.1 ppm).

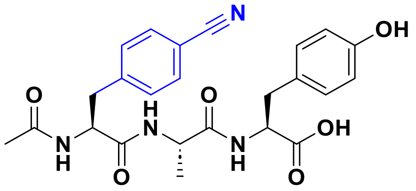

1-OH

The modified peptide was synthesized based on the general protocols **2a-d** with the exception of the use of Wang resin with yield 27%. The peptide was not further purified and characterized.

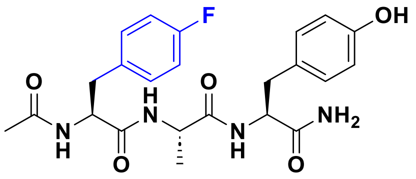

2

The modified peptide was synthesized based on the general protocols **2a-d** with NMR yield 38% (isolated yield – 4 mg, 29%), white amorphous solid.

^{1}H NMR (700 MHz, DMSO-d$_6$) δ: 8.19 (d, *J*=7.2 Hz, 1H), 8.09 (d, *J*=8.3 Hz, 1H), 7.70 (d, *J*=8.1 Hz, 1H), 7.31 (d, *J*=2.1 Hz, 1H), 7.26 (dd, *J*=8.5, 5.7 Hz, 2H), 7.09 – 7.03 (m, 3H), 6.99 (d, *J*=8.2 Hz, 2H), 6.62 (d, *J*=8.3 Hz, 2H), 4.46 (ddd, *J*=10.2, 8.3, 4.0 Hz, 1H), 4.31 (td, *J*=8.2, 5.2 Hz, 1H), 4.20 (p, *J*=7.1 Hz, 1H), 2.95 (dd, *J*=14.0, 4.1 Hz, 1H), 2.88 (dd, *J*=13.9, 5.2 Hz, 1H), 2.72 (dd, *J*=13.9, 8.3 Hz, 1H), 2.67 (dd, *J*=14.0, 10.3 Hz, 1H), 1.74 (s, 3H), 1.17 (d, *J*=7.1 Hz, 3H). **^{13}C NMR (176 MHz, DMSO-d$_6$) δ:** 172.7, 171.7, 171.2, 169.2, 161.6, 160.2, 155.8, 134.2, 131.0, 130.9, 130.1, 127.7, 114.8, 114.7, 114.6, 53.88, 53.85, 48.5, 40.0, 36.7, 36.6, 22.4, 18.0; **^{19}F NMR (470 MHz, DMSO-*d*$_6$) δ:** -120.0 (td, *J*=9.2, 4.9 Hz) **HRMS ESI m/z** calculated for $C_{23}H_{28}FN_4O_5$ [M+H]$^+$: 459.2044, measured: 459.2038 (error = -1.3 ppm).

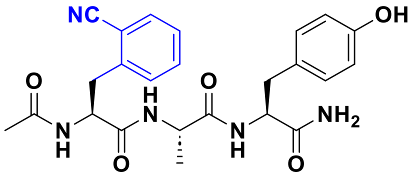

3

The modified peptide was synthesized based on the general protocols **2a-d** with NMR yield 39% (isolated yield – 4.2 mg, 30%), white amorphous solid.

^{1}H NMR (700 MHz, DMSO-d$_6$) δ: 8.17 (d, *J*=8.2 Hz, 1H), 8.10 (d, *J*=7.2 Hz, 1H), 7.80 (d, *J*=8.2 Hz, 1H), 7.73 (dd, *J*=7.8, 1.4 Hz, 1H), 7.54 (td, *J*=7.7, 1.4 Hz, 1H), 7.42 (d, *J*=7.3 Hz, 1H), 7.38 (td, *J*=7.6, 1.2 Hz, 1H), 7.27 (d, *J*=2.3 Hz, 1H), 7.02 (d, *J*=2.3 Hz, 1H), 7.00 (d, *J*=8.5 Hz, 2H), 6.63 (d, *J*=8.5 Hz, 2H), 4.67 (td, *J*=8.5, 5.6 Hz, 1H), 4.30 (td, *J*=8.2, 5.2 Hz, 1H), 4.24 - 4.16 (m, 1H), 3.20 (dd, *J*=14.4, 5.4 Hz, 1H), 3.00 (dd, *J*=14.4, 8.9 Hz, 1H), 2.87 (dd, *J*=13.9, 5.2 Hz, 1H), 2.72 (dd, *J*=13.9, 8.5 Hz, 1H), 1.77 (s, 3H), 1.17 (d, *J*=7.1 Hz, 3H). **^{13}C NMR (176 MHz, DMSO-d$_6$) δ:** 172.8, 171.7, 170.2, 169.5,

155.8, 141.2, 132.9, 132.7, 130.2, 130.1, 127.9, 127.3, 117.9, 114.9, 112.3, 54.2, 52.6, 48.5, 36.6, 35.9, 22.5, 18.0. **HRMS ESI m/z** calculated for $C_{24}H_{28}N_5O_5$ $[M+H]^+$: 466.2085, measured: 466.2087 (error = 0.4 ppm).

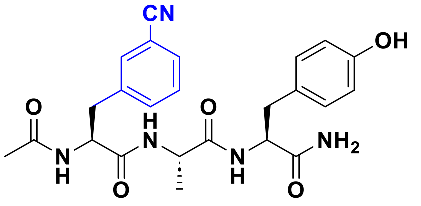

4

The modified peptide was synthesized based on the general protocols **2a-d** with NMR yield 31% (isolated yield – 3.1 mg, 22%), white amorphous solid.

^{1}H NMR (700 MHz, DMSO-d$_6$) δ: 8.22 (d, *J*=7.2 Hz, 1H), 8.12 (d, *J*=8.5 Hz, 1H), 7.73 (d, *J*=8.2 Hz, 1H), 7.70 (s, 1H), 7.66 (d, *J*=7.6 Hz, 1H), 7.57 (d, *J*=7.7 Hz, 1H), 7.45 (t, *J*=7.7 Hz, 1H), 7.31 (s, 1H), 7.05 (s, 1H), 6.99 (d, *J*=8.3 Hz, 1H), 6.62 (d, *J*=8.3 Hz, 1H), 4.55 – 4.49 (m, 1H), 4.35 – 4.29 (m, 1H), 4.21 (p, *J*=7.1 Hz, 1H), 3.01 (dd, *J*=13.8, 3.8 Hz, 1H), 2.88 (dd, *J*=13.9, 5.2 Hz, 1H), 2.74 (d, *J*=5.2 Hz, 1H), 2.73 – 2.70 (m, 1H), 1.73 (s, 3H), 1.18 (d, *J*=7.1 Hz, 3H). **^{13}C NMR (176 MHz, DMSO-d$_6$) δ:** 172.8, 171.6, 170.8, 169.3, 155.8, 139.8, 134.2, 132.8, 130.1, 129.2, 127.7, 119.0, 114.8, 110.9, 53.9, 53.4, 48.5, 40.0, 37.0, 36.7, 22.4, 18.0; **HRMS ESI m/z** calculated for $C_{24}H_{28}N_5O_5$ $[M+H]^+$: 466.2085, measured: 466.2095 (error = +2.1 ppm).

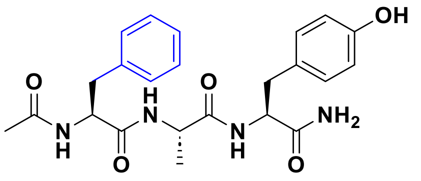

5

The modified peptide was synthesized based on the general protocols **2a-d** with NMR yield 37% (isolated yield – 3 mg, 23%), white amorphous solid.

^{1}H NMR (700 MHz, DMSO-d$_6$) δ: 8.18 (d, *J*=7.1 Hz, 1H), 8.09 (d, *J*=8.3 Hz, 1H), 7.69 (d, *J*=8.2 Hz, 1H), 7.29 (s, 1H), 7.24 (d, *J*=4.3 Hz, 2H), 7.17 (dt, *J*=8.7, 4.5 Hz, 1H), 7.05 (s, 1H), 6.99 (d, *J*=8.5 Hz, 2H), 6.63 (d, *J*=8.5 Hz, 2H), 4.48 (ddd, *J*=10.4, 8.3, 4.0 Hz, 1H), 4.31 (td, *J*=8.2, 5.3 Hz, 1H), 4.19 (p, *J*=7.2 Hz, 1H), 2.98 (dd, *J*=13.9, 4.0 Hz, 1H), 2.88 (dd, *J*=13.9, 5.2 Hz, 1H), 2.75 – 2.72 (m, 1H), 2.70 – 2.67 (m, 1H), 1.74 (s, 3H), 1.17 (d, *J*=7.1 Hz, 3H). **^{13}C NMR (176 MHz, DMSO-d$_6$) δ:** 172.7, 171.7, 171.4, 169.3, 155.8, 138.1, 130.1, 129.1, 128.0, 127.7, 126.2, 114.4, 55.3, 53.90, 53.87, 48.6, 40.0, 37.4, 36.7, 22.4, 17.9; **HRMS ESI m/z** calculated for $C_{23}H_{29}N_4O_5$ $[M+H]^+$: 441.2132, measured: 441.2144 (error = +2.7 ppm).

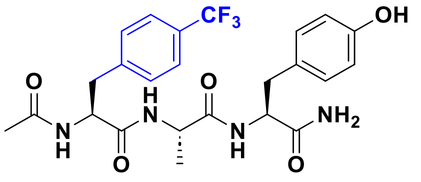

6

The modified peptide was synthesized based on the general protocols **2a-d** with NMR yield 49% (isolated yield – 5.5 mg, 36%), white amorphous solid.

^{1}H NMR (700 MHz, DMSO-d$_6$) δ: 8.25 (d, *J*=7.2 Hz, 1H), 8.14 (d, *J*=8.4 Hz, 1H), 7.72 (d, *J*=8.1 Hz, 1H), 7.61 (d, *J*=8.1 Hz, 2H), 7.46 (d, *J*=8.0 Hz, 2H), 7.32 (s, 1H), 7.06 (s, 1H), 6.99 (d, *J*=8.2 Hz, 2H), 6.62 (d, *J*=8.2 Hz, 2H), 4.58 – 4.52 (m, 1H), 4.32 (q, *J*=8.0 Hz, 1H), 4.21 (p, *J*=7.0 Hz, 1H), 3.05 (dd, *J*=13.9, 3.7 Hz, 1H), 2.88 (dd, *J*=14.0, 5.1 Hz, 1H), 2.78 (dd, *J*=13.7, 10.5 Hz, 1H), 2.73 (dd, *J*=13.8, 8.4 Hz, 1H), 1.74 (s, 3H), 1.18 (d, *J*=7.1 Hz, 3H); **^{13}C NMR (176 MHz, DMSO-d$_6$) δ:** 172.7, 171.7, 171.0, 169.3, 155.8, 143.1, 130.1, 130.0, 127.7, 124.80, 124.78, 114.8, 53.9, 53.4, 48.6, 40.0, 37.3, 36.7, 22.4, 17.9; **^{19}F NMR (470 MHz, DMSO-*d*$_6$) δ:** -63.7 (s) **HRMS ESI m/z** calculated for $C_{24}H_{28}F_3N_4O_5$ $[M+H]^+$: 509.2006, measured: 509.2018 (error = +2.4 ppm).

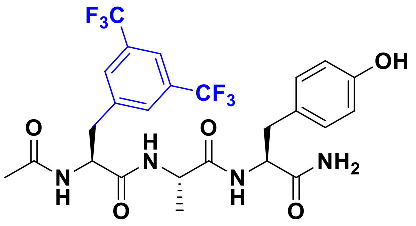

7

The modified peptide was synthesized based on the general protocols **2a-d** with NMR yield 22% (isolated yield – 2 mg, 12%), white amorphous solid.

^{1}H NMR (700 MHz, DMSO-d$_6$) δ: 8.27 (d, *J*=7.2 Hz, 1H), 8.16 (d, *J*=8.7 Hz, 1H), 7.97 (s, 2H), 7.93 (s, 1H), 7.78 (d, *J*=8.2 Hz, 1H), 7.32 (s, 1H), 7.05 (s, 1H), 6.99 (d, *J*=8.4 Hz, 2H), 6.62 (d, *J*=8.4 Hz, 2H), 4.58 – 4.54 (m, 1H), 4.39 – 4.28 (m, 1H), 4.24 (p, *J*=7.2 Hz, 1H), 3.15 (dd, *J*=13.8, 3.5 Hz, 1H), 2.88 (dd, *J*=13.9, 5.3 Hz, 1H), 2.83 (dd, *J*=13.6, 11.0 Hz, 1H), 2.72 (dd, *J*=13.9, 8.2 Hz, 1H), 1.69 (s, 3H), 1.19 (d, *J*=7.0 Hz, 3H). **^{13}C NMR (176 MHz, DMSO-d$_6$) δ:** 172.8, 171.7, 170.6, 169.2, 155.8, 141.6, 130.1, 129.7, 127.7, 122.4, 114.9, 53.9, 53.2, 48.6, 37.2, 36.7, 22.2, 18.1. **^{19}F NMR (470 MHz, DMSO-*d*$_6$) δ:** -64.3 (s) **HRMS ESI m/z** calculated for $C_{25}H_{27}F_6N_4O_5$ [M+H]$^+$: 577.1886, measured: 577.1887 (error = +0.2 ppm).

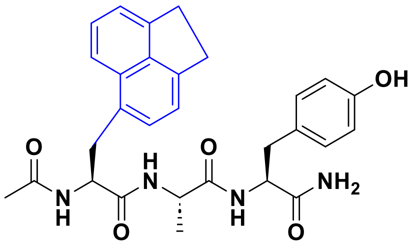

8

The modified peptide was synthesized based on the general protocols **2a-d** with NMR yield 14% (isolated yield – 1.9 mg, 12%), white amorphous solid.

^{1}H NMR (700 MHz, DMSO-d$_6$) δ: 8.20 (d, *J*=7.0 Hz, 1H), 8.14 (d, *J*=8.1 Hz, 1H), 7.82 (d, *J*=8.4 Hz, 2H), 7.72 (d, *J*=8.1 Hz, 1H), 7.50 – 7.42 (m, 1H), 7.37 – 7.25 (m, 2H), 7.17 (d, *J*=7.0 Hz, 1H), 7.05 (s, 1H), 7.00 (d, *J*=8.4 Hz, 2H), 6.63 (d, *J*=8.4 Hz, 2H), 4.63 – 4.56 (m, 1H), 4.30 (d, *J*=5.3 Hz, 1H), 4.24 – 4.16 (m, 1H), 3.35 – 3.26 (m, 4H), 3.11 – 3.05 (m, 1H), 2.88 (dd, *J*=13.7, 5.2 Hz, 1H), 2.72 (dd, *J*=13.7, 8.2 Hz, 1H), 1.71 (s, 3H), 1.18 (d, *J*=7.0 Hz, 3H). **^{13}C NMR (176 MHz, DMSO-d$_6$) δ:** 172.5, 171.8, 171.0, 169.5, 155.5, 133.0, 131.1, 127.6, 127.0, 125.2, 124.3, 120.3, 114.7, 108.3, 54.3, 53.0, 48.5, 40.0, 36.9, 35.0, 30.5, 30.2, 22.4, 17.9 **HRMS ESI m/z** calculated for $C_{29}H_{33}N_4O_5$ [M+H]$^+$: 517.2445, measured: 517.2446 (error = +0.2 ppm)..

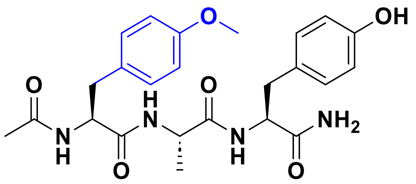

9

The modified peptide was synthesized based on the general protocols **2a-d** with NMR yield 14% (isolated yield – 1.5 mg, 11%), white amorphous solid.

^{1}H NMR (700 MHz, DMSO-d$_6$) δ: 8.17 (d, *J*=7.1 Hz, 1H), 8.05 (d, *J*=8.2 Hz, 1H), 7.68 (d, *J*=8.1 Hz, 1H), 7.29 (s, 1H), 7.16 (d, *J*=8.6 Hz, 2H), 7.06 (s, 1H), 6.98 (d, *J*=8.5 Hz, 2H), 6.81 (d, *J*=8.6 Hz, 2H), 6.63 (d, *J*=8.4 Hz, 2H), 4.44 – 4.39 (m, 1H), 4.31 (td, *J*=8.1, 5.3 Hz, 1H), 4.19 (p, *J*=7.0 Hz, 1H), 3.70 (s, 3H), 2.94 – 2.85 (m, 2H), 2.72 (dd, *J*=13.8, 8.2 Hz, 1H), 2.63 (dd, *J*=14.0, 10.4 Hz, 1H), 1.74 (s, 3H), 1.17 (d, *J*=7.1 Hz, 3H). **^{13}C NMR (176 MHz, DMSO-d$_6$) δ:** 172.7, 171.7, 171.5, 169.3, 157.7, 155.8, 130.2, 130.1, 130.0, 129.9, 127.7, 114.8, 113.4, 54.9, 54.2, 53.9, 48.6, 40.0, 36.7, 36.6, 22.5, 17.9. **HRMS ESI m/z** calculated for $C_{24}H_{31}N_4O_6$ [M+H]$^+$: 471.2238, measured: 471.2242 (error = +0.8 ppm).

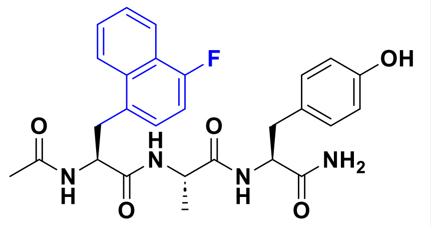

10

The modified peptide was synthesized based on the general protocols **2a-d** with NMR yield 30% (isolated yield – 3.8 mg, 25%), white amorphous solid.

^{1}H NMR (700 MHz, DMSO-d$_6$) δ: 8.25 (d, *J*=7.0 Hz, 1H), 8.19 (d, *J*=8.3 Hz, 1H), 8.05 (d, *J*=8.0 Hz, 1H), 7.74 (d, *J*=8.2 Hz, 1H), 7.69 – 7.58 (m, 2H), 7.35 – 7.32 (m, 1H), 7.31 (s, 1H), 7.18 (dd, *J*=10.5, 8.0 Hz, 1H), 7.05 (s, 1H), 7.01 (d, *J*=8.4 Hz, 2H), 6.63 (d, *J*=8.4 Hz, 2H), 4.61 (td, *J*=9.0, 4.7 Hz, 1H), 4.36 – 4.29 (m, 1H), 4.22 (p, *J*=7.1 Hz, 1H), 3.47 (dd, *J*=14.5, 4.6 Hz, 2H), 3.11 (dd, *J*=14.4, 9.7 Hz, 1H), 2.89 (dd, *J*=13.9, 5.1 Hz, 1H), 2.73 (dd, *J*=13.9, 8.5 Hz, 1H), 1.71 (s, 3H), 1.18 (d, *J*=7.1 Hz, 3H). **^{13}C NMR (176 MHz, DMSO-d$_6$) δ:** 172.8, 171.6, 171.1, 169.3, 155.8, 132.8, 130.1, 127.8, 127.1, 126.2, 124.4, 120.3, 114.9, 108.8, 54.0, 53.2, 48.6, 40.0, 36.7, 34.2, 22.4, 17.9; **^{19}F NMR (470 MHz, DMSO-*d$_6$*) δ:** -128.6 (dd, *J*=11.1, 5.6 Hz) **HRMS ESI m/z** calculated for $C_{27}H_{30}FN_4O_5$ [M+H]$^+$: 509.2195, measured: 509.2209 (error = +2.7 ppm).

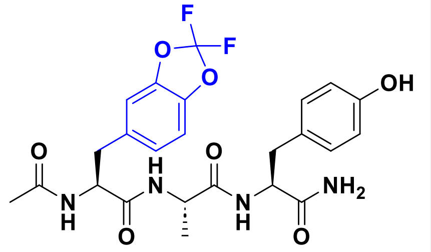

11

The modified peptide was synthesized based on the general protocols **2a-d** with NMR yield 37% (isolated yield – 3.5 mg, 22%), white amorphous solid.

^{1}H NMR (700 MHz, DMSO-d$_6$) δ: 8.19 (d, *J*=7.1 Hz, 1H), 8.07 (d, *J*=8.4 Hz, 1H), 7.73 (d, *J*=8.2 Hz, 1H), 7.31 (s, 1H), 7.28 – 7.24 (m, 2H), 7.05 (s, 1H), 7.04 (s, 1H), 6.99 (d, *J*=8.3 Hz, 2H), 6.62 (d, *J*=8.4 Hz, 2H), 4.56 – 4.42 (m, 1H), 4.38 – 4.28 (m, 1H), 4.21 (p, *J*=7.2 Hz, 1H), 2.97 (dd, *J*=13.9, 4.0 Hz, 1H), 2.87 (dd, *J*=13.9, 5.3 Hz, 1H), 2.73 (t, *J*=6.9 Hz, 1H), 2.70 (d, *J*=10.6 Hz, 1H), 1.74 (s, 3H), 1.17 (d, *J*=7.1 Hz, 3H); **^{13}C NMR (176 MHz, DMSO-d$_6$) δ:** 172.7, 171.7, 170.9, 169.3, 155.8, 142.4, 141.3, 135.0, 130.1, 127.7, 125.1, 114.8, 110.8, 109.4, 55.3, 53.9, 53.8, 48.5, 40.0, 37.2, 36.7, 22.4, 18.0; **^{19}F NMR (470 MHz, DMSO-*d$_6$*) δ:** -52.1 (s) **HRMS ESI m/z** calculated for $C_{24}H_{27}F_2N_4O_7$ [M+H]$^+$: 521.1842, measured: 521.1842 (error = 0.0 ppm).

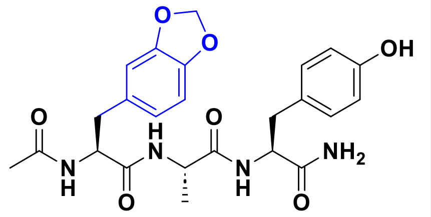

12

The modified peptide was synthesized based on the general protocols **2a-d** with NMR yield 14% (isolated yield – 1.4 mg, 10%), white amorphous solid.

^{1}H NMR (700 MHz, DMSO-d$_6$) δ: 8.16 (d, *J*=7.1 Hz, 1H), 8.05 (d, *J*=8.3 Hz, 1H), 7.68 (d, *J*=8.1 Hz, 1H), 7.29 (s, 1H), 7.05 (s, 1H), 6.98 (d, *J*=8.5 Hz, 2H), 6.85 (d, *J*=1.6 Hz, 1H), 6.77 (d, *J*=7.9 Hz, 1H), 6.69 (dd, *J*=8.0, 1.7 Hz, 1H), 6.62 (d, *J*=8.5 Hz, 2H), 4.44 – 4.38 (m, 1H), 4.31 (td, *J*=7.9, 5.2 Hz, 1H), 4.19 (p, *J*=7.2 Hz, 1H), 2.94 – 2.81 (m, 2H), 2.72 (dd, *J*=13.9, 8.2 Hz, 1H), 2.60 (dd, *J*=14.0, 10.4 Hz, 1H), 1.75 (s, 3H), 1.17 (d, *J*=7.1 Hz, 3H). **^{13}C NMR (176 MHz, DMSO-d$_6$) δ:** 172.7, 171.7, 171.4, 169.3, 158.3, 158.0, 155.7, 146.9, 145.6, 131.8, 130.1, 127.7, 122.2, 114.8, 109.5, 107.8, 100.6, 54.2, 53.9, 48.5, 37.1, 36.7, 22.5, 17.9. **HRMS ESI m/z** calculated for $C_{24}H_{29}N_4O_7$ [M+H]$^+$: 485.2031, measured: 485.2035 (error = +0.8 ppm).

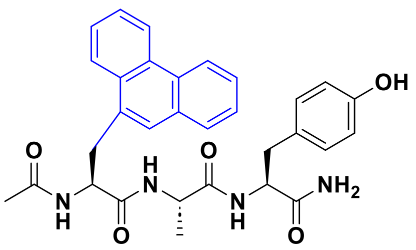

13

The modified peptide was synthesized based on the general protocols **2a-d** with NMR yield 26% (isolated yield – 2.8 mg, 17%), white amorphous solid.

^{1}H NMR (700 MHz, DMSO-d$_6$) δ: 8.88 – 8.84 (m, 1H), 8.79 (d, *J*=8.2 Hz, 1H), 8.28 (d, *J*=6.9 Hz, 1H), 8.27 – 8.26 (m, 1H), 8.24 (d, *J*=8.2 Hz, 1H), 7.84 (d, *J*=7.6 Hz, 1H), 7.76 (d, *J*=8.2 Hz, 1H), 7.72 – 7.68 (m, 2H), 7.64 (t, *J*=7.0 Hz, 1H), 7.61 (t, *J*=7.3 Hz, 1H), 7.29 (s, 1H), 7.05 (s, 1H), 7.00 (d, *J*=8.4 Hz, 1H), 6.63 (d, *J*=8.4 Hz, 1H), 4.73 (td, *J*=9.3, 4.5 Hz, 1H), 4.34 – 4.28 (m, 1H), 4.22 (p, *J*=7.0 Hz, 1H), 3.58 (dd, *J*=14.5, 4.4 Hz, 2H), 3.20 (dd, *J*=14.6, 9.8 Hz, 1H), 2.88 (dd, *J*=13.9, 5.2 Hz, 1H), 2.71 (dd, *J*=13.9, 8.4 Hz, 1H), 1.71 (s, 2H), 1.20 (d, *J*=7.1 Hz, 2H); **^{13}C NMR (176 MHz, DMSO-d$_6$) δ:** 172.8, 171.7, 171.3, 169.4, 155.8, 132.0, 131.1, 130.8, 130.1, 130.0, 129.3, 128.1, 127.8, 127.5, 126.9, 126.8, 126.4, 124.5, 123.3, 122.7, 114.8, 55.3, 54.0, 52.9, 48.7, 40.0, 36.6, 34.9, 22.4, 17.8. **HRMS ESI m/z** calculated for $C_{31}H_{33}N_4O_5$ [M+H]$^+$: 541.2445, measured: 541.2446 (error = +0.2 ppm).

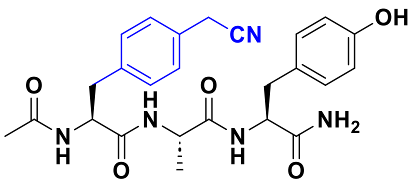

14

The modified peptide was synthesized based on the general protocols **2a-d** with NMR yield 20% (isolated yield – 2 mg, 14%), white amorphous solid.

^{1}H NMR (700 MHz, DMSO-d$_6$) δ: 8.20 (d, *J*=7.1 Hz, 1H), 8.08 (d, *J*=8.3 Hz, 1H), 7.70 (d, *J*=8.1 Hz, 1H), 7.30 (s, 1H), 7.26 (d, *J*=8.1 Hz, 2H), 7.22 (d, *J*=8.2 Hz, 2H), 7.05 (s, 1H), 6.99 (d, *J*=8.4 Hz, 2H), 6.63 (d, *J*=8.4 Hz, 2H), 4.51 – 4.45 (m, 1H), 4.34 – 4.28 (m, 1H), 4.20 (p, *J*=7.0 Hz, 1H), 3.97 (s, 2H), 2.97 (dd, *J*=13.9, 3.9 Hz, 1H), 2.88 (dd, *J*=13.9, 5.3 Hz, 1H), 2.71 (ddd, *J*=24.6, 13.9, 9.4 Hz, 2H), 1.73 (s, 3H), 1.17 (d, *J*=7.1 Hz, 3H). **^{13}C NMR (176 MHz, DMSO-d$_6$) δ:** 172.7, 171.7, 171.3, 169.3, 137.5, 130.1, 129.7, 129.0, 127.7, 119.3, 114.8, 53.9, 53.8, 48.6, 40.0, 37.0, 36.7, 22.4, 22.0, 17.9. **HRMS ESI m/z** calculated for $C_{25}H_{30}N_5O_5$ [M+H]$^+$: 480.2241, measured: 480.2245 (error = +0.8 ppm).

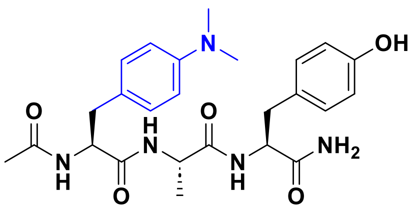

15

The modified peptide was synthesized based on the general protocols **2a-d** with NMR yield 27% (isolated yield – 2.6 mg, 18%), white amorphous solid.

^{1}H NMR (700 MHz, DMSO-d$_6$) δ: (mixtures of two conformers) 8.65 (d, *J*=7.4 Hz, 0.6H), 8.16 (d, *J*=7.0 Hz, 1H), 8.05 (d, *J*=8.1 Hz, 2H), 7.76 (d, *J*=8.0 Hz, 0.6H), 7.68 (d, *J*=8.1 Hz, 1H), 7.29 (s, 1H), 7.05 (s, 1H), 6.98 (d, *J*=8.4 Hz, 2H), 6.96 (d, *J*=8.5 Hz, 0.9H), 6.62 (d, *J*=8.5 Hz, 2H), 6.61 (d, *J*=8.5 Hz, 0.7H), 4.41 (m, 1H), 4.35 – 4.27 (m, 1.5H), 4.25 – 4.21 (m, 1H), 4.20 – 4.15 (m, 1.4H), 3.01 – 2.95 (m, 1H), 2.89 – 2.79 (m, 3H), 2.76 – 2.60 (m, 3H), 1.86 (s, 1H), 1.75 (s, 3H), 1.32 (d, *J*=7.2 Hz, 1H), 1.17 (d, *J*=7.1 Hz, 3H). **^{13}C NMR (176 MHz, DMSO-d$_6$) δ:** 172.6, 171.7, 171.3, 169.3, 157.0, 155.5, 130.2, 130.1, 130.0, 129.8, 127.7, 114.5, 113.4, 54.9, 54.2, 53.9, 48.6, 41.4, 41.2, 40.0, 36.7, 36.6, 22.5, 17.9 **HRMS ESI m/z** calculated for $C_{25}H_{34}N_5O_5$ [M+H]$^+$: 484.2554, measured: 484.2559 (error = +1.0 ppm).

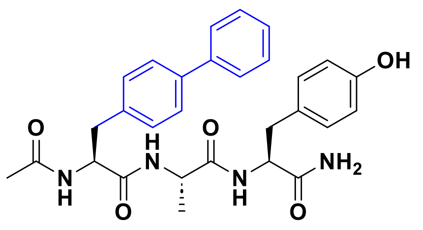

16

The modified peptide was synthesized based on the general protocols **2a-d** with NMR yield 38% (isolated yield – 4 mg, 26%), white amorphous solid.
^{1}H NMR (700 MHz, DMSO-*d*$_6$) δ: 8.24 (d, *J*=7.1 Hz, 1H), 8.14 (d, *J*=8.3 Hz, 1H), 7.71 (d, *J*=8.2 Hz, 1H), 7.64 (d, *J*=7.2 Hz, 2H), 7.56 (d, *J*=8.3 Hz, 2H), 7.44 (t, *J*=7.7 Hz, 2H), 7.34 (d, *J*=8.0 Hz, 3H), 7.31 (s, 1H), 7.06 (s, 1H), 6.99 (d, *J*=8.4 Hz, 2H), 6.63 (d, *J*=8.4 Hz, 2H), 4.55 – 4.50 (m, 1H), 4.35 – 4.30 (m, 1H), 4.22 (p, *J*=7.0 Hz, 1H), 3.02 (dd, *J*=14.0, 3.9 v, 1H), 2.88 (dd, *J*=13.9, 5.3 Hz, 1H), 2.79 – 2.70 (m, 2H), 1.76 (s, 3H), 1.19 (d, *J*=7.1 Hz, 3H). **^{13}C NMR (176 MHz, DMSO-*d*$_6$) δ:** 172.7, 171.7, 169.3, 155.8, 140.0, 138.0, 137.4, 130.1, 129.8, 128.9, 127.7, 127.2, 126.5, 126.3, 114.8, 53.9, 40.0, 37.0, 36.7, 22.5, 17.9. **HRMS ESI m/z** calculated for $C_{29}H_{33}N_4O_5$ $[M+H]^+$: 517.2445, measured: 517.2440 (error = -1.0 ppm).

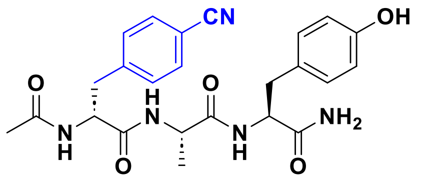

17

The modified peptide was synthesized based on the general protocols **2a-d** with NMR yield 31% (isolated yield – 2.7 mg, 19%), white amorphous solid.
^{1}H NMR (700 MHz, DMSO-*d*$_6$) δ: 8.43 (d, *J*=6.8 Hz, 1H), 8.35 (d, *J*=7.2 Hz, 1H), 7.75 (d, *J*=8.3 Hz, 2H), 7.71 (d, *J*=8.4 Hz, 1H), 7.44 (d, *J*=8.3 Hz, 2H), 7.08 (d, *J*=2.3 Hz, 1H), 7.04 – 6.97 (m, 3H), 6.63 (d, *J*=8.4 Hz, 2H), 4.51 – 4.46 (m, 1H), 4.21 (ddd, *J*=9.9, 8.4, 4.6 Hz, 1H), 4.05 (td, *J*=8.7, 7.9, 6.3 Hz, 1H), 2.98 (dd, *J*=13.6, 5.7 Hz, 1H), 2.93 (dd, *J*=13.9, 4.6 Hz, 1H), 2.87 (dd, *J*=13.6, 9.3 Hz, 1H), 2.72 (dd, *J*=14.0, 9.9 Hz, 1H), 1.79 (s, 3H), 1.03 (d, *J*=7.1 Hz, 3H). **^{13}C NMR (176 MHz, DMSO-d$_6$) δ:** 173.1, 171.6, 171.5, 170.1, 158.3, 155.7, 143.7, 132.0, 130.3, 130.0, 128.1, 119.0, 114.9, 109.2, 55.3, 54.4, 54.1, 48.8, 40.0, 37.2, 36.3, 22.5, 22.3, 17.5. **HRMS ESI m/z** calculated for $C_{24}H_{28}N_5O_5$ $[M+H]^+$: 466.2085, measured: 466.2089 (error = +0.9 ppm).

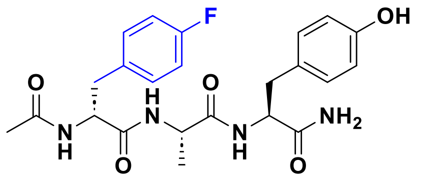

18

The modified peptide was synthesized based on the general protocols **2a-d** with NMR yield 32% (isolated yield – 3 mg, 22%), white amorphous solid.
^{1}H NMR (700 MHz, DMSO-*d*$_6$) δ: 8.42 (d, *J*=6.6 Hz, 1H), 8.32 (d, *J*=6.9 Hz, 1H), 7.68 (d, *J*=8.5 Hz, 1H), 7.27 (dd, *J*=8.6, 5.6 Hz, 2H), 7.14 – 7.07 (m, 3H), 7.01 (d, *J*=8.5 Hz, 2H), 6.97 (s, 1H), 6.63 (d, *J*=8.5 Hz, 2H), 4.45 – 4.37 (m, 1H), 4.19 (td, *J*=10.1, 9.3, 4.6 Hz, 1H), 4.09 – 3.98 (m, 1H), 2.94 (dd, *J*=14.0, 4.4 Hz, 1H), 2.87 (dd, *J*=13.7, 5.9 Hz, 1H), 2.78 (dd, *J*=13.7, 9.1 Hz, 1H), 2.74 – 2.69 (m, 1H), 1.80 (s, 3H), 1.01 (d, *J*=7.2 Hz, 3H). **^{13}C NMR (176 MHz, DMSO-d$_6$) δ:** 173.1, 172.0, 171.7, 170.2, 160.3, 133.7, 131.01, 130.96, 129.9, 128.2, 114.9, 114.8, 114.7, 55.3, 54.8, 54.5, 48.9, 40.0, 36.2, 22.3, 17.4. **^{19}F NMR (470 MHz, DMSO-*d*$_6$) δ:** -119.4 (ddd, *J*=14.6, 9.3, 5.5 Hz) **HRMS ESI m/z** calculated for $C_{23}H_{28}FN_4O_5$ $[M+H]^+$: 459.2038, measured: 459.2038 (error = 0.0 ppm).

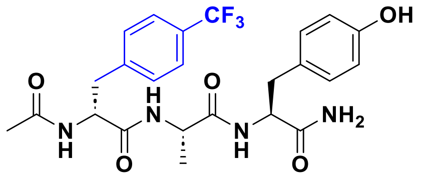

19

The modified peptide was synthesized based on the general protocols **2a-d** with NMR yield 28% (isolated yield – 3 mg, 20%), white amorphous solid.
^{1}H NMR (700 MHz, DMSO-*d*$_6$) δ: 8.44 (d, *J*=6.7 Hz, 1H), 8.35 (d, *J*=7.1 Hz, 1H), 7.70 (d, *J*=8.4 Hz, 1H), 7.64 (d, *J*=8.2 Hz, 2H), 7.47 (d, *J*=8.1 Hz, 2H), 7.08 (s, 1H), 7.02 – 6.98 (m, 3H), 6.63 (d, *J*=8.5 Hz, 2H), 4.52 – 4.45 (m, 1H), 4.20 (td, *J*=9.9, 9.2, 4.6 Hz, 1H), 4.04 (p, *J*=7.0 Hz, 1H), 2.98 (dd, *J*=13.5, 5.8 Hz, 1H), 2.95 – 2.92 (m, 1H), 2.89 (dd, *J*=13.6, 9.2 Hz, 1H), 2.72 (dd, *J*=13.9, 10.0 Hz, 1H), 1.80 (s, 3H), 1.01 (d, *J*=7.1 Hz, 3H). **^{13}C NMR (176 MHz, DMSO-d$_6$) δ:** 173.1, 171.68, 171.65, 170.2, 155.7, 142.6, 130.0, 129.9, 128.2, 125.2, 124.9, 123.7, 114.9, 54.4, 54.3, 48.9, 40.0, 36.9, 36.3, 22.3, 17.4. **^{19}F NMR (470 MHz, DMSO-*d*$_6$) δ:** 63.0 (s) **HRMS ESI m/z** calculated for $C_{24}H_{28}F_3N_4O_5$ [M+H]$^+$: 509.2006, measured: 509.2006 (error = 0.0 ppm).

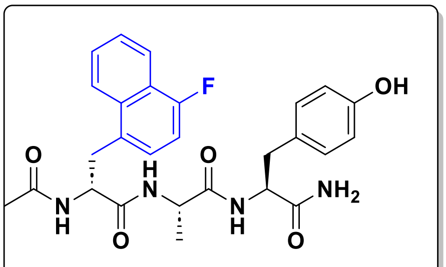

20

The modified peptide was synthesized based on the general protocols **2a-d** (initial scale was 100 μmol scale) with NMR yield 8% (isolated yield – 2.5 mg, 5%), white amorphous solid.
^{1}H NMR (700 MHz, DMSO-*d*$_6$) δ: 8.40 (s, 1H), 8.35 (d, *J*=6.7 Hz, 1H), 8.20 (d, *J*=8.2 Hz, 1H), 8.06 (d, 1H), 7.74 – 7.60 (m, 3H), 7.36 (dd, *J*=7.9, 5.6 Hz, 1H), 7.25 (dd, *J*=10.7, 7.9 Hz, 1H), 7.09 (s, 1H), 7.02 (s, 1H), 6.99 (d, *J*=8.5 Hz, 2H), 6.61 (d, *J*=8.5 Hz, 2H), 4.50 (q, *J*=7.1 Hz, 1H), 4.19 (td, *J*=9.8, 4.6 Hz, 1H), 3.99 (p, *J*=7.1 Hz, 1H), 3.37 (dd, *J*=14.0, 6.6 Hz, 1H), 3.23 (dd, *J*=14.1, 8.0 Hz, 1H), 2.93 (dd, *J*=13.8, 4.3 Hz, 1H), 2.71 (dd, *J*=13.9, 9.9 Hz, 1H), 1.81 (s, 3H), 0.92 (d, *J*=7.3 Hz, 3H). **^{13}C NMR (176 MHz, DMSO-*d*$_6$) δ:** 173.1, 171.8, 171.7, 170.2, 158.1, 156.1, 155.7, 132.9, 129.9, 129.8, 128.1, 127.3, 127.24, 127.17, 126.3, 124.3, 123.0, 122.9, 120.3, 114.9, 109.0, 108.8, 54.4, 54.1, 49.0, 40.1, 36.3, 33.9, 22.4, 17.3. **^{19}F NMR (470 MHz, DMSO-*d*$_6$) δ:** -127.51 (dd, *J*=9.9, 5.4 Hz) **HRMS ESI m/z** calculated for $C_{27}H_{30}FN_4O_5$ [M+H]$^+$: 509.2195, measured: 509.2195 (error = 0.0 ppm).

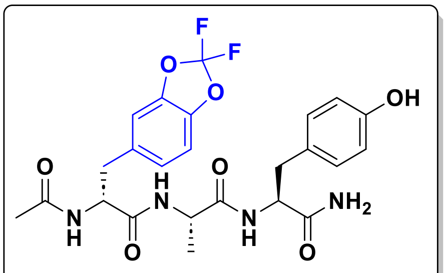

21

The modified peptide was synthesized based on the general protocols **2a-d** (initial scale was 100 μmol scale) with NMR yield 13% (isolated yield – 3 mg, 6%), white amorphous solid.
^{1}H NMR (700 MHz, DMSO-*d*$_6$) δ: 8.39 (d, *J*=6.9 Hz, 1H), 8.30 (d, *J*=7.2 Hz, 1H), 7.71 (d, *J*=8.4 Hz, 1H), 7.33 – 7.27 (m, 2H), 7.08 (s, 1H), 7.05 (dd, *J*=8.4, 1.5 Hz, 1H), 7.01 (d, *J*=8.5 Hz, 3H), 6.63 (d, *J*=8.5 Hz, 2H), 4.47 – 4.37 (m, 1H), 4.20 (td, *J*=9.6, 4.6 Hz, 1H), 4.04 (p, *J*=7.0 Hz, 1H), 3.04 – 2.87 (m, 2H), 2.81 (dd, *J*=13.6, 9.0 Hz, 1H), 2.72 (dd, *J*=13.9, 9.9 Hz, 1H), 1.80 (s, 3H), 1.02 (d, *J*=7.2 Hz, 3H). **^{13}C NMR (176 MHz, DMSO-*d*$_6$) δ:** 173.2, 171.8, 171.7, 170.3, 155.7, 142.5, 141.4, 134.6, 131.2, 130.0, 128.2, 125.2, 119.0, 114.9, 110.9, 109.6, 54.8, 54.5, 48.9, 36.8, 36.3, 22.4, 17.5. **^{19}F NMR (470 MHz, DMSO-*d*$_6$) δ:** -51.6 (s) **HRMS ESI m/z** calculated for $C_{24}H_{27}F_2N_4O_7$ [M+H]$^+$: 521.1842, measured: 521.1851 (error = +1.7 ppm).

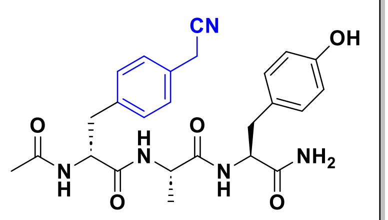

22

The modified peptide was synthesized based on the general protocols **2a-d** with NMR yield 18% (isolated yield – 2 mg, 14%), white amorphous solid.
^{1}H NMR (700 MHz, DMSO-*d*$_6$) δ: 8.44 (d, *J*=6.6 Hz, 1H), 8.31 (d, *J*=6.9 Hz, 1H), 7.68 (d, *J*=8.5 Hz, 1H), 7.30 – 7.21 (m, 4H), 7.09 (s, 1H), 7.01 (d, *J*=8.5 Hz, 2H), 6.96 (s, 1H), 6.63 (d, *J*=8.5 Hz, 2H), 4.47 – 4.37 (m, 1H), 4.23 – 4.16 (m, 1H), 4.05 – 3.99 (m, 1H), 3.98 (s, 2H), 2.94 (dd, *J*=14.0, 4.4 Hz, 1H), 2.89 (dd, *J*=13.7, 5.8 Hz, 1H), 2.79 (dd, *J*=13.7, 9.1 Hz,

1H), 2.73 (dd, *J*=13.9, 10.2 Hz, 1H), 1.80 (s, 3H), 1.01 (d, *J*=7.2 Hz, 3H). **^{13}C NMR (176 MHz, DMSO-d$_6$) δ:** 173.2, 172.2, 171.8, 170.4, 155.7, 137.1, 130.0, 129.8, 129.3, 128.2, 127.9, 119.4, 114.9, 54.8, 54.5, 49.0, 36.6, 36.2, 22.4, 22.1, 17.3. **HRMS ESI m/z** calculated for $C_{25}H_{30}N_5O_5$ [M+H]$^+$: 480.2241, measured: 480.2246 (error = +1.0 ppm).

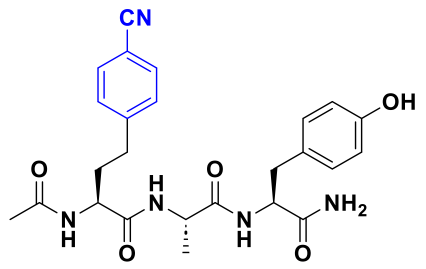

23

The modified peptide was synthesized based on the general protocols **2a-d** with NMR yield 34% (isolated yield – 4.1 mg, 28%), white amorphous solid.

^{1}H NMR (700 MHz, DMSO-d$_6$) δ: 8.13 (d, *J*=7.7 Hz, 1H), 8.11 (d, *J*=7.0 Hz, 1H), 7.73 (d, *J*=8.3 Hz, 2H), 7.69 (d, *J*=8.1 Hz, 1H), 7.38 (d, *J*=8.2 Hz, 2H), 7.27 (s, 1H), 7.04 (s, 1H), 6.97 (d, *J*=8.5 Hz, 2H), 6.61 (d, *J*=8.5 Hz, 2H), 4.31 – 4.25 (m, 1H), 4.24 – 4.20 (m, 1H), 4.17 (p, *J*=7.1 Hz, 1H), 2.86 (dd, *J*=13.9, 5.1 Hz, 1H), 2.71 (dd, *J*=13.9, 8.3 Hz, 1H), 2.68 – 2.59 (m, 2H), 1.91 (d, *J*=14.0 Hz, 1H), 1.87 (s, 3H), 1.82 – 1.75 (m, 1H), 1.16 (d, *J*=7.1 Hz, 3H). **^{13}C NMR (176 MHz, DMSO-d$_6$) δ:** 172.7, 171.8, 171.4, 169.5, 155.7, 147.6, 132.2, 130.1, 129.5, 127.7, 119.1, 114.8, 108.7, 54.0, 52.2, 48.6, 40.0, 36.6, 33.1, 31.5, 22.5, 17.8; **HRMS ESI m/z** calculated for $C_{25}H_{30}N_5O_5$ [M+H]$^+$: 480.2241, measured: 480.2241 (error = 0.0 ppm).

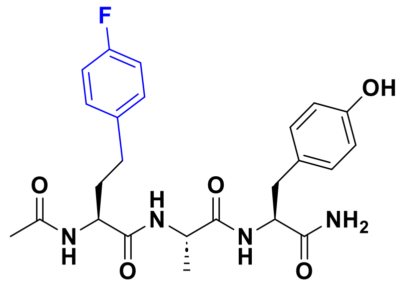

24

The modified peptide was synthesized based on the general protocols **2a-d** with NMR yield 35% (isolated yield – 3.8 mg, 27%), white amorphous solid.

^{1}H NMR (700 MHz, DMSO-d$_6$) δ: 8.13 (d, *J*=7.7 Hz, 1H), 8.08 (d, *J*=7.0 Hz, 1H), 7.70 (d, *J*=8.1 Hz, 1H), 7.25 (s, 1H), 7.20 (dd, *J*=8.6, 5.6 Hz, 2H), 7.08 (t, *J*=8.9 Hz, 2H), 7.04 (s, 1H), 6.97 (d, *J*=8.5 Hz, 2H), 6.61 (d, *J*=8.5 Hz, 2H), 4.27 (td, *J*=8.2, 5.3 Hz, 1H), 4.22 – 4.19 (m, 1H), 4.18 – 4.15 (m, 1H), 2.86 (dd, *J*=13.9, 5.2 Hz, 1H), 2.72 (dd, *J*=13.9, 8.4 Hz, 1H), 2.62 – 2.51 (m, 2H), 1.88 (s, 3H), 1.87 – 1.84 (m, 1H), 1.79 – 1.71 (m, 1H), 1.15 (d, *J*=7.1 Hz, 3H). **^{13}C NMR (176 MHz, DMSO-d$_6$) δ:** 172.8, 171.8, 171.6, 169.6, 161.3, 159.9, 155.7, 137.6, 130.1, 130.06, 130.03, 127.8, 115.0, 114.9, 114.8, 54.0, 52.3, 48.6, 40.0, 36.6, 33.7, 30.5, 22.5, 17.8. **^{19}F NMR (470 MHz, DMSO-*d*$_6$) δ:** -127.5 (dd, *J*=9.9, 5.4 Hz) **HRMS ESI m/z** calculated for $C_{24}H_{30}FN_4O_5$ [M+H]$^+$: 473.2195, measured: 473.2191 (error = -0.8 ppm).

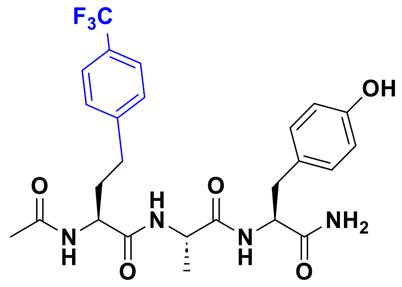

25

The modified peptide was synthesized based on the general protocols **2a-d** with NMR yield 26% (isolated yield – 2.8 mg, 18%), white amorphous solid.

^{1}H NMR (700 MHz, DMSO-*d*$_6$) δ: 9.14 (s, 1H), 8.15 (d, *J*=7.7 Hz, 1H), 8.11 (d, *J*=7.0 Hz, 1H), 7.70 (d, *J*=8.2 Hz, 1H), 7.63 (d, *J*=8.1 Hz, 2H), 7.41 (d, *J*=8.1 Hz, 2H), 7.26 (s, 1H), 7.05 (s, 1H), 6.97 (d, *J*=8.4 Hz, 2H), 6.61 (d, *J*=8.4 Hz, 2H), 4.28 – 4.24 (m, 1H), 4.23 (td, *J*=8.2, 5.4 Hz, 1H), 4.18 (p, *J*=7.1 Hz, 1H), 2.87 (dd, *J*=13.9, 5.2 Hz, 1H), 2.72 (dd, *J*=13.9, 8.3 Hz, 1H), 2.69 – 2.63 (m, 2H), 1.97 – 1.89 (m, 1H), 1.87 (s, 3H), 1.79 (m, 1H), 1.16 (d, *J*=7.2 Hz, 3H). **^{13}C NMR (176 MHz, DMSO-*d*$_6$) δ:** 172.8, 171.8, 171.5, 169.6, 155.8, 146.5, 130.1, 129.1, 127.8, 125.2, 125.14, 125.11, 114.8, 54.0, 52.2, 48.6, 40.1, 36.6, 33.2, 31.2, 22.5, 17.8. **^{19}F NMR (470 MHz, DMSO-*d*$_6$) δ:** -63.9 (s) **HRMS ESI m/z** calculated for $C_{25}H_{30}F_3N_4O_5$ [M+H]$^+$: 523.2163, measured: 523.2163 (error = 0.0 ppm).

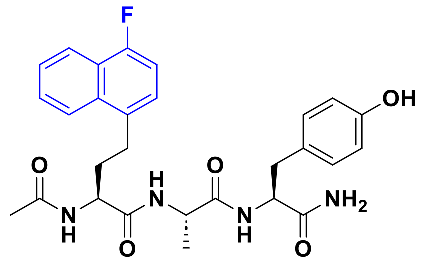

26

The modified peptide was synthesized based on the general protocols **2a-d** with NMR yield 29% (isolated yield – 3.1 mg, 20%), white amorphous solid.

^{1}H NMR (700 MHz, DMSO-*d*$_6$) δ: 8.25 (d, *J*=7.7 Hz, 1H), 8.16 (d, *J*=6.9 Hz, 1H), 8.15 – 8.12 (m, 1H), 8.06 (dd, *J*=7.0, 2.6 Hz, 1H), 7.77 (d, *J*=8.1 Hz, 1H), 7.66 – 7.56 (m, 2H), 7.32 (dd, *J*=7.8, 5.5 Hz, 1H), 7.25 – 7.18 (m, 2H), 7.01 (s, 1H), 6.96 (d, *J*=8.4 Hz, 2H), 6.59 (d, *J*=8.4 Hz, 2H), 4.36 (q, *J*=7.7 Hz, 1H), 4.27 (td, *J*=8.1, 5.5 Hz, 1H), 4.23 (p, *J*=7.1 Hz, 1H), 3.02 (t, *J*=8.2 Hz, 2H), 2.85 (dd, *J*=13.9, 5.3 Hz, 1H), 2.71 (dd, *J*=13.9, 8.2 Hz, 1H), 1.96 (td, *J*=13.7, 8.0 Hz, 1H), 1.91 (s, 3H), 1.88 (dd, *J*=13.9, 8.2 Hz, 1H), 1.18 (d, *J*=7.1 Hz, 3H). **^{13}C NMR (176 MHz, DMSO-d$_6$) δ:** 172.7, 171.9, 171.5, 169.6, 157.4, 156.0, 155.7, 134.1, 134.0, 132.43, 132.41, 130.1, 127.8, 127.1, 126.3, 125.7, 125.6, 124.3, 123.1, 123.0, 120.4, 114.8, 109.2, 109.1, 54.1, 52.5, 48.5, 40.0, 36.6, 33.3, 28.1, 22.6, 17.9. **^{19}F NMR (470 MHz, DMSO-*d*$_6$) δ:** -128.9 (dd, *J*=10.9, 5.6 Hz) **HRMS ESI m/z** calculated for $C_{28}H_{32}FN_4O_5$ [M+H]$^+$: 523.2351, measured: 523.2351 (error = 0.0 ppm).

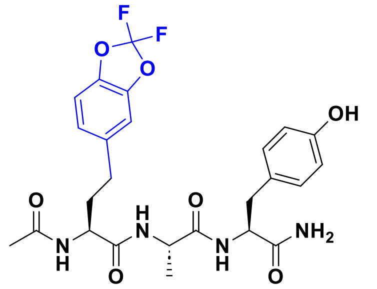

27

The modified peptide was synthesized based on the general protocols **2a-d** (initial scale was 100 μmol scale) with NMR yield 13% (isolated yield – 4.3 mg, 8%), white amorphous solid.

^{1}H NMR (700 MHz, DMSO-*d*$_6$) δ: 8.11 (d, *J*=7.7 Hz, 1H), 8.09 (d, *J*=7.1 Hz, 1H), 7.69 (d, *J*=8.2 Hz, 1H), 7.28 (d, *J*=8.3 Hz, 1H), 7.26 (s, 1H), 7.23 (s, 1H), 7.04 (s, 1H), 7.00 (d, *J*=8.3 Hz, 1.6, 1H), 6.97 (d, *J*=8.5 Hz, 2H), 6.60 (d, *J*=8.4 Hz, 2H), 4.28 (td, *J*=8.2, 5.4 Hz, 1H), 4.19 (dt, *J*=14.2, 7.7 Hz, 2H), 2.86 (dd, *J*=13.8, 5.2 Hz, 1H), 2.72 (dd, *J*=13.9, 8.4 Hz, 1H), 2.64 – 2.56 (m, 2H), 1.87 (s, 4H), 1.81 – 1.73 (m, 1H), 1.15 (d, *J*=7.1 Hz, 3H). **^{13}C NMR (176 MHz, DMSO-d$_6$):** 172.8, 171.8, 171.5, 169.5, 155.7, 142.7, 140.9, 138.5, 130.1, 127.8, 124.1, 114.8, 110.1, 109.7, 54.0, 52.0, 48.5, 40.1, 36.6, 33.7, 31.1, 22.5, 17.8. **^{19}F NMR (470 MHz, DMSO-*d*$_6$) δ:** -52.1 (s) **HRMS ESI m/z** calculated for $C_{25}H_{29}F_2N_4O_7$ [M+H]$^+$: 535.1999, measured: 535.1999 (error = 0.0 ppm).

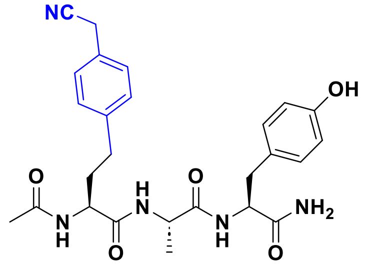

28

The modified peptide was synthesized based on the general protocols **2a-d** with NMR yield 35% (isolated yield – 3.4 mg, 23%), white amorphous solid.

^{1}H NMR (700 MHz, DMSO-*d*$_6$) δ: 8.14 (d, *J*=7.6 Hz, 1H), 8.08 (d, *J*=7.0 Hz, 1H), 7.70 (d, *J*=8.2 Hz, 1H), 7.25 (s, 1H), 7.24 (s, 2H), 7.20 (d, *J*=8.2 Hz, 2H), 7.04 (s, 1H), 6.97 (d, *J*=8.5 Hz, 2H), 6.61 (d, *J*=8.4 Hz, 2H), 4.28 (td, *J*=8.2, 5.4 Hz, 1H), 4.21 (dt, *J*=8.1, 4.1 Hz, 1H), 4.20 – 4.14 (m, 1H), 3.97 (s, 2H), 2.86 (dd, *J*=13.9, 5.2 Hz, 1H), 2.72 (dd, *J*=13.9, 8.4 Hz, 1H), 2.63 – 2.52 (m, 2H), 1.92 – 1.89 (m, 1H), 1.88 (s, 3H), 1.80 – 1.75 (m, 1H), 1.15 (d, *J*=7.2 Hz, 3H). **^{13}C NMR (176 MHz, DMSO-d$_6$) δ:** 172.8, 171.8, 171.6, 169.6, 155.7, 140.9, 130.1, 128.9, 128.6, 128.0, 127.8, 119.4, 114.8, 54.0, 52.4, 48.6, 40.0, 36.6, 33.5, 31.0, 22.5, 22.0, 17.8. **HRMS ESI m/z** calculated for $C_{26}H_{32}N_5O_5$ [M+H]$^+$: 494.2398, measured: 494.2398 (error = 0.0 ppm).

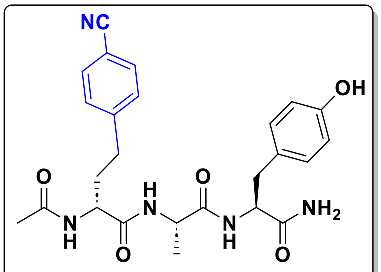

29

The modified peptide was synthesized based on the general protocols **2a-d** with NMR yield 79% (isolated yield – 9.36 mg, 65%), white amorphous solid.
^{1}H NMR (700 MHz, DMSO-*d*$_6$) δ: 8.48 (d, *J*=6.5 Hz, 1H), 8.34 (d, *J*=6.3 Hz, 1H), 7.75 (d, *J*=8.2 Hz, 2H), 7.69 (d, *J*=8.5 Hz, 1H), 7.40 (d, *J*=8.3 Hz, 2H), 7.09 (s, 1H), 7.01 (d, *J*=8.5 Hz, 2H), 6.93 (s, 1H), 6.63 (d, *J*=8.5 Hz, 2H), 4.19 (ddd, *J*=10.2, 8.6, 4.4 Hz, 1H), 4.16 – 4.12 (m, 1H), 4.02 (p, *J*=7.1 Hz, 1H), 2.96 (dd, *J*=14.0, 4.2 Hz, 1H), 2.74 (ddd, *J*=14.8, 10.1, 4.9 Hz, 2H), 2.64 (ddd, *J*=14.0, 9.9, 6.7 Hz, 1H), 1.88 (s, 3H), 1.84 (ddt, *J*=18.1, 8.2, 4.6 Hz, 2H), 1.09 (d, *J*=7.2 Hz, 3H). **^{13}C NMR (176 MHz, DMSO-d$_6$) δ:** 173.2, 172.6, 171.8, 171.5, 170.5, 155.7, 147.3, 132.3, 129.9, 129.5, 128.3, 119.0, 116.3, 114.9, 114.6, 108.8, 54.5, 53.0, 49.2, 36.1, 32.2, 31.5, 22.5, 22.4, 17.4. **HRMS ESI m/z** calculated for $C_{25}H_{30}N_5O_5$ $[M+H]^+$: 480.2241, measured: 480.2248 (error = +1.5 ppm).

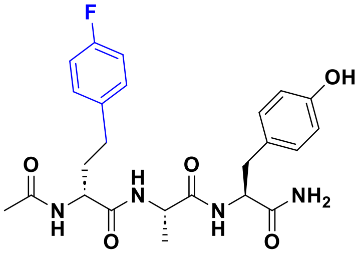

30

The modified peptide was synthesized based on the general protocols **2a-d** with NMR yield 52% (isolated yield – 5.7 mg, 40%), white amorphous solid.
^{1}H NMR (700 MHz, DMSO-*d*$_6$) δ: 8.50 (d, *J*=6.4 Hz, 1H), 8.34 (d, *J*=6.3 Hz, 1H), 7.69 (d, *J*=8.5 Hz, 1H), 7.22 (dd, *J*=8.5, 5.7 Hz, 2H), 7.11 (s, 1H), 7.10 – 7.07 (m, 2H), 7.02 (d, *J*=8.5 Hz, 2H), 6.91 (d, *J*=2.2 Hz, 1H), 6.64 (d, *J*=8.5 Hz, 2H), 4.18 (ddd, *J*=10.4, 8.5, 4.3 Hz, 1H), 4.13 (dt, *J*=8.2, 6.0 Hz, 1H), 4.02 (p, *J*=7.1 Hz, 1H), 2.96 (dd, *J*=14.0, 4.3 Hz, 1H), 2.74 (dd, *J*=14.0, 10.3 Hz, 1H), 2.64 (ddd, *J*=14.9, 10.1, 5.3 Hz, 1H), 2.57 – 2.50 (m, 1H), 1.88 (s, 3H), 1.87 – 1.77 (m, 2H), 1.10 (d, *J*=7.2 Hz, 3H). **^{13}C NMR (176 MHz, DMSO-d$_6$) δ:** 173.2, 172.8, 171.8, 171.5, 170.5, 161.4, 160.0, 158.4, 158.1, 155.7, 137.26, 137.25, 130.04, 129.99, 129.9, 128.3, 116.3, 115.1, 114.94, 114.89, 114.6, 55.3, 54.5, 53.2, 49.3, 40.0, 36.1, 32.9, 30.6, 22.5, 22.4, 17.3. **^{19}F NMR (470 MHz, DMSO-*d*$_6$) δ:** -119.6 (ddd, *J*=14.6, 9.0, 5.5 Hz) **HRMS ESI m/z** calculated for $C_{24}H_{30}FN_4O_5$ $[M+H]^+$: 473.2195, measured: 473.2201 (error = +1.3 ppm).

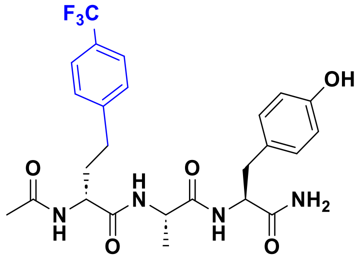

31

The modified peptide was synthesized based on the general protocols **2a-d** with NMR yield 75% (isolated yield – 9.4 mg, 60%), white amorphous solid.
^{1}H NMR (700 MHz, DMSO-*d*$_6$) δ: 8.15 (d, *J*=7.8 Hz, 1H), 8.11 (d, *J*=7.0 Hz, 1H), 7.70 (d, *J*=8.1 Hz, 1H), 7.63 (d, *J*=8.1 Hz, 2H), 7.41 (d, *J*=8.1 Hz, 2H), 7.26 (s, 1H), 7.04 (s, 1H), 6.97 (d, *J*=8.4 Hz, 2H), 6.61 (d, *J*=8.5 Hz, 2H), 4.31 – 4.26 (m, 1H), 4.25 – 4.21 (m, 1H), 4.17 (p, *J*=7.0 Hz, 1H), 2.87 (dd, *J*=13.9, 5.2 Hz, 1H), 2.72 (dd, *J*=14.0, 8.3 Hz, 1H), 2.69 – 2.59 (m, 2H), 1.96 – 1.89 (m, 1H), 1.88 (s, 3H), 1.83 – 1.73 (m, 1H), 1.16 (d, *J*=7.2 Hz, 3H). **^{13}C NMR (176 MHz, DMSO-d$_6$) δ:** 172.7, 171.8, 171.5, 169.5, 155.8, 146.5, 130.1, 129.1, 127.8, 125.1, 114.8, 54.0, 52.2, 48.6, 40.0, 36.6, 33.2, 31.2, 22.5, 17.8. **^{19}F NMR (470 MHz, DMSO-*d*$_6$) δ:** -64.4 (s) **HRMS ESI m/z** calculated for $C_{25}H_{30}F_3N_4O_5$ $[M+H]^+$: 523.2163, measured: 523.2163 (error = 0.0 ppm).

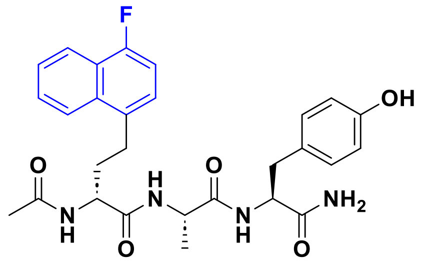

32

The modified peptide was synthesized based on the general protocols **2a-d** with NMR yield 34% (isolated yield – 4.1 mg, 26%), white amorphous solid.

^{1}H NMR (700 MHz, DMSO-*d*$_6$) δ: 8.54 (d, *J*=6.4 Hz, 1H), 8.42 (d, *J*=6.5 Hz, 1H), 8.10 (d, *J*=8.3 Hz, 1H), 8.07 (d, 1H), 7.73 (d, *J*=8.4 Hz, 1H), 7.69 – 7.58 (m, 2H), 7.33 (dd, *J*=7.8, 5.5 Hz, 1H), 7.25 (dd, *J*=10.6, 7.9 Hz, 1H), 7.09 (s, 1H), 7.02 (d, *J*=8.5 Hz, 2H), 6.95 (s, 1H), 6.64 (d, *J*=8.4 Hz, 2H), 4.29 (q, *J*=6.5 Hz, 1H), 4.20 (td, *J*=10.1, 4.4 Hz, 1H), 4.08 (p, *J*=7.1 Hz, 1H), 3.18 – 3.03 (m, 1H), 3.02 – 2.91 (m, 2H), 2.75 (dd, *J*=13.9, 10.3 Hz, 1H), 2.01 – 1.93 (m, 1H), 1.92 (s, 4H), 1.11 (d, *J*=7.2 Hz, 3H). **^{13}C NMR (176 MHz, DMSO-*d*$_6$) δ:** 173.2, 172.7, 171.8, 170.5, 157.6, 155.9, 155.7, 133.70, 133.67, 132.39, 132.37, 129.9, 128.3, 127.2, 126.4, 125.8, 125.7, 124.1, 124.0, 123.2, 123.1, 120.50, 120.47, 114.9, 109.3, 109.1, 54.5, 53.3, 49.2, 40.1, 36.1, 32.5, 28.2, 22.4, 17.4. **^{19}F NMR (470 MHz, DMSO-*d*$_6$) δ:** -128.1 (dd, *J*=10.0, 5.4 Hz) **HRMS ESI m/z** calculated for $C_{28}H_{32}FN_4O_5$ $[M+H]^+$: 523.2351, measured: 523.2351 (error = 0.0 ppm).

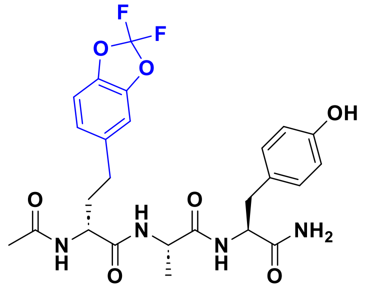

33

The modified peptide was synthesized based on the general protocols **2a-d** with NMR yield 21% (isolated yield – 2.4 mg, 15%), white amorphous solid.

^{1}H NMR (700 MHz, DMSO-*d*$_6$) δ: 8.48 (d, *J*=6.5 Hz, 1H), 8.33 (d, *J*=6.3 Hz, 1H), 7.68 (d, *J*=8.6 Hz, 1H), 7.30 (d, *J*=8.3 Hz, 1H), 7.26 (d, *J*=1.5 v, 1H), 7.09 (s, 1H), 7.01 (d, *J*=8.4 Hz, 3H), 6.91 (s, 1H), 6.63 (d, *J*=8.5 Hz, 2H), 4.18 (ddd, *J*=10.3, 8.6, 4.4 Hz, 1H), 4.13 – 4.09 (m, 1H), 4.01 (p, *J*=7.2 Hz, 1H), 2.96 (dd, *J*=14.0, 4.2 Hz, 1H), 2.77 – 2.70 (m, 1H), 2.67 (ddd, *J*=14.5, 9.5, 5.5 Hz, 1H), 2.61 – 2.55 (m, 1H), 1.88 (s, 3H), 1.87 – 1.79 (m, 2H), 1.09 (d, *J*=7.2 Hz, 3H). **^{13}C NMR (176 MHz, DMSO-d$_6$) δ:** 173.2, 172.7, 171.8, 170.5, 155.7, 142.8, 141.0, 138.1, 129.9, 128.3, 124.1, 114.9, 110.1, 109.7, 54.5, 52.9, 49.2, 40.0, 36.1, 32.7, 31.1, 22.4, 17.3. **^{19}F NMR (470 MHz, DMSO-*d*$_6$) δ:** -51.3 (d, *J*=2.7 Hz) **HRMS ESI m/z** calculated for $C_{25}H_{29}F_2N_4O_7$ $[M+H]^+$: 535.1999, measured: 535.1999 (error = 0.0 ppm).

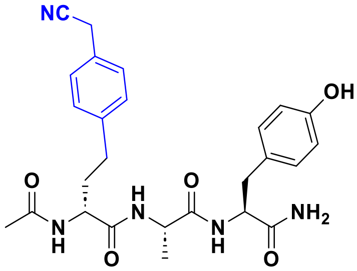

34

The modified peptide was synthesized based on the general protocols **2a-d** with NMR yield 61% (isolated yield – 7.9 mg, 53%), white amorphous solid.

^{1}H NMR (700 MHz, DMSO-*d*$_6$) δ: 8.51 (d, *J*=6.5 Hz, 1H), 8.35 (d, *J*=6.2 Hz, 1H), 7.69 (d, *J*=8.5 Hz, 1H), 7.26 (d, *J*=8.2 Hz, 2H), 7.21 (d, *J*=8.1 Hz, 2H), 7.09 (s, 1H), 7.02 (d, *J*=8.5 Hz, 2H), 6.91 (s, 1H), 6.64 (d, *J*=8.5 Hz, 2H), 4.21 – 4.16 (m, 1H), 4.15 – 4.12 (m, 1H), 4.02 (p, *J*=7.2 Hz, 1H), 3.98 (s, 2H), 2.96 (dd, *J*=14.0, 4.2 Hz, 1H), 2.75 (dd, *J*=14.0, 10.4 Hz, 1H), 2.65 (ddd, *J*=14.8, 10.3, 5.3 Hz, 2H), 2.54 (ddd, *J*=13.8, 10.1, 6.4 Hz, 2H), 1.89 (s, 3H), 1.88 – 1.78 (m, 3H), 1.10 (d, *J*=7.2 Hz, 3H). **^{13}C NMR (176 MHz, DMSO-d$_6$) δ:** 173.2, 172.8, 171.8, 171.5, 170.5, 155.7, 140.6, 129.9, 128.9, 128.8, 128.3, 128.1, 119.4, 116.3, 114.9, 114.7, 54.5, 53.2, 49.3, 36.1, 32.7, 31.0, 22.5, 22.4, 22.0, 17.3, **HRMS ESI m/z** calculated for $C_{26}H_{32}N_5O_5$ $[M+H]^+$: 494.2398, measured: 494.2405 (error = +1.4 ppm).

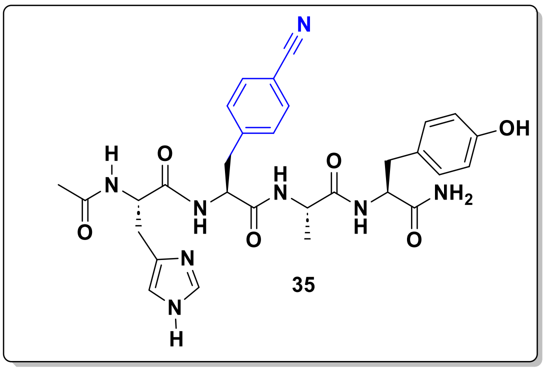

35

The modified peptide was synthesized based on the general protocols **2a-d** (initial scale was 100 μmol scale) with NMR yield 14% (isolated yield – 4.8 mg, 8%), white amorphous solid.
^{1}H NMR (700 MHz, DMSO-*d_6*) δ: 8.94 (s, 1H), 8.32 (d, *J*=7.3 Hz, 1H), 8.10 (d, *J*=8.2 Hz, 1H), 8.04 (d, *J*=8.3 Hz, 1H), 7.78 (d, *J*=8.1 Hz, 1H), 7.69 (d, *J*=8.3 Hz, 2H), 7.41 (d, *J*=8.3 Hz, 3H), 7.38 (s, 1H), 7.28 (s, 1H), 7.07 (s, 1H), 7.00 (d, *J*=8.5 Hz, 2H), 6.62 (d, *J*=8.5 Hz, 2H), 4.56 (td, *J*=9.9, 4.0 Hz, 1H), 4.54 – 4.49 (m, 1H), 4.38 – 4.32 (m, 1H), 4.24 (p, *J*=7.1 Hz, 1H), 3.10 (dd, *J*=14.0, 3.9 Hz, 1H), 2.96 (dd, *J*=15.2, 5.4 Hz, 1H), 2.91 – 2.86 (m, 1H), 2.85 – 2.79 (m, 2H), 2.73 (dd, *J*=13.9, 8.2 Hz, 1H), 1.79 (s, 3H), 1.19 (d, *J*=7.1 Hz, 3H). **^{13}C NMR (176 MHz, DMSO-d$_6$):** 172.7, 171.5, 170.3, 169.94, 169.40, 158.0, 157.8, 155.8, 143.7, 133.7, 131.9, 130.4, 130.1, 129.3, 127.7, 119.0, 116.7, 114.8, 109.2, 53.9, 53.1, 51.3, 48.5, 37.4, 36.8, 26.9, 22.5, 18.1. **HRMS ESI m/z** calculated for $C_{30}H_{35}N_8O_6$ [M+H]$^+$: 603.2674, measured: 603.2690 (error = +2.7 ppm).

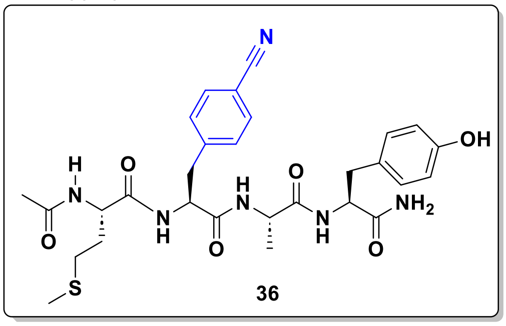

36

The modified peptide was synthesized based on the general protocols **2a-d** (initial scale was 100 μmol scale) with yield 17% (isolated yield – 5.9 mg, 10%), white amorphous solid.
^{1}H NMR (700 MHz, DMSO-*d_6*) δ: 8.10 (d, *J*=7.3 Hz, 1H), 8.01 (d, *J*=8.4 Hz, 1H), 7.97 (d, *J*=7.7 Hz, 1H), 7.76 (d, *J*=8.1 Hz, 1H), 7.68 (d, *J*=8.2 Hz, 2H), 7.41 (d, *J*=8.3 Hz, 2H), 7.32 (s, 1H), 7.05 (s, 1H), 6.99 (d, *J*=8.4 Hz, 2H), 6.62 (d, *J*=8.4 Hz, 2H), 4.60 – 4.51 (m, 1H), 4.36 – 4.29 (m, 1H), 4.27 – 4.15 (m, 2H), 3.10 (dd, *J*=13.9, 4.0 Hz, 1H), 2.91 – 2.81 (m, 2H), 2.72 (dd, *J*=13.9, 8.3 Hz, 1H), 2.31 (t, *J*=7.9 Hz 2H), 1.99 (s, 3H), 1.82 (s, 1H), 1.77 – 1.69 (m, 1H), 1.68 – 1.59 (m, 3H). **^{13}C NMR (176 MHz, DMSO-d$_6$):** 172.7, 171.6, 171.2, 170.2, 169.4, 155.8, 143.9, 131.8, 130.4, 130.1, 127.7, 119.0, 114.8, 114.7, 109.1, 69.8, 67.9, 53.9, 52.9, 52.0, 48.5, 37.3, 36.7, 31.6, 29.4, 22.5, 22.4, 18.0, 14.6. **HRMS ESI m/z** calculated for $C_{29}H_{37}N_6O_6S$ [M+H]$^+$: 597.2490, measured: 597.2503 (error = +2.2 ppm).

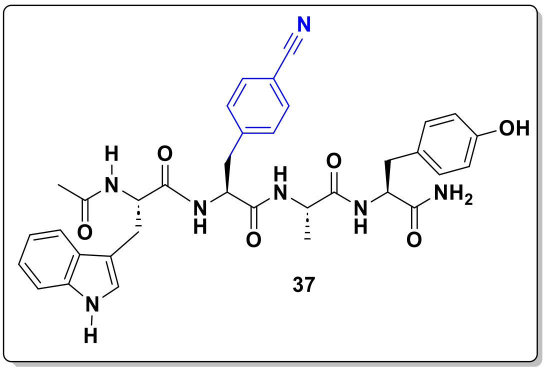

37

The modified peptide was synthesized based on the general protocols **2a-d** (initial scale was 100 μmol scale) with yield 18% (isolated yield – 7.3 mg, 11%), white amorphous solid.
^{1}H NMR (700 MHz, DMSO-*d_6*) δ: 10.76 (s, 1H), 8.13 (dd, *J*=7.6, 4.8 Hz, 2H), 7.93 (d, *J*=8.0 Hz, 1H), 7.78 (d, *J*=8.1 Hz, 1H), 7.67 (d, *J*=8.2 Hz, 2H), 7.54 (d, *J*=7.9 Hz, 1H), 7.40 (d, *J*=8.2 Hz, 2H), 7.33 (s, 1H), 7.30 (d, *J*=8.1 Hz, 1H), 7.06 (m, 3H), 7.00 (d, *J*=8.2 Hz, 2H), 6.96 (t, *J*=7.4 Hz, 1H), 6.62 (d, *J*=8.4 Hz, 2H), 4.57 (td, *J*=9.4, 4.3 Hz, 1H), 4.45 (td, *J*=8.9, 4.7 Hz 1H), 4.39 – 4.31 (m, 1H), 4.24 (dt, *J*=14.3, 7.0 Hz, 1H), 3.10 (dd, *J*=13.9, 4.1 Hz, 1H), 3.00 (dd, *J*=14.8, 4.5 Hz, 1H), 2.89 (d, *J*=8.1 Hz, 1H), 2.88 – 2.83 (m, 1H), 2.79 (dd, *J*=14.9, 9.3 Hz, 1H), 2.73 (dd, *J*=13.9, 8.2 Hz, 1H), 1.74 (s, 3H), 1.18 (d, *J*=7.1 Hz, 3H). **^{13}C NMR (176 MHz, DMSO-d$_6$):** 172.8, 171.7, 171.6, 170.2, 169.2, 158.2, 158.0, 155.8, 143.9, 136.0, 131.8, 130.4, 130.1, 127.7, 127.3, 123.4, 120.8, 119.0, 118.4, 118.2, 114.8, 111.3, 110.1, 109.1, 53.9, 53.3, 53.0, 48.5, 37.4, 36.7, 27.5, 22.5, 18.1. **HRMS ESI m/z** calculated for $C_{35}H_{38}N_7O_6$ [M+H]$^+$: 652.2878, measured: 652.2894 (error = +2.5 ppm).

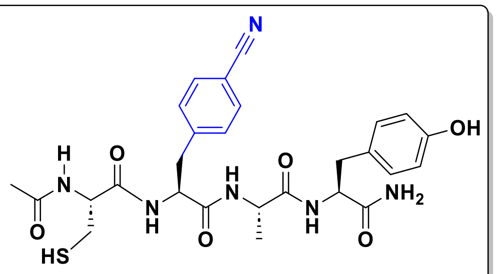

38

The modified peptide was synthesized based on the general protocols **2a-d** with NMR yield 19%. However, due to impurities that were difficult to separate further characterization was prohibited.

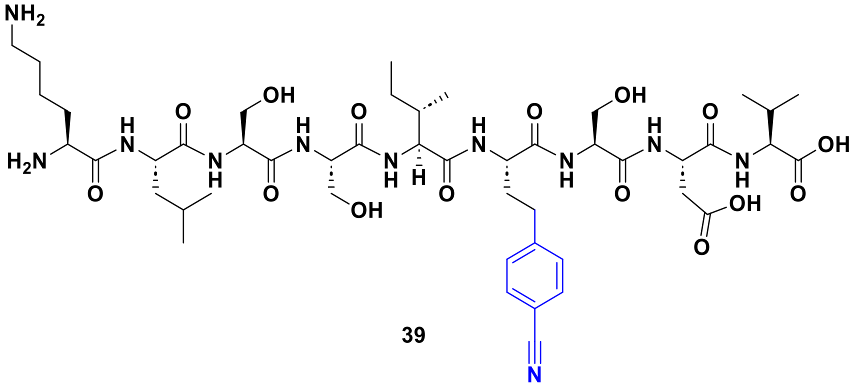

39

The modified peptide was synthesized based on the general protocols **2a-d** with isolated yield – 3.4 mg, 11%, white amorphous solid.

^{1}H NMR (700 MHz, DMSO-*d*$_6$) δ: 8.52 (d, *J*=7.9 Hz, 1H), 8.49 (d, *J*=7.9 Hz, 1H), 8.30 (d, *J*=8.0 Hz, 1H), 8.20 (d, *J*=7.6 Hz, 1H), 7.98 (d, *J*=7.5 Hz, 1H), 7.89 (d, *J*=7.8 Hz, 1H), 7.77 (d, *J*=8.3 Hz, 2H), 7.67 (d, *J*=8.5 Hz, 1H), 7.45 (d, *J*=8.3 v, 2H), 4.84 – 4.75 (m, 2H), 4.63 (p, *J*=8.4 Hz, 1H), 4.45 – 4.40 (m, 1H), 4.39 – 4.26 (m, 3H), 4.24 – 4.15 (m, 1H), 4.12 – 4.03 (m, 1H), 3.86 – 3.77 (m, 1H), 3.75 – 3.65 (m, 1H), 3.64 – 3.61 (m, 2H), 3.58 – 3.44 (m, 6H), 2.79 – 2.71 (m, 2H), 2.70 – 2.68 (m, 1H), 2.54 (d, *J*=7.6 Hz, 1H), 2.25 – 2.09 (m, 2H), 2.05 – 1.97 (m, 1H), 1.96 – 1.81 (m, 2H), 1.74 – 1.69 (m, 2H), 1.67 – 1.61 (m, 1H), 1.60 – 1.50 (m, 2H), 1.49 – 1.44 (m, 2H), 1.40 – 1.31 (m, 2H), 1.13 – 0.99 (m, 1H), 0.87 (t, *J*=7.1 Hz, 9H), 0.85 – 0.82 (m, 9H). **^{13}C NMR (176 MHz, DMSO-d$_6$):** 172.32, 172.27, 172.0, 171.0, 170.7, 170.6, 170.4, 168.8, 149.7, 132.73, 132.66, 128.0, 127.9, 120.2, 119.3, 118.2, 116.3, 114.3, 110.1, 62.4, 62.2, 62.0, 57.8, 57.5, 55.3, 52.9, 52.3, 51.6, 49.8, 41.2, 39.0, 37.1, 36.3, 32.4, 32.0, 31.0, 30.3, 26.9, 24.5, 23.6, 21.8, 21.4, 19.48, 19.45, 18.4, 15.9, 11.5. **HRMS ESI m/z** calculated for $C_{47}H_{76}N_{11}O_{15}$ [M+H]$^+$: 1034.5517, measured: 1034.5535 (error = +1.7 ppm).

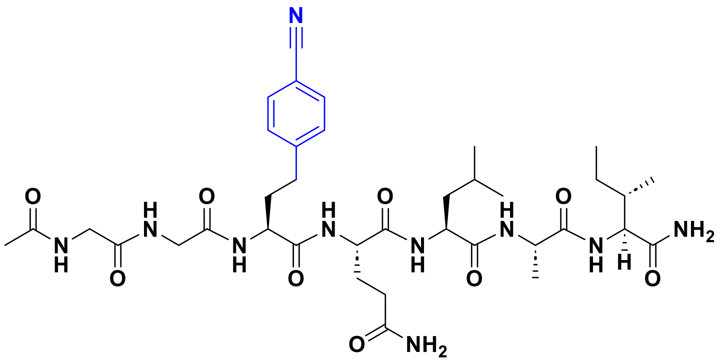

40

The modified peptide was synthesized based on the general protocols **2a-d** with NMR yield 18% (isolated yield – 3.1 mg, 13%), white amorphous solid.

^{1}H NMR (700 MHz, DMSO-*d*$_6$) δ: 8.20 (t, *J*=5.7 Hz, 1H), 8.16 (t, *J*=5.8 Hz, 1H), 8.11 (d, *J*=7.7 Hz, 1H), 8.05 (d, *J*=7.3 Hz, 1H), 8.03 (d, *J*=7.9 Hz, 1H), 7.89 (d, *J*=8.2 Hz, 1H), 7.74 (d, *J*=8.3 Hz, 2H), 7.51 (d, *J*=8.9 Hz, 1H), 7.40 (d, *J*=8.3 Hz, 2H), 7.33 (s, 1H), 7.27 (s, 1H), 7.02 (s, 1H), 6.78 (s, 1H), 4.34 – 4.24 (m, 3H), 4.19 (q, *J*=7.9 Hz, 1H), 4.11 – 4.07 (m, 1H), 3.78 – 3.73 (m, 2H), 3.69 (d, *J*=5.5 Hz, 2H), 2.71 – 2.57 (m, 2H), 2.15 – 2.01 (m, 2H), 1.99 – 1.87 (m, 1H), 1.85 (s, 3H), 1.84 – 1.80 (m, 1H), 1.76 – 1.62 (m, 2H), 1.61 – 1.52 (m, 1H), 1.46 – 1.35 (m, 3H), 1.17 (d, *J*=7.1 Hz, 3H), 1.07 – 0.97 (m, 1H), 0.81 (d, *J*=6.7 Hz, 9H), 0.76 (d, *J*=6.5 Hz, 3H). **^{13}C NMR (176 MHz, DMSO-d$_6$):** 174.3, 173.2, 172.3, 172.2, 171.6, 171.5, 170.5, 170.1, 169.4, 148.2, 132.7, 130.0, 119.5, 109.2, 57.0, 52.8, 52.5, 51.2, 48.6, 42.8, 42.6, 41.4, 37.3, 33.8, 31.9,

31.8, 28.0, 24.52, 24.49, 23.6, 23.0, 21.9, 18.0, 15.9, 11.7. **HRMS ESI m/z** calculated for $C_{37}H_{57}N_{10}O_9$ $[M+H]^+$: 785.4304, measured: 785.4319 (error = +1.9 ppm).

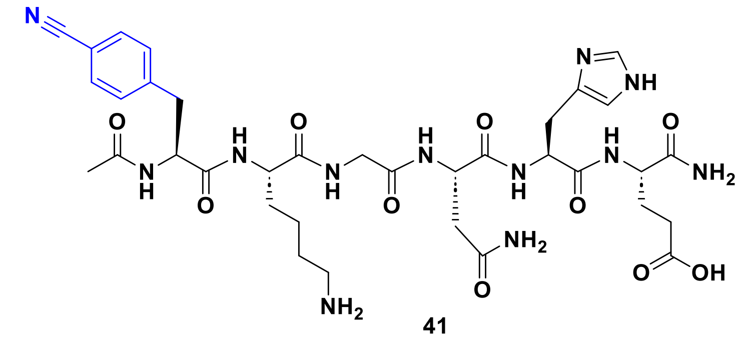

41

The modified peptide was synthesized based on the general protocols **2a-d** with NMR yield 22% (isolated yield – 2.2 mg, 9%), white amorphous solid.

^{1}H NMR (700 MHz, DMSO-d_6) δ: 8.95 (s, 1H), 8.31 (d, J=7.8 Hz, 1H), 8.23 (d, J=7.8 Hz, 1H), 8.18 – 8.12 (m, 2H), 8.02 (d, J=7.6 Hz, 1H), 7.73 (d, J=8.3 Hz, 2H), 7.72 (s, 1H), 7.51 (s, 1H), 7.44 (d, J=8.3 Hz, 2H), 7.35 (s, 1H), 7.30 (s, 1H), 7.17 (s, 1H), 6.99 (s, 1H), 4.61 – 4.56 (m, 1H), 4.55 – 4.52 (m, 1H), 4.51 – 4.47 (m, 1H), 4.27 – 4.24 (m, 1H), 4.14 (q, J=13.0, 8.7 Hz, 1H), 3.79 – 3.70 (m, 2H), 3.16 (dd, J=15.2, 5.0 Hz, 1H), 3.09 (dd, J=13.8, 4.0 Hz, 1H), 2.98 (dd, J=15.3, 8.2 Hz, 1H), 2.81 (dd, J=13.9, 10.5 Hz, 1H), 2.78 – 2.73 (m, 2H), 2.54 (dd, J=15.5, 6.8 Hz, 1H), 2.46 (dd, J=15.5, 6.7 Hz, 1H), 2.35 – 2.18 (m, 2H), 2.01 – 1.91 (m, 1H), 1.84 – 1.76 (m, 1H), 1.75 (s, 3H), 1.69 (m, 1H), 1.63 – 1.45 (m, 3H), 1.40 – 1.26 (m, 2H). **^{13}C NMR (176 MHz, DMSO-d$_6$):** 173.9, 173.4, 171.9, 171.7, 171.19, 171.15, 169.6, 169.5, 168.7, 144.1, 133.7, 131.9, 130.2, 129.2, 119.0, 117.8, 117.1, 115.8, 109.2, 53.4, 52.5, 52.3, 52.0, 49.7, 41.8, 38.7, 37.4, 36.9, 31.3, 30.2, 26.9, 26.6, 26.5, 22.4, 22.2. **HRMS ESI m/z** calculated for $C_{35}H_{49}N_{12}O_{10}$ $[M+H]^+$: 797.3689, measured: 797.3700 (error = +1.4 ppm).

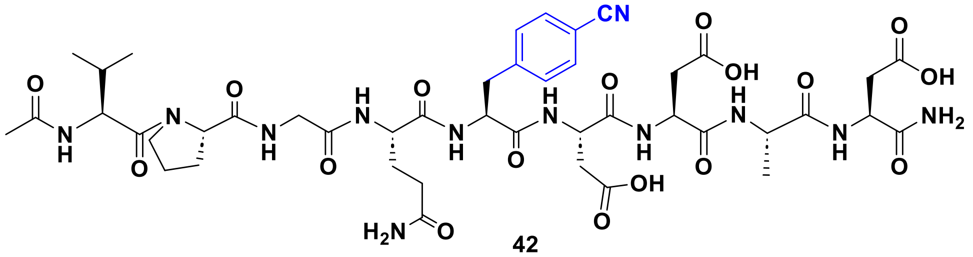

42

The modified peptide was synthesized based on the general protocols **2g** with isolated yield – 23 mg, 11%, white amorphous solid.

HRMS ESI m/z calculated for $C_{44}H_{62}N_{12}O_{17}$ $[M+2H]^{2+}$: 515.2178, measured: 515.2173 (error = -1.0 ppm).

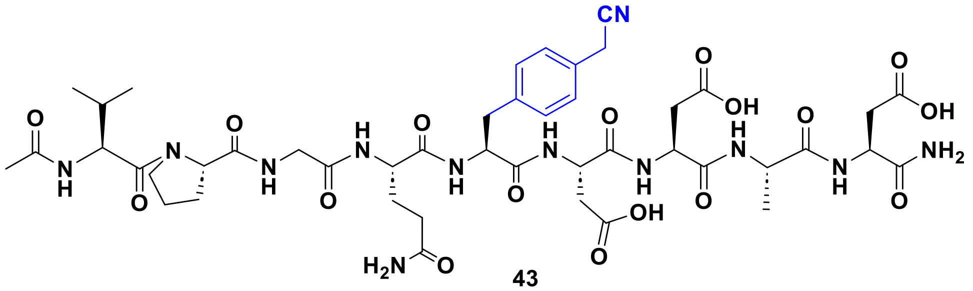

43

The modified peptide was synthesized based on the general protocols **2g** with isolated yield – 18.8 mg, 9%, white amorphous solid.

HRMS ESI m/z calculated for $C_{45}H_{63}O_{17}N_{12}$ $[M+H]^+$: 1043.4429, measured: 1043.4451 (error = +2.1 ppm).

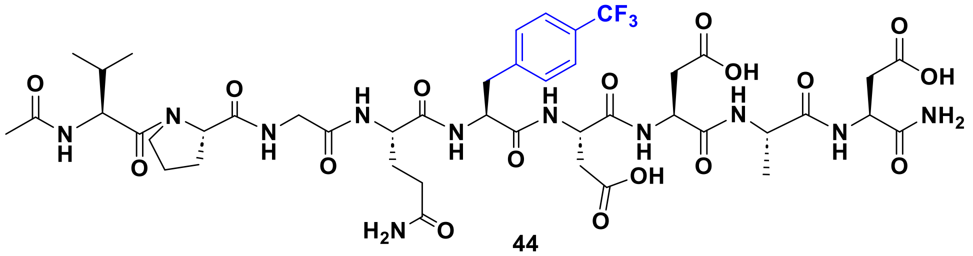

The modified peptide was synthesized based on the general protocols **2g** with isolated yield – 17.2 mg, 8%, white amorphous solid.
^{19}F NMR (470 MHz, DMSO-*d*$_6$) δ: -63.3 (s) **HRMS ESI m/z** calculated for $C_{44}H_{61}O_{17}N_{11}F_3$ $[M+H]^+$: 1072.4194, measured: 1072.4218 (error = +2.2 ppm).

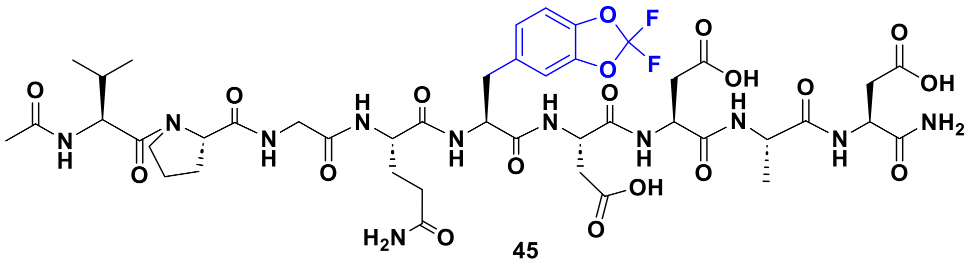

The modified peptide was synthesized based on the general protocols **2g** with isolated yield – 10.8 mg, 5%, white amorphous solid.
^{19}F NMR (470 MHz, DMSO-*d*$_6$) δ: -52.0 (d, *J*=5.2 Hz) **HRMS ESI m/z** calculated for $C_{44}H_{60}O_{19}N_{11}F_2$ $[M+H]^+$: 1084.4030, measured: 1084.4055 (error = +2.3 ppm).

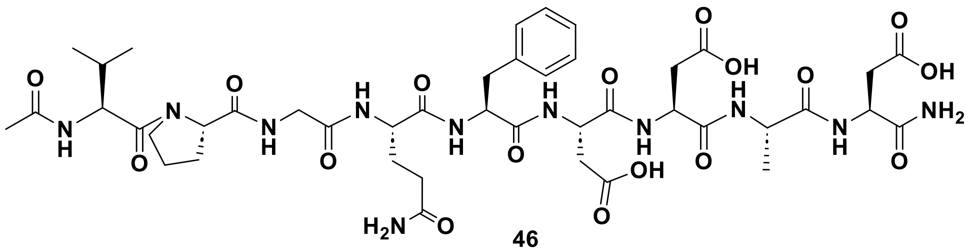

Peptide was prepared as described in Krzyzanowski et al.[3]

Purity data:

Compound **1**

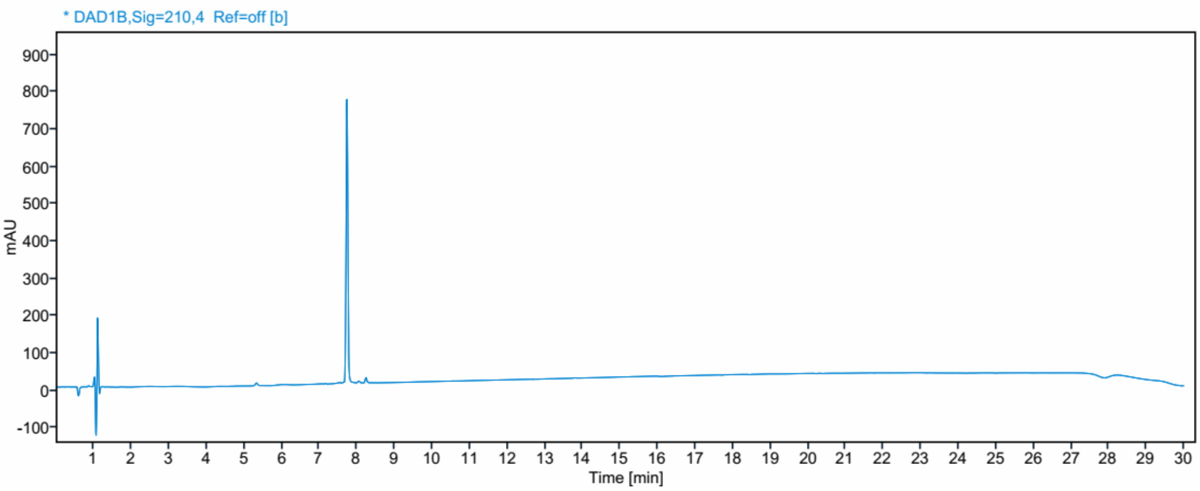

Compound **2**

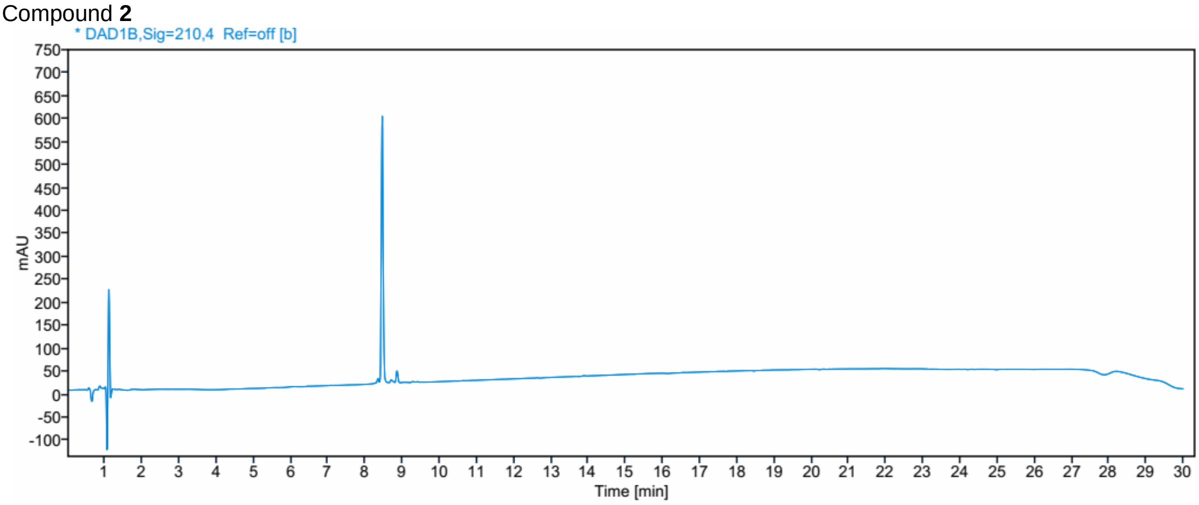

Compound **3**

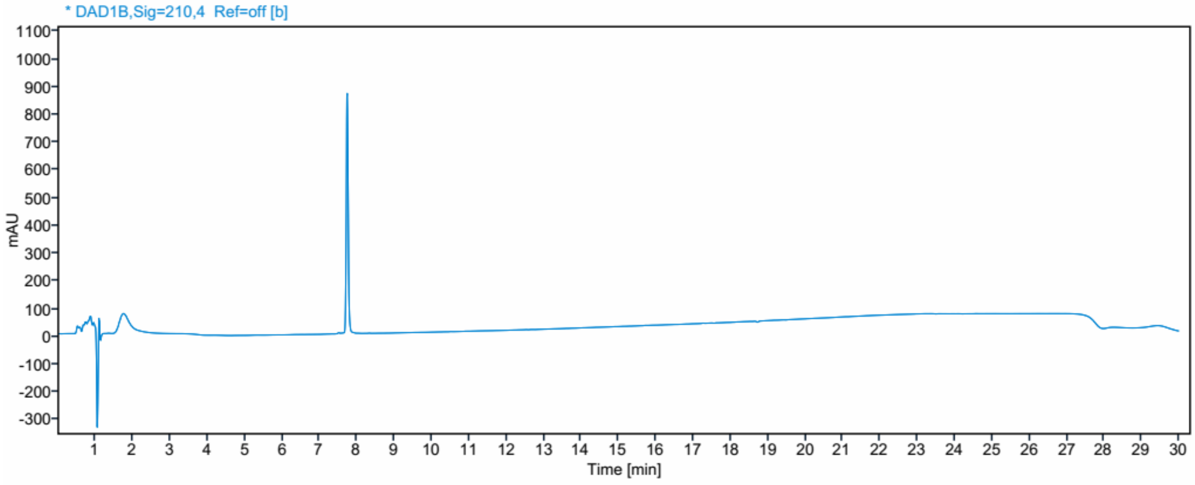

Compound **4**

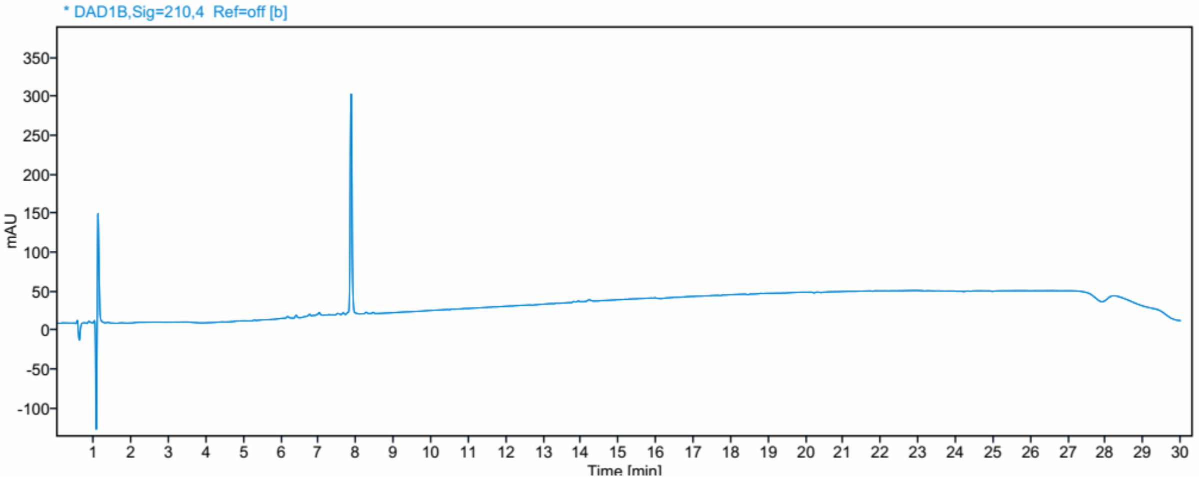

Compound **5**

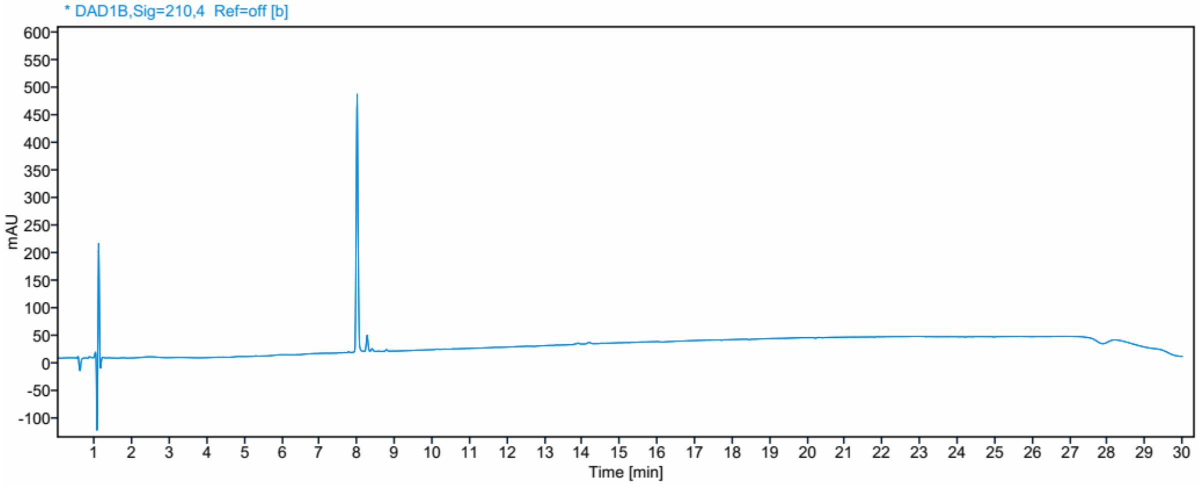

Compound **6**

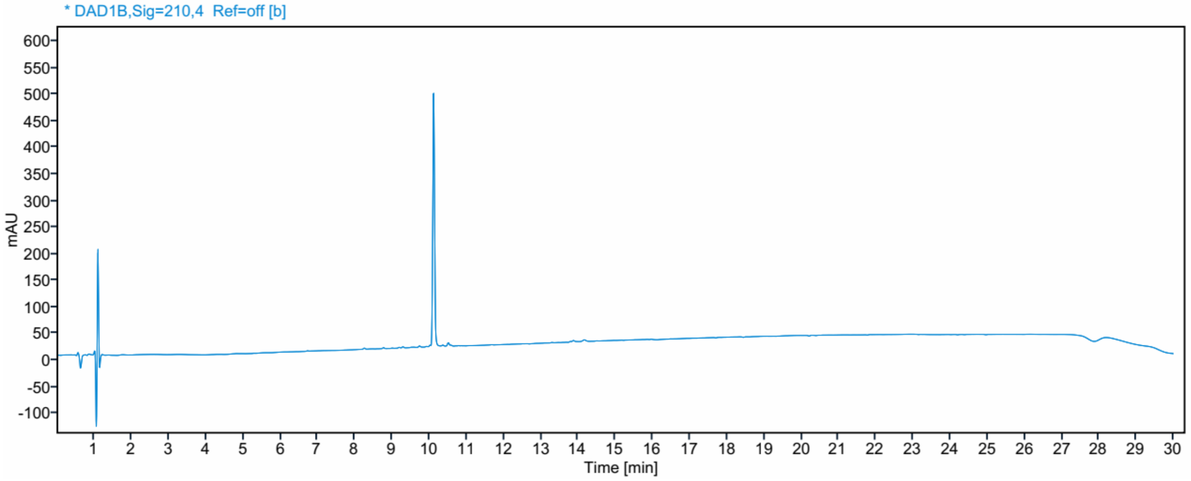

Compound **7**

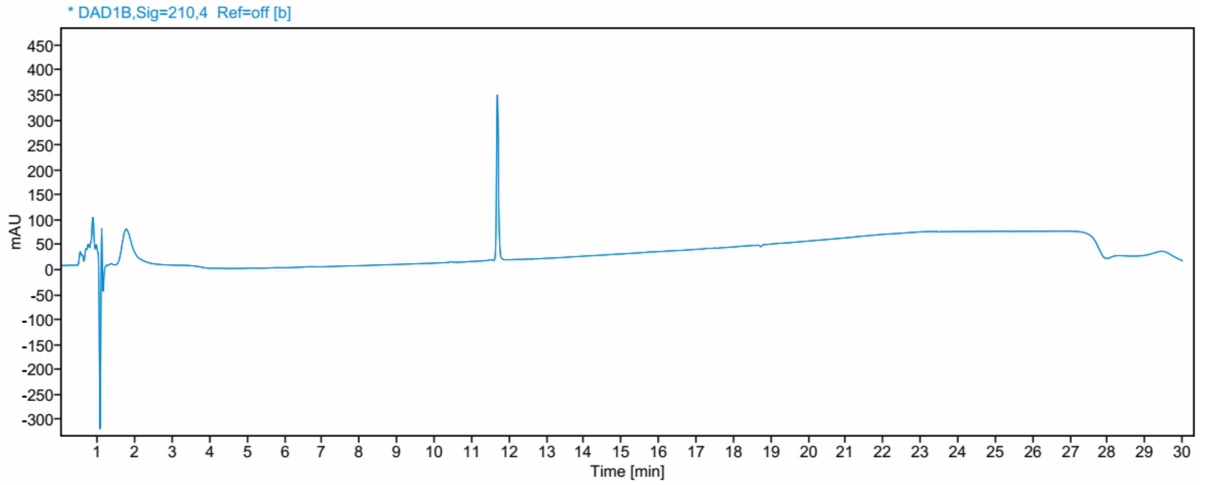

Compound **8**

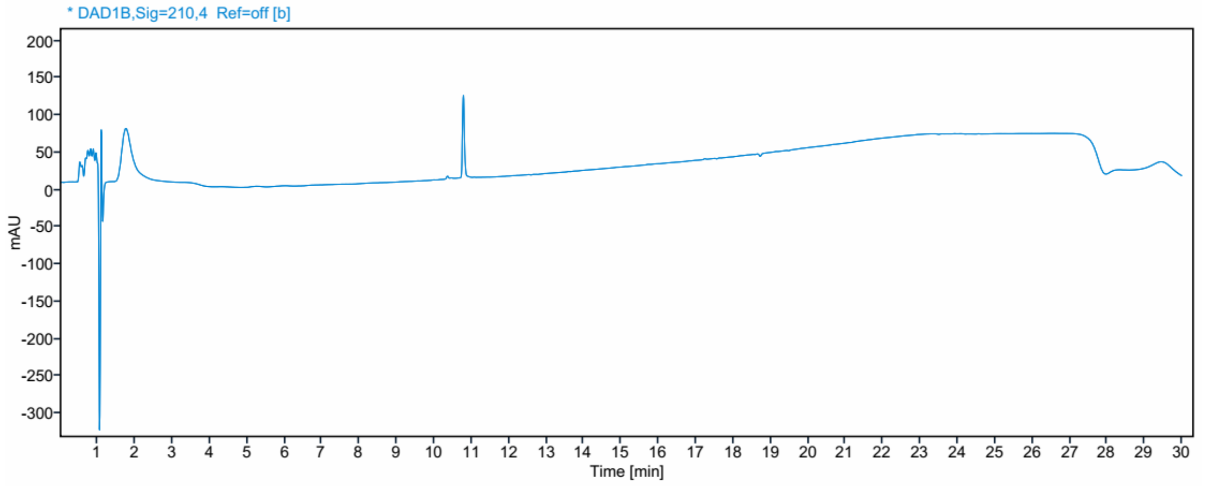

Compound **9**

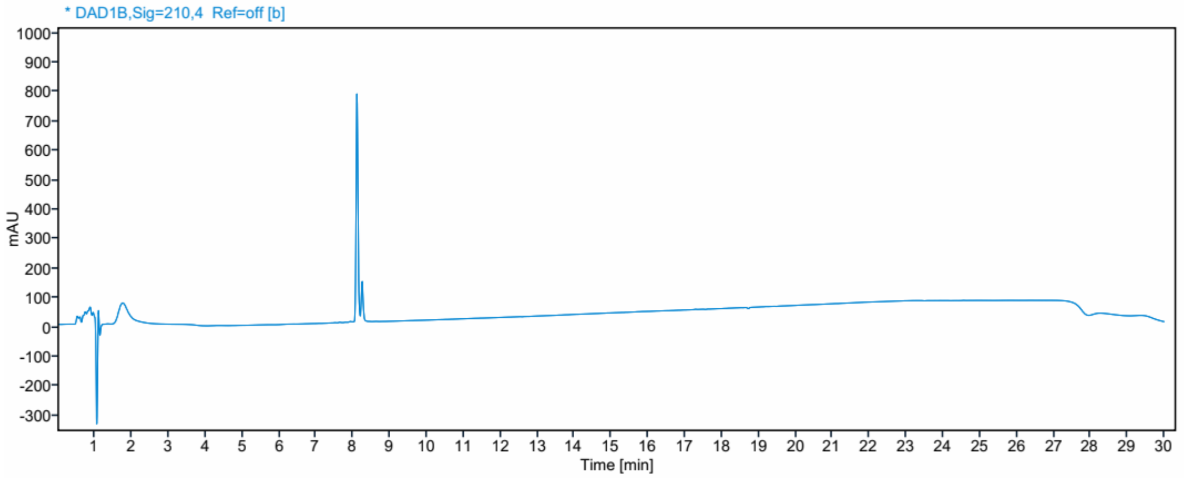

Compound **10**

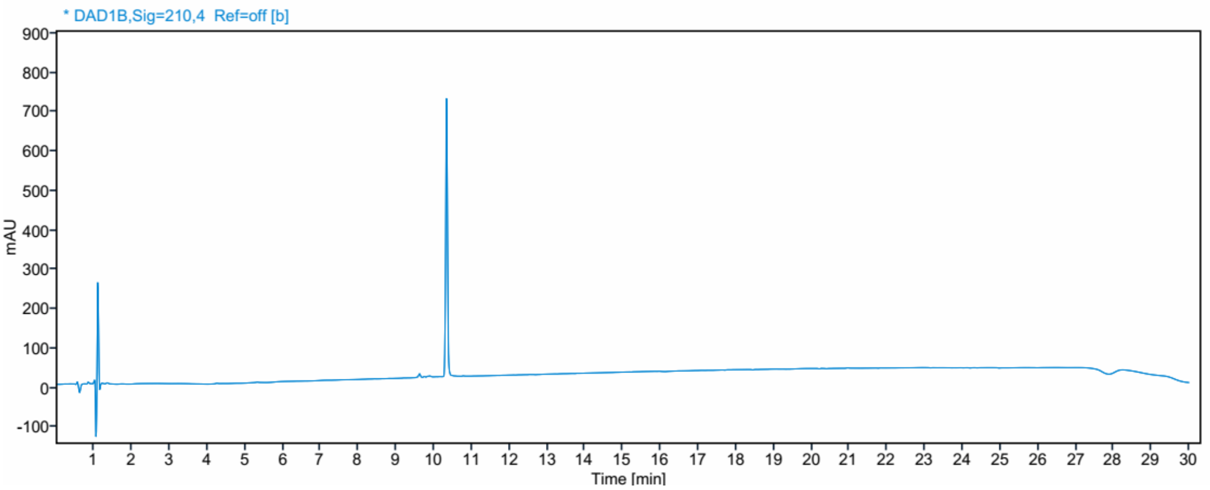

Compound **11**

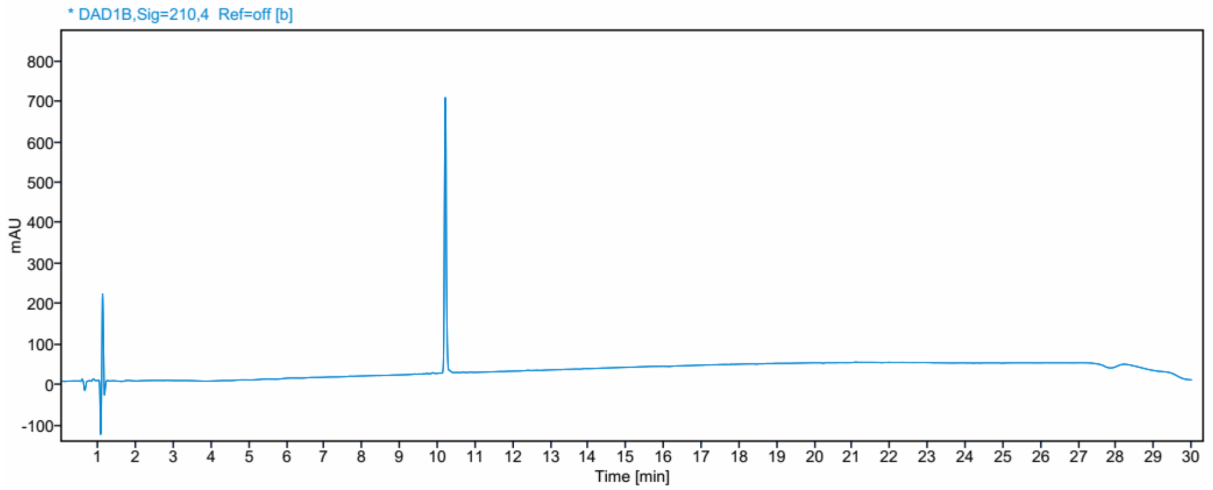

Compound **12**

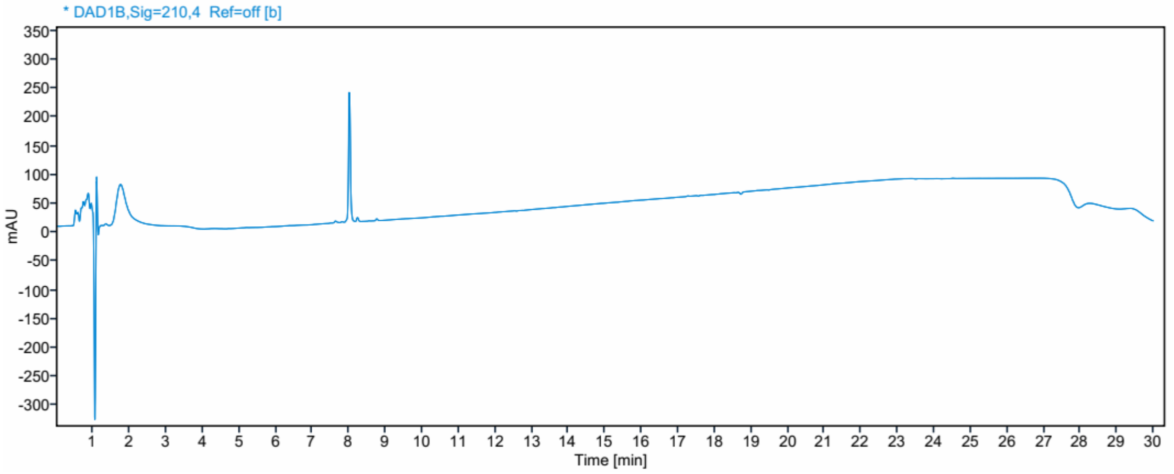

Compound **13**

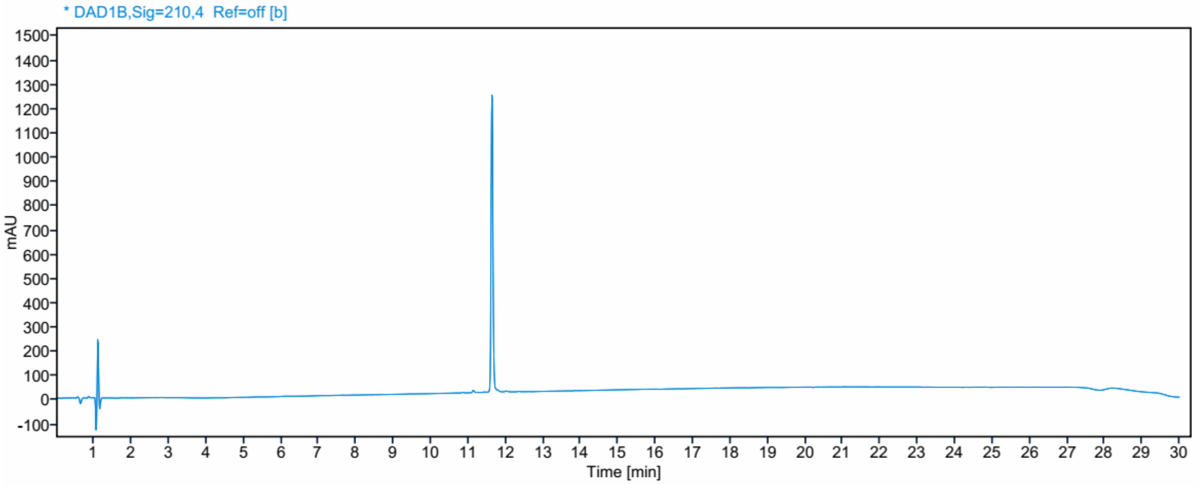

Compound **14**

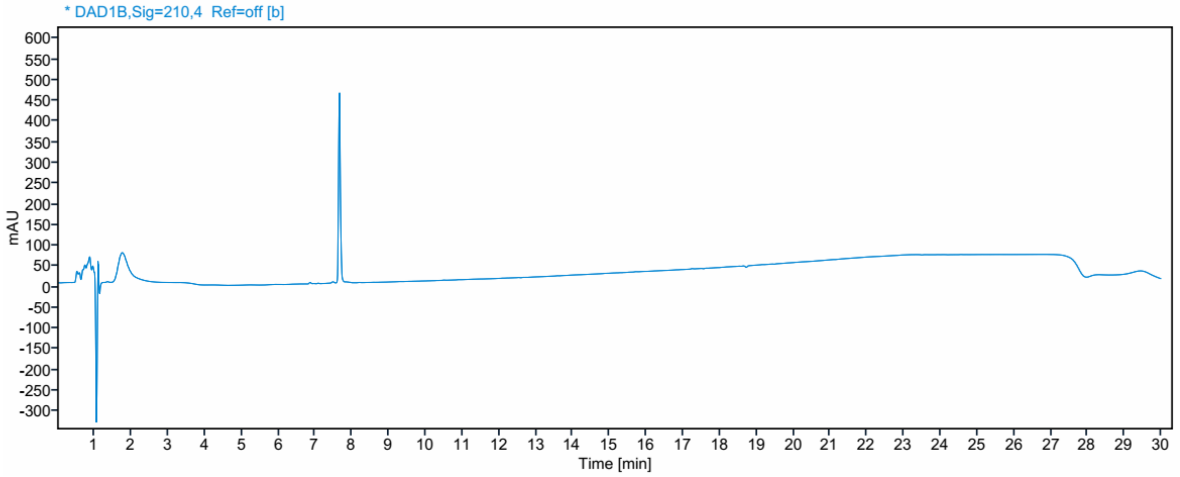

Compound **15**

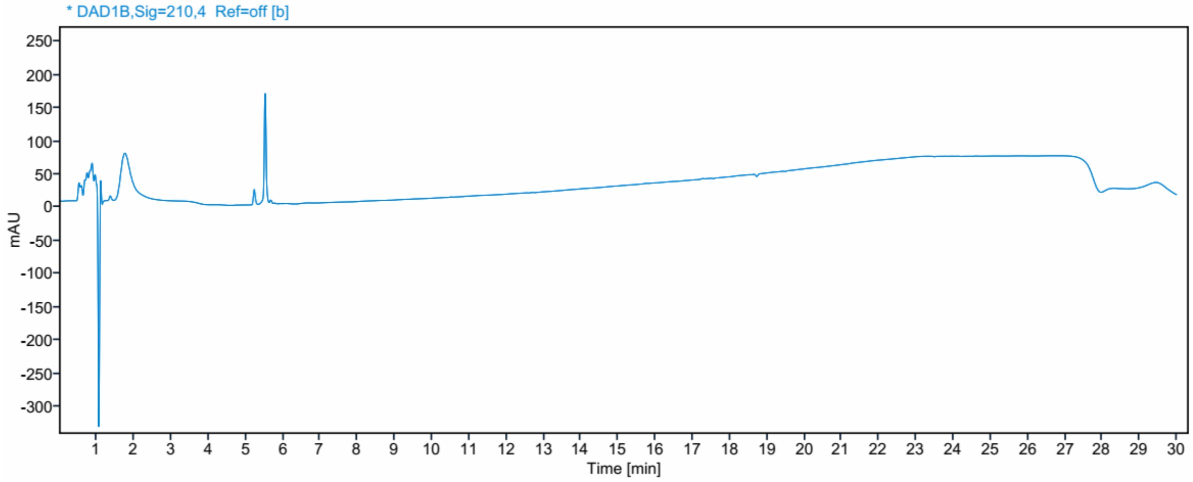

Compound **16**

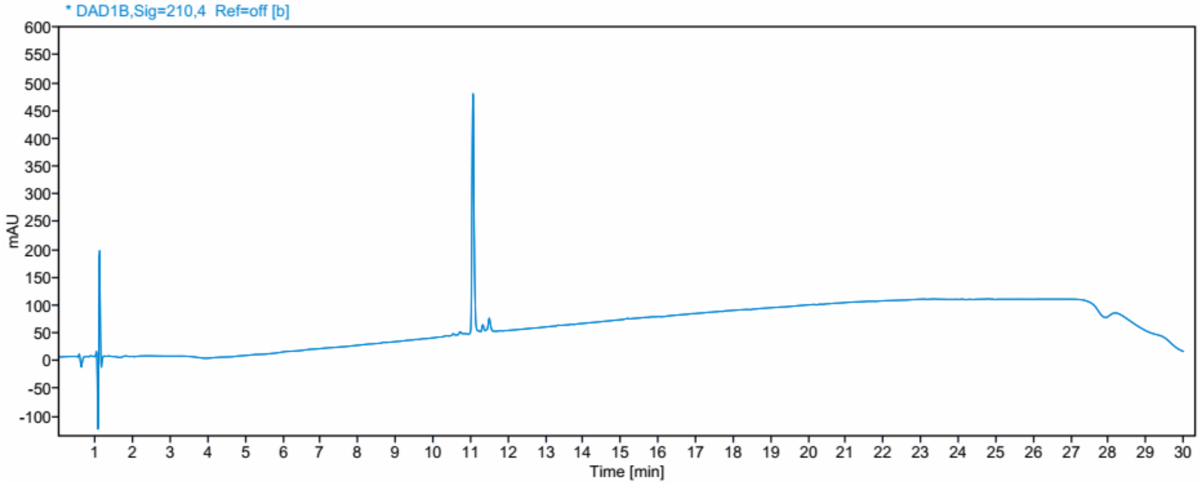

Compound **17**

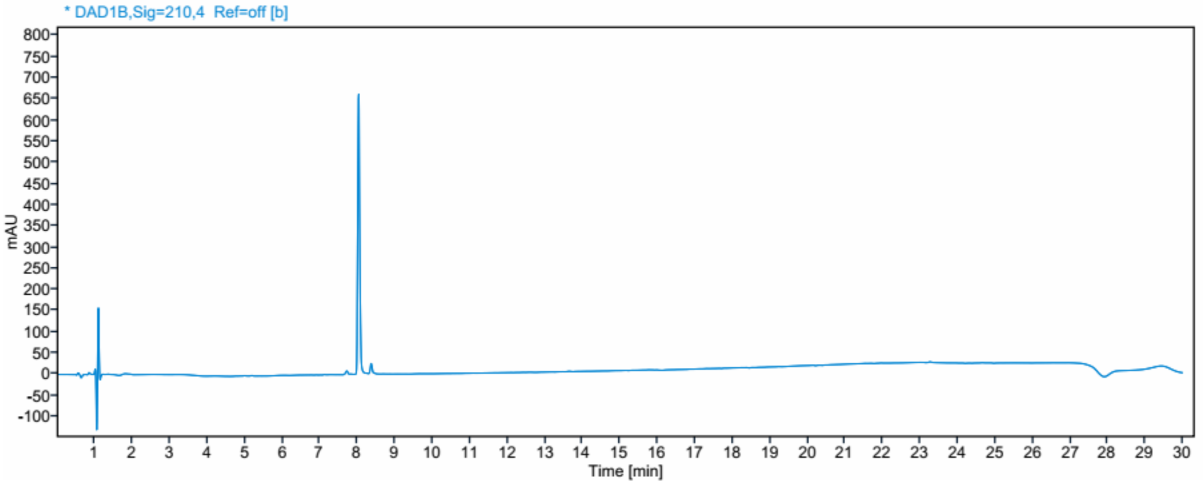

Compound **18**

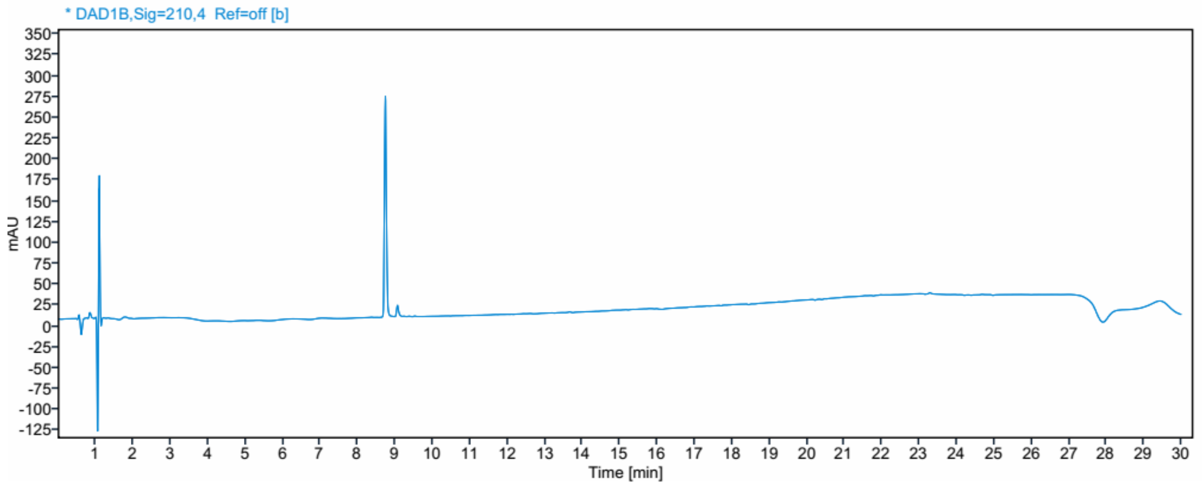

Compound **19**

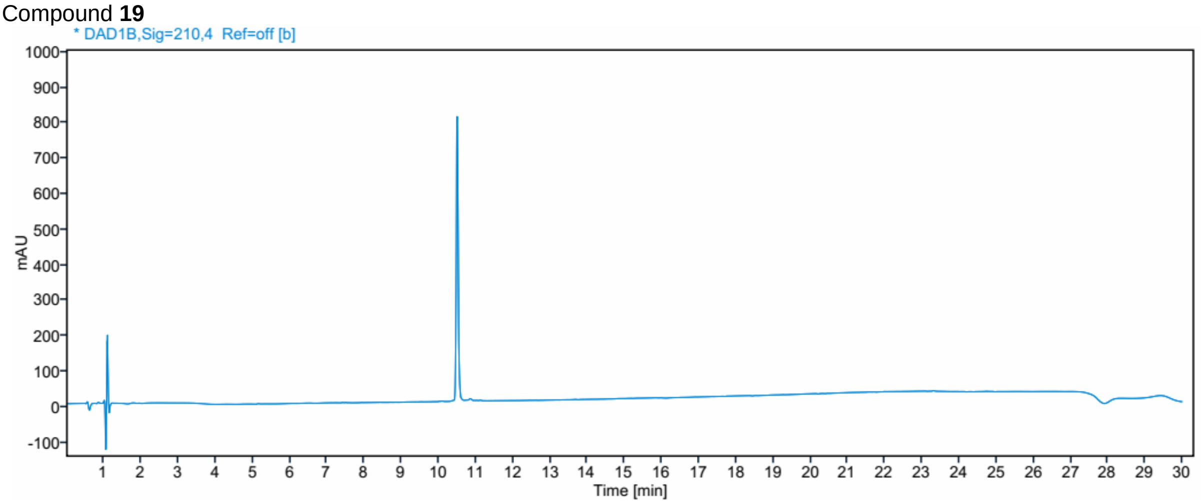

Compound **20**

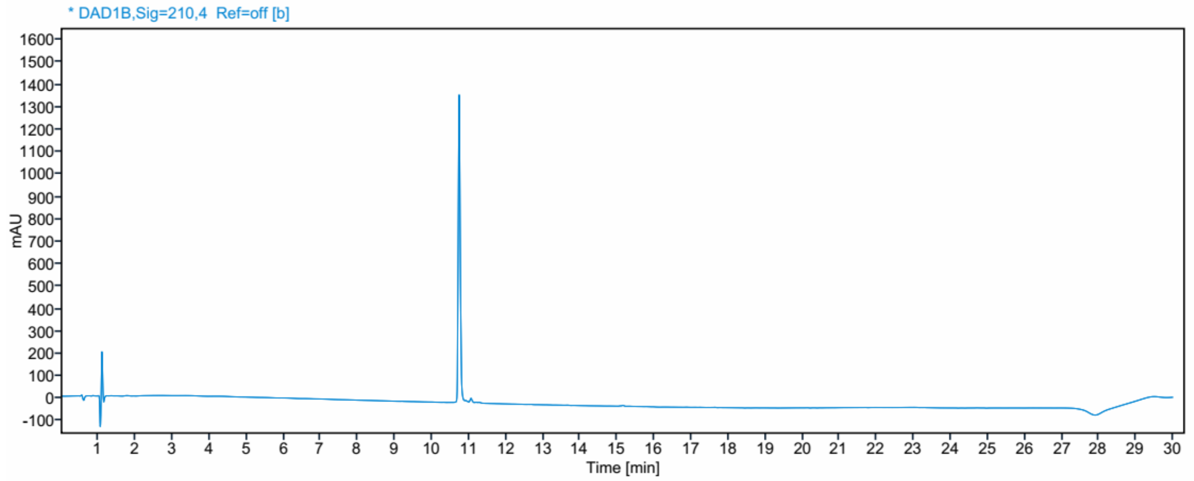

Compound **21**

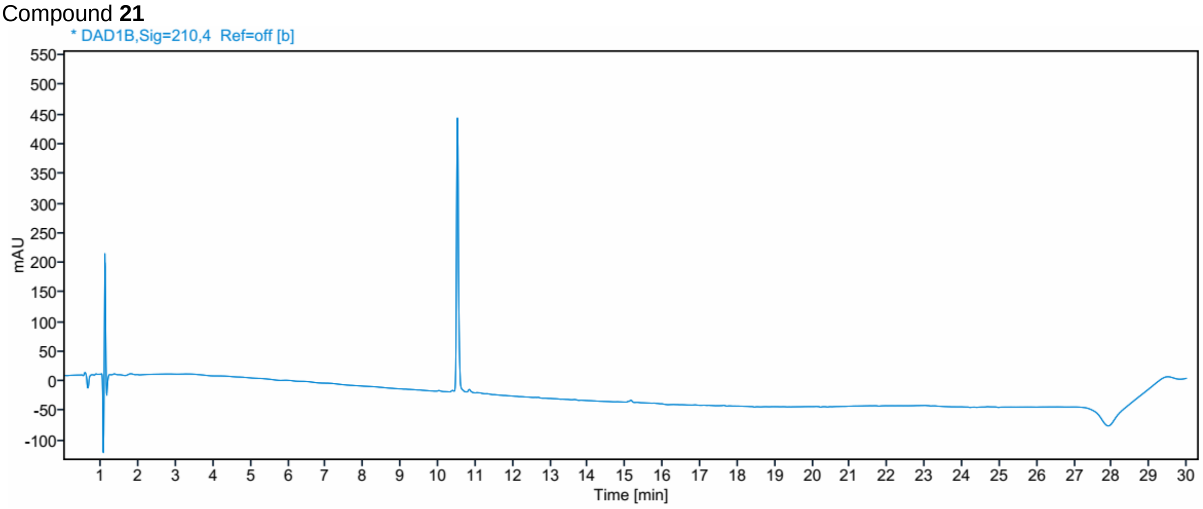

Compound **22**

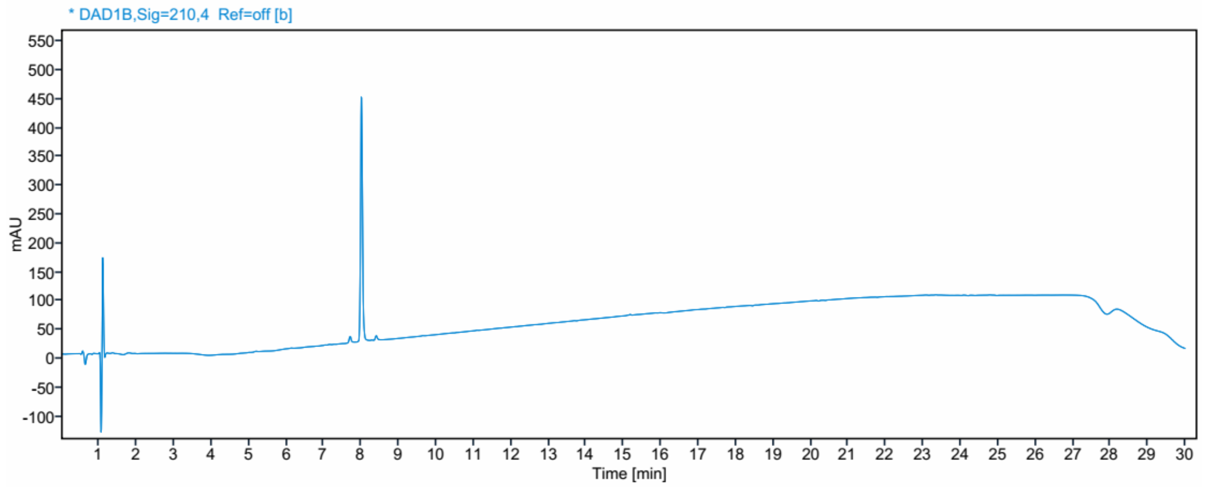

Compound **23**

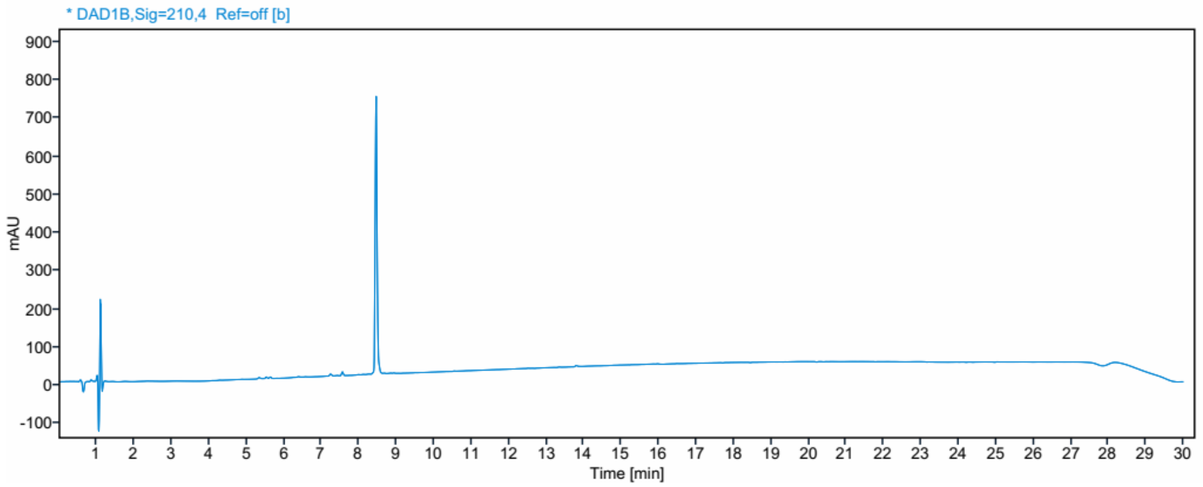

Compound **24**

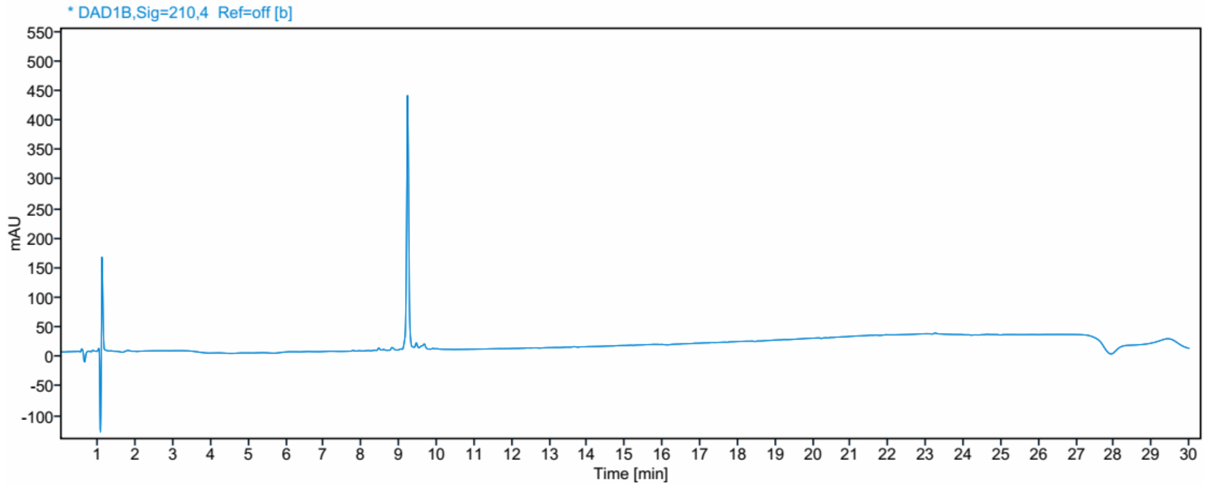

Compound **25**

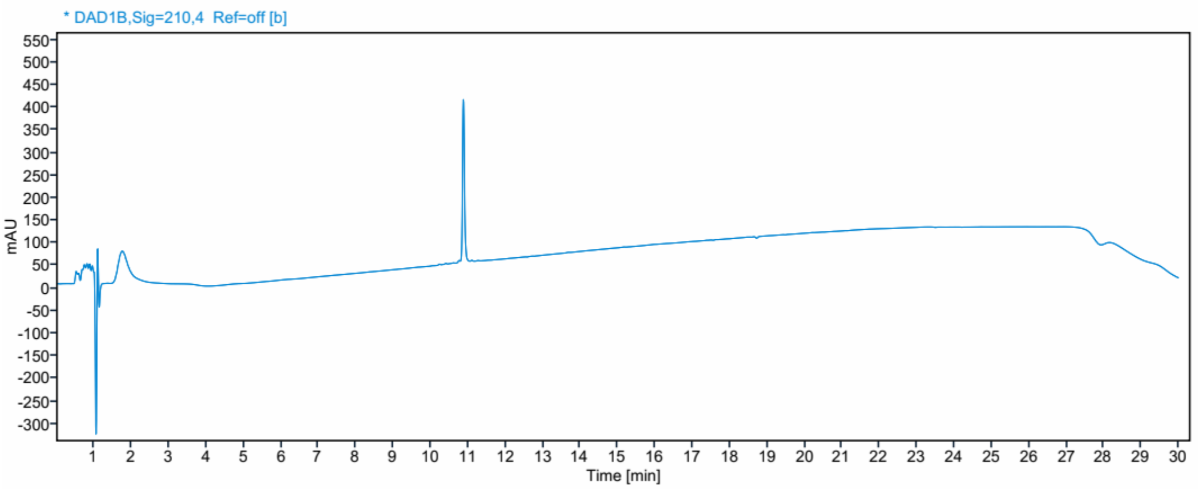

Compound **26**

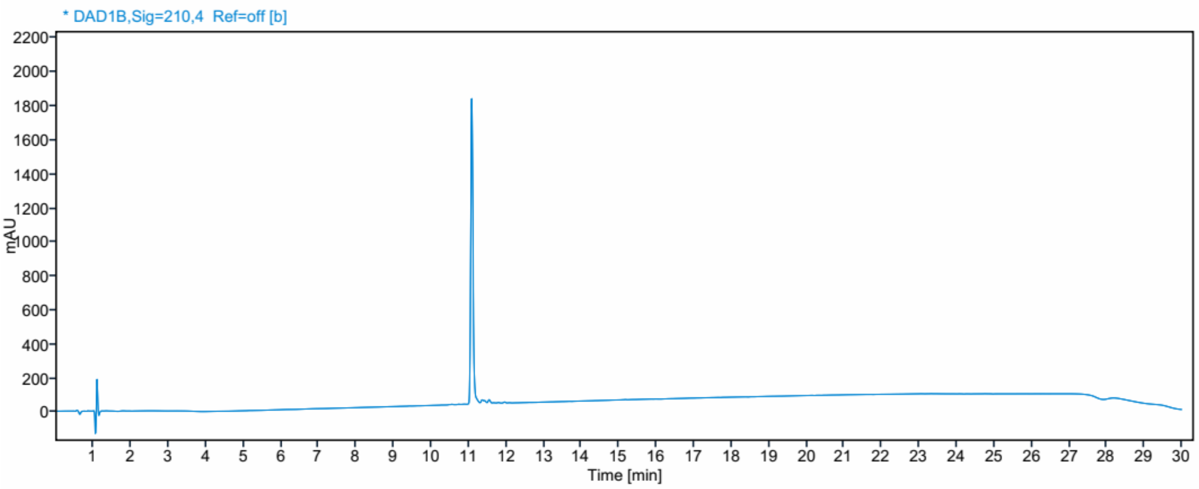

Compound **27**

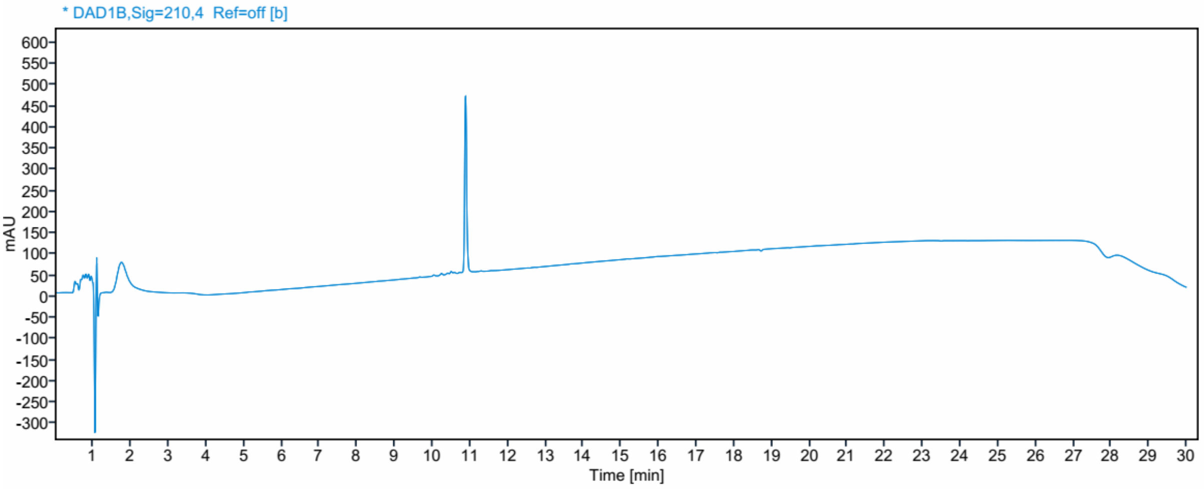

Compound **28**

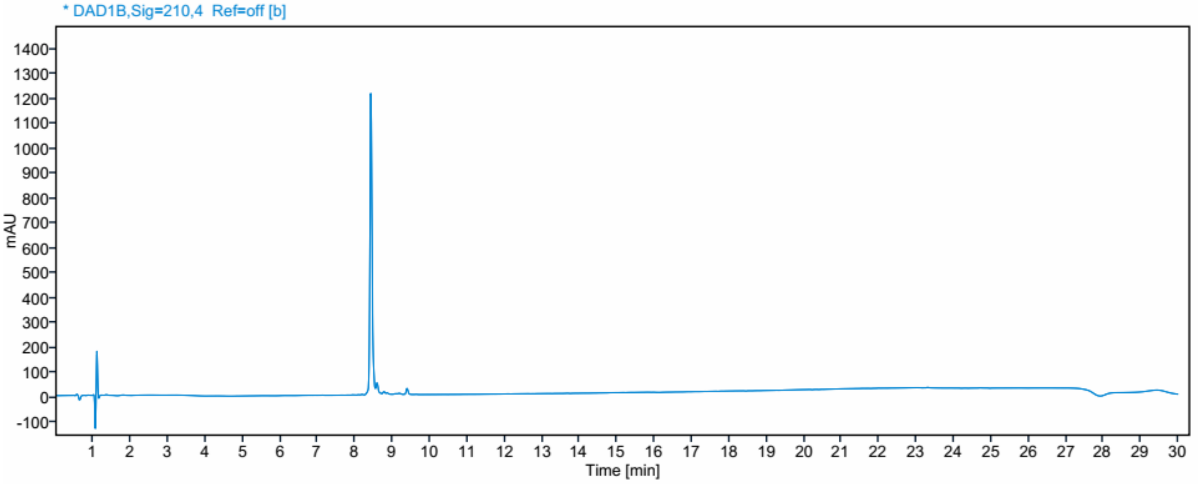

Compound **29**

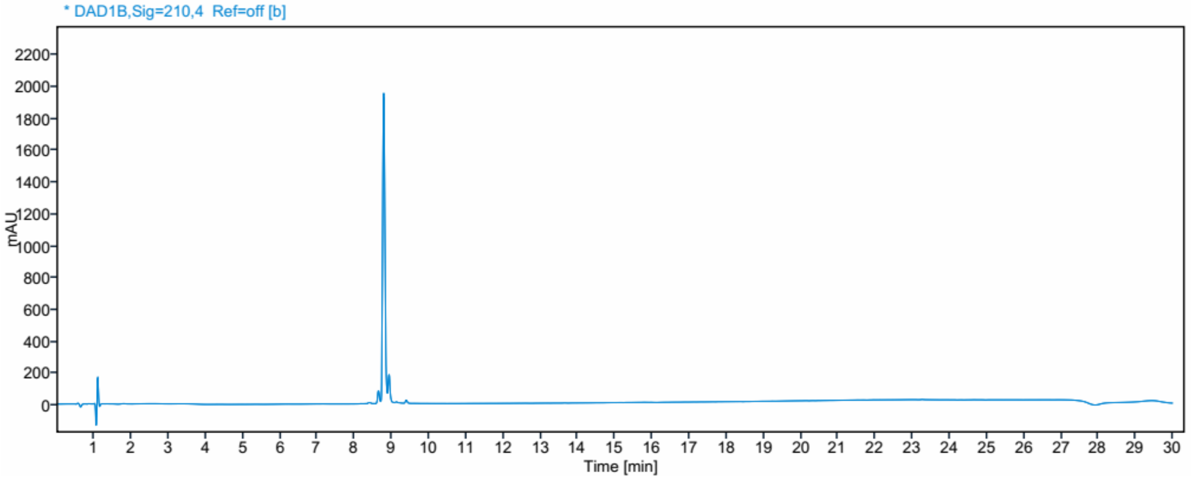

Compound **30**

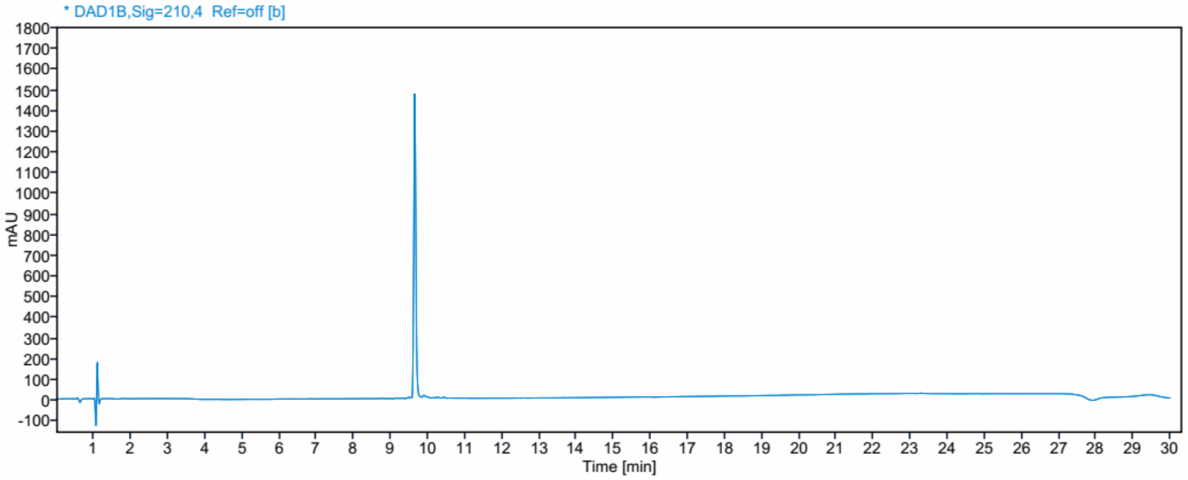

Compound **31**

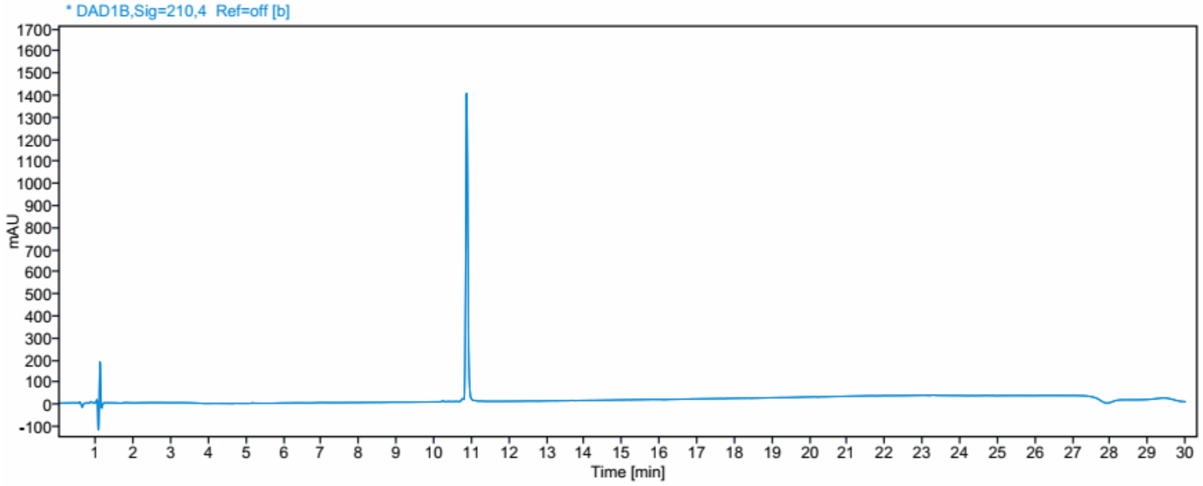

Compound **32**

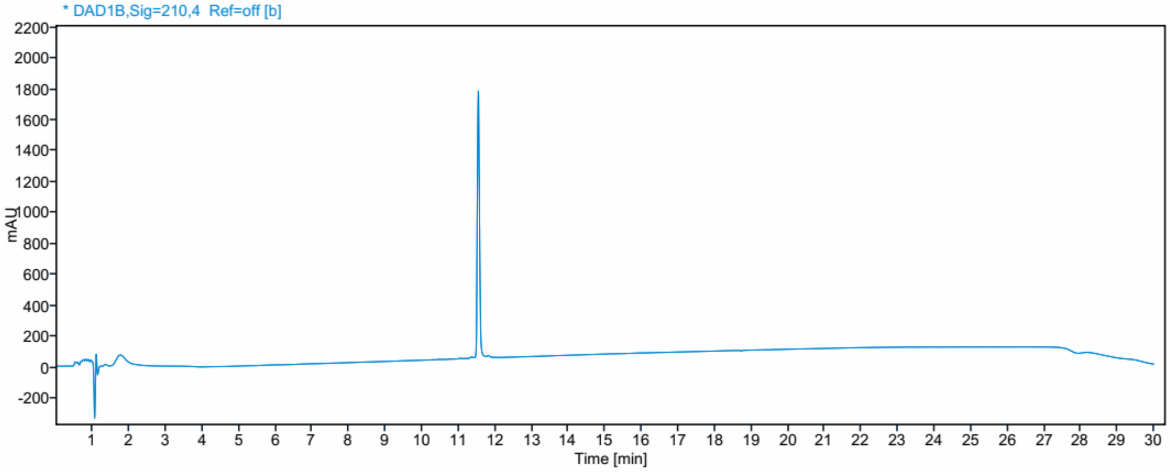

Compound **33**

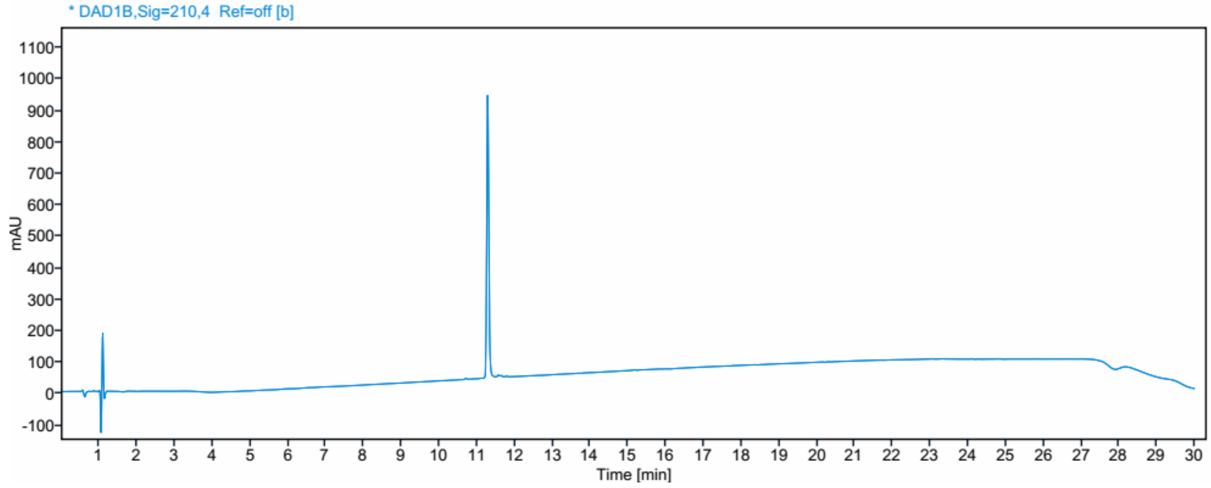

Compound **34**

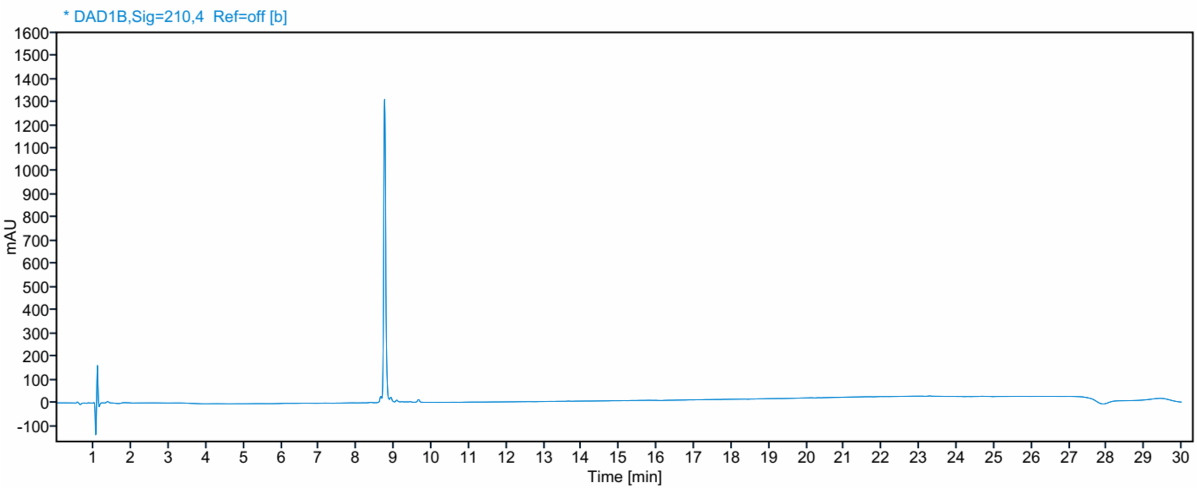

Compound **35**

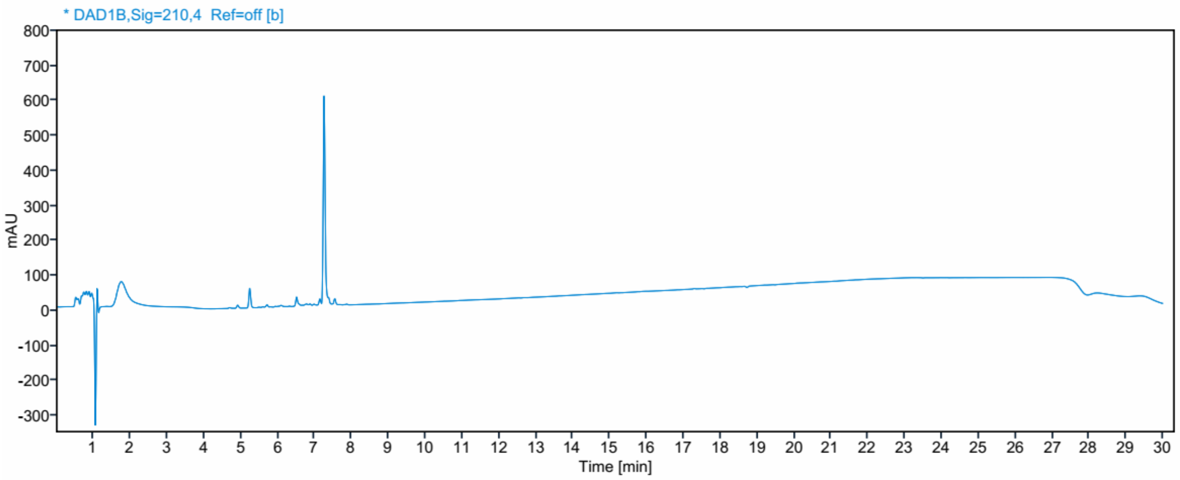

Compound **36**

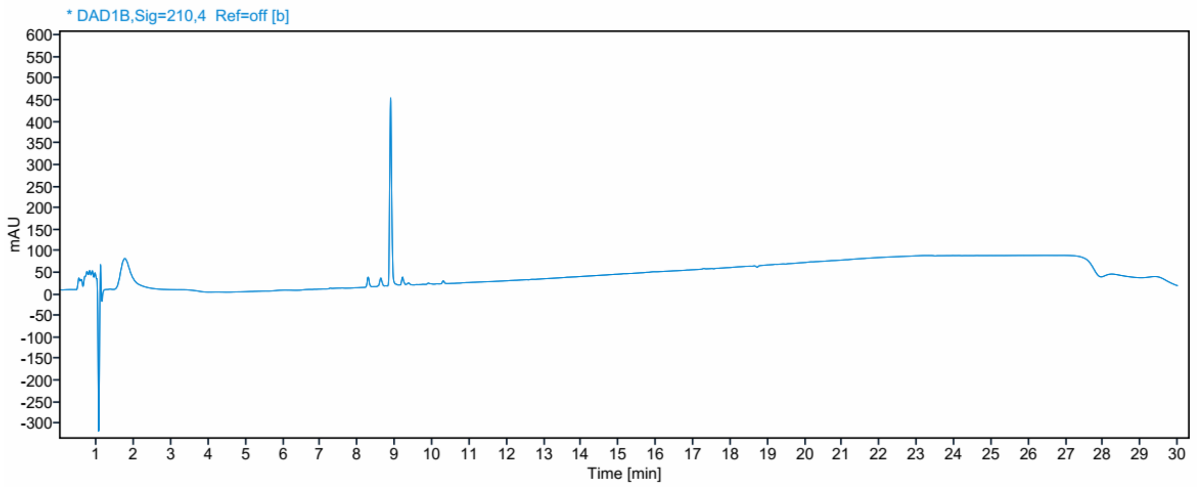

Compound **37**

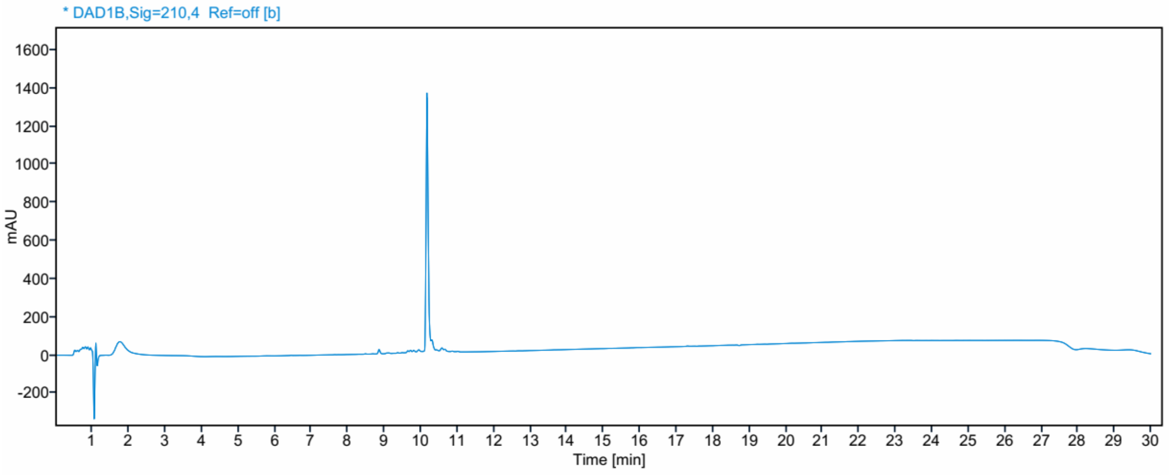

Compound **39**

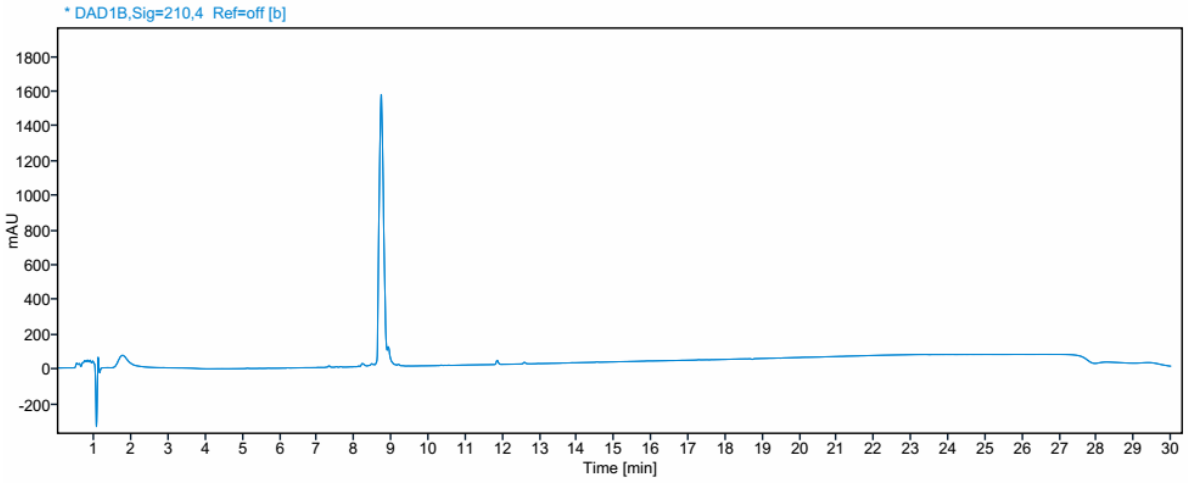

Compound **40**

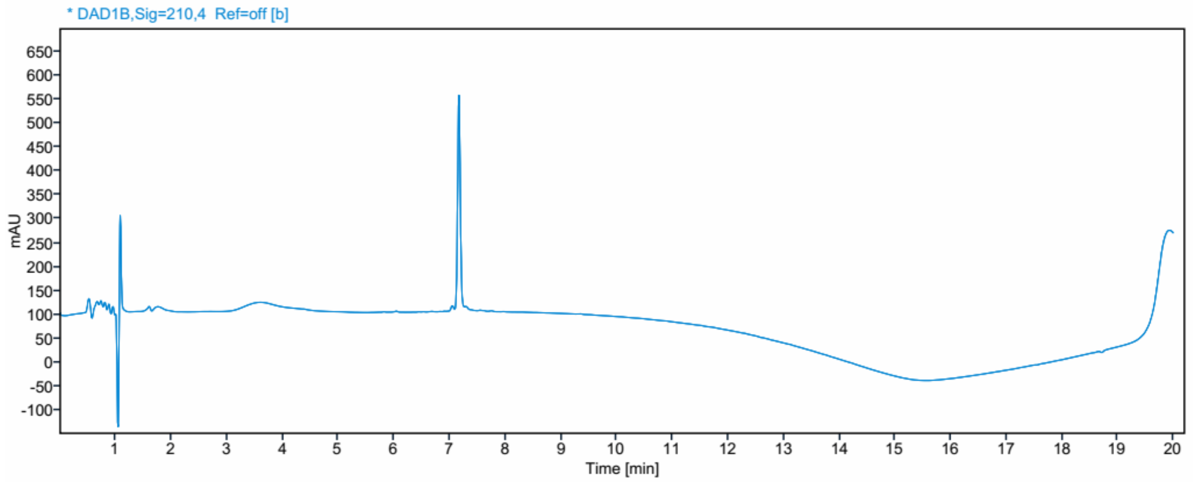

Compound **41**

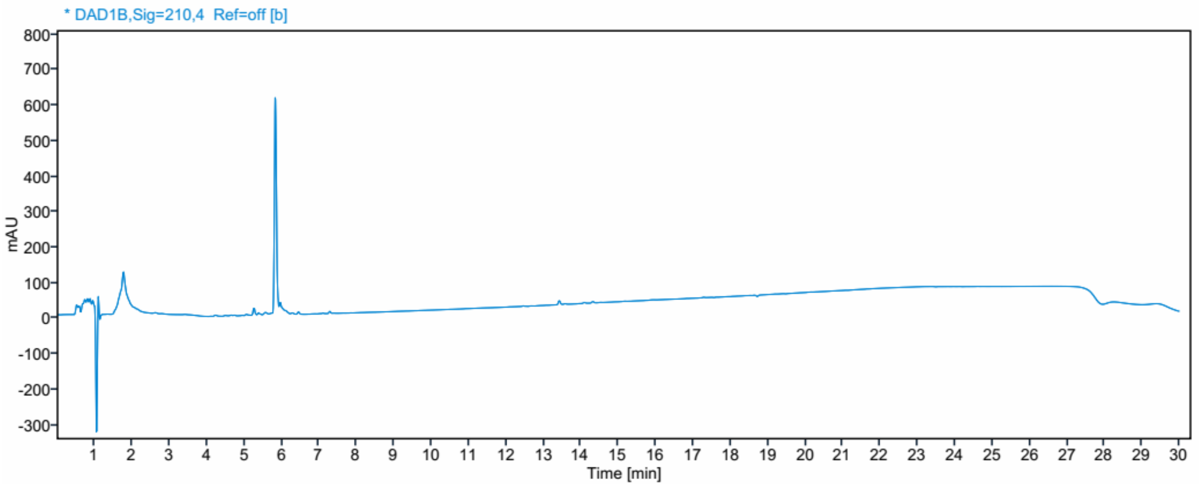

Compound **42**

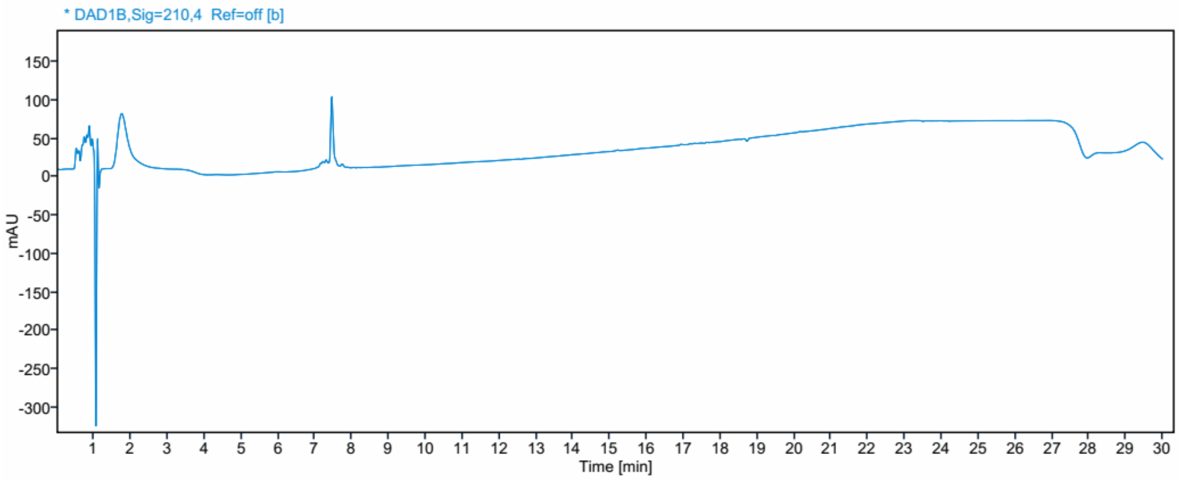

Compound **43**

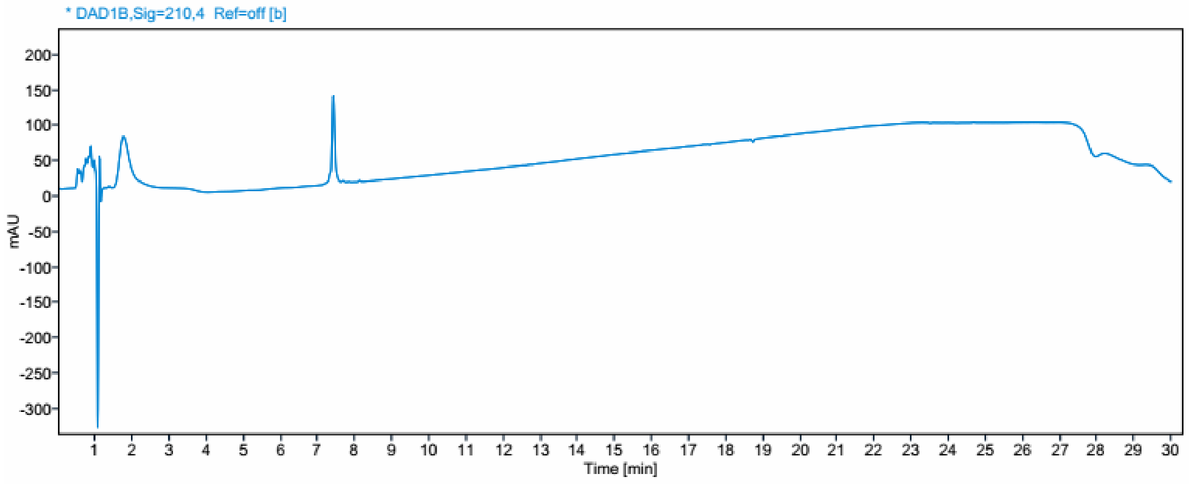

Compound **44**

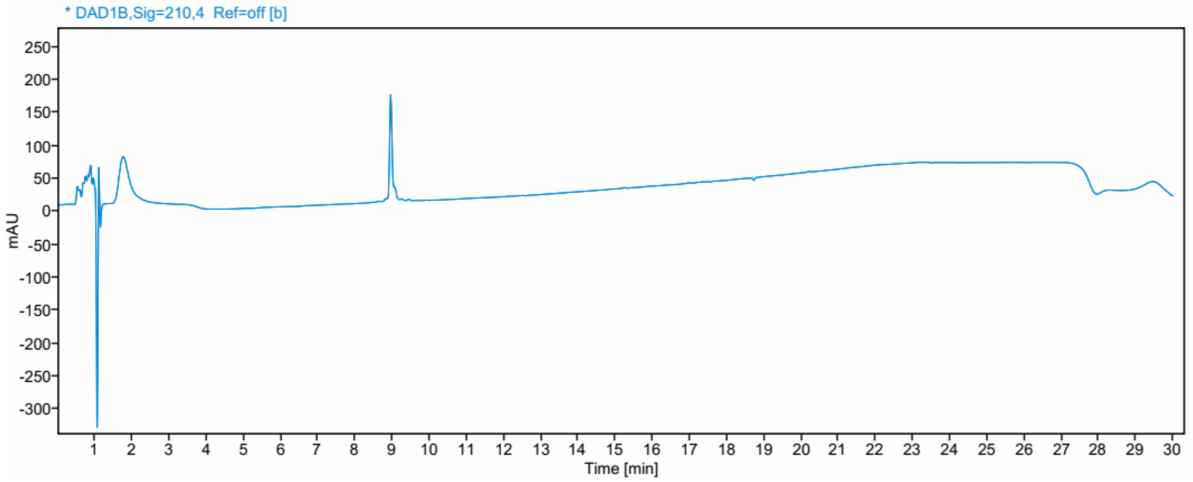

Compound **45**

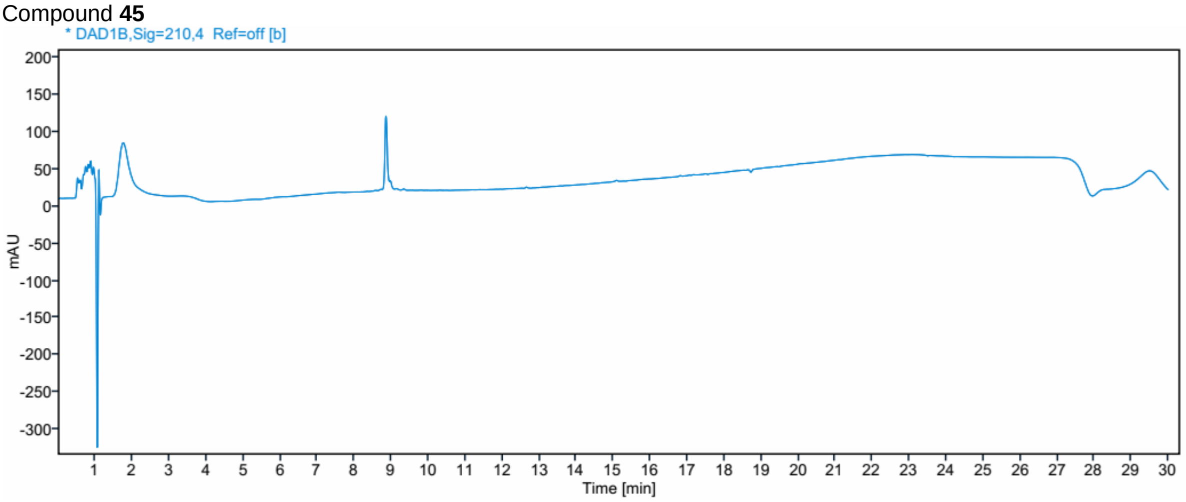

Compound **46**

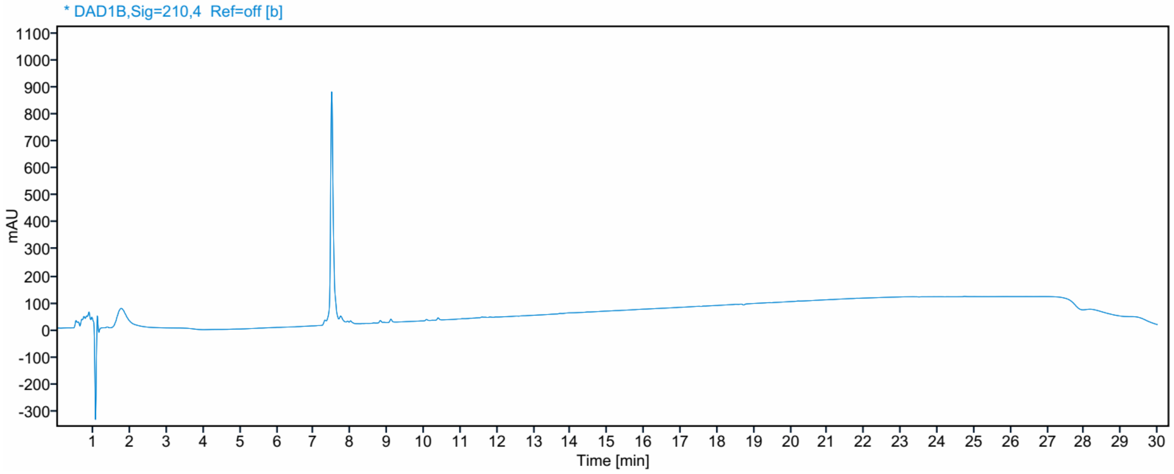

NMR data:

^{1}H NMR of compound **1** in DMSO-d$_6$, 700 MHz

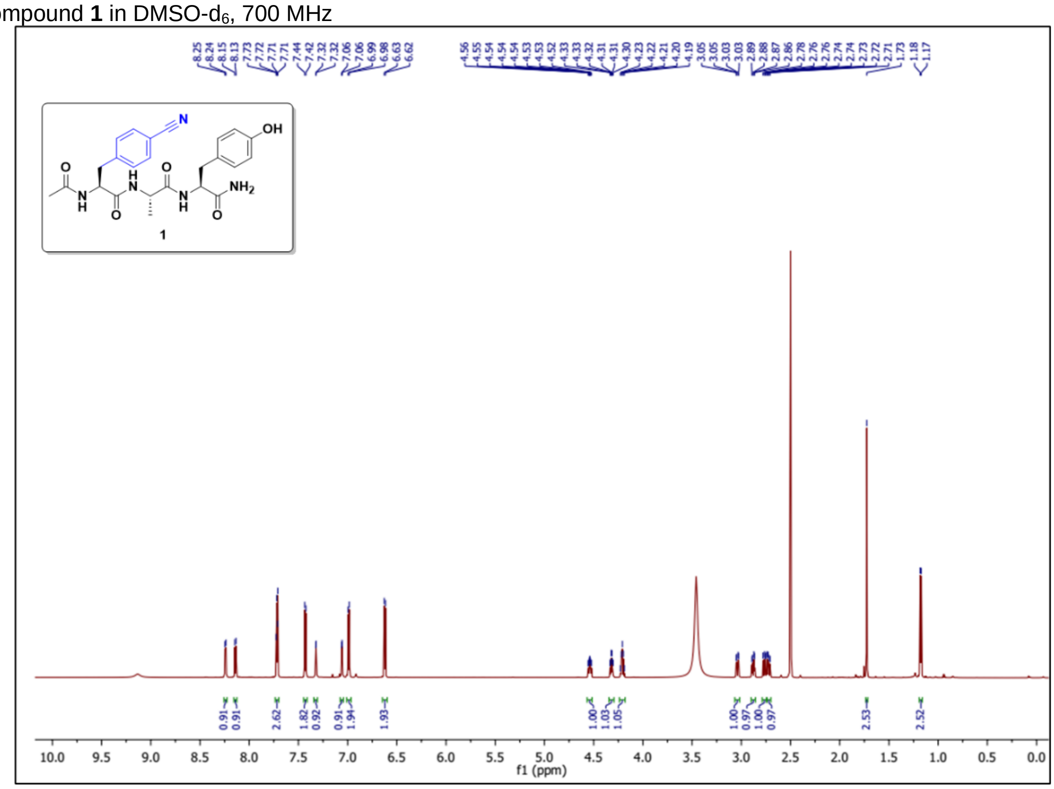

^{13}C NMR of compound **1** in DMSO-d$_6$, 176 MHz

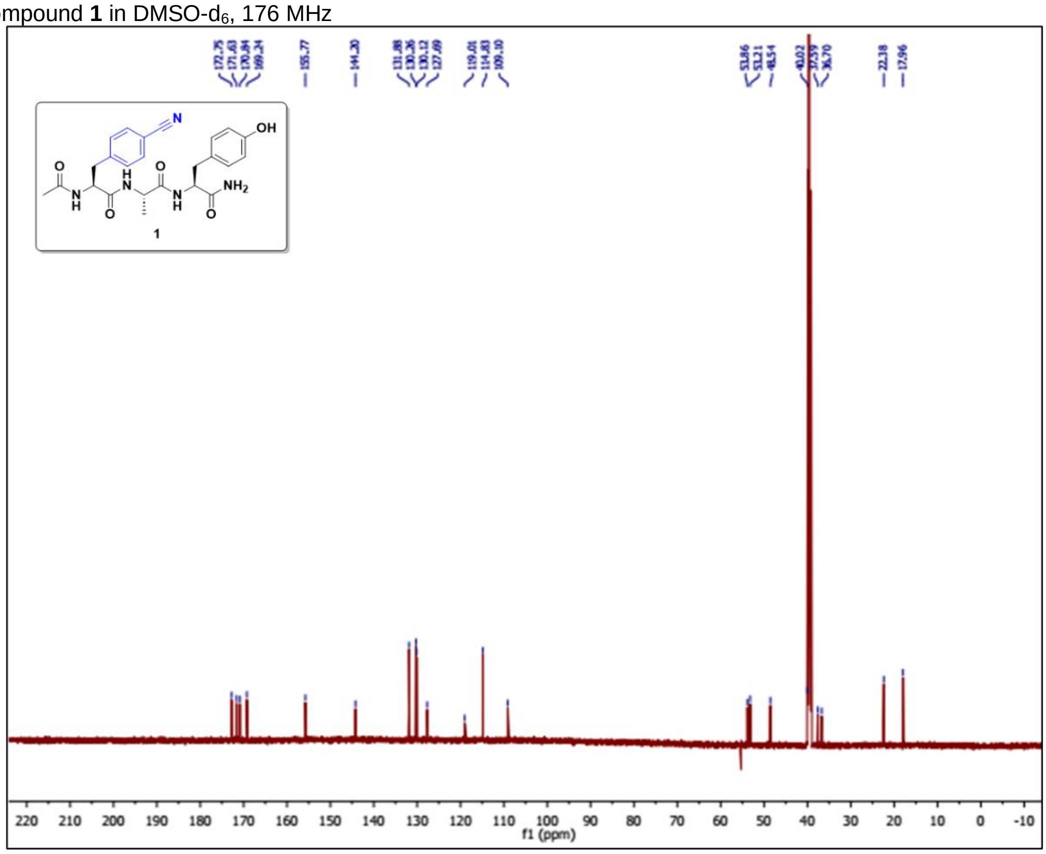

^{1}H NMR of compound **2** in DMSO-d_6, 700 MHz

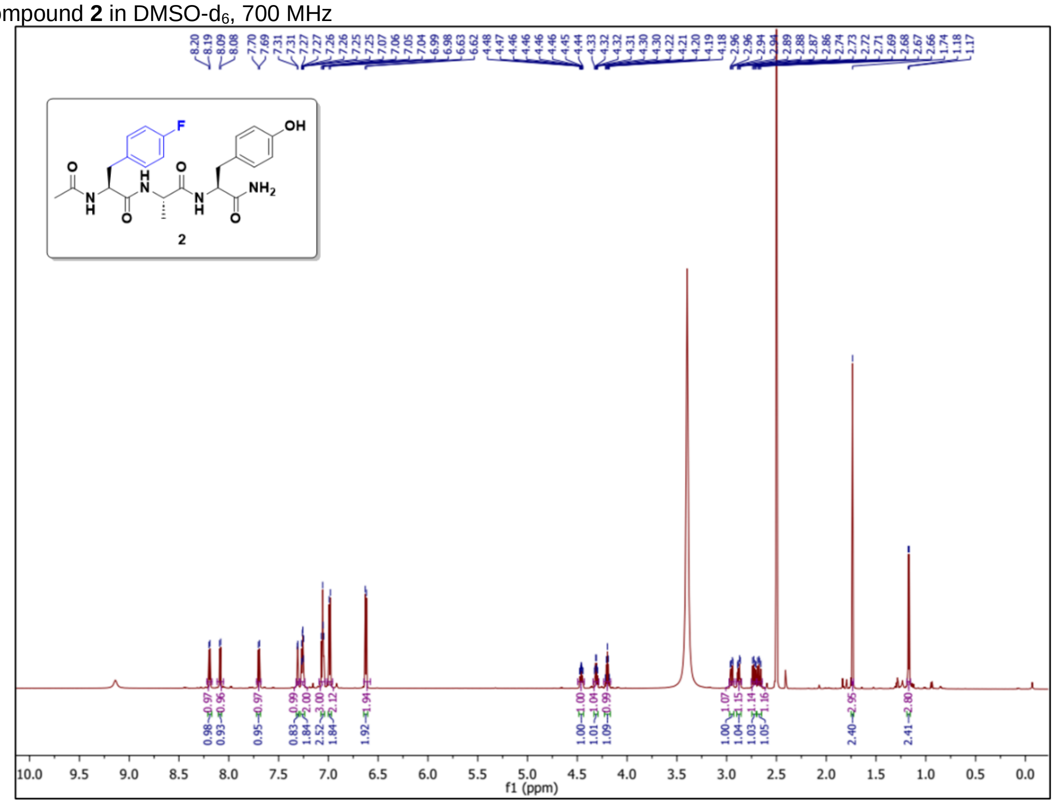

^{13}C NMR of compound **2** in DMSO-d_6, 176 MHz

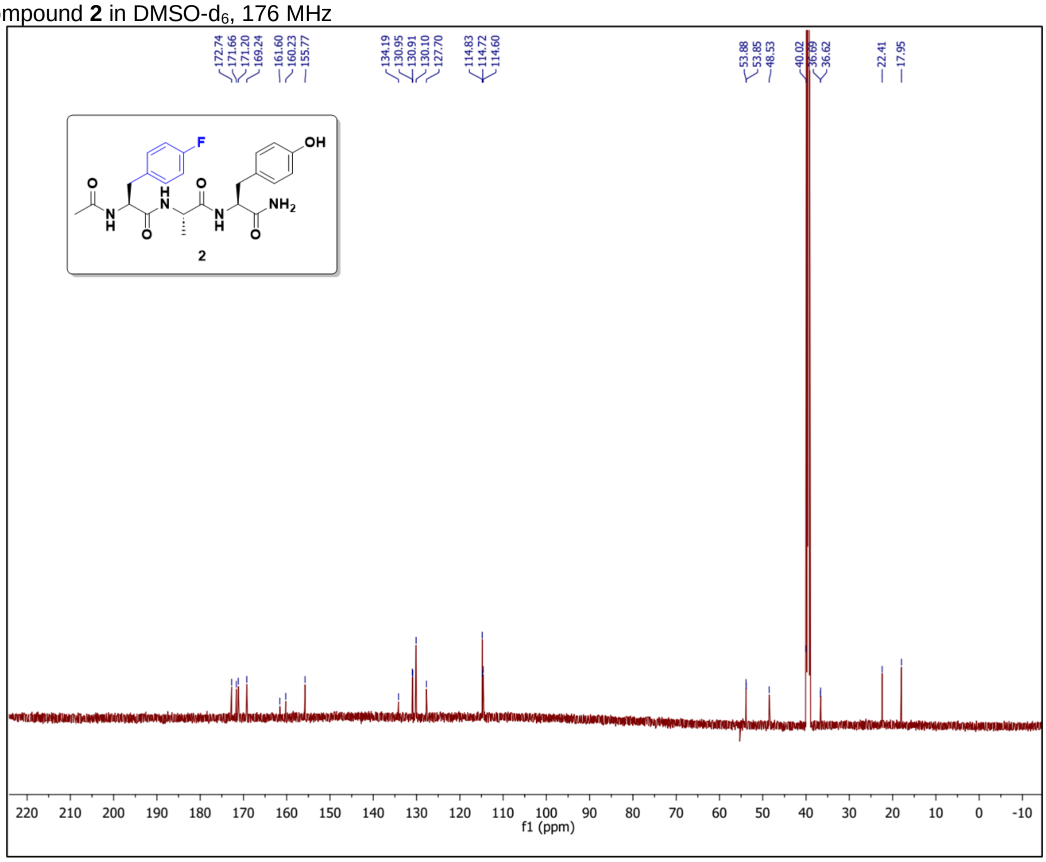

^{19}F NMR of compound **2** in DMSO-d$_6$, 470 MHz (* corresponds to residual trifluoroacetic acid)

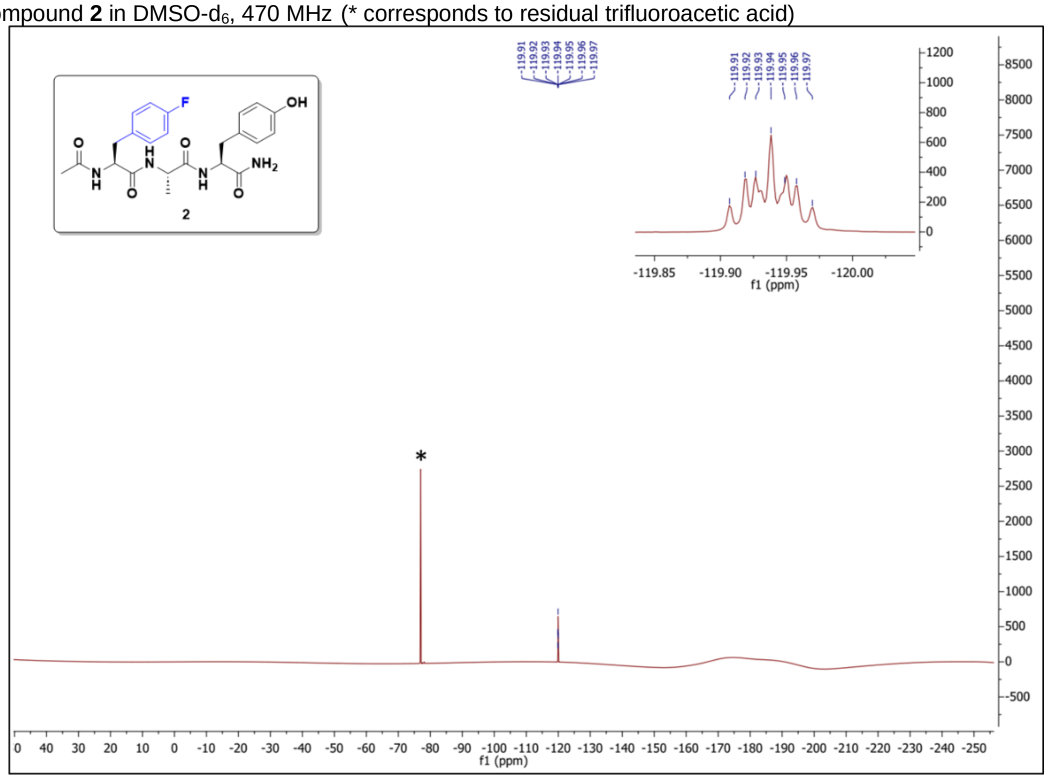

^{1}H NMR of compound **3** in DMSO-d$_6$, 700 MHz

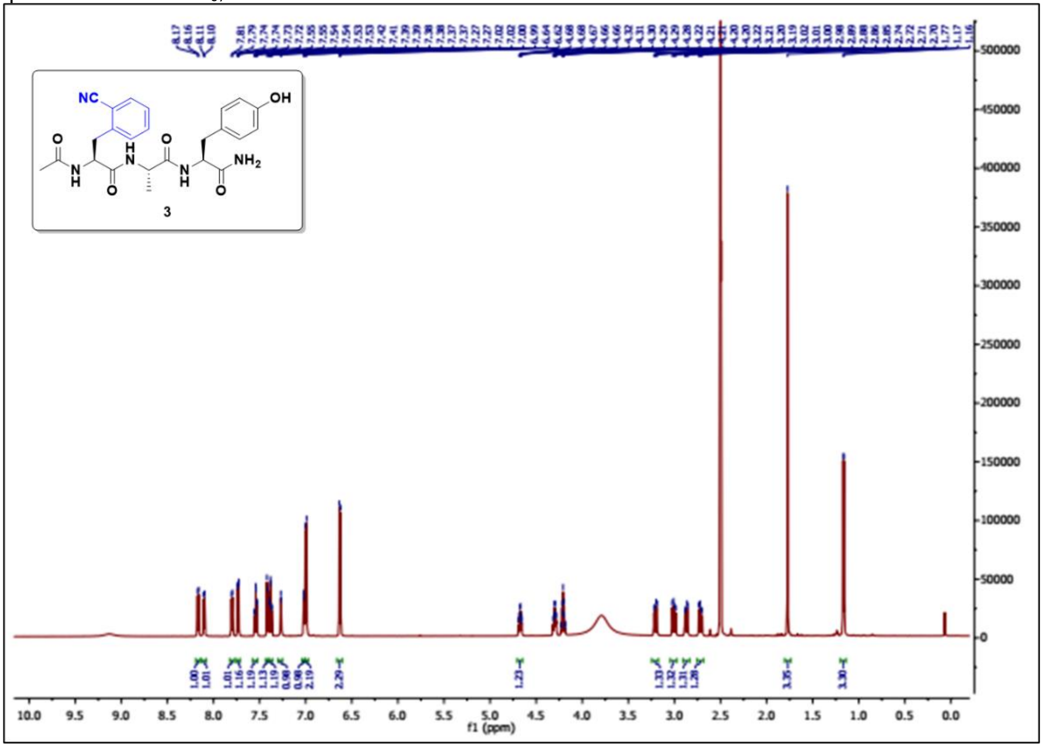

^{13}C NMR of compound **3** in DMSO-d$_6$, 176 MHz

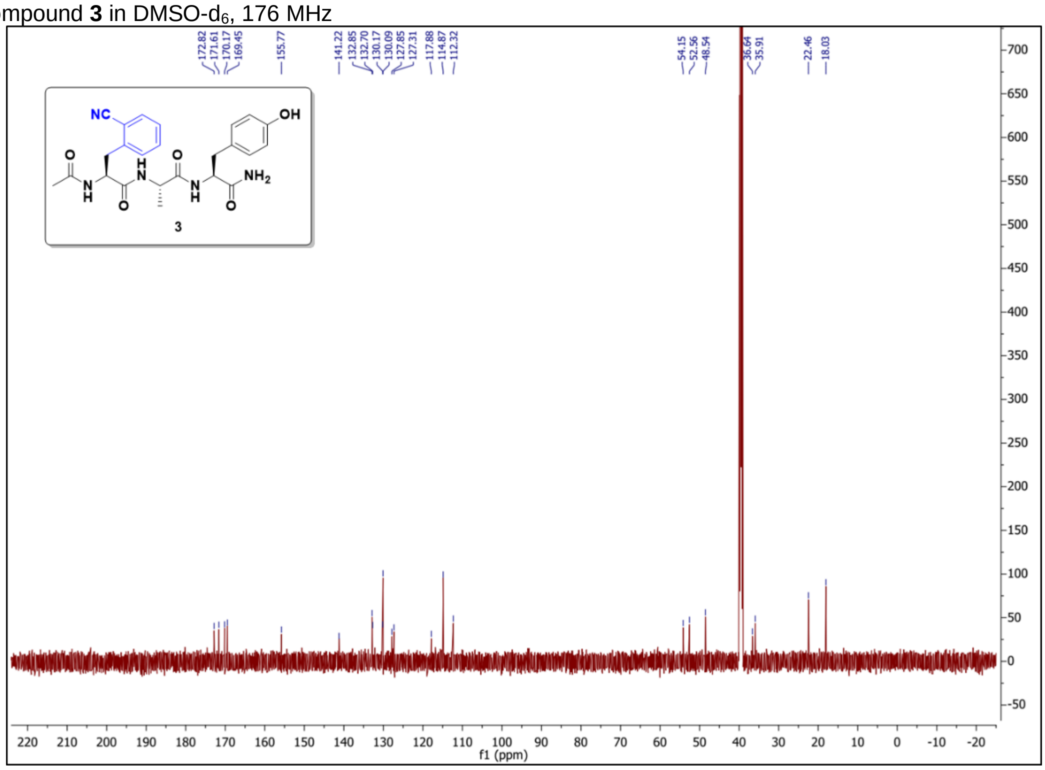

^{1}H NMR of compound **4** in DMSO-d$_6$, 700 MHz

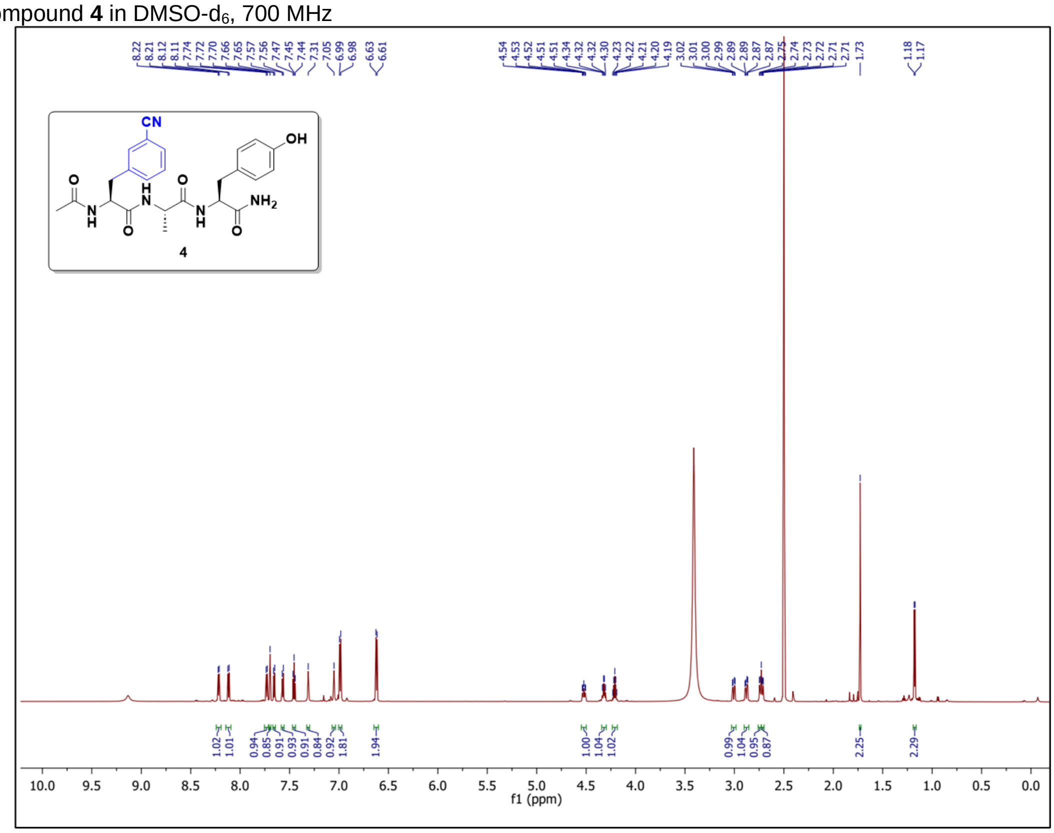

^{13}C NMR of compound **4** in DMSO-d$_6$, 176 MHz

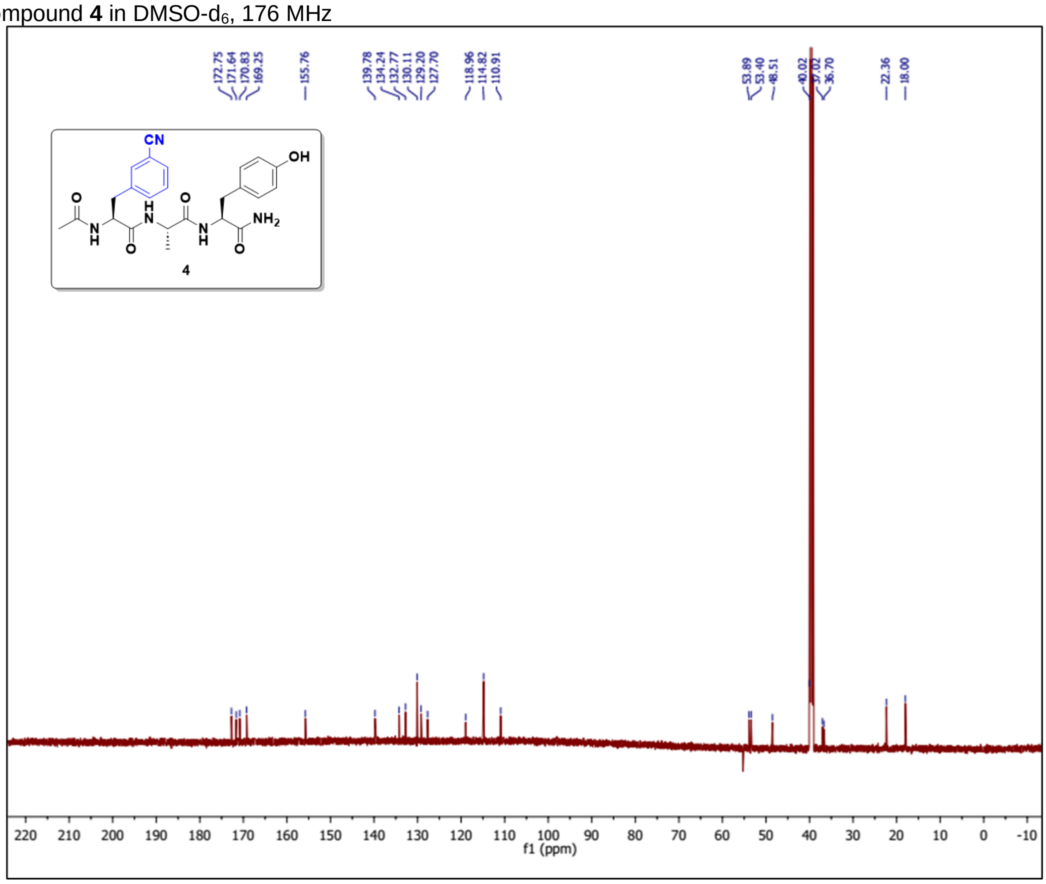

^{1}H NMR of compound **5** in DMSO-d$_6$, 700 MHz

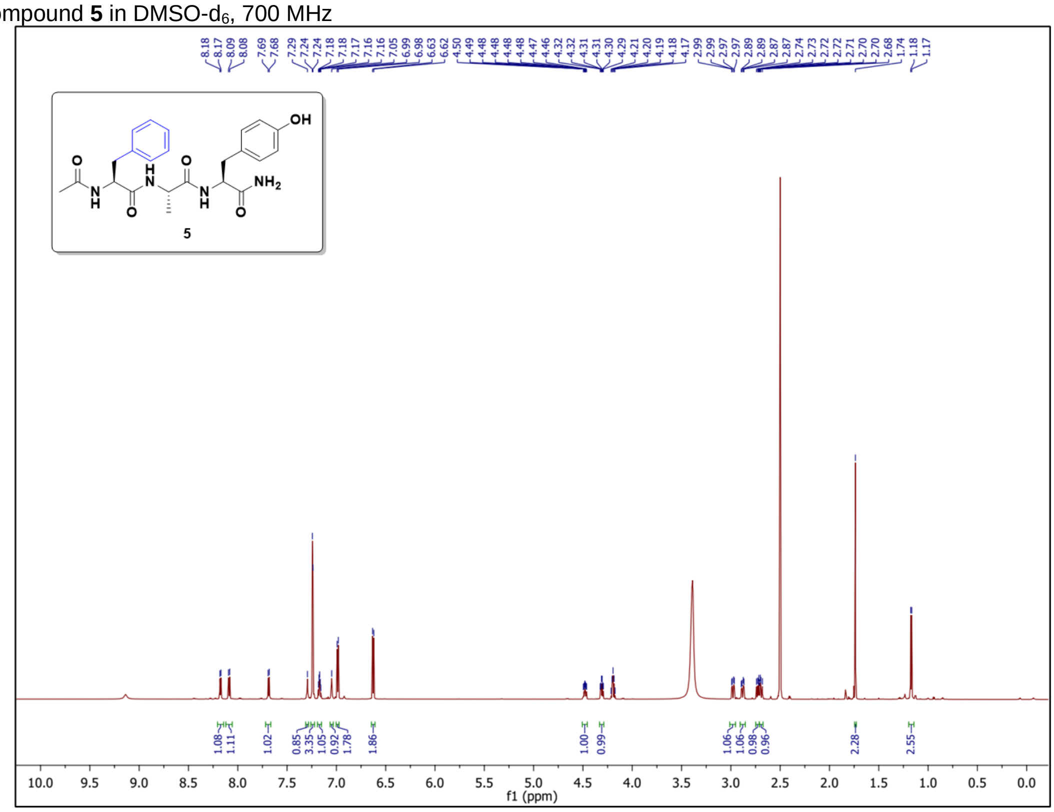

^{13}C NMR of compound **5** in DMSO-d$_6$, 176 MHz

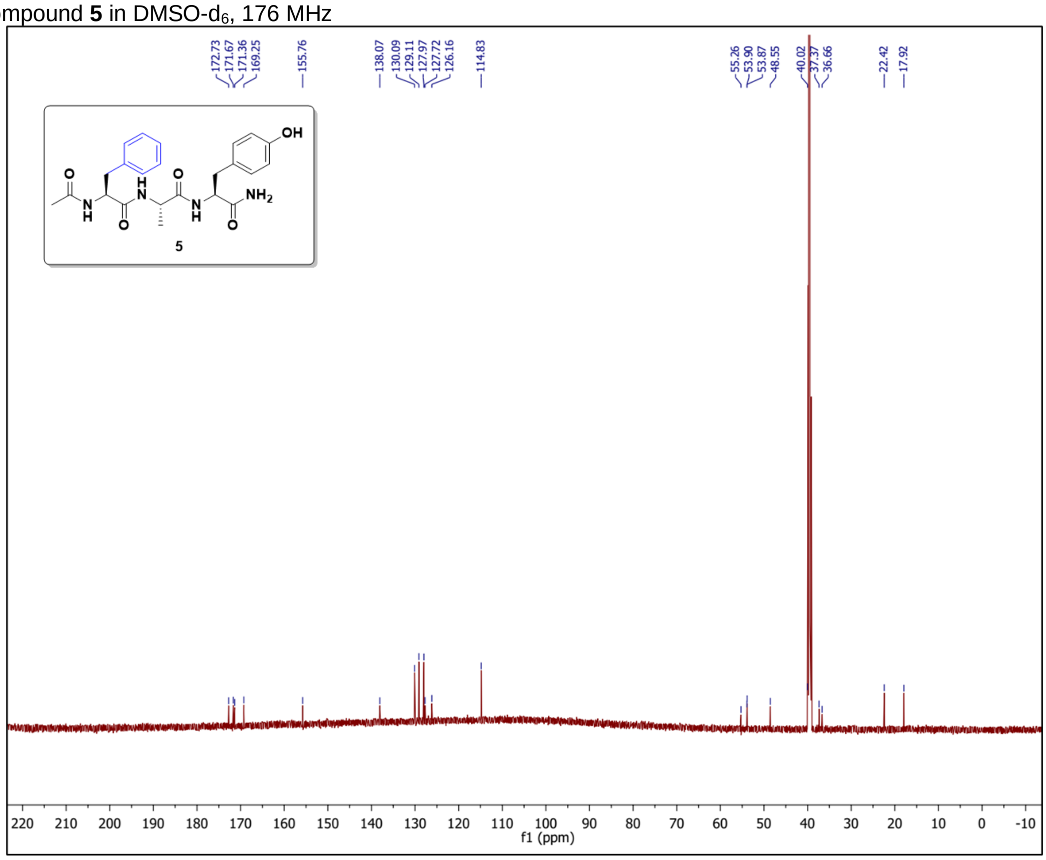

^{1}H NMR of compound **6** in DMSO-d$_6$, 700 MHz

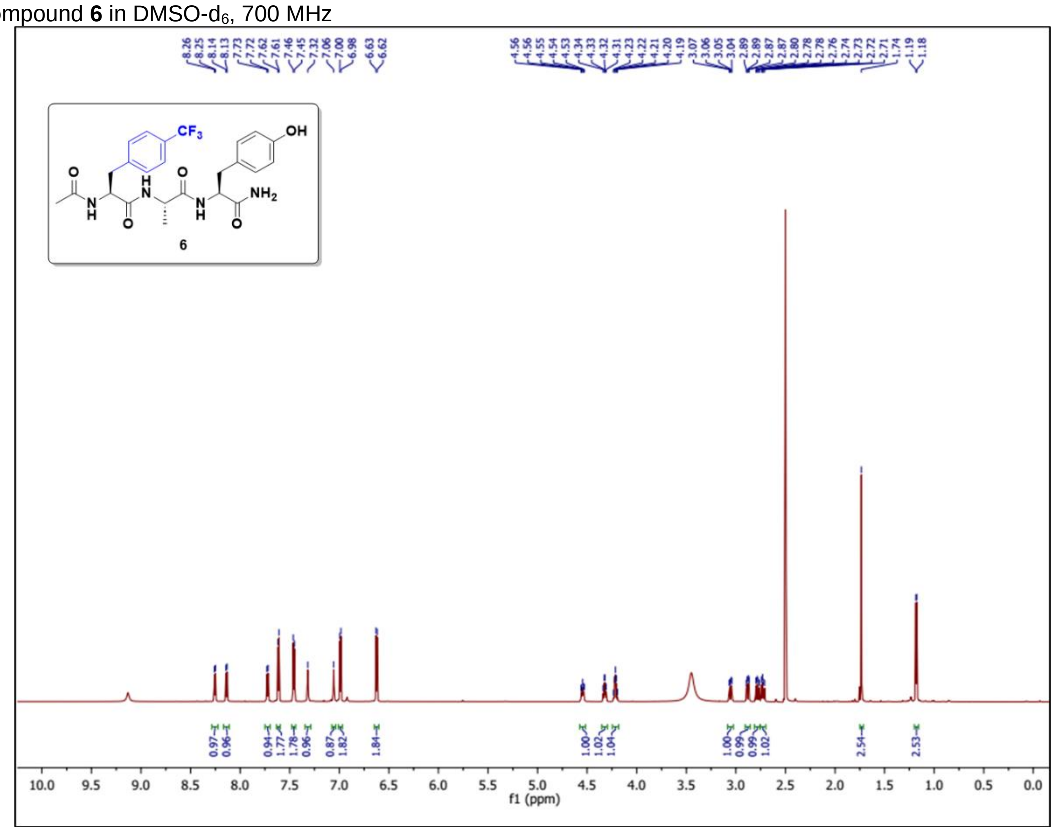

^{13}C NMR of compound **6** in DMSO-d_6, 176 MHz

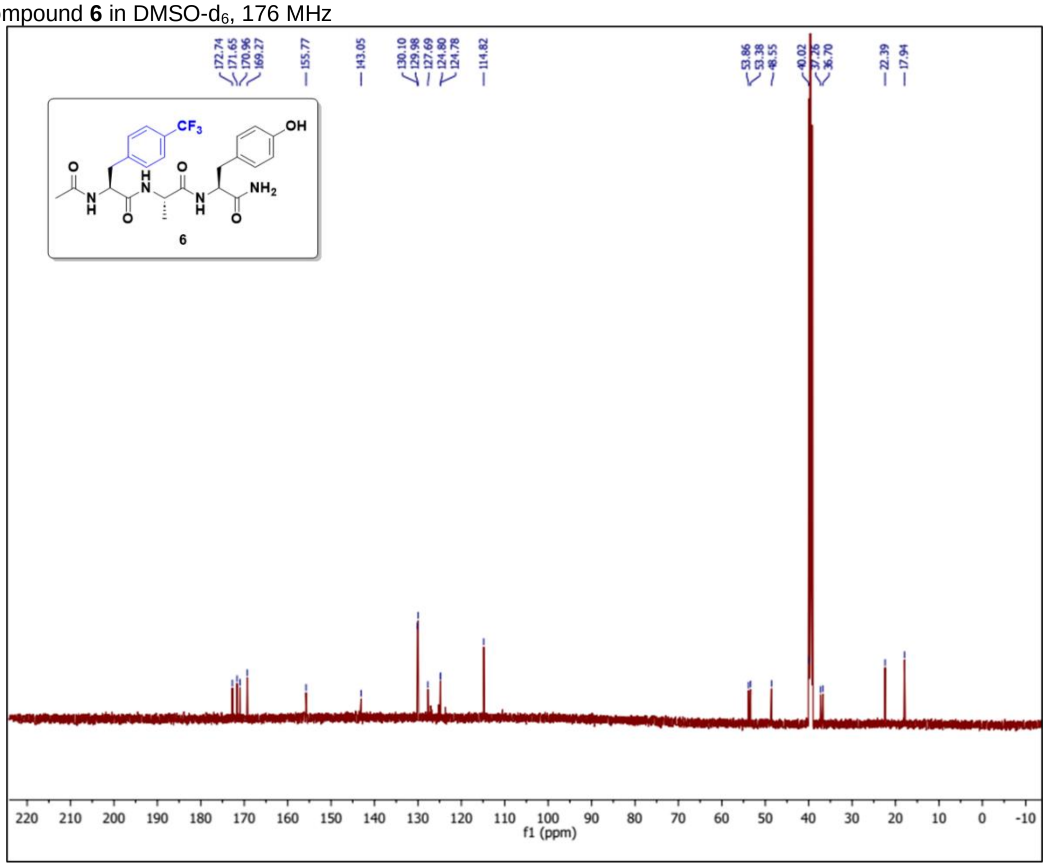

^{19}F NMR of compound **6** in DMSO-d_6, 470 MHz (* corresponds to residual trifluoroacetic acid)

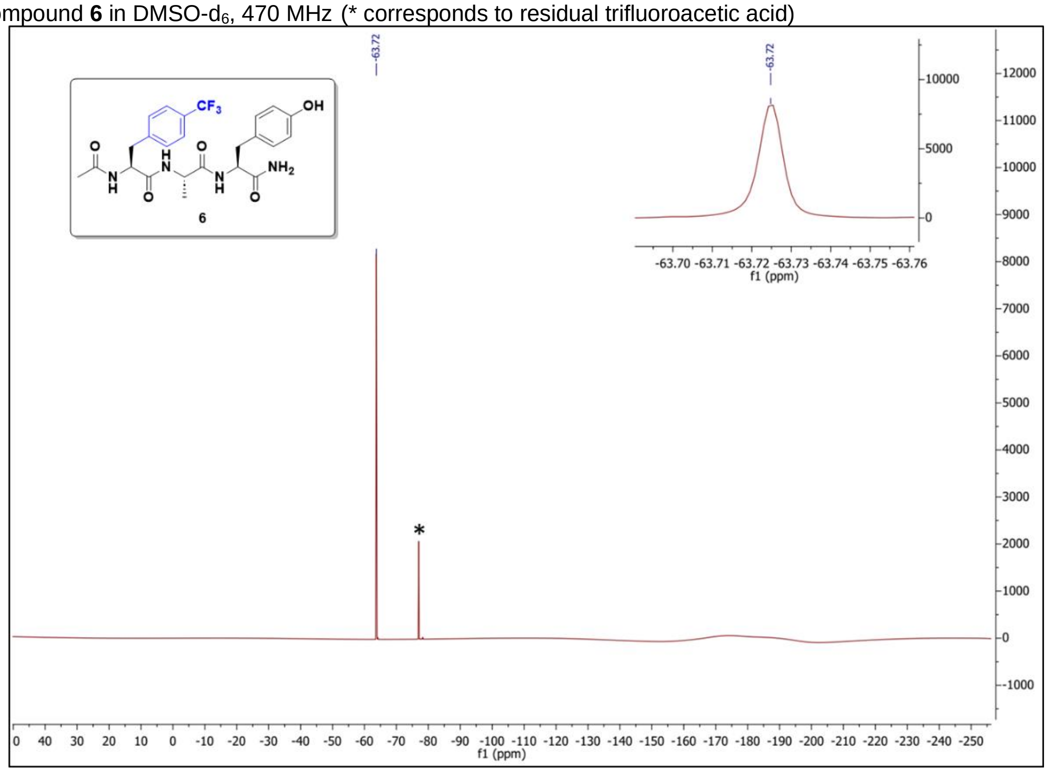

^{1}H NMR of compound **7** in DMSO-d_6, 700 MHz

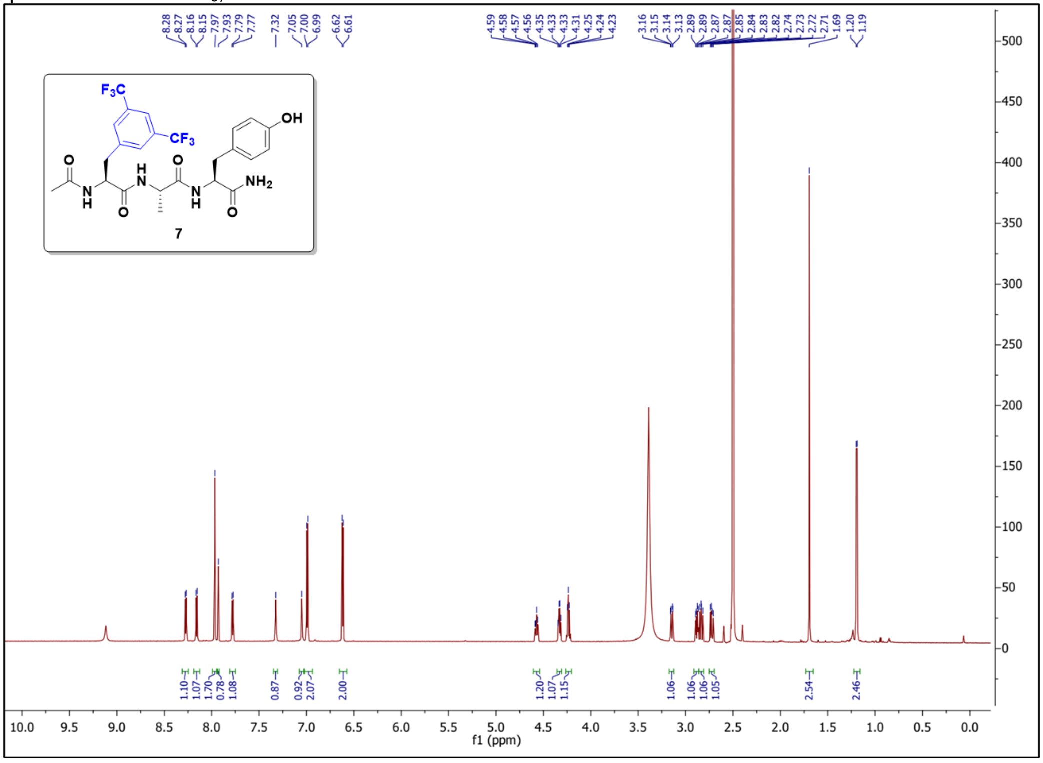

^{19}F NMR of compound **7** in DMSO-d_6, 470 MHz (* corresponds to residual trifluoroacetic acid)

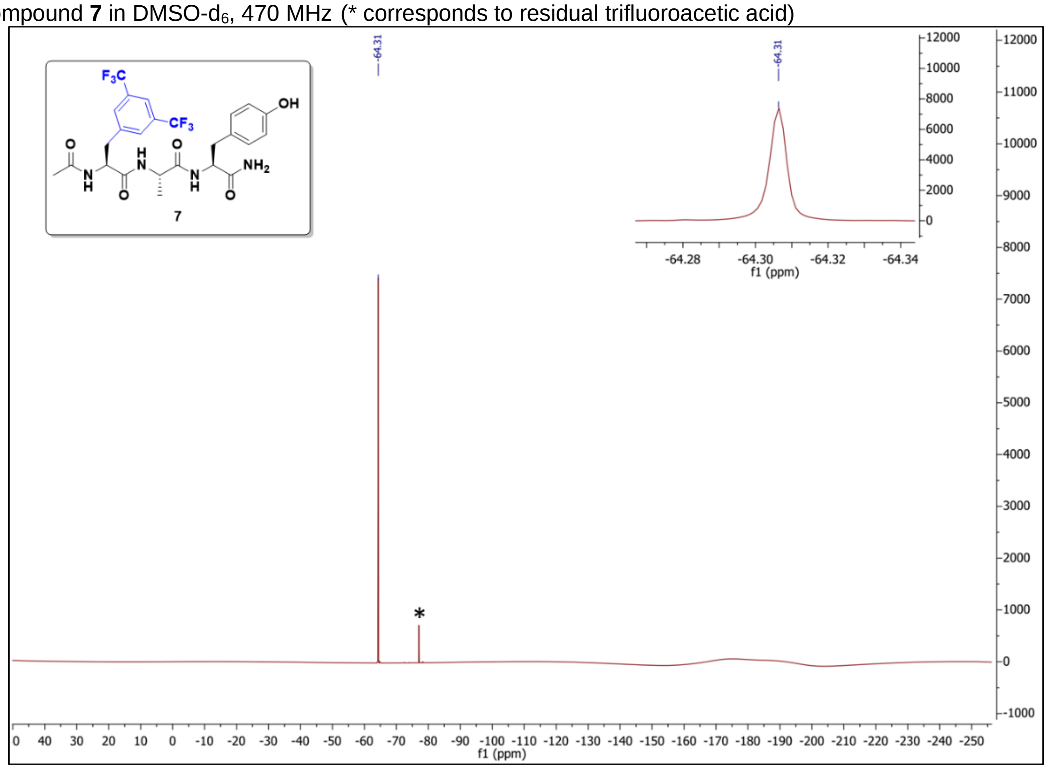

^{1}H NMR of compound **8** in DMSO-d$_6$, 700 MHz

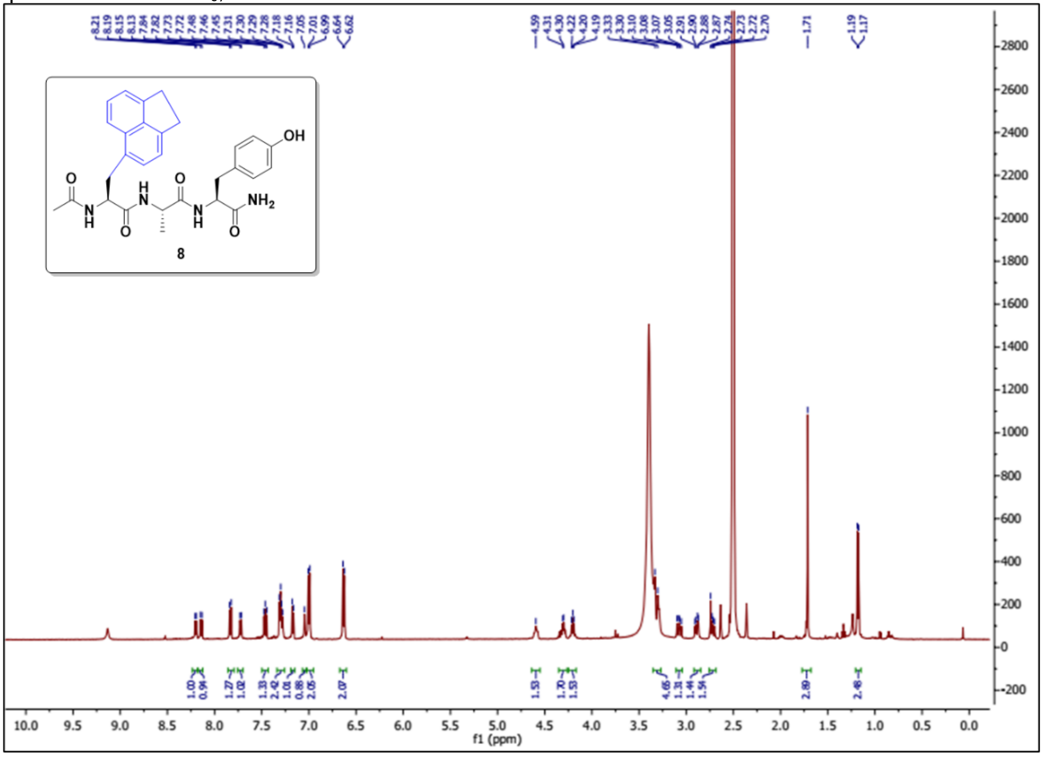

^{1}H NMR of compound **9** in DMSO-d$_6$, 700 MHz

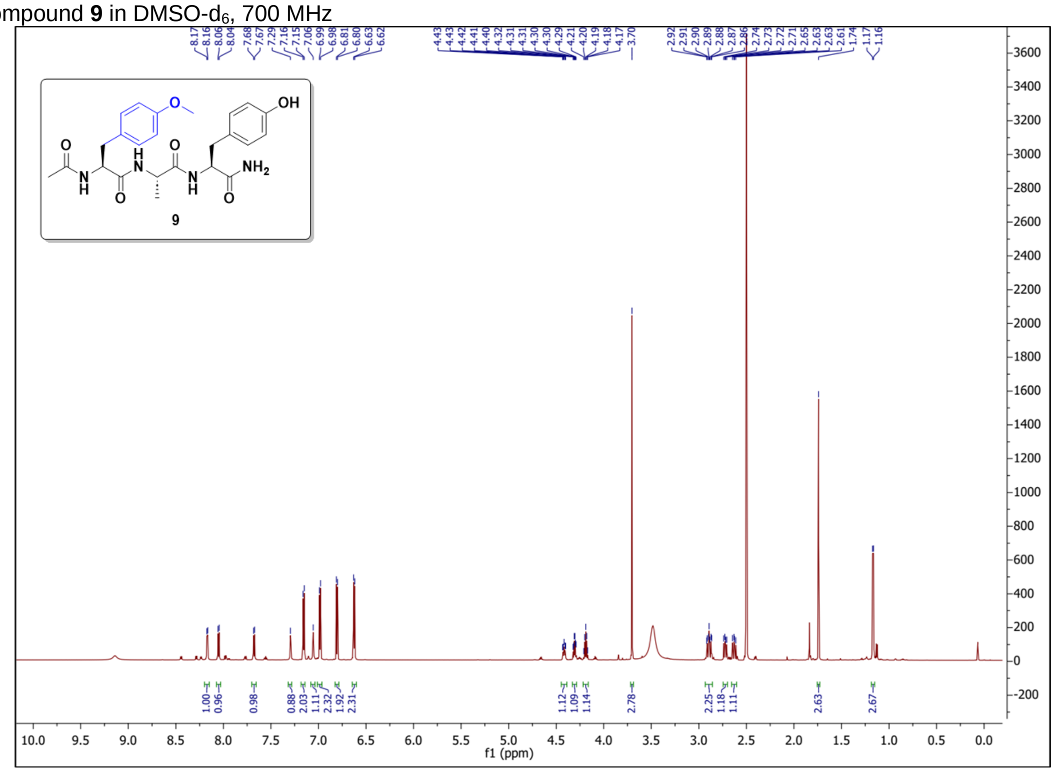

^{13}C NMR of compound **9** in DMSO-d$_6$, 176 MHz

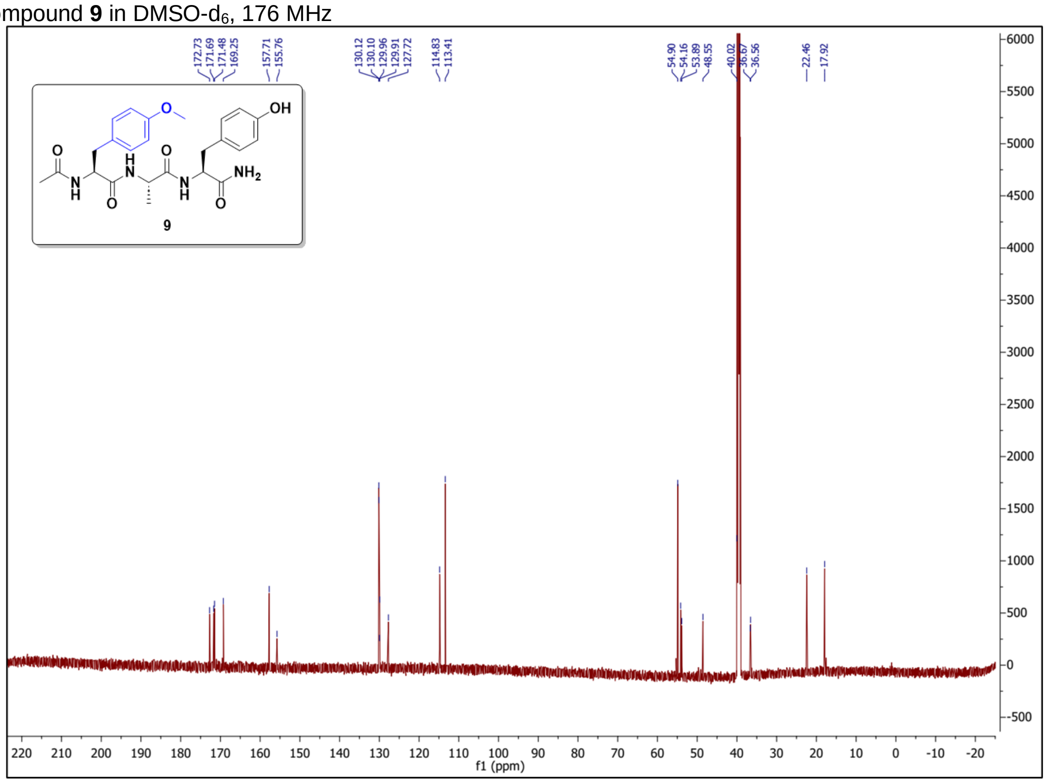

^{1}H NMR of compound **10** in DMSO-d$_6$, 700 MHz

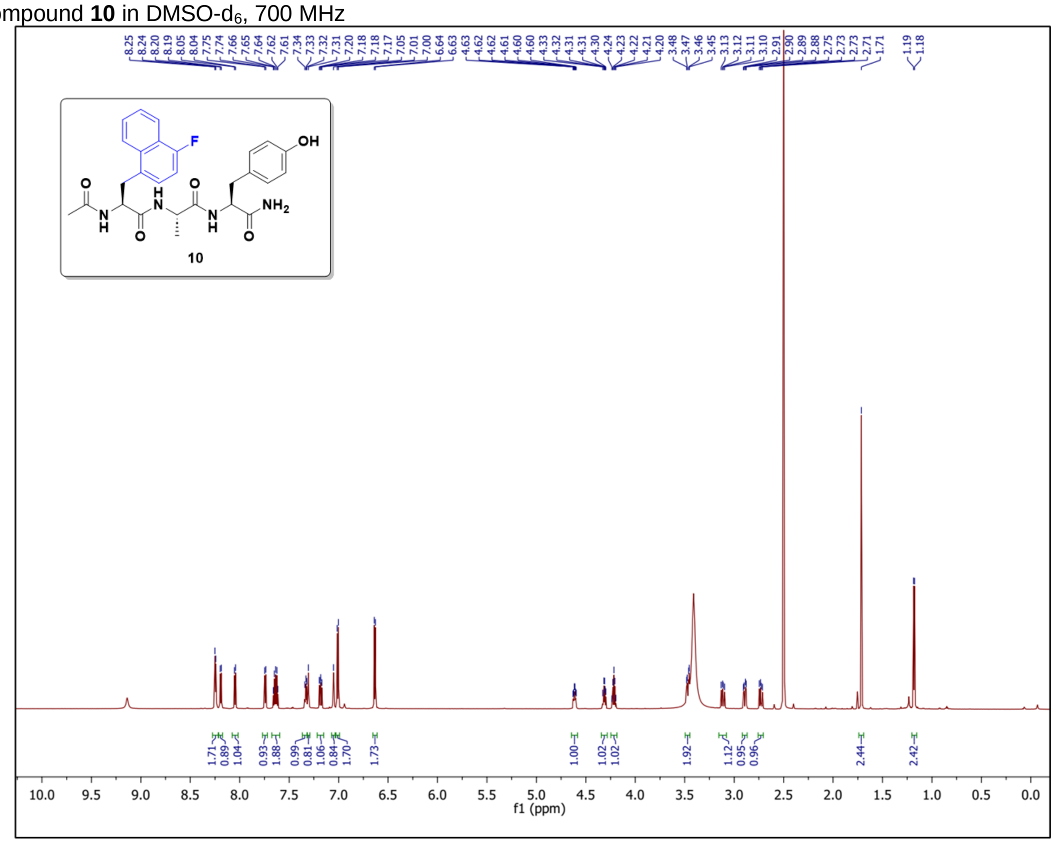

^{13}C NMR of compound **10** in DMSO-d$_6$, 176 MHz

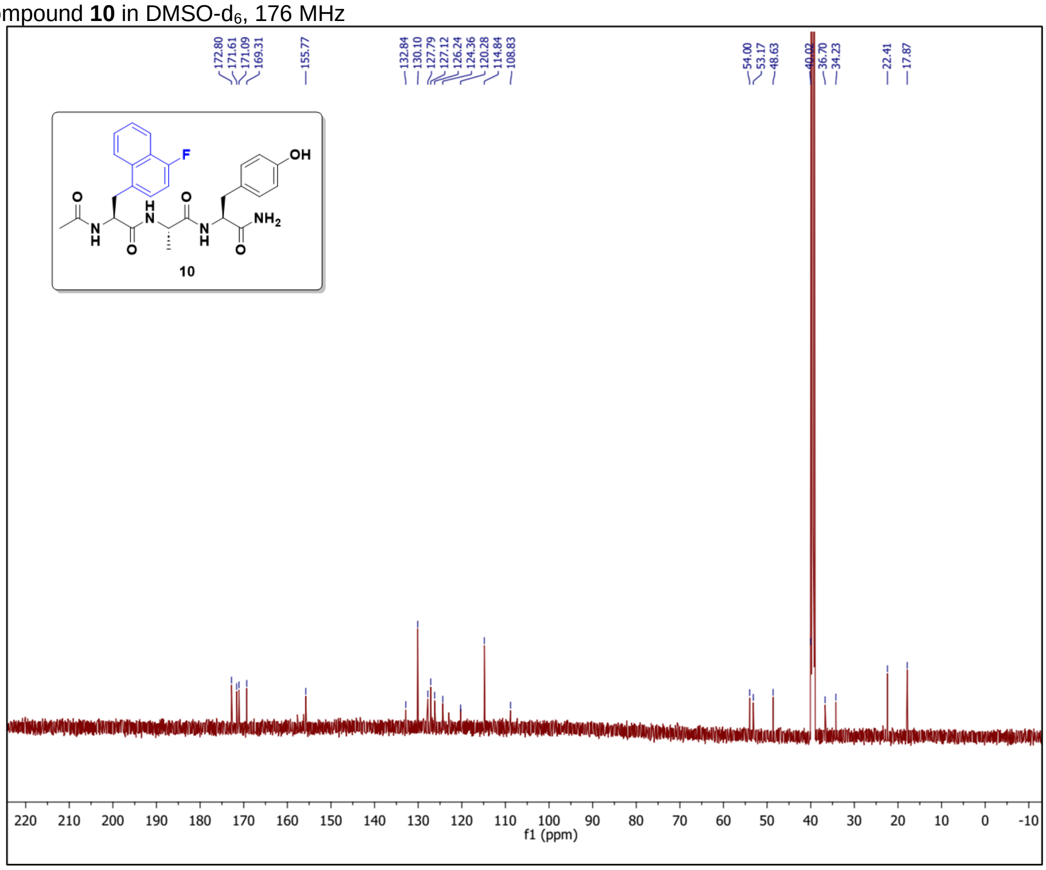

^{19}F NMR of compound **10** in DMSO-d$_6$, 470 MHz (* corresponds to residual trifluoroacetic acid)

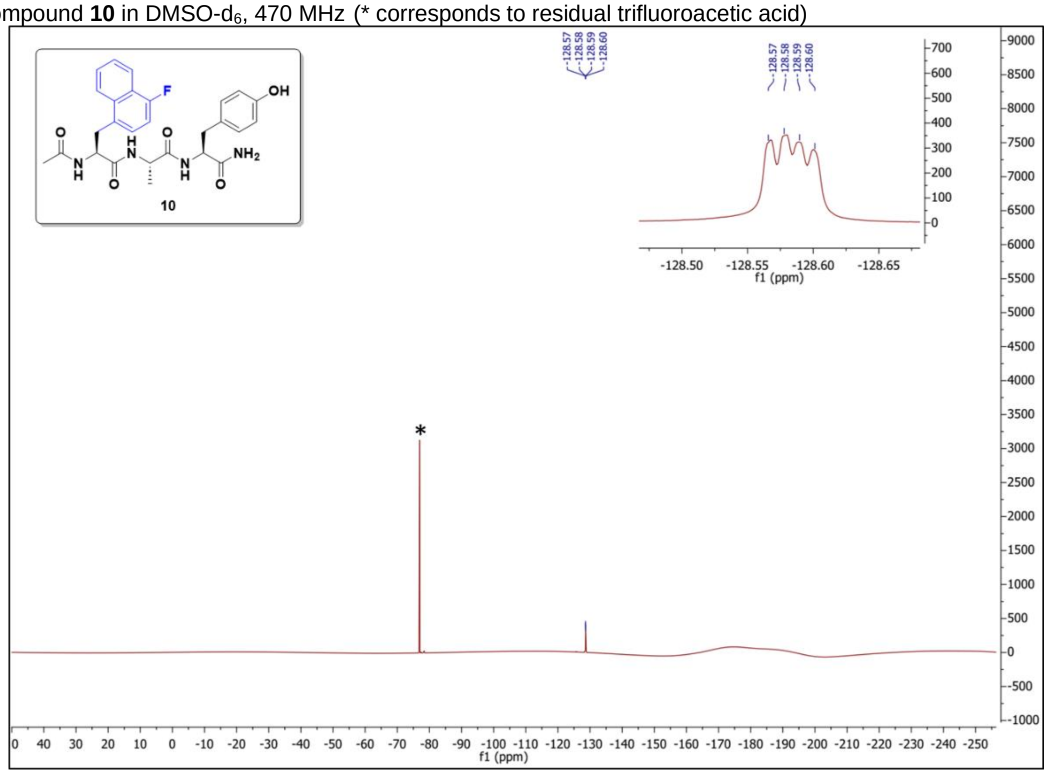

^1H NMR of compound **11** in DMSO-d_6, 700 MHz

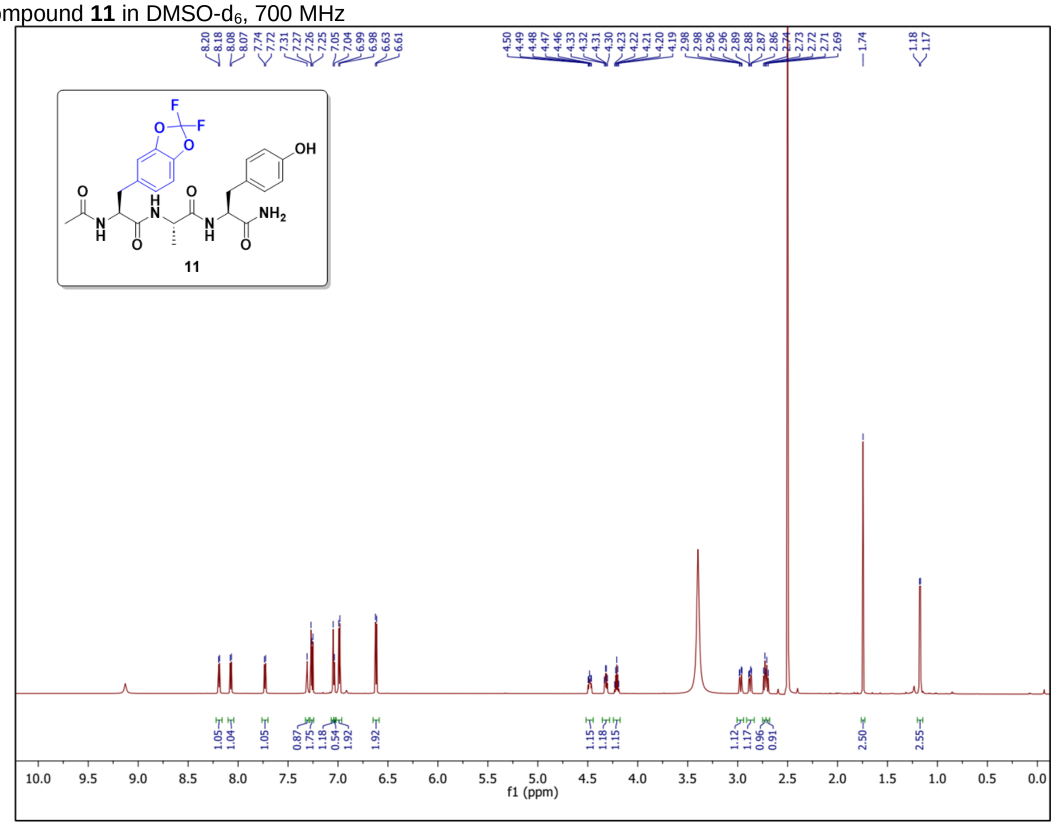

^{19}F NMR of compound **11** in DMSO-d_6, 470 MHz (* corresponds to residual trifluoroacetic acid)

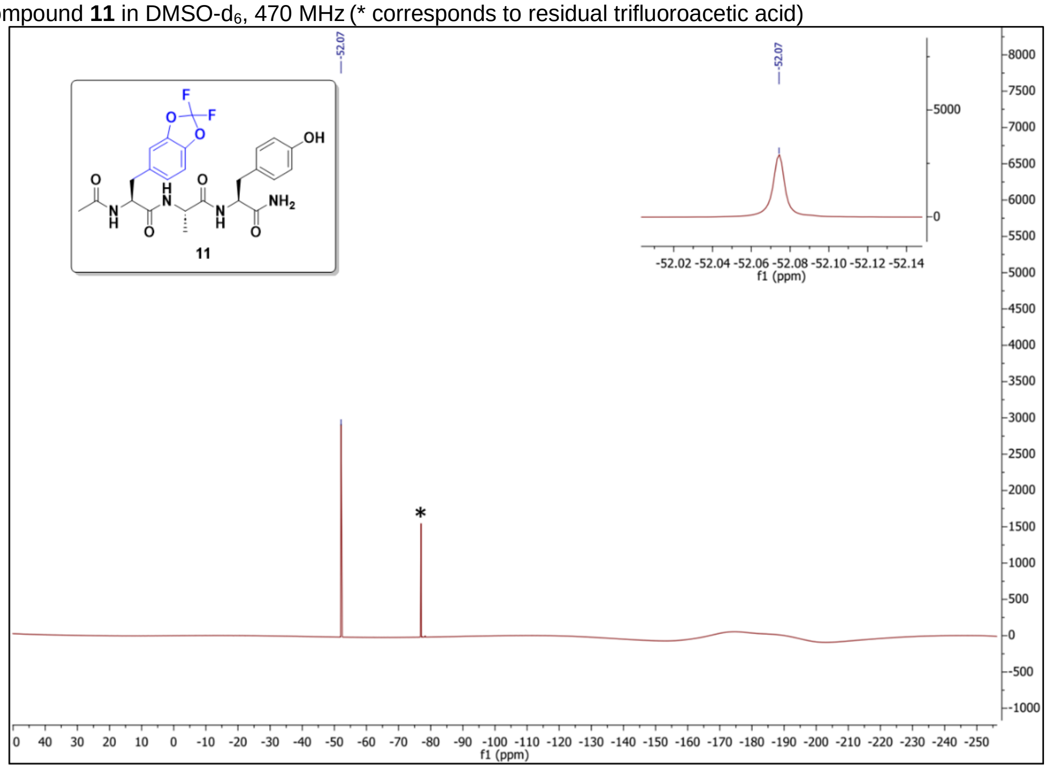

^{13}C NMR of compound **11** in DMSO-d$_6$, 176 MHz

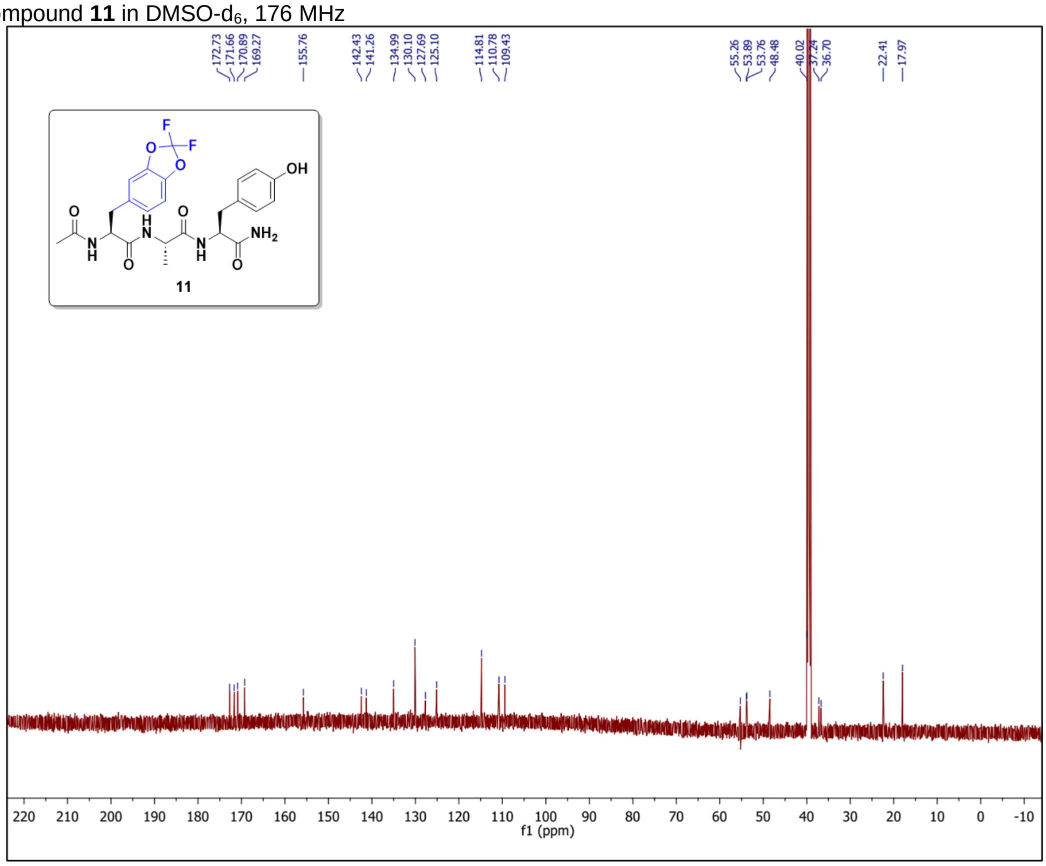

^{1}H NMR of compound **12** in DMSO-d$_6$, 700 MHz

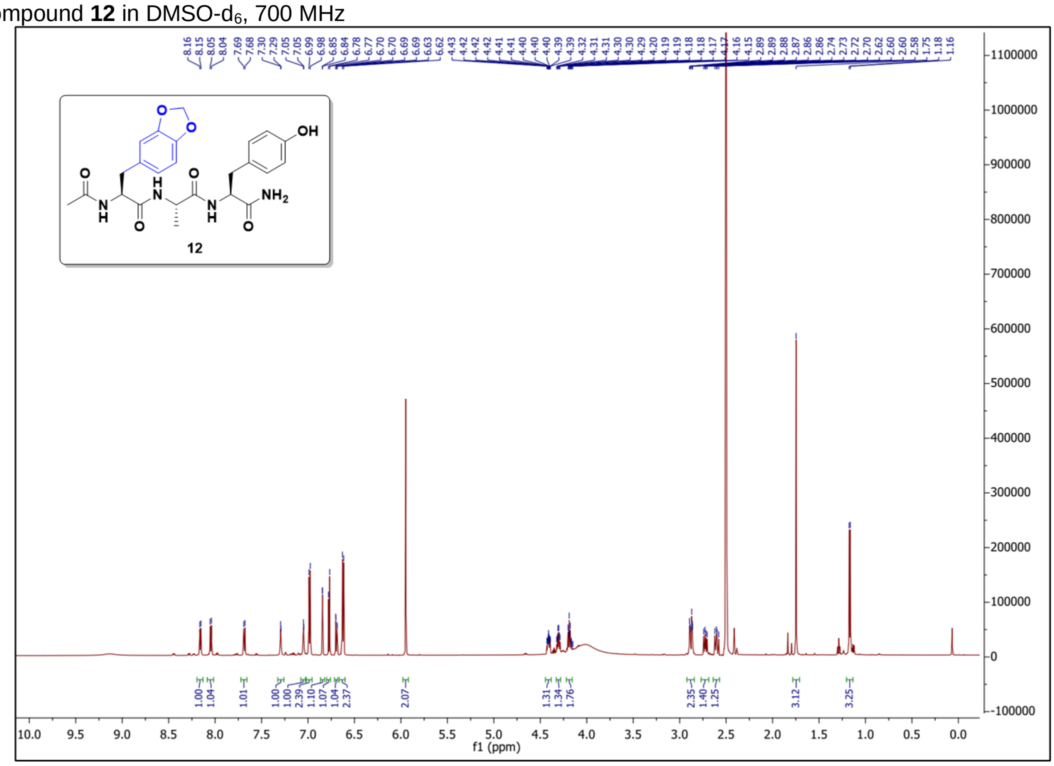

^{13}C NMR of compound **12** in DMSO-d$_6$, 176 MHz

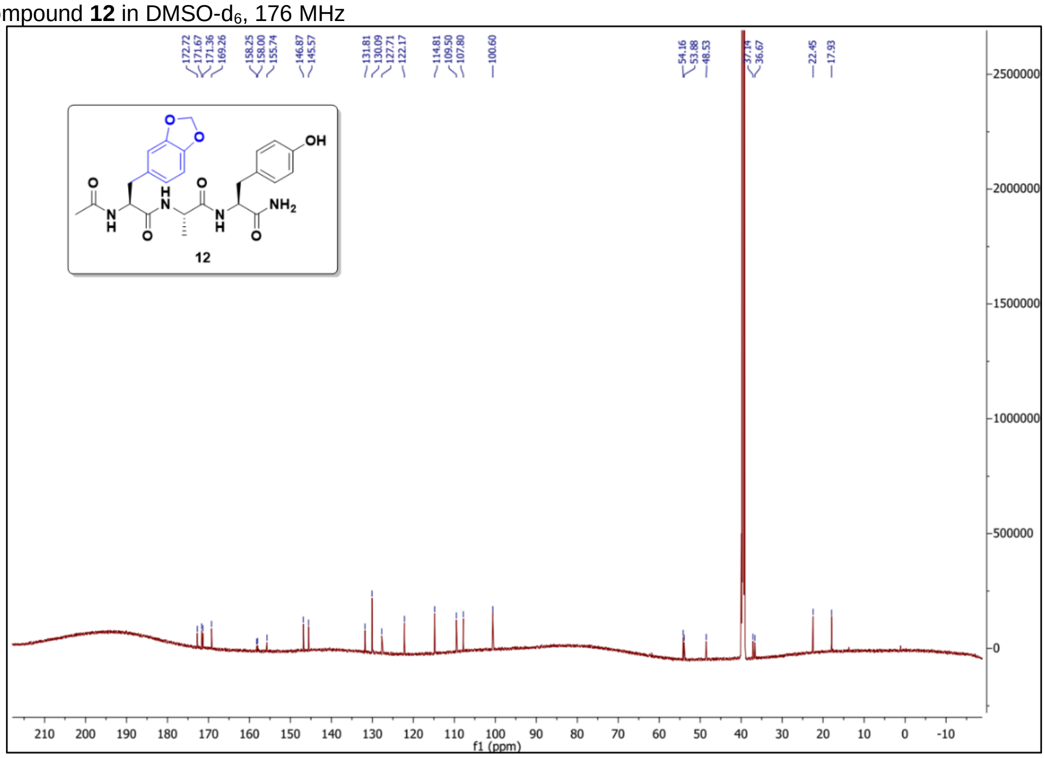

^{1}H NMR of compound **13** in DMSO-d$_6$, 700 MHz

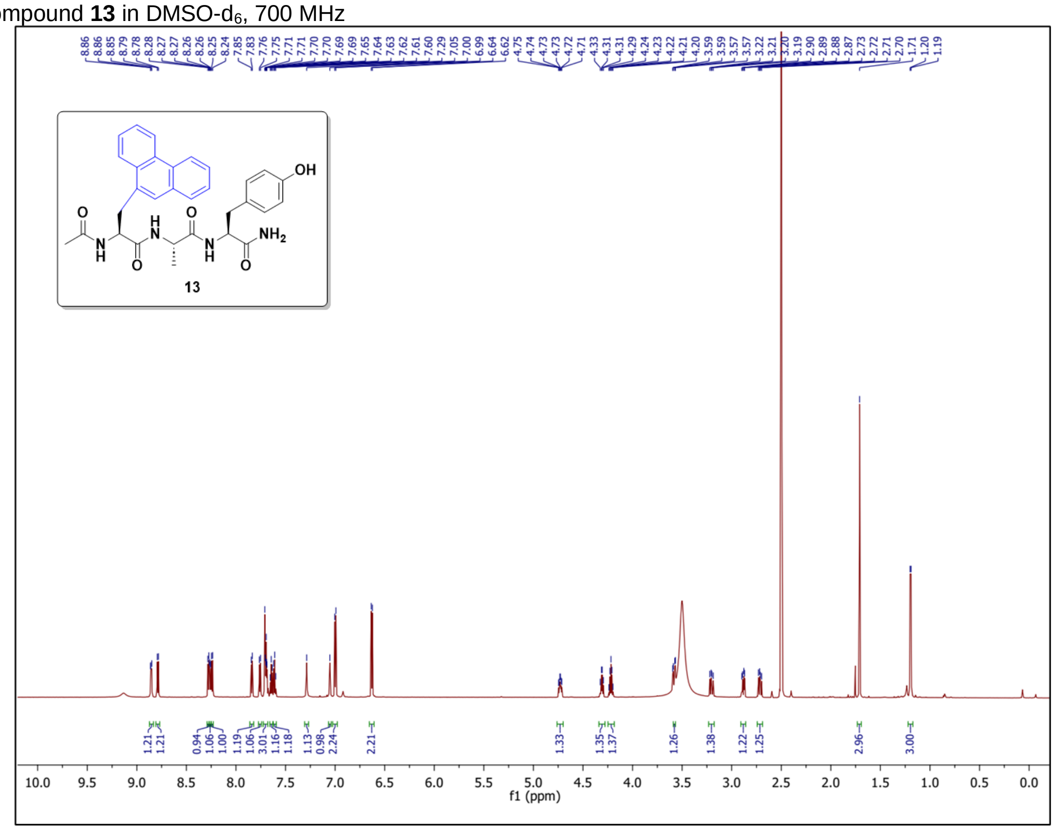

^{13}C NMR of compound **13** in DMSO-d$_6$, 176 MHz

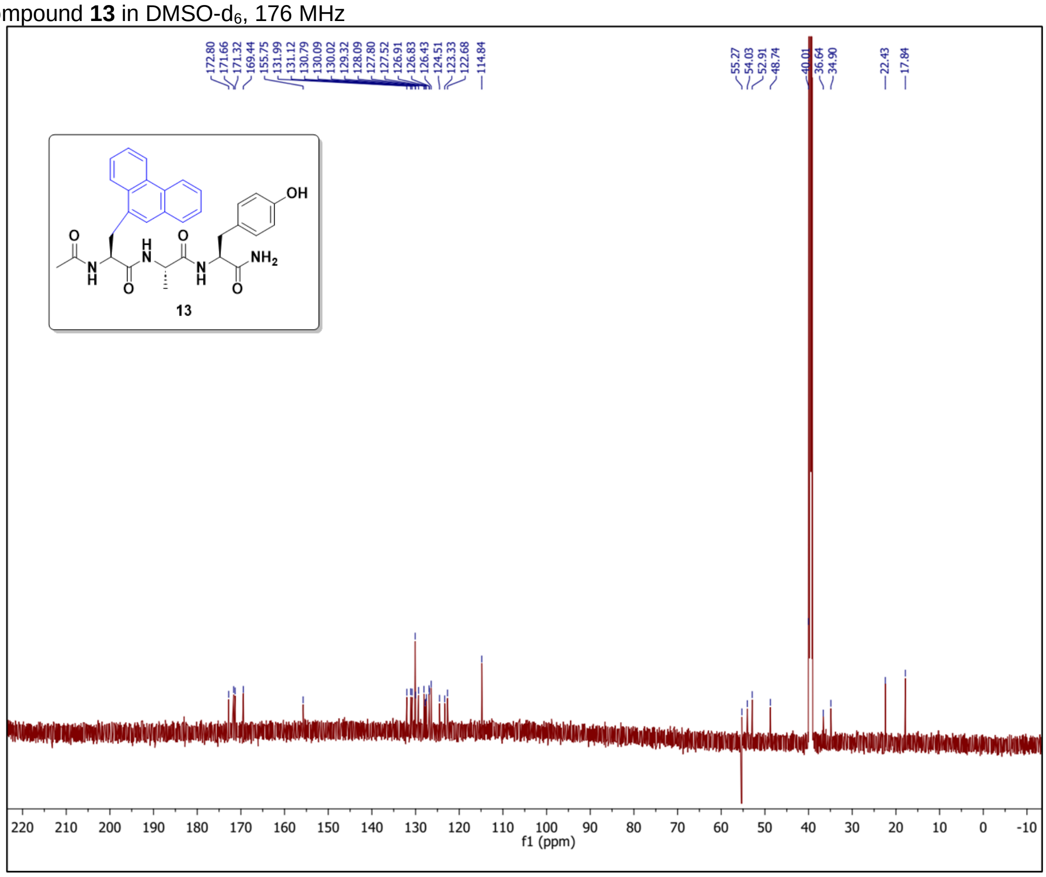

^{1}H NMR of compound **14** in DMSO-d$_6$, 700 MHz

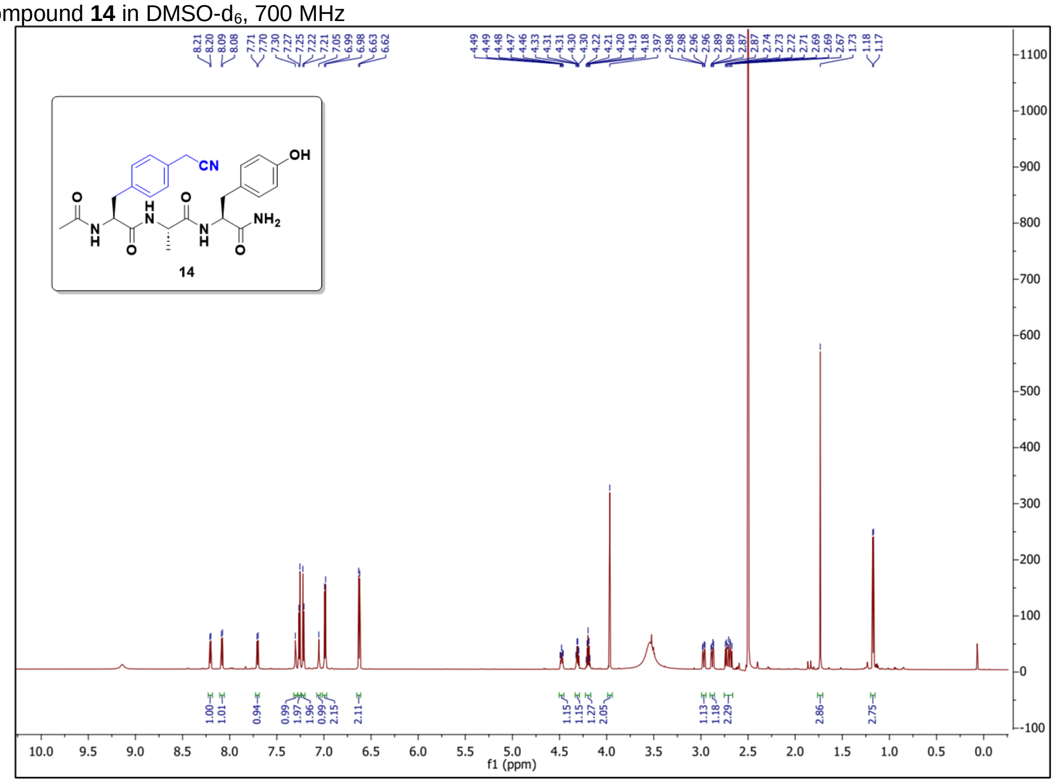

^{13}C NMR of compound **14** in DMSO-d_6, 176 MHz

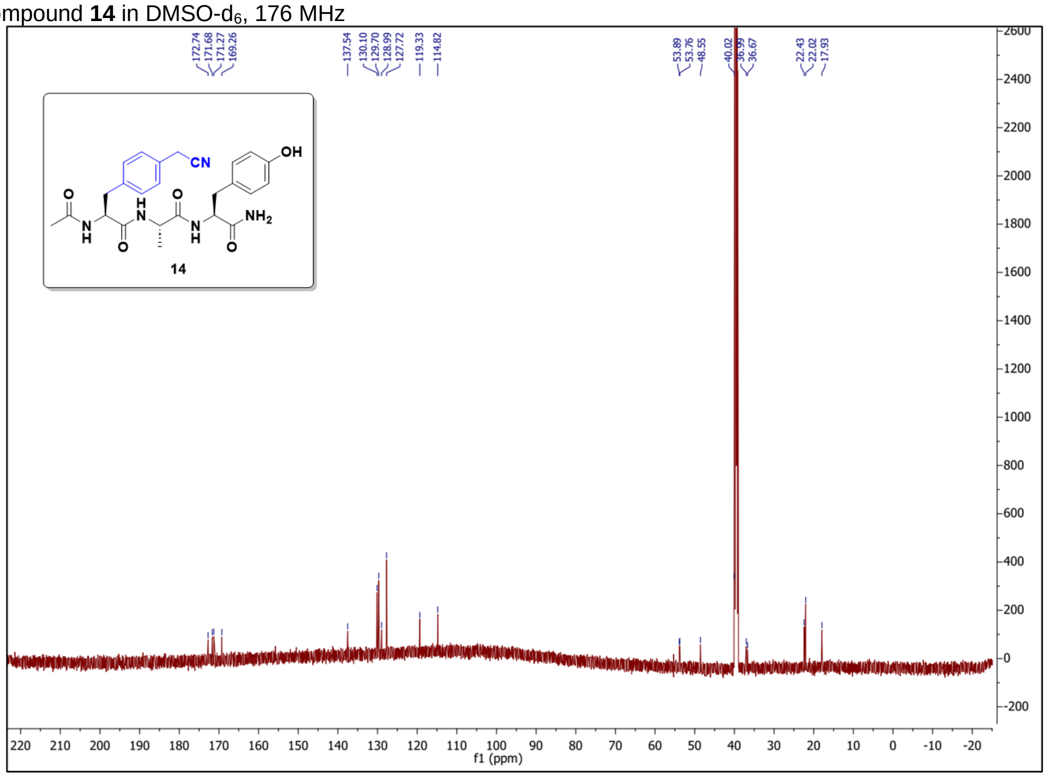

^{1}H NMR of compound **15** in DMSO-d_6, 700 MHz

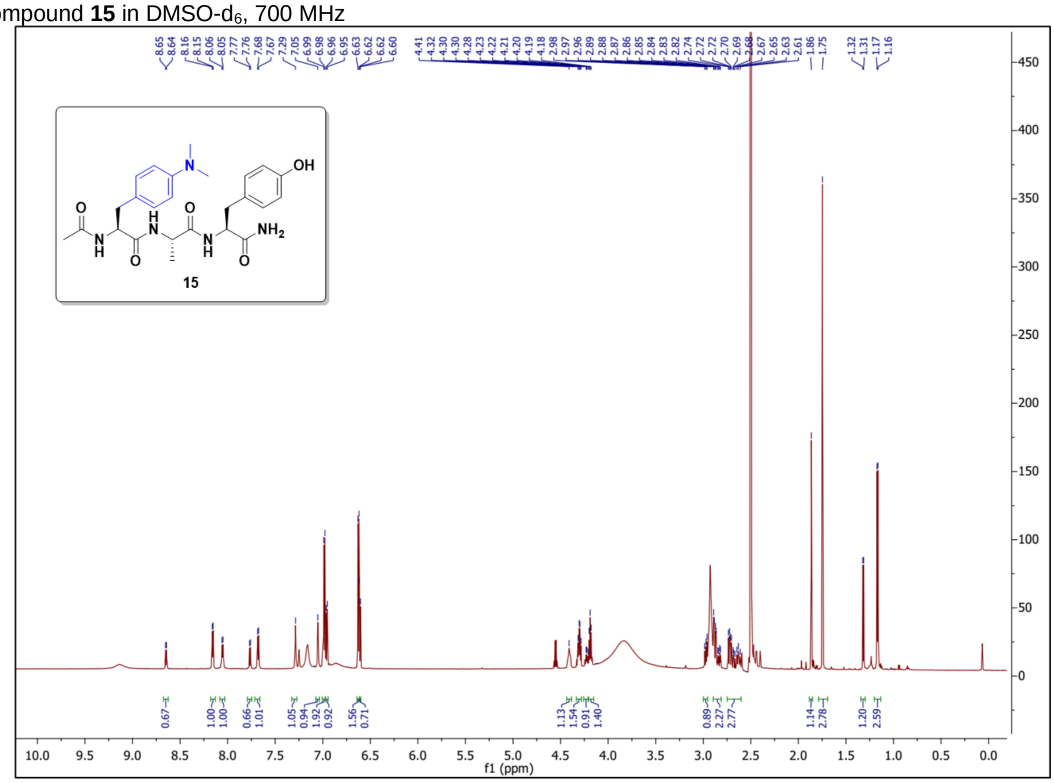

^{1}H NMR of compound **16** in DMSO-d$_6$, 700 MHz

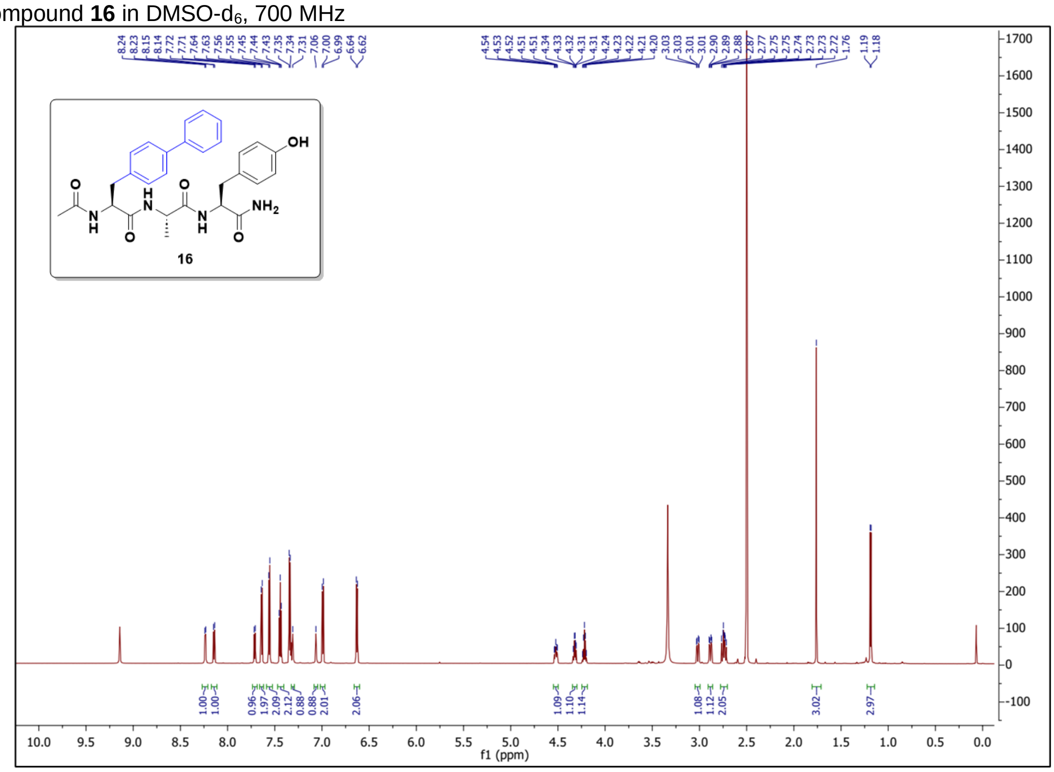

^{13}C NMR of compound **16** in DMSO-d$_6$, 176 MHz

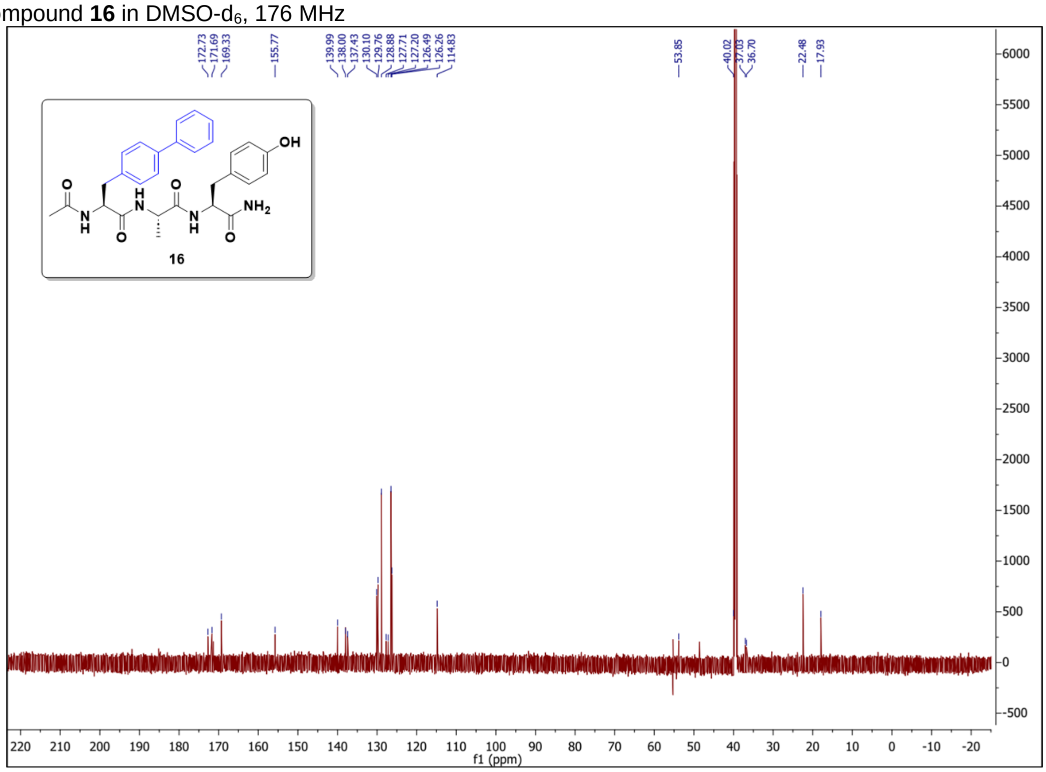

^1H NMR of compound **17** in DMSO-d_6, 700 MHz

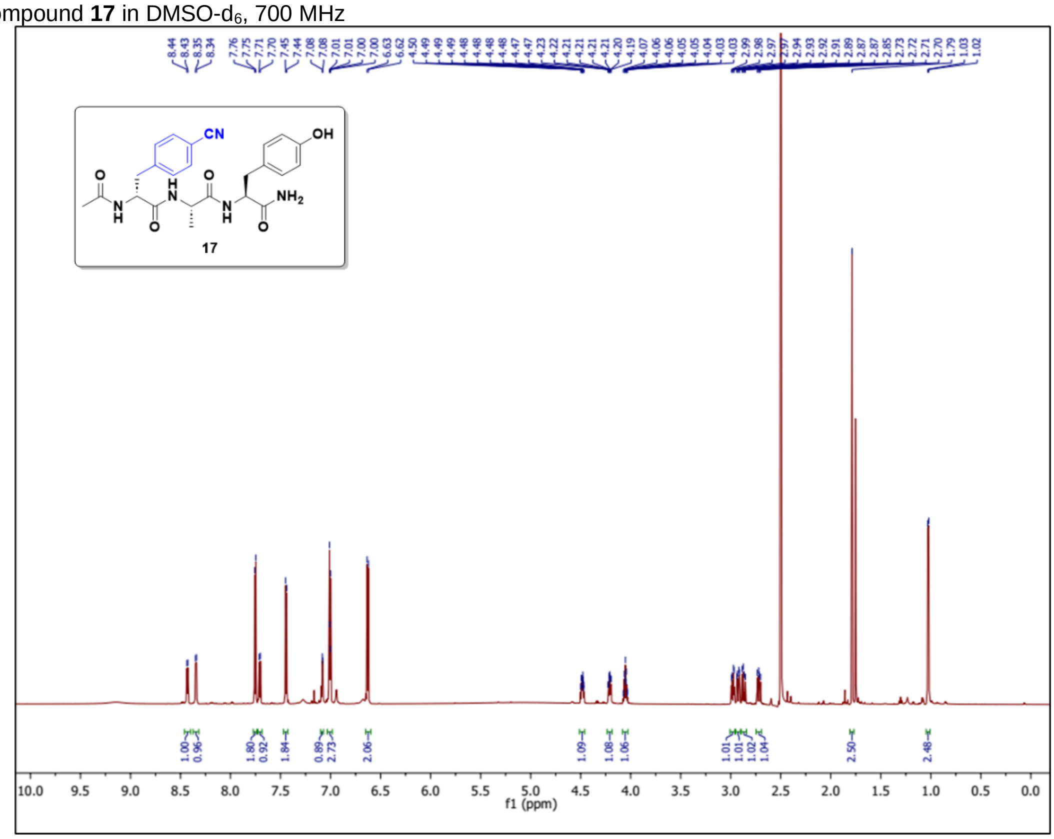

^{13}C NMR of compound **17** in DMSO-d_6, 176 MHz

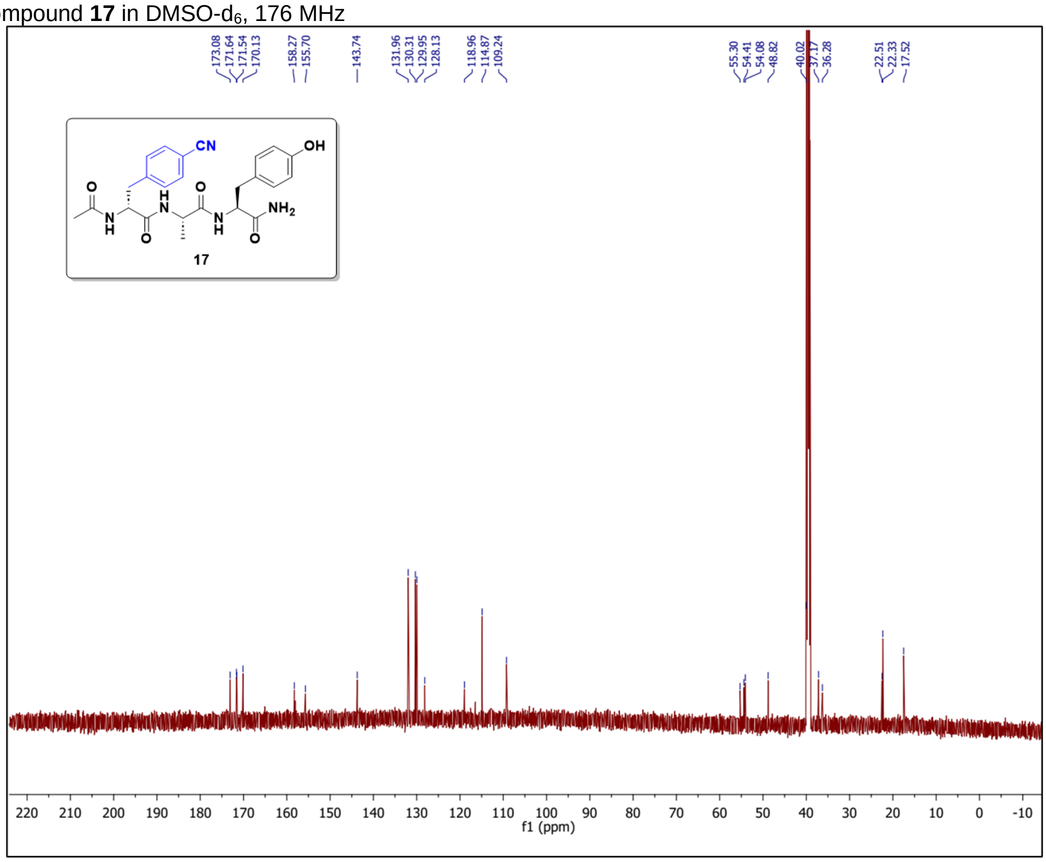

^{1}H NMR of compound **18** in DMSO-d$_6$, 700 MHz

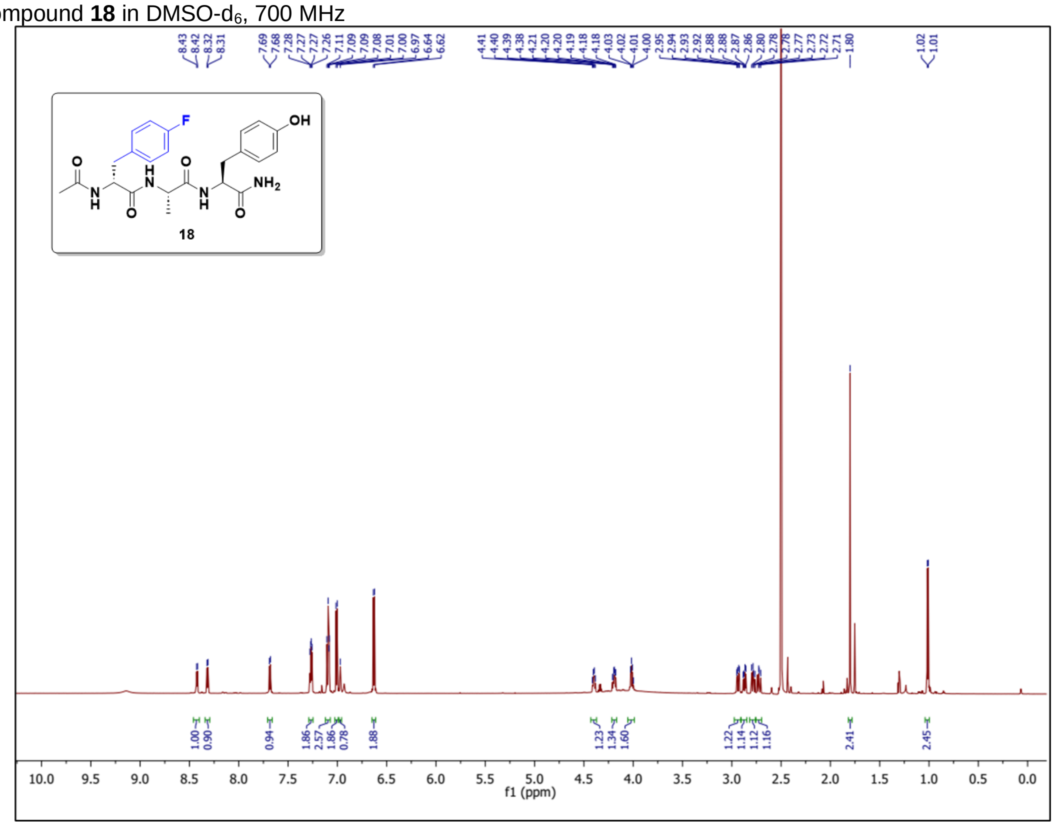

^{13}C NMR of compound **18** in DMSO-d$_6$, 176 MHz

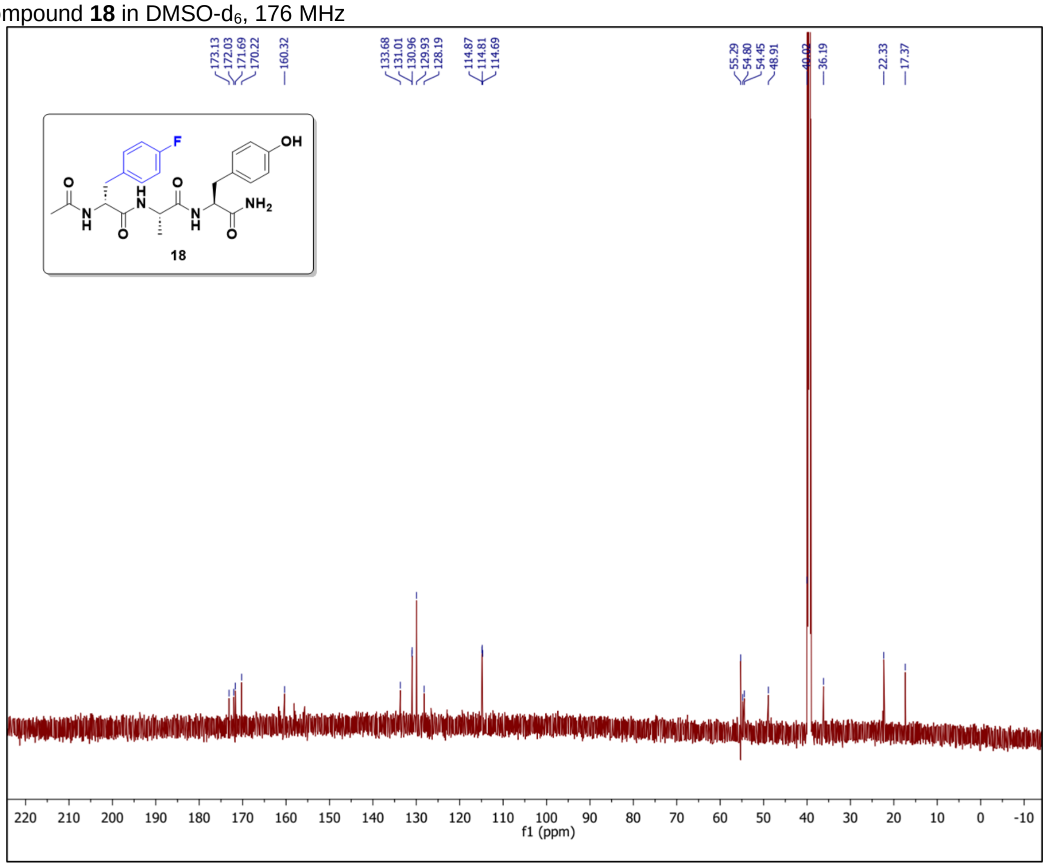

^{19}F NMR of compound **18** in DMSO-d_6, 470 MHz (* corresponds to residual trifluoroacetic acid)

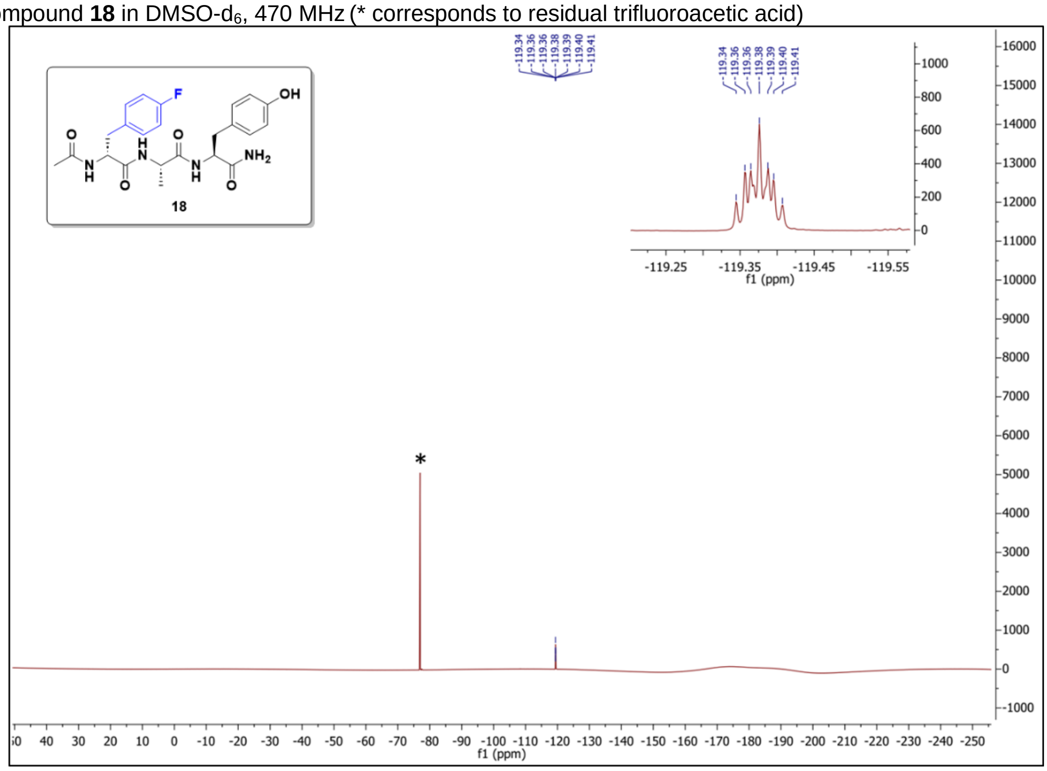

^{1}H NMR of compound **19** in DMSO-d_6, 700 MHz

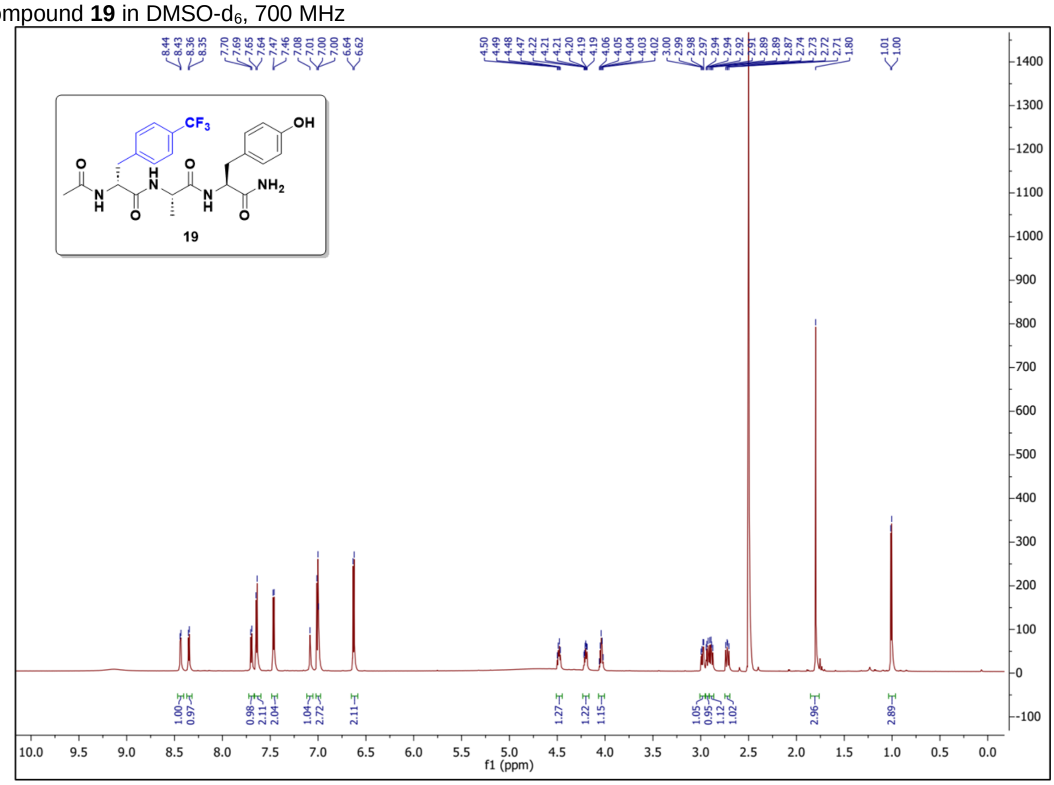

^{13}C NMR of compound **19** in DMSO-d_6, 176 MHz

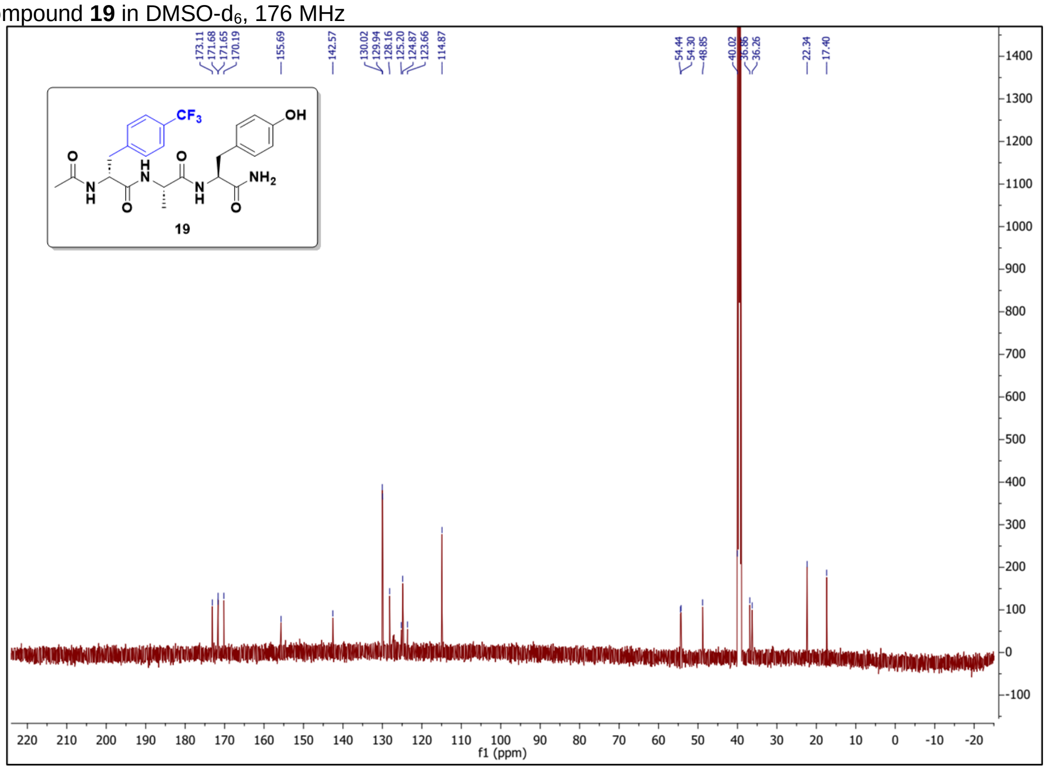

^{19}F NMR of compound **19** in DMSO-d_6, 470 MHz (* corresponds to residual trifluoroacetic acid)

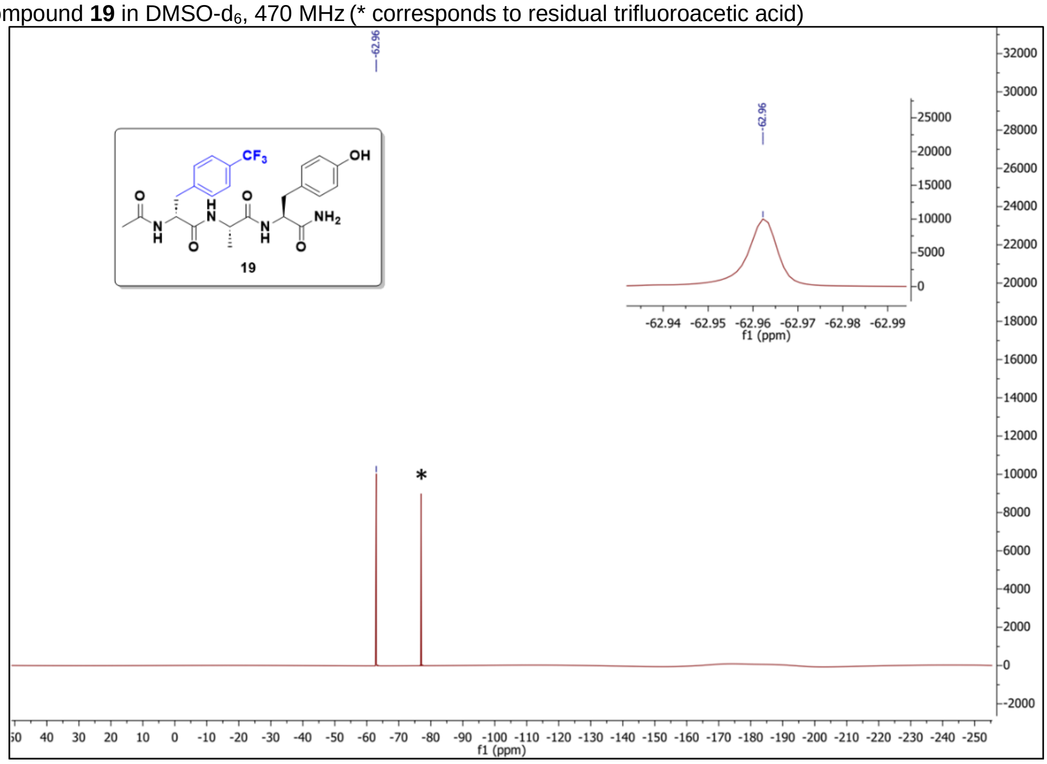

^{1}H NMR of compound **20** in DMSO-d$_6$, 700 MHz

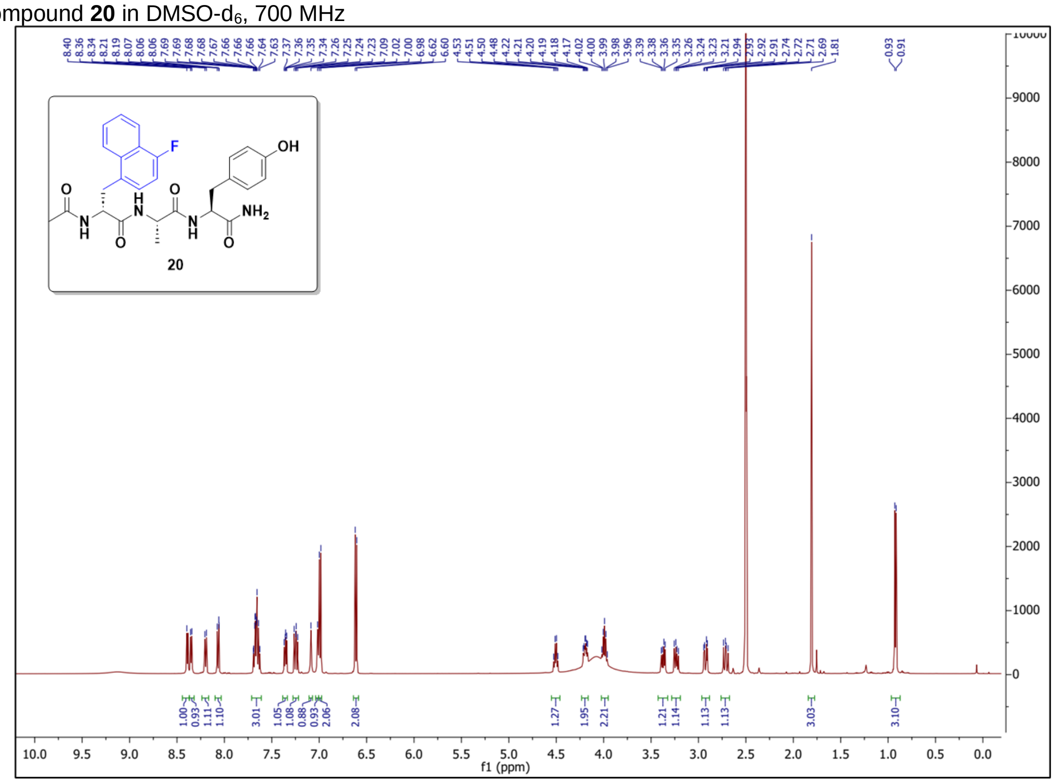

^{13}C NMR of compound **20** in DMSO-d$_6$, 176 MHz

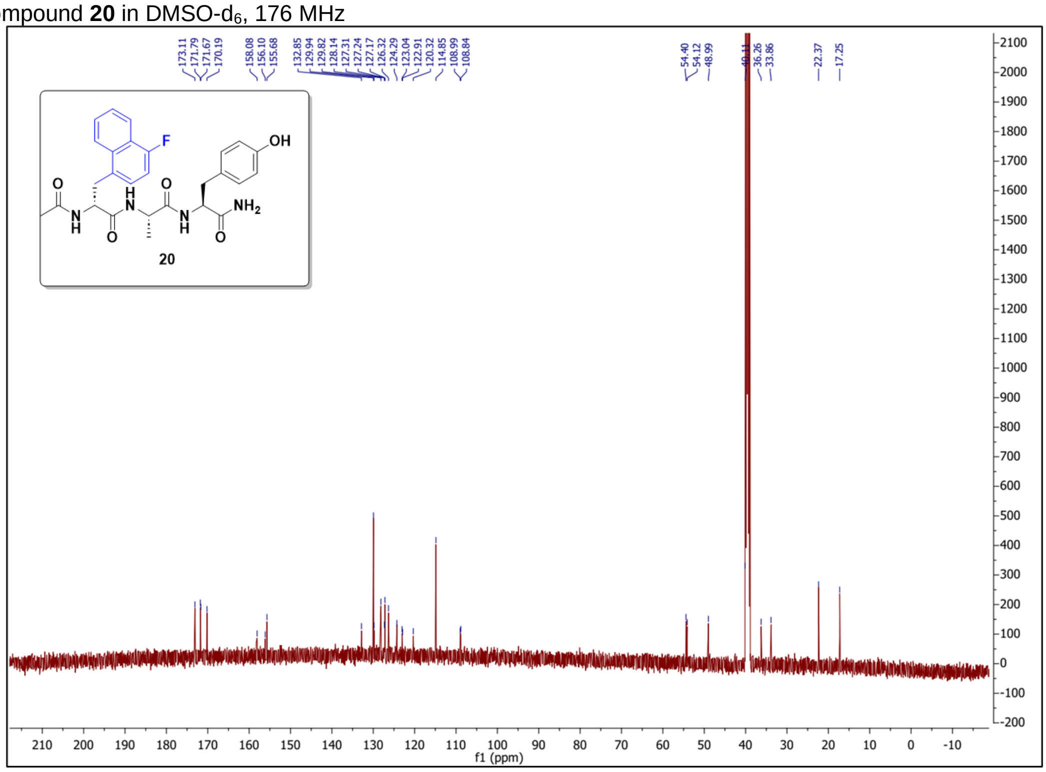

^{19}F NMR of compound **20** in DMSO-d$_6$, 470 MHz (* corresponds to residual trifluoroacetic acid)

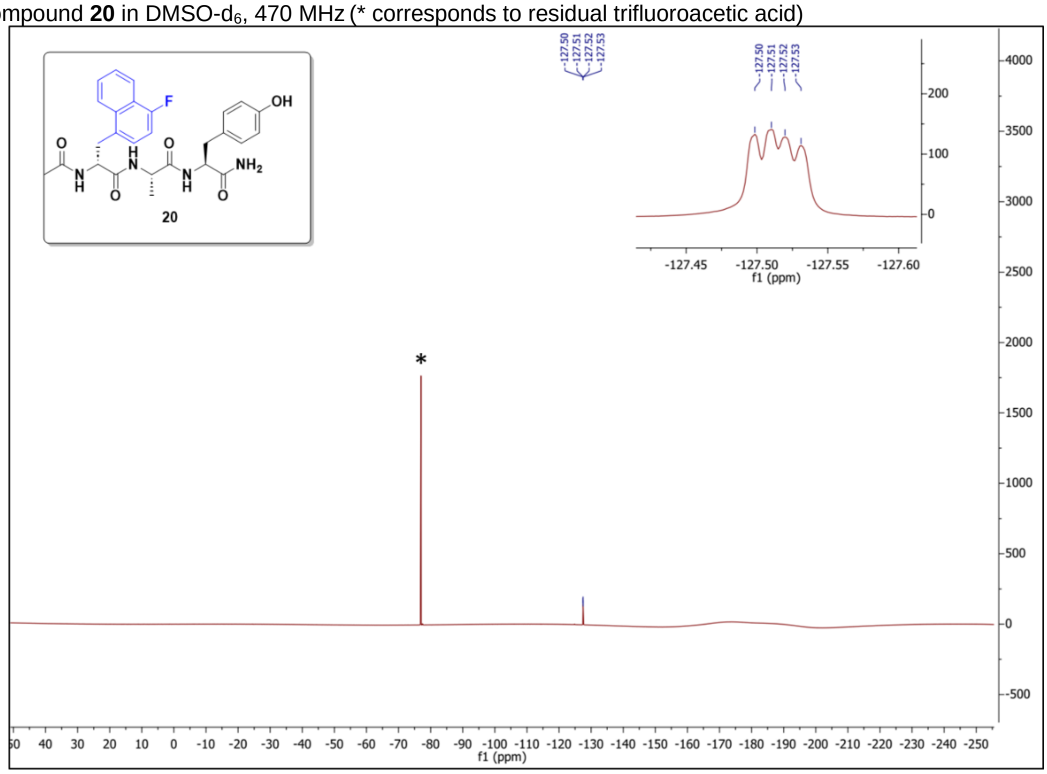

^{1}H NMR of compound **21** in DMSO-d$_6$, 700 MHz

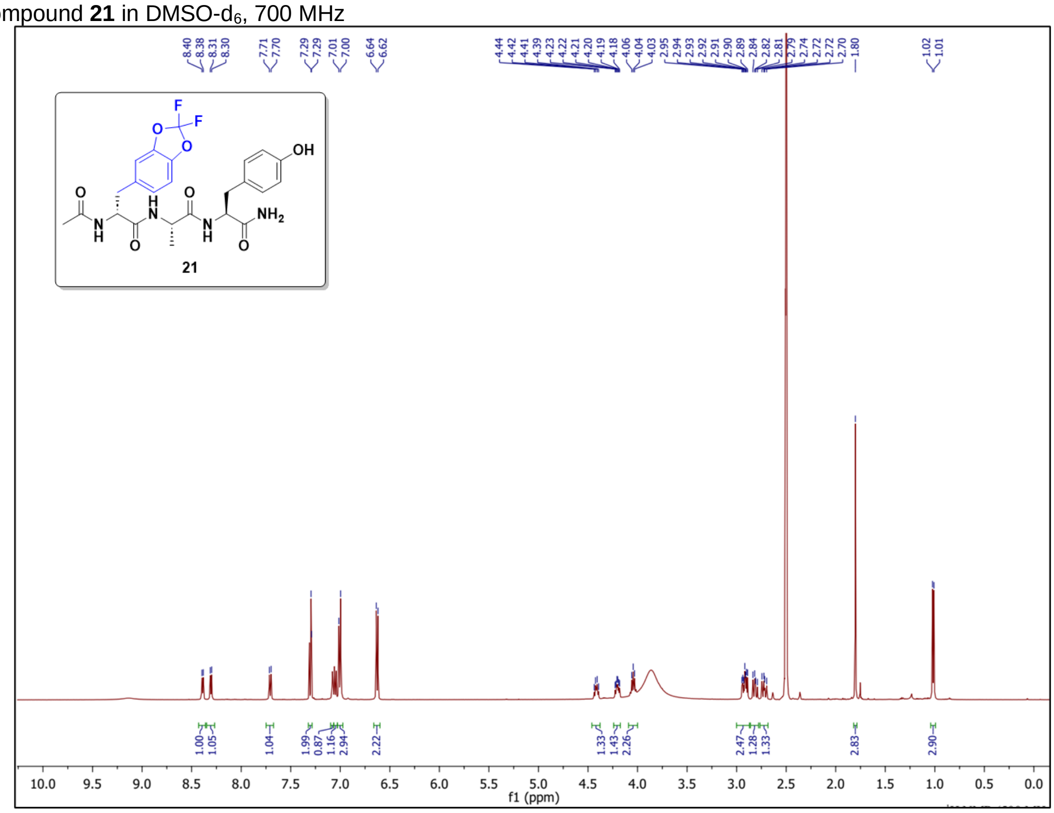

^{13}C NMR of compound **21** in DMSO-d_6, 176 MHz

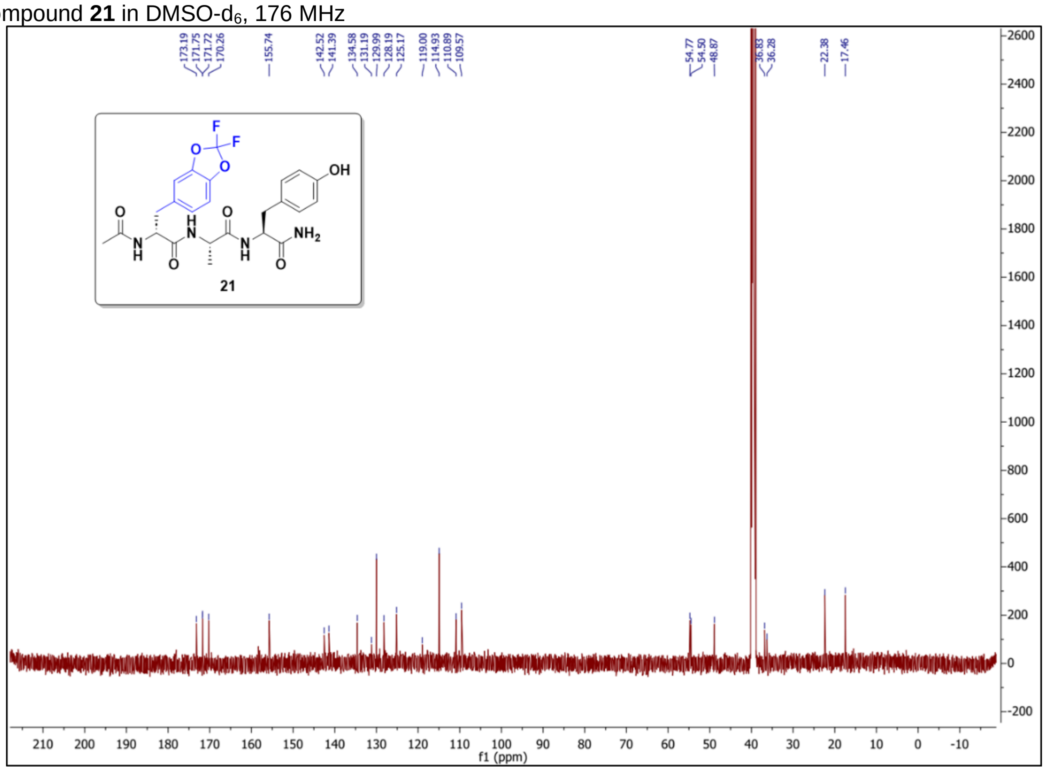

^{19}F NMR of compound **21** in DMSO-d_6, 470 MHz (* corresponds to residual trifluoroacetic acid)

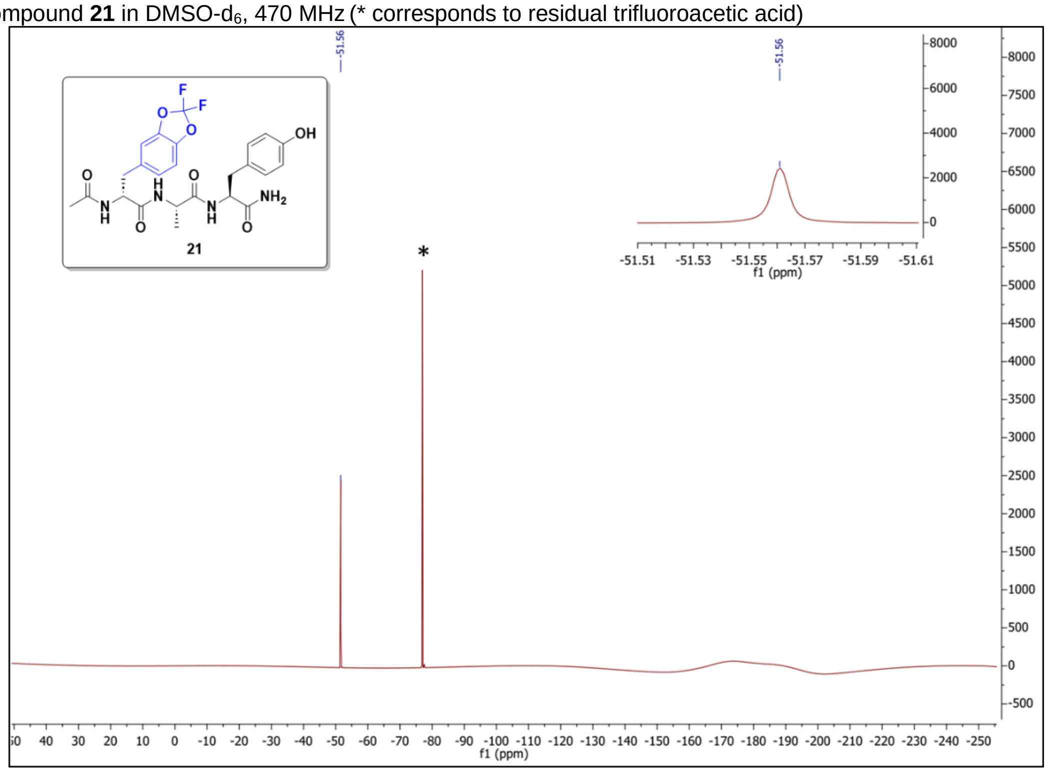

^{1}H NMR of compound **22** in DMSO-d_6, 700 MHz

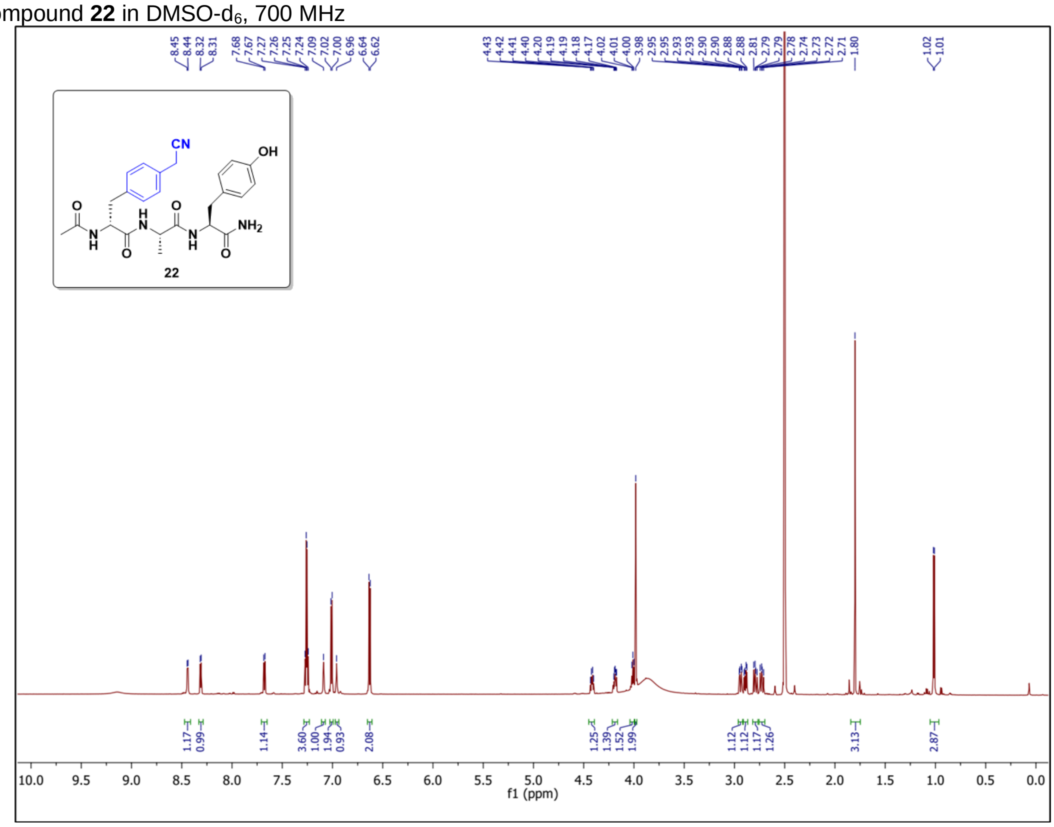

^{13}C NMR of compound **22** in DMSO-d_6, 176 MHz

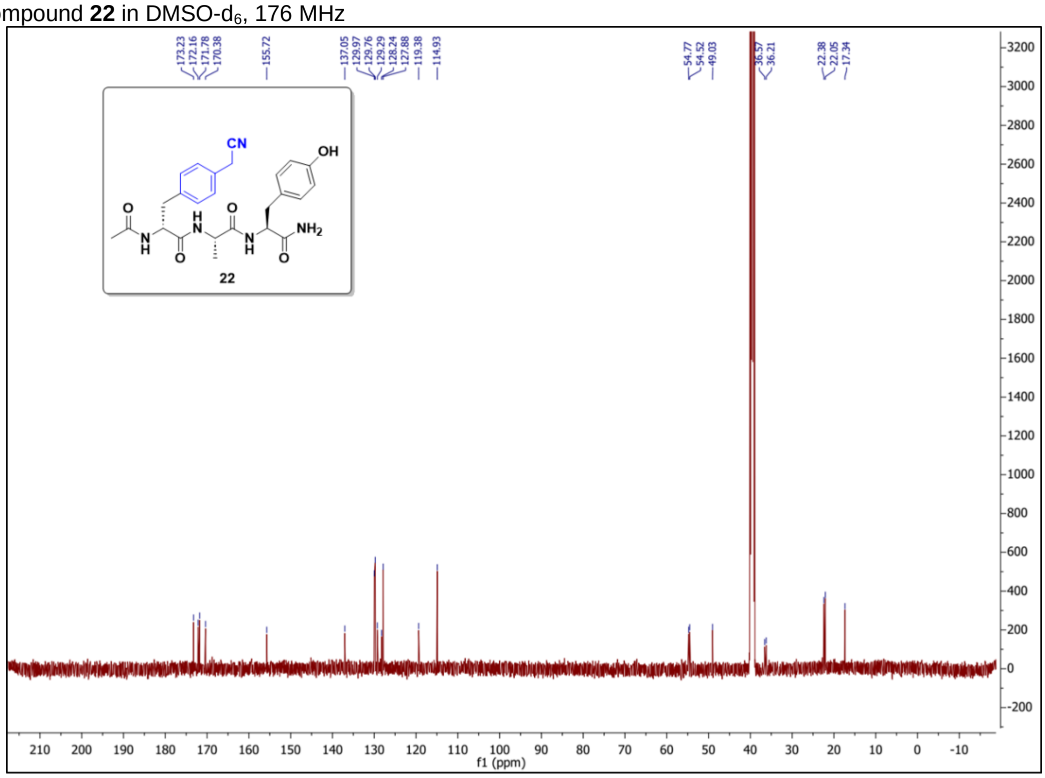

^{1}H NMR of compound **23** in DMSO-d_6, 700 MHz

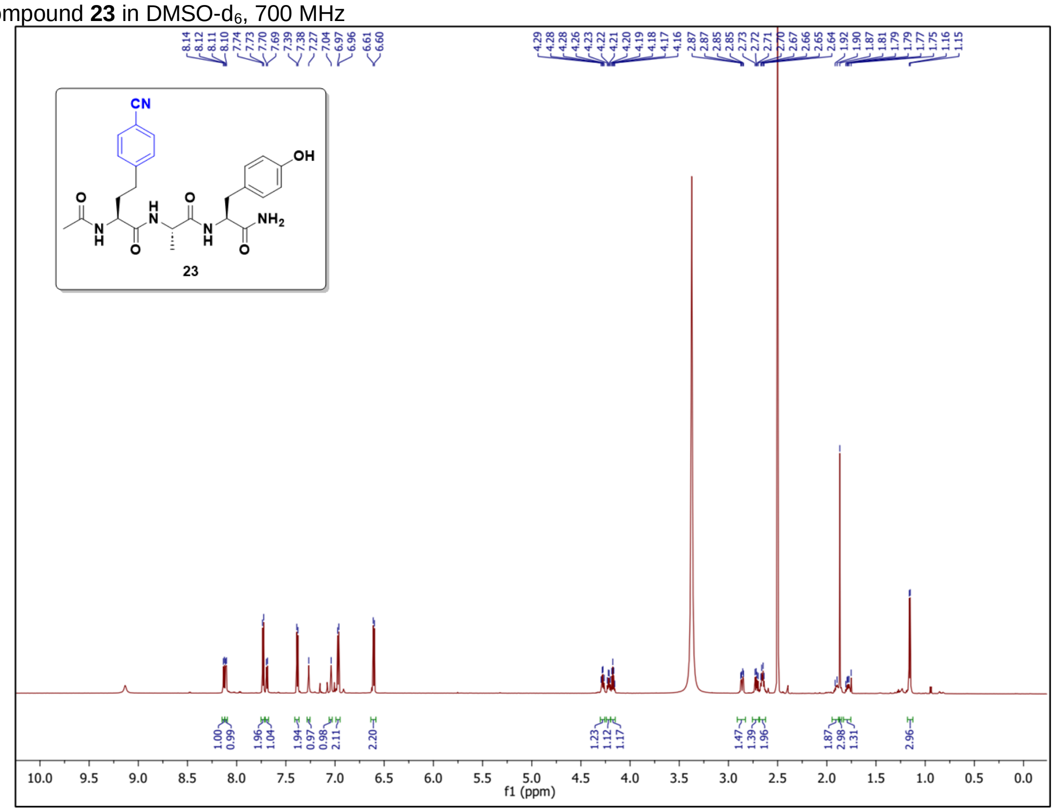

^{13}C NMR of compound **23** in DMSO-d_6, 176 MHz

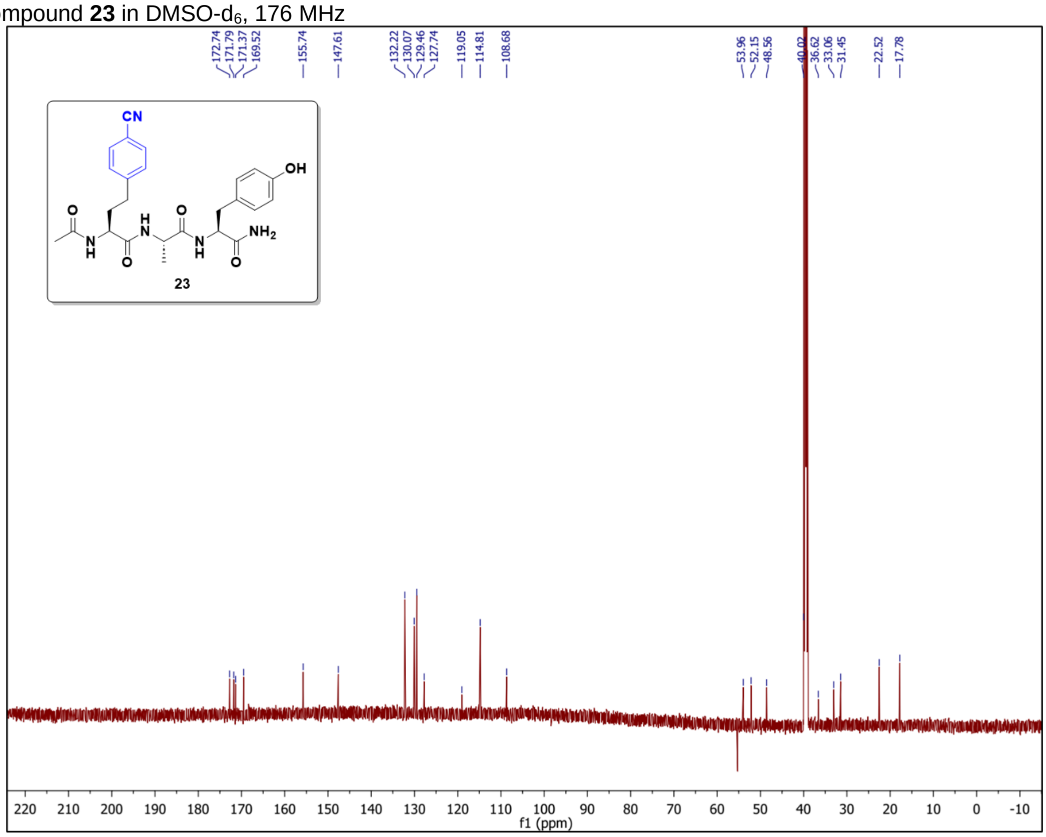

^{1}H NMR of compound **24** in DMSO-d$_6$, 700 MHz

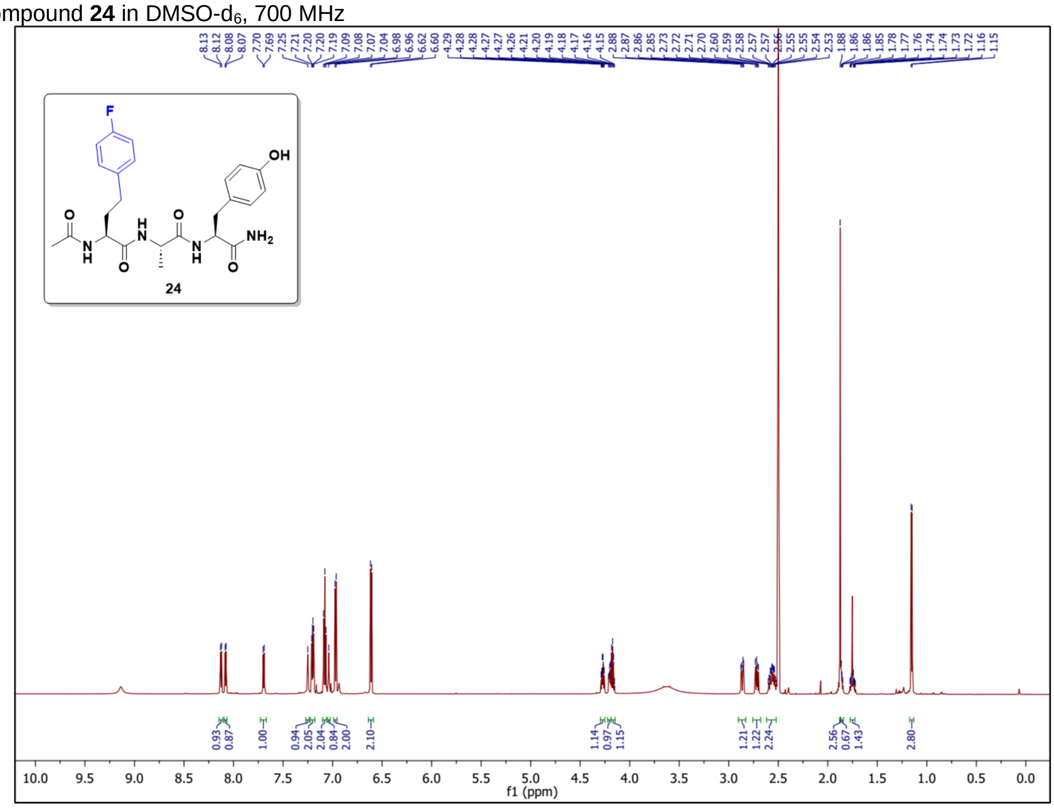

^{13}C NMR of compound **24** in DMSO-d$_6$, 176 MHz

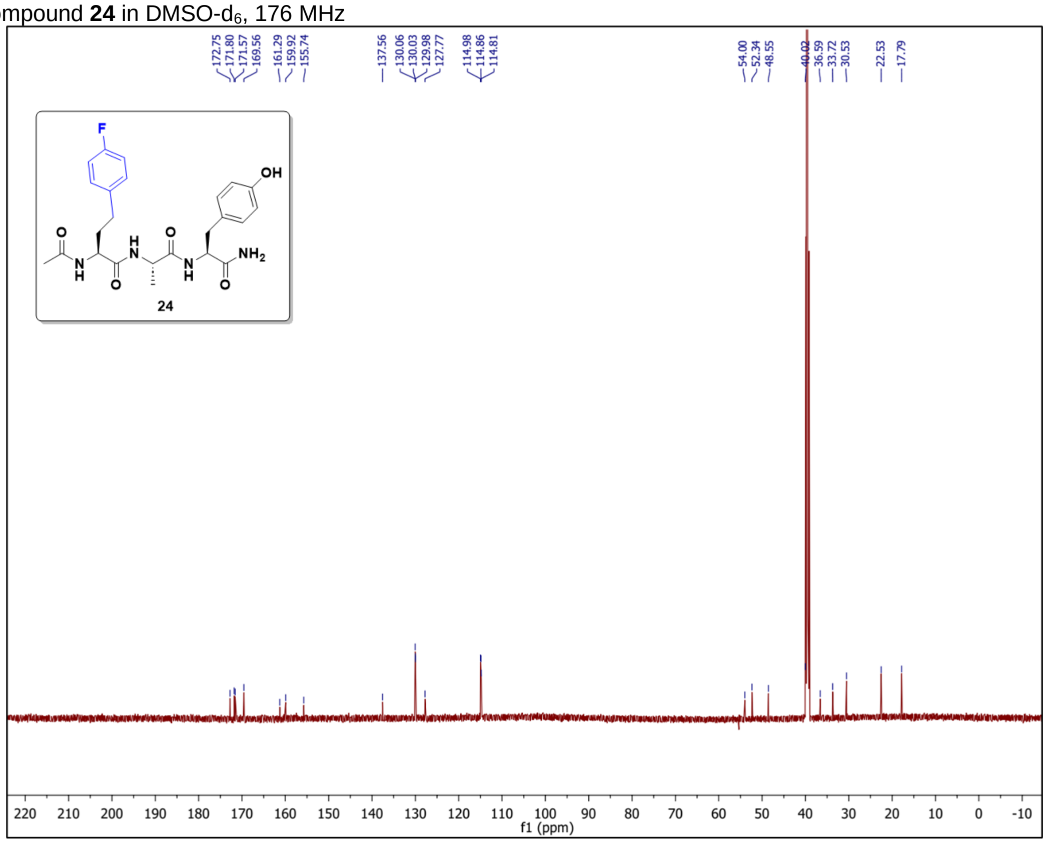

^{19}F NMR of compound **24** in DMSO-d_6, 470 MHz (* corresponds to residual trifluoroacetic acid)

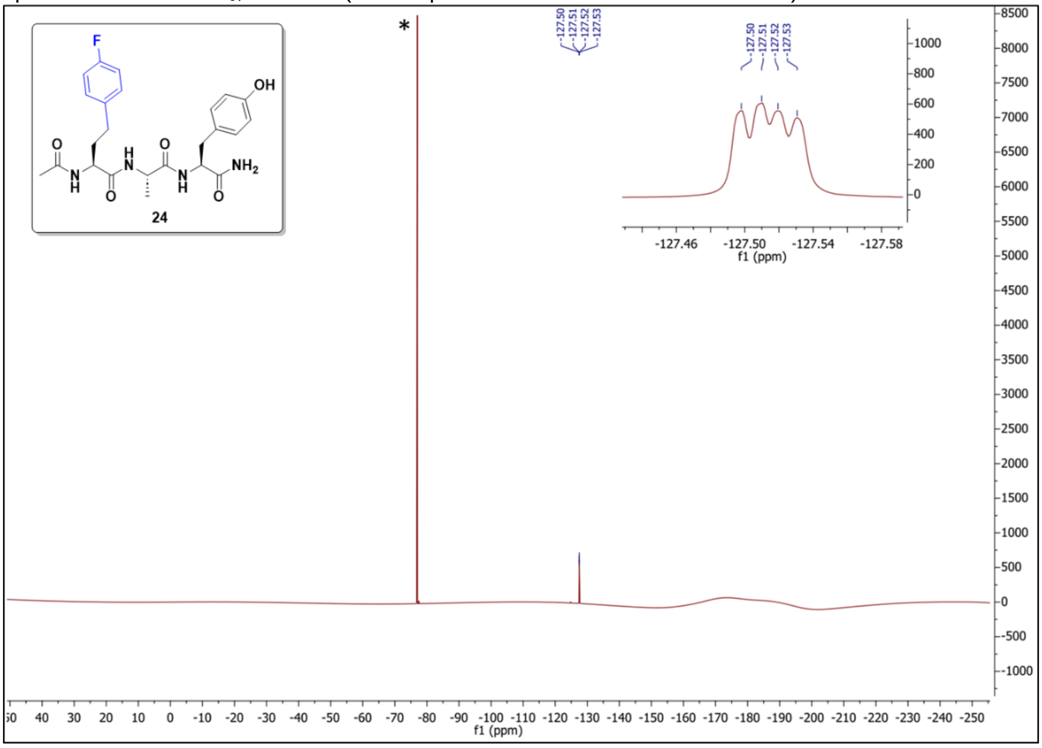

^{1}H NMR of compound **25** in DMSO-d_6, 700 MHz

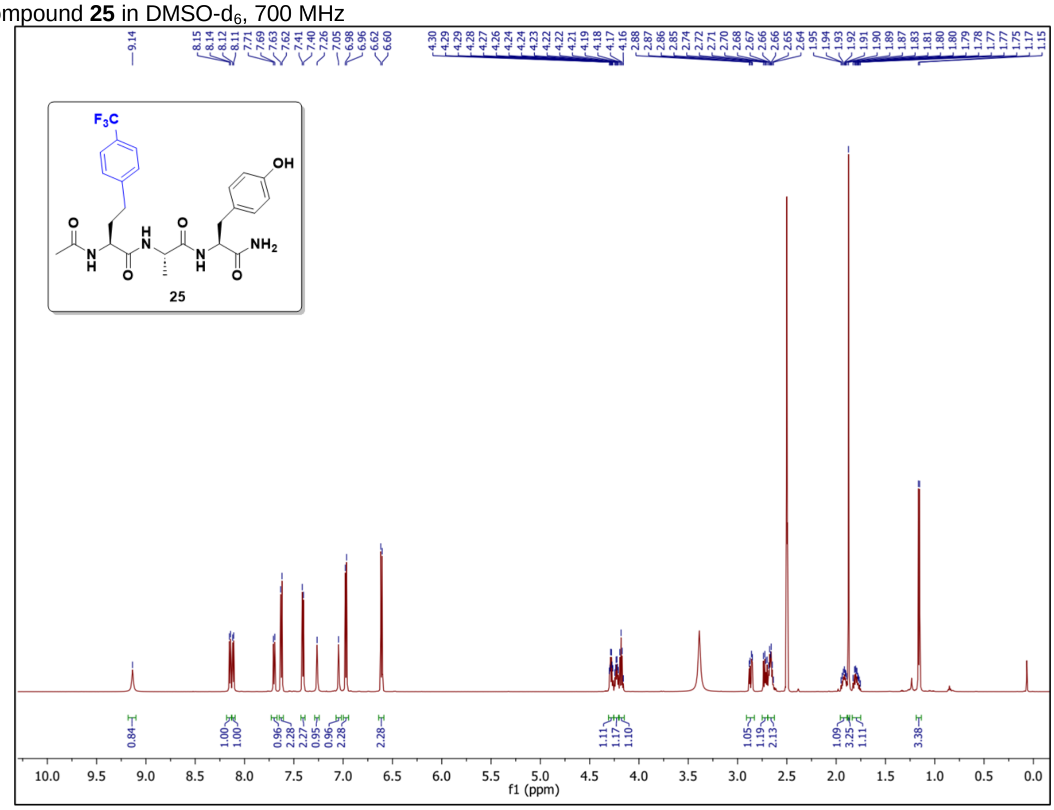

^{13}C NMR of compound **25** in DMSO-d_6, 176 MHz

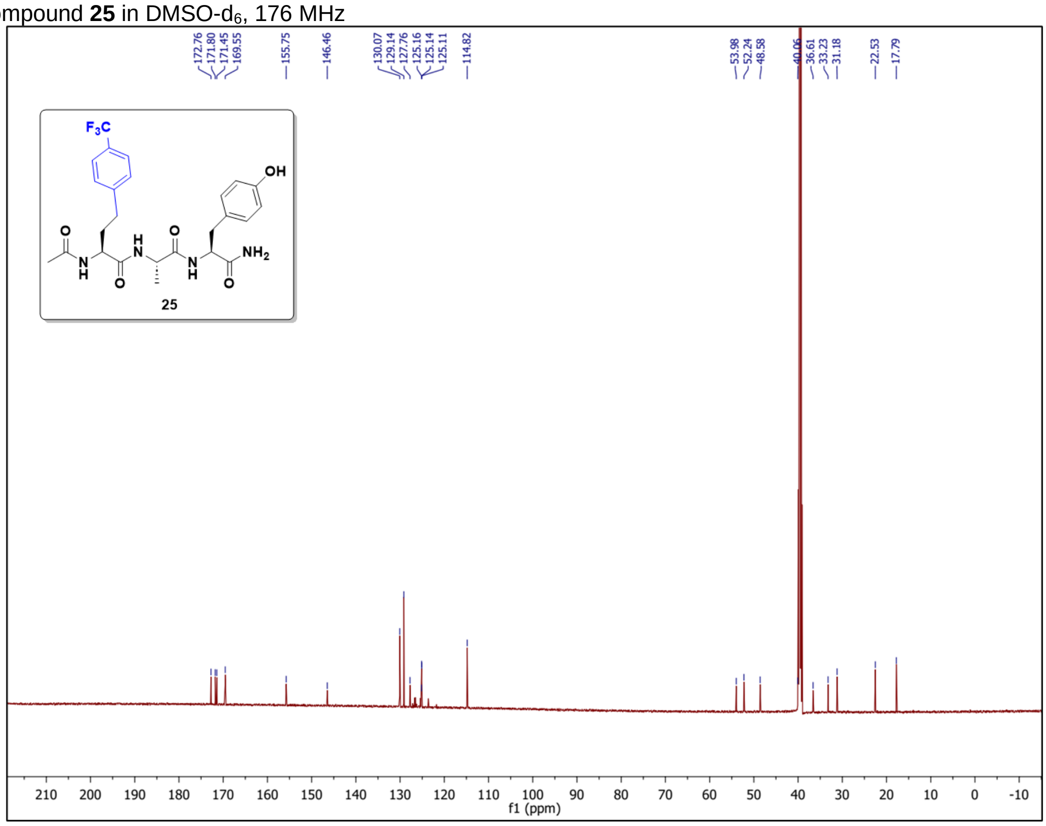

^{19}F NMR of compound **25** in DMSO-d_6, 470 MHz (* corresponds to residual trifluoroacetic acid)

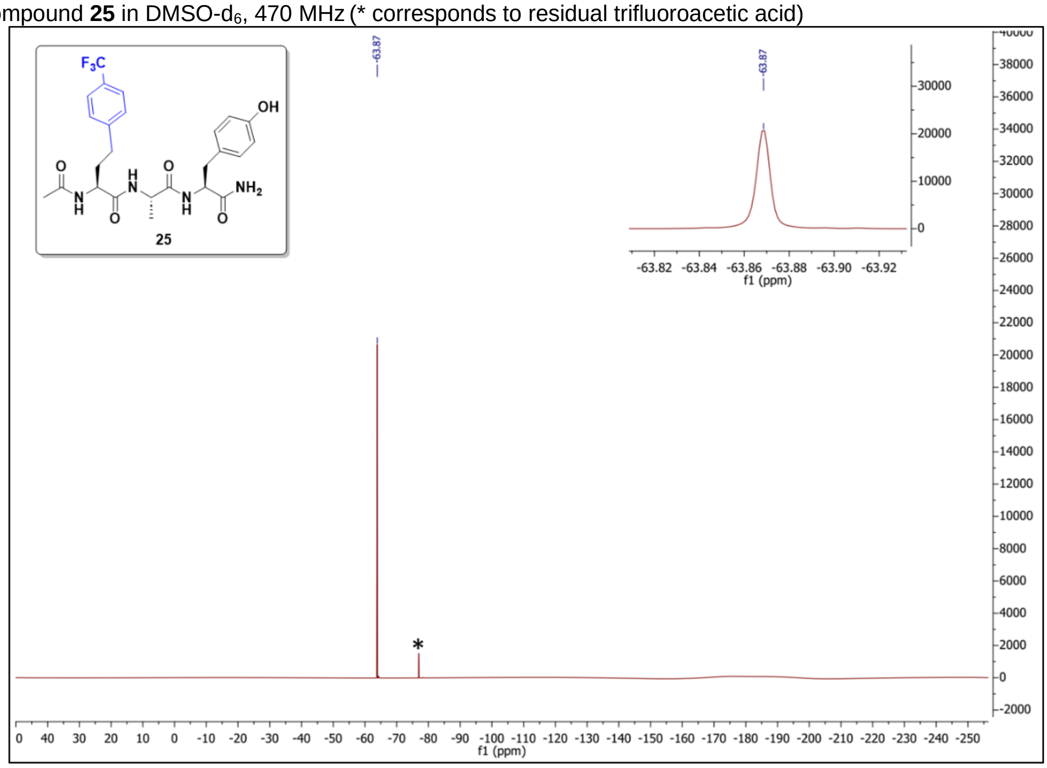

^{1}H NMR of compound **26** in DMSO-d$_6$, 700 MHz

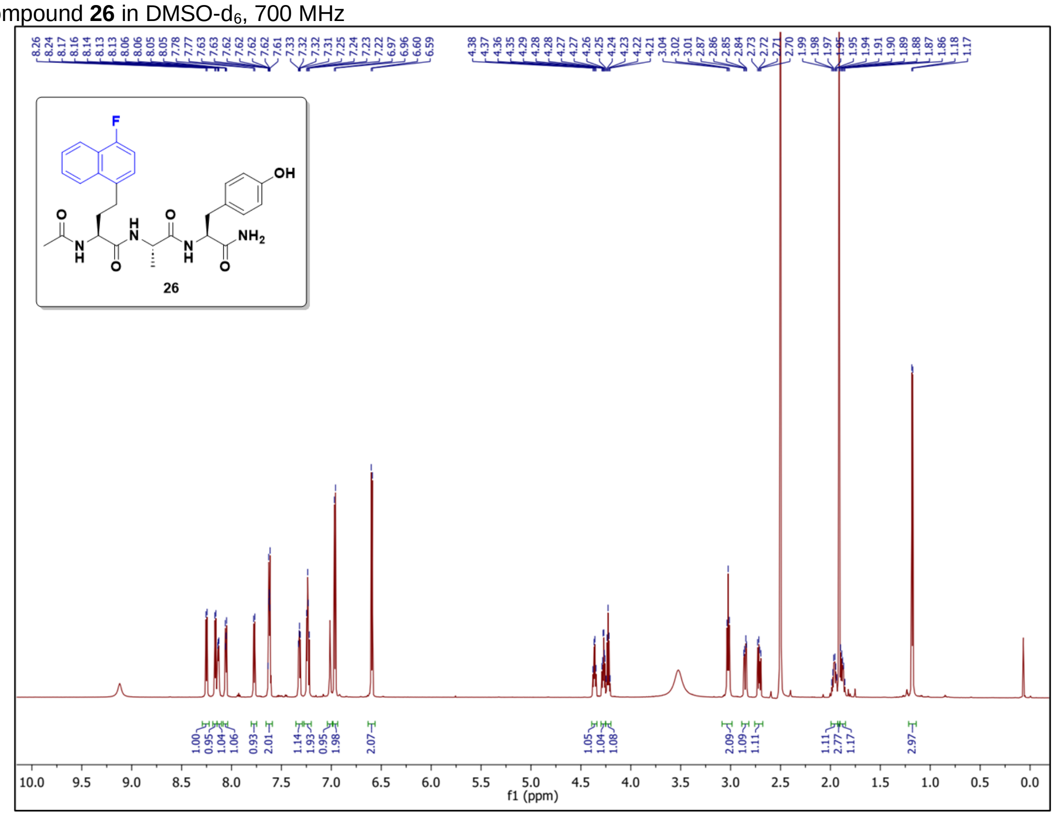

^{13}C NMR of compound **26** in DMSO-d$_6$, 176 MHz

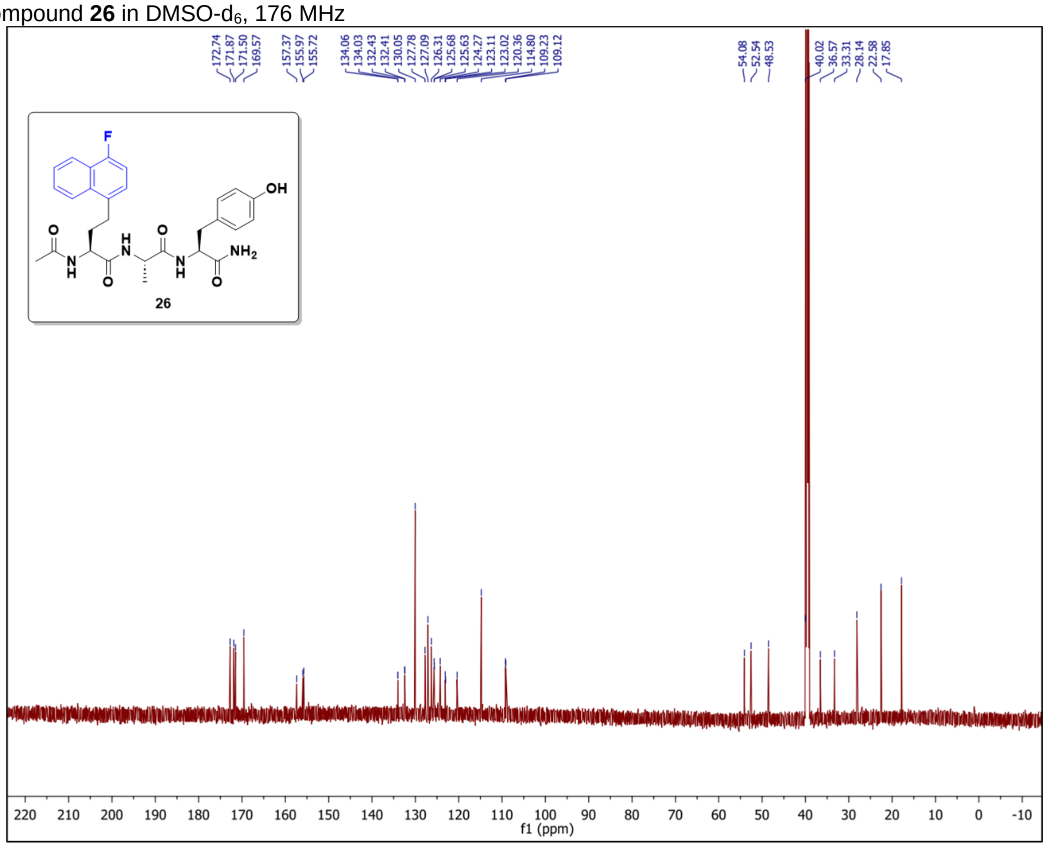

^{19}F NMR of compound **26** in DMSO-d_6, 470 MHz (* corresponds to residual trifluoroacetic acid)

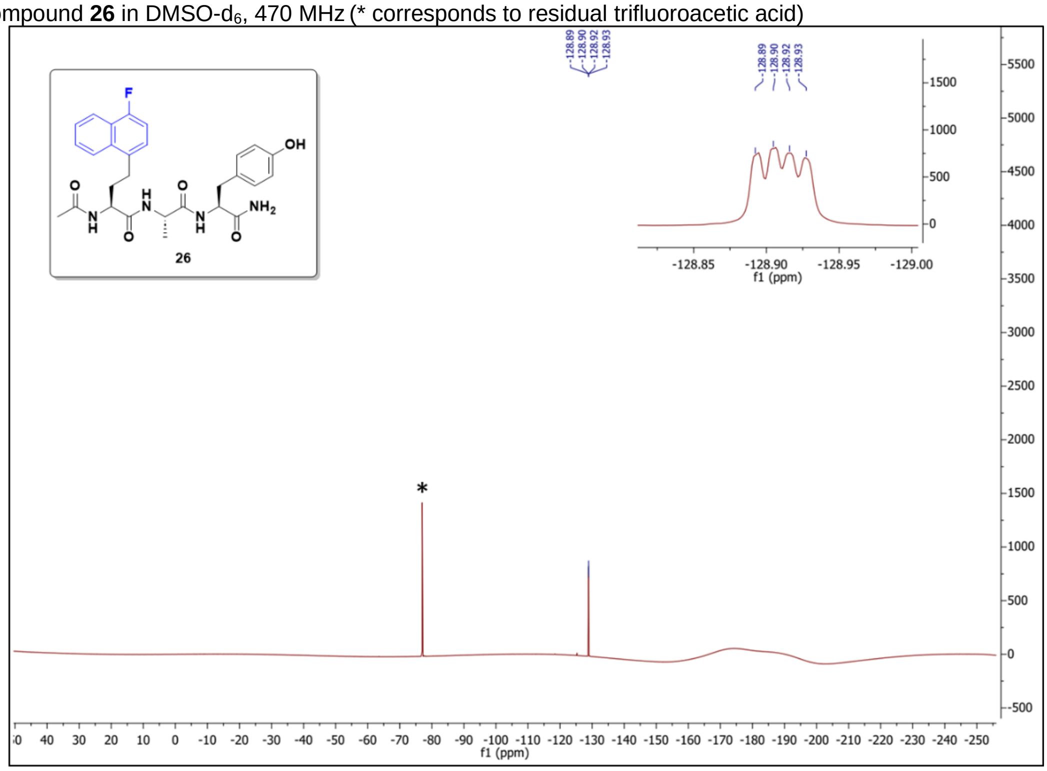

^{1}H NMR of compound **27** in DMSO-d_6, 700 MHz

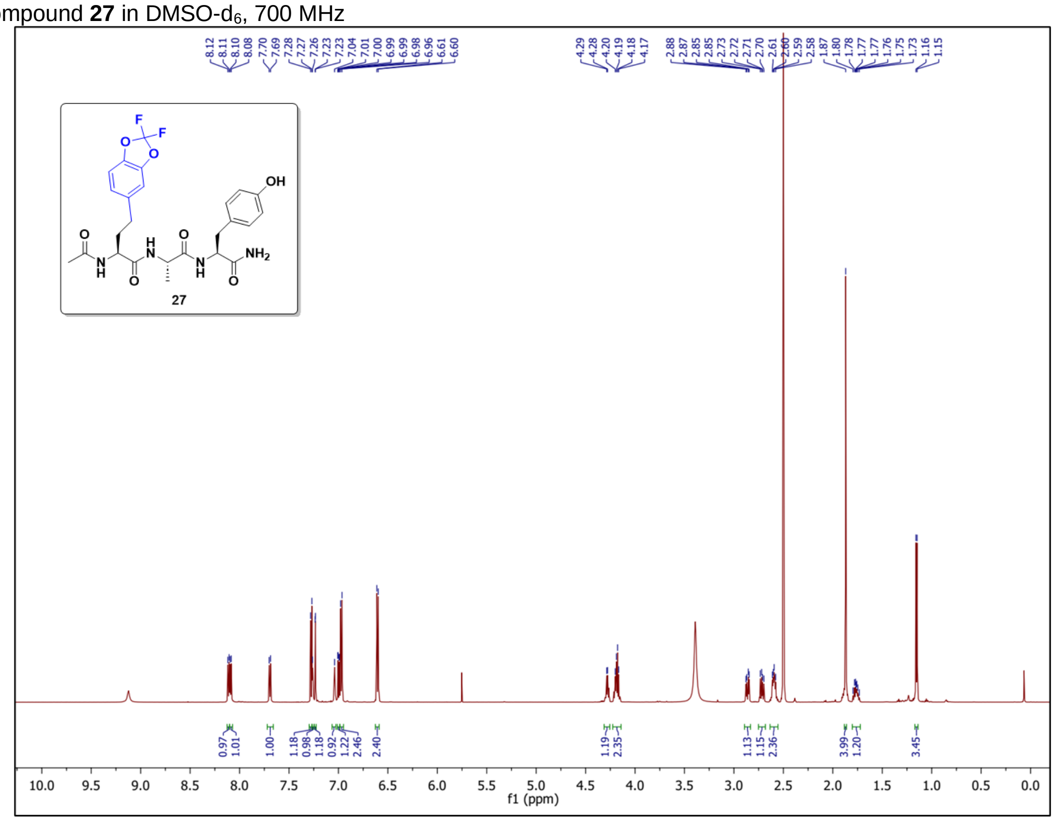

^{13}C NMR of compound **27** in DMSO-d_6, 176 MHz

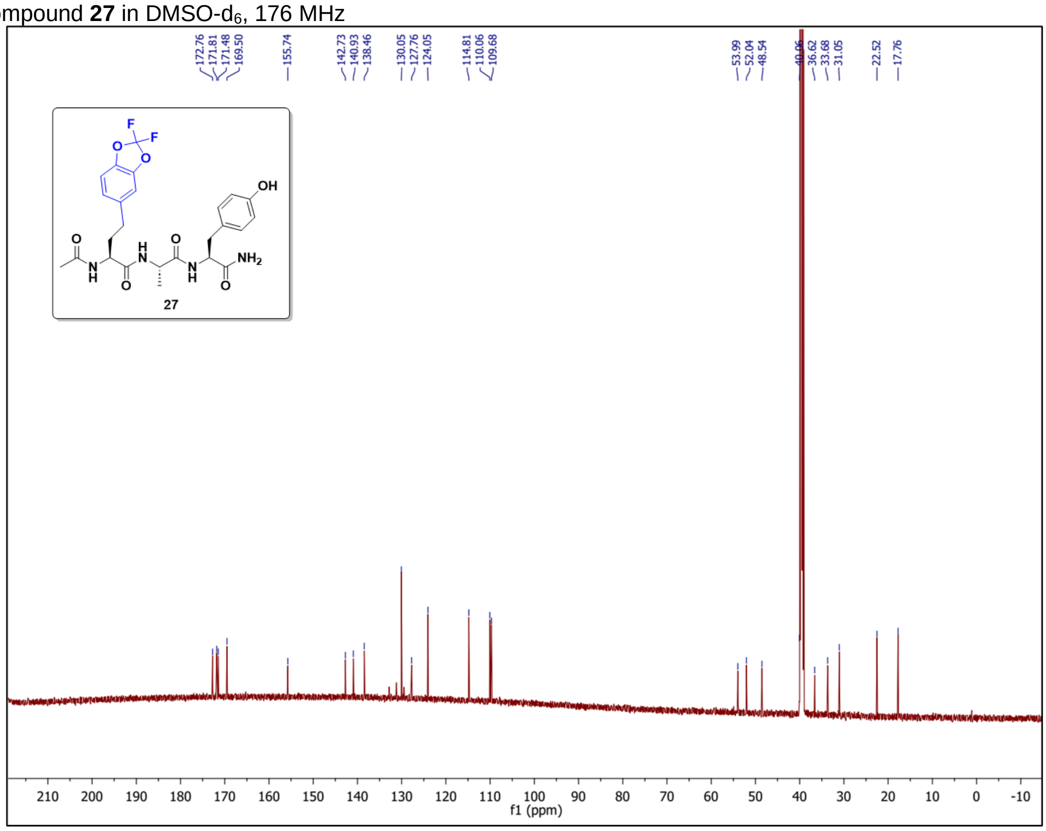

^{19}F NMR of compound **27** in DMSO-d_6, 470 MHz (* corresponds to residual trifluoroacetic acid)

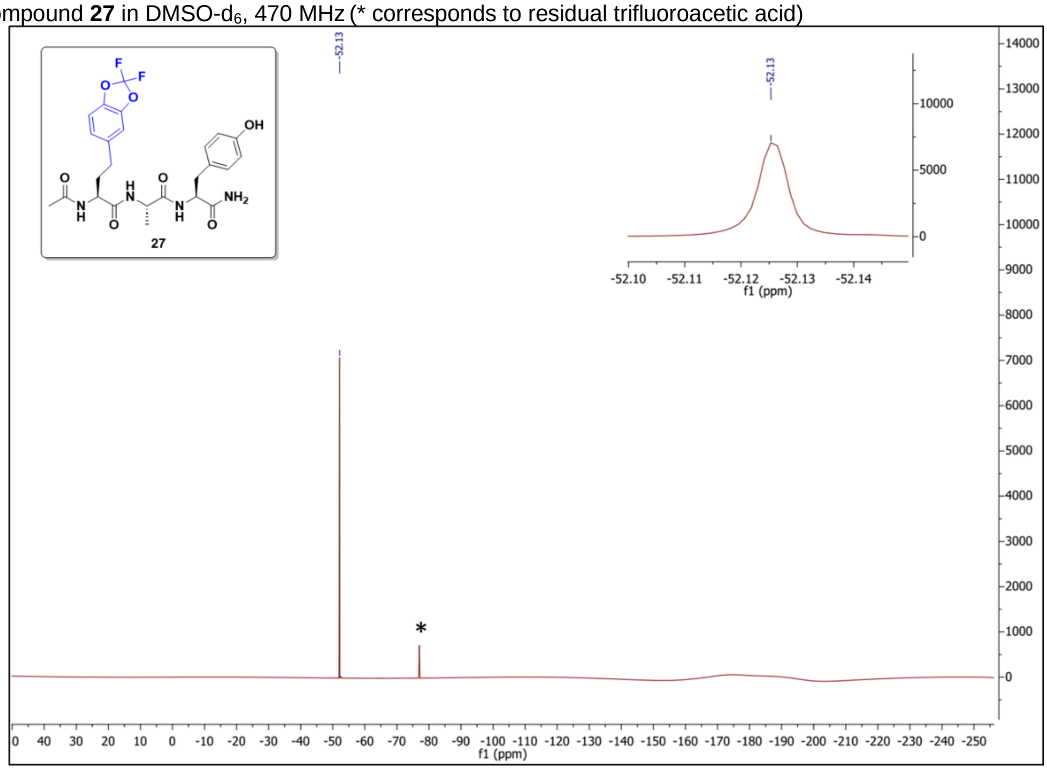

^{1}H NMR of compound **28** in DMSO-d_6, 700 MHz

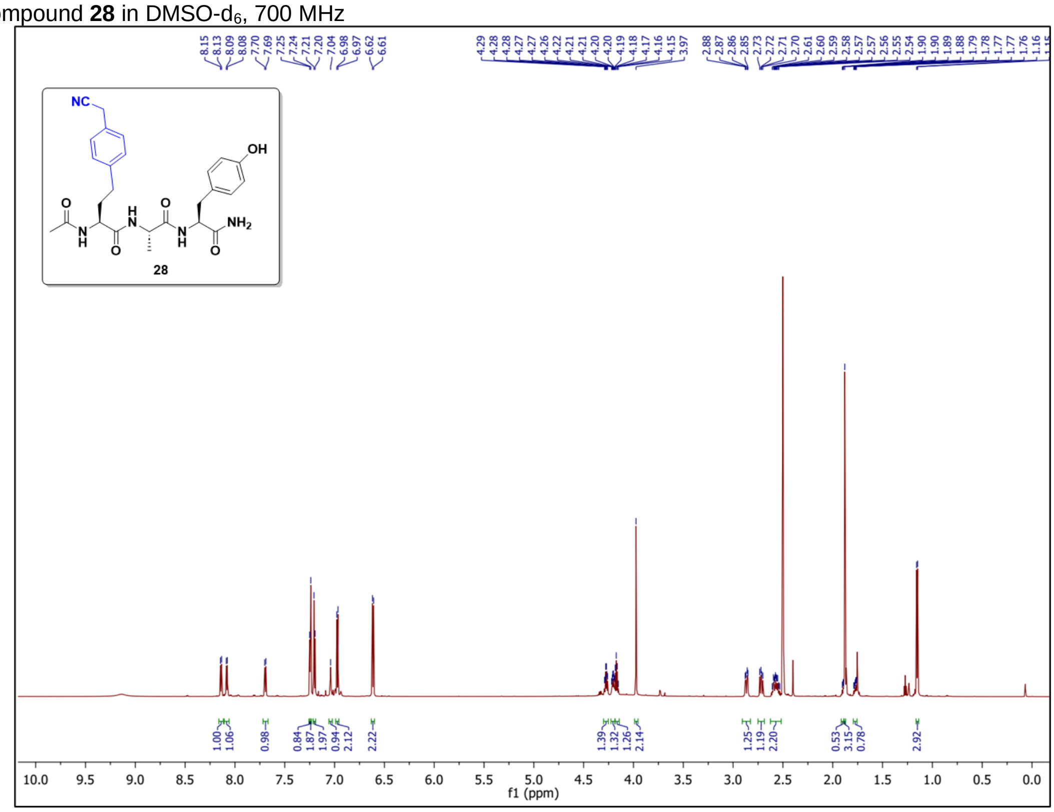

^{13}C NMR of compound **28** in DMSO-d_6, 176 MHz

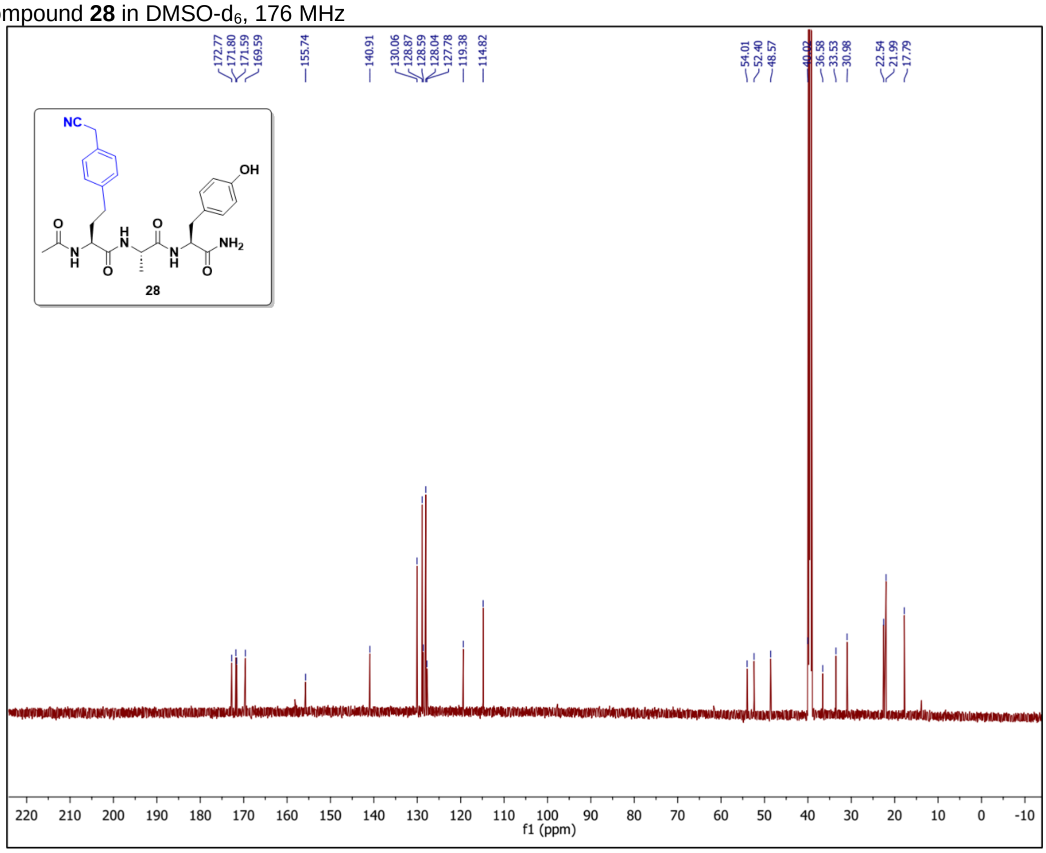

^{1}H NMR of compound **29** in DMSO-d$_6$, 700 MHz

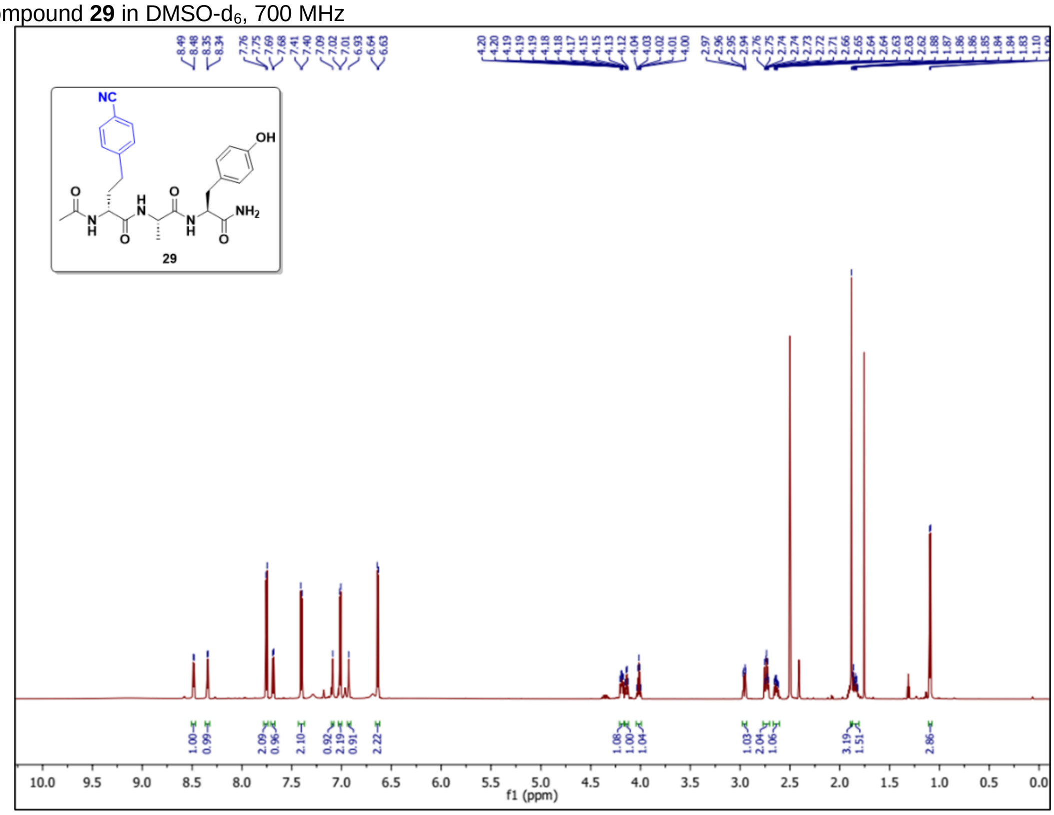

^{13}C NMR of compound **29** in DMSO-d$_6$, 176 MHz

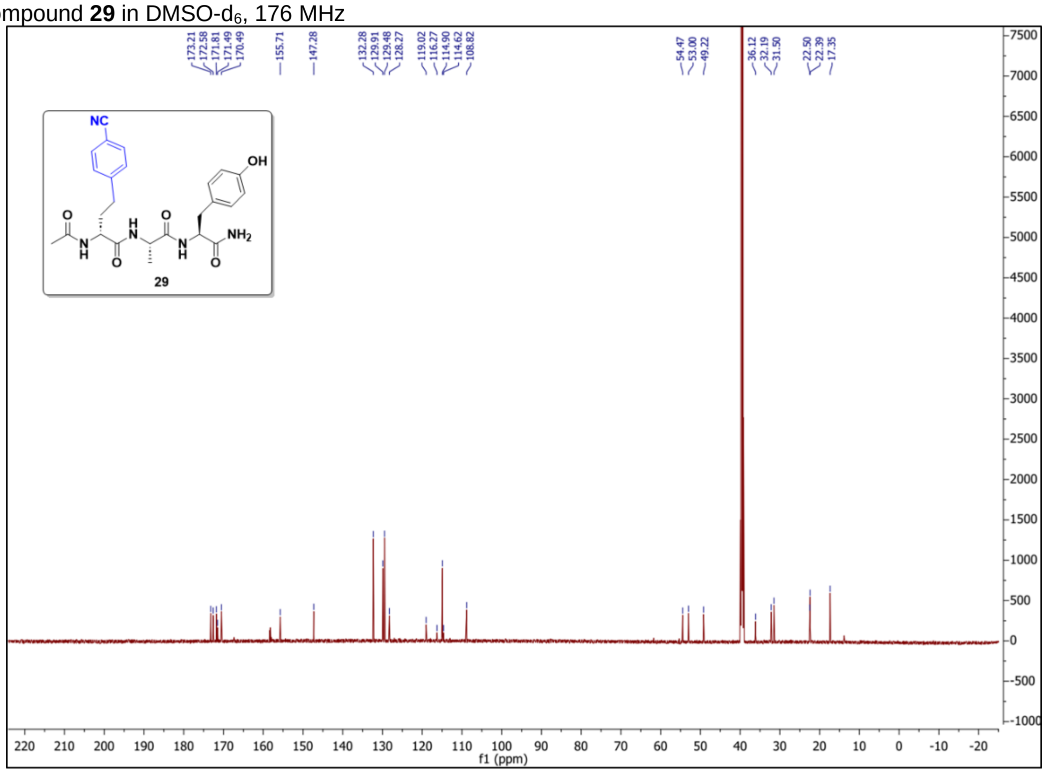

^{1}H NMR of compound **30** in DMSO-d_6, 700 MHz

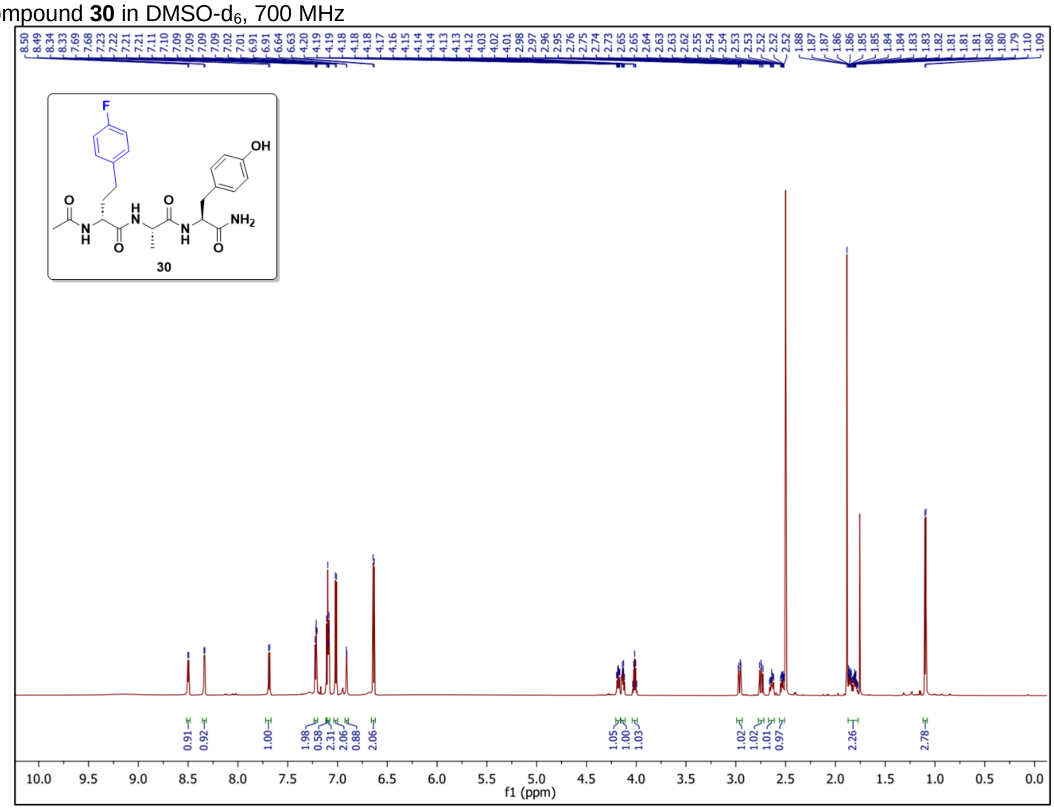

^{13}C NMR of compound **30** in DMSO-d_6, 176 MHz

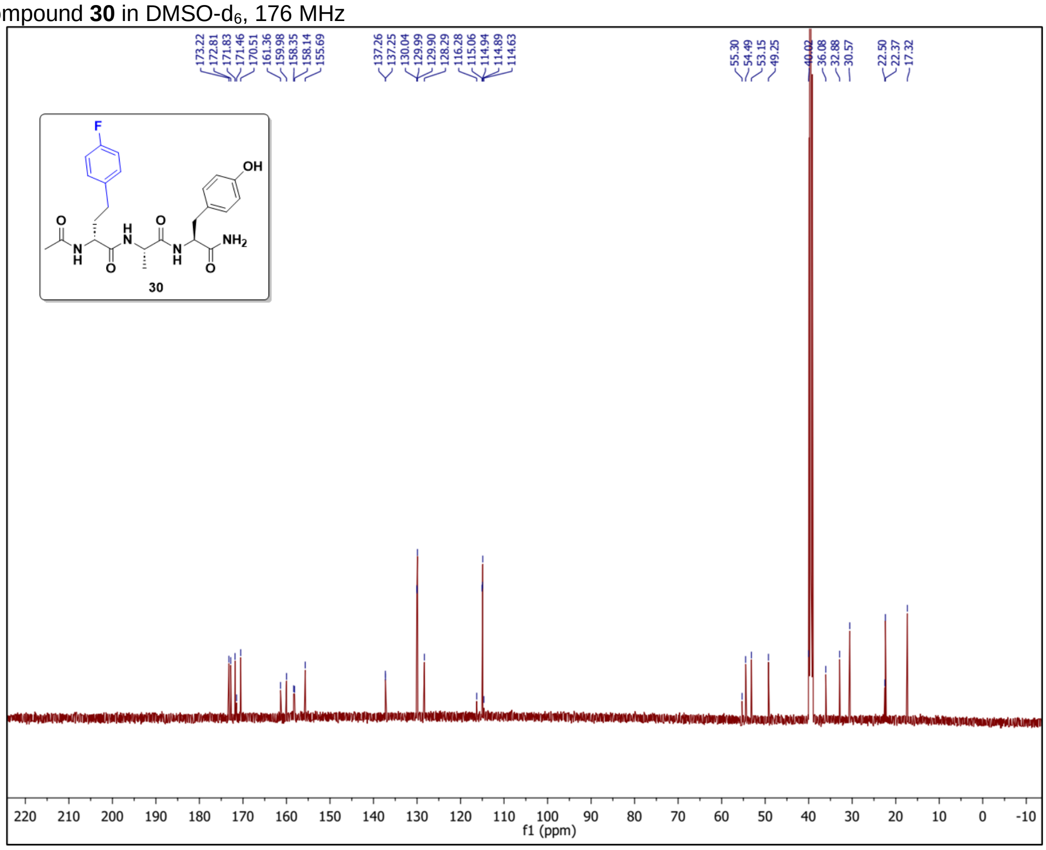

^{19}F NMR of compound **30** in DMSO-d$_6$, 470 MHz (* corresponds to residual trifluoroacetic acid)

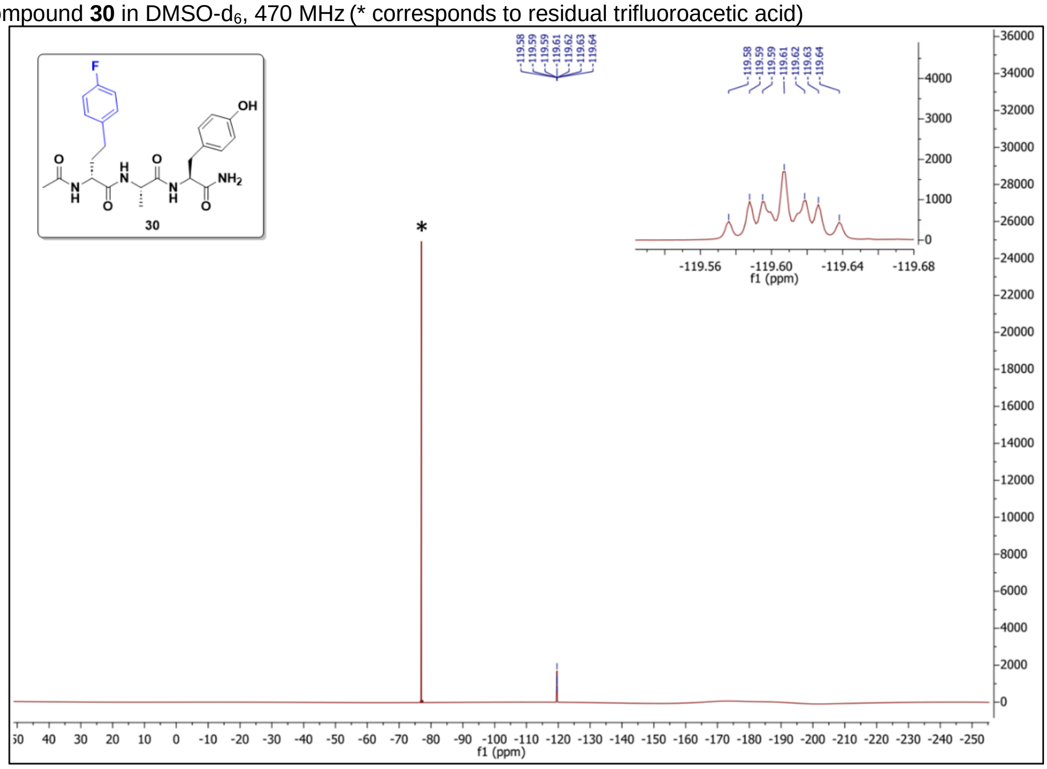

^{1}H NMR of compound **31** in DMSO-d$_6$, 700 MHz

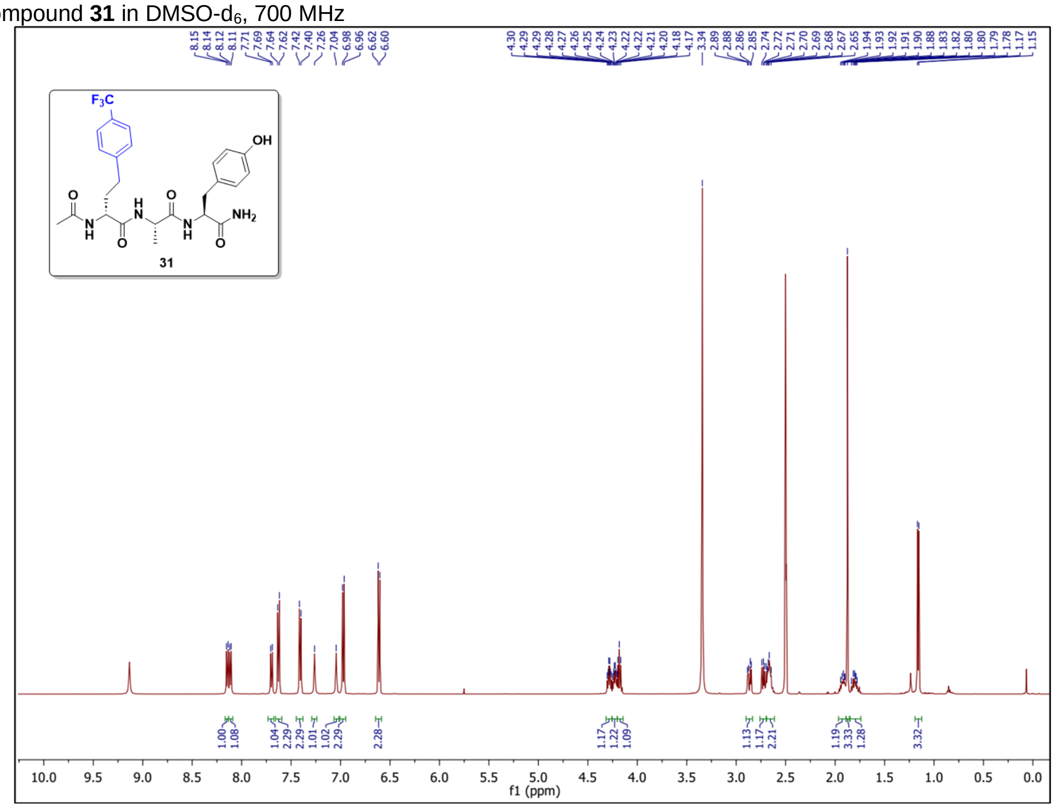

^{13}C NMR of compound **31** in DMSO-d$_6$, 176 MHz

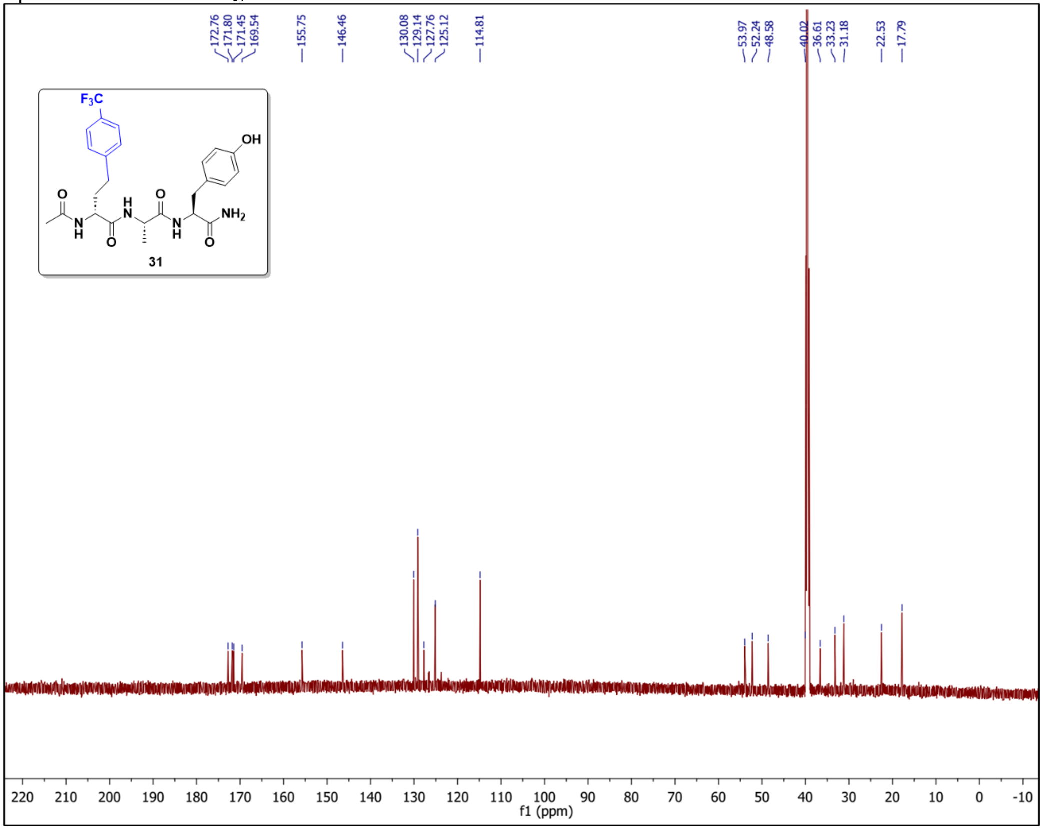

^{19}F NMR of compound **31** in DMSO-d$_6$, 470 MHz (* corresponds to residual trifluoroacetic acid)

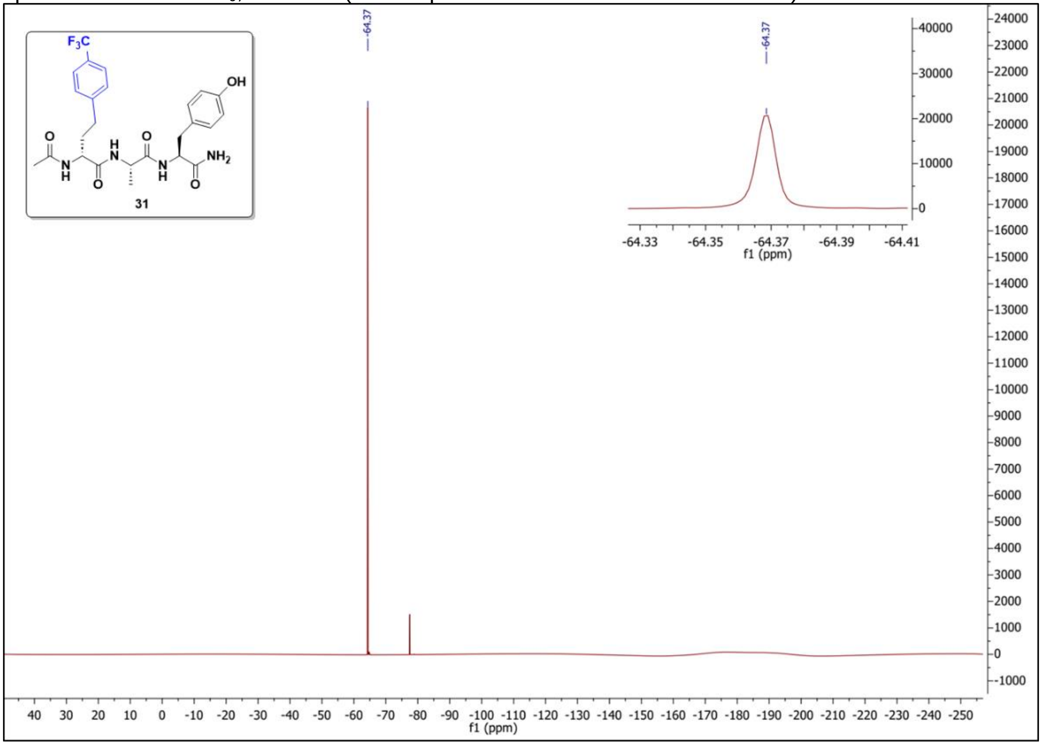

^{1}H NMR of compound **32** in DMSO-d$_6$, 700 MHz

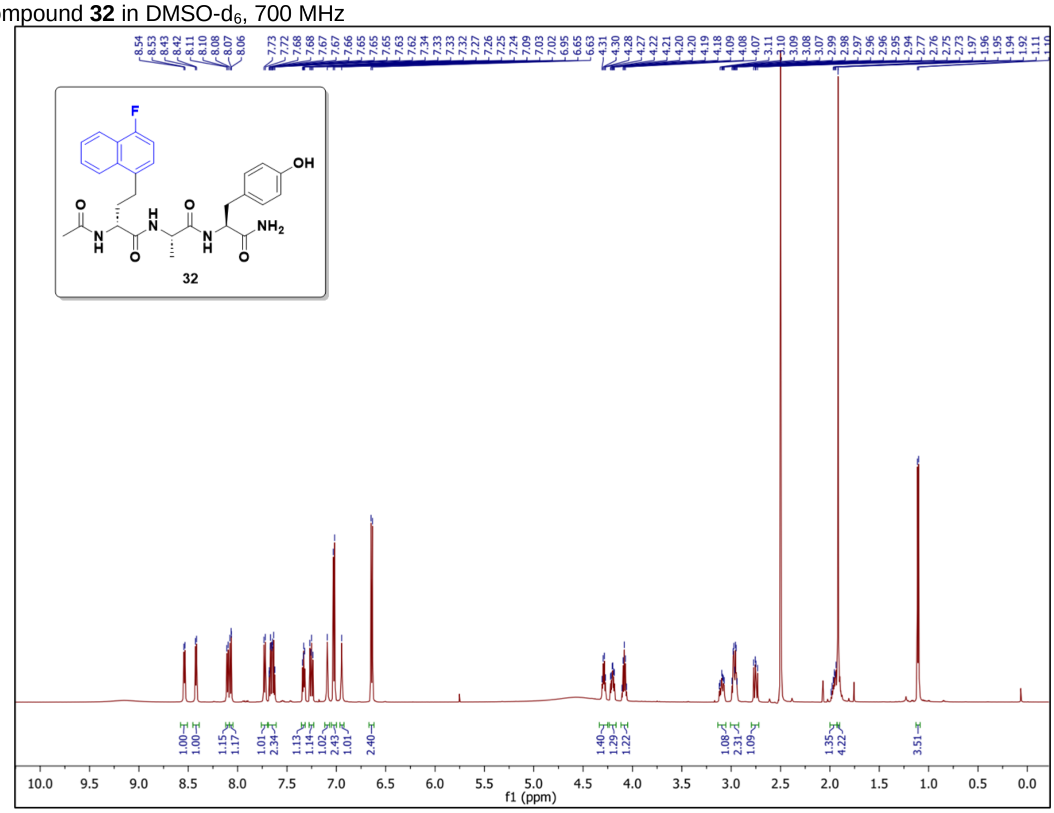

^{13}C NMR of compound **32** in DMSO-d$_6$, 176 MHz

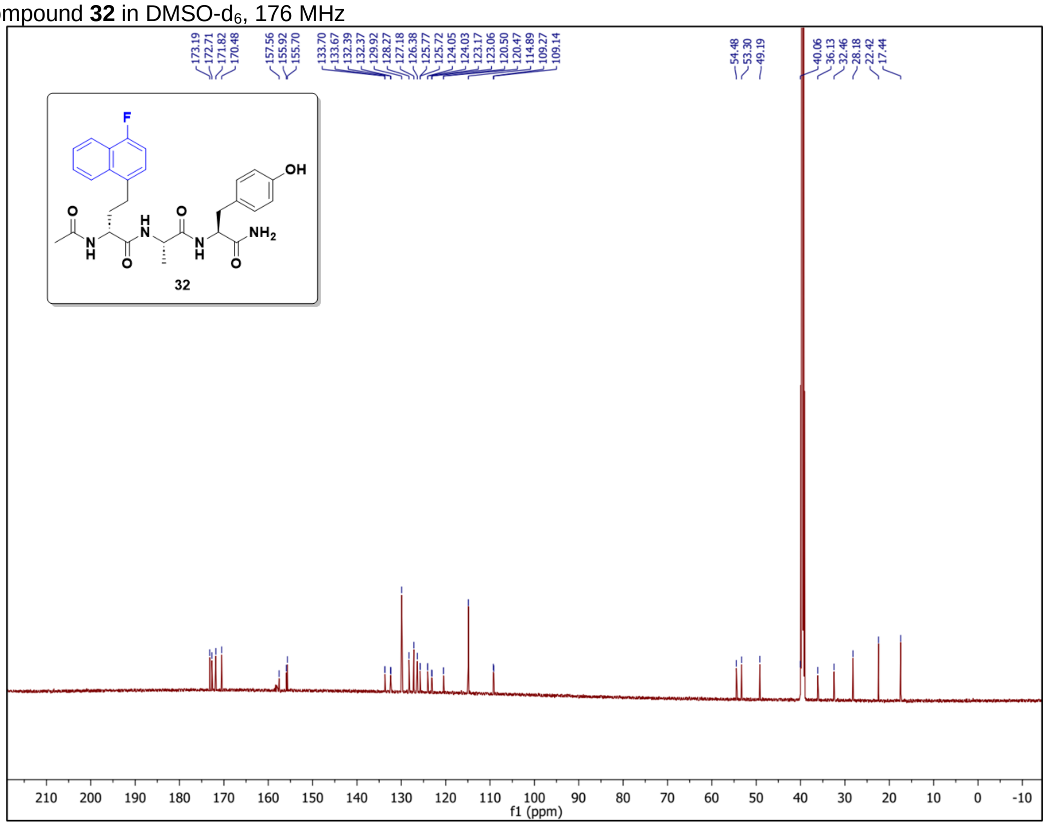

^{19}F NMR of compound **32** in DMSO-d$_6$, 470 MHz (* corresponds to residual trifluoroacetic acid)

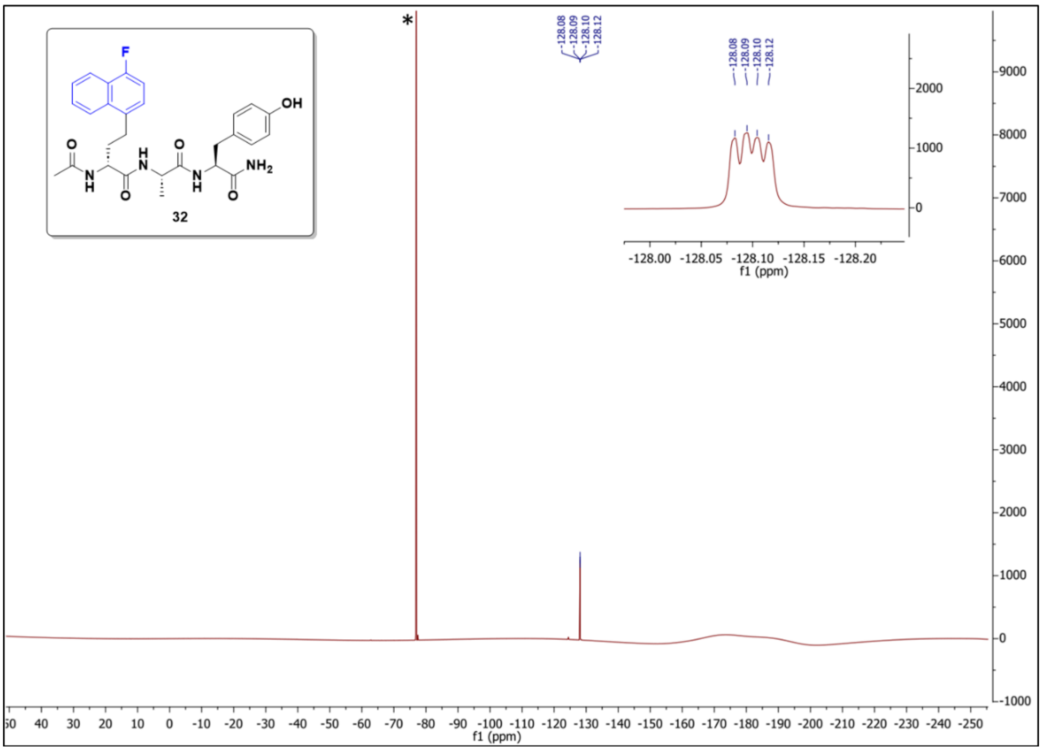

^{1}H NMR of compound **33** in DMSO-d$_6$, 700 MHz

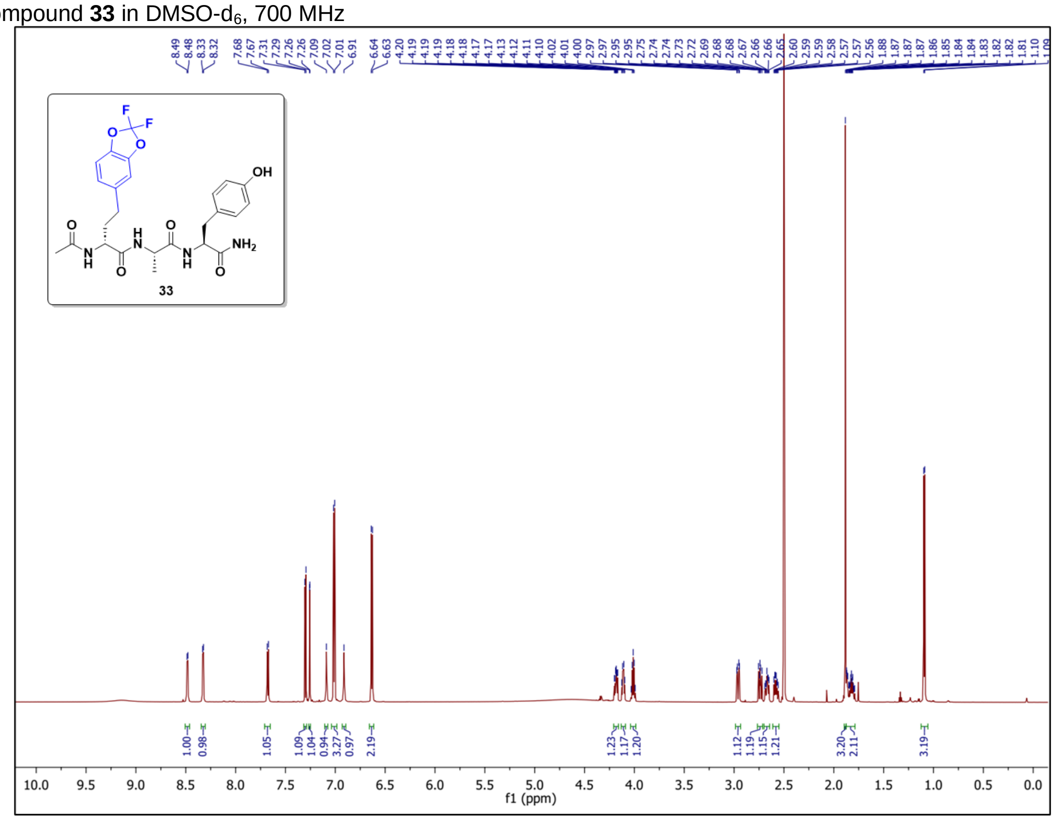

^{13}C NMR of compound **33** in DMSO-d_6, 176 MHz

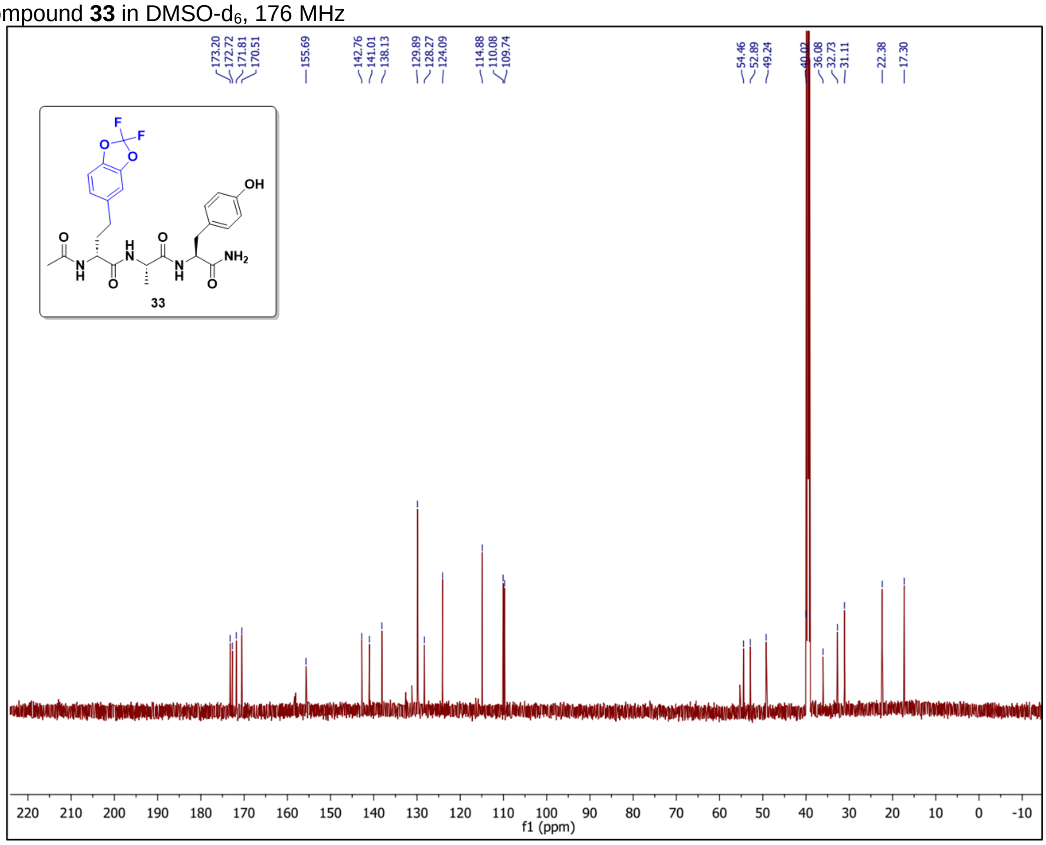

^{19}F NMR of compound **33** in DMSO-d_6, 470 MHz (* corresponds to residual trifluoroacetic acid)

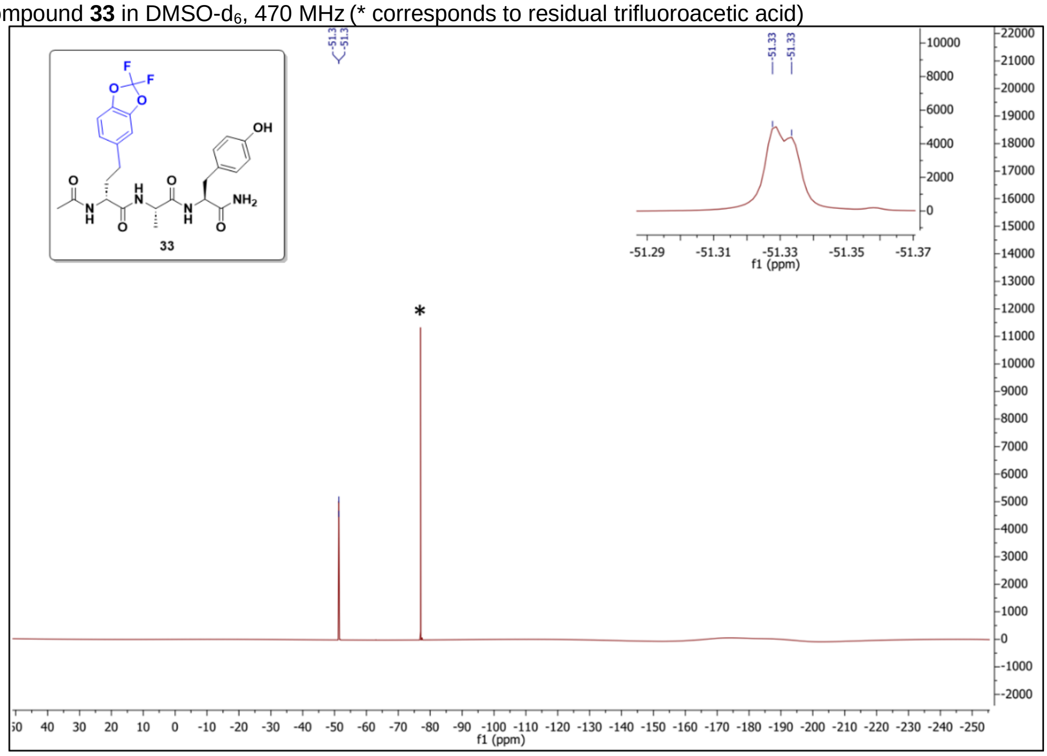

^{1}H NMR of compound **34** in DMSO-d$_6$, 700 MHz

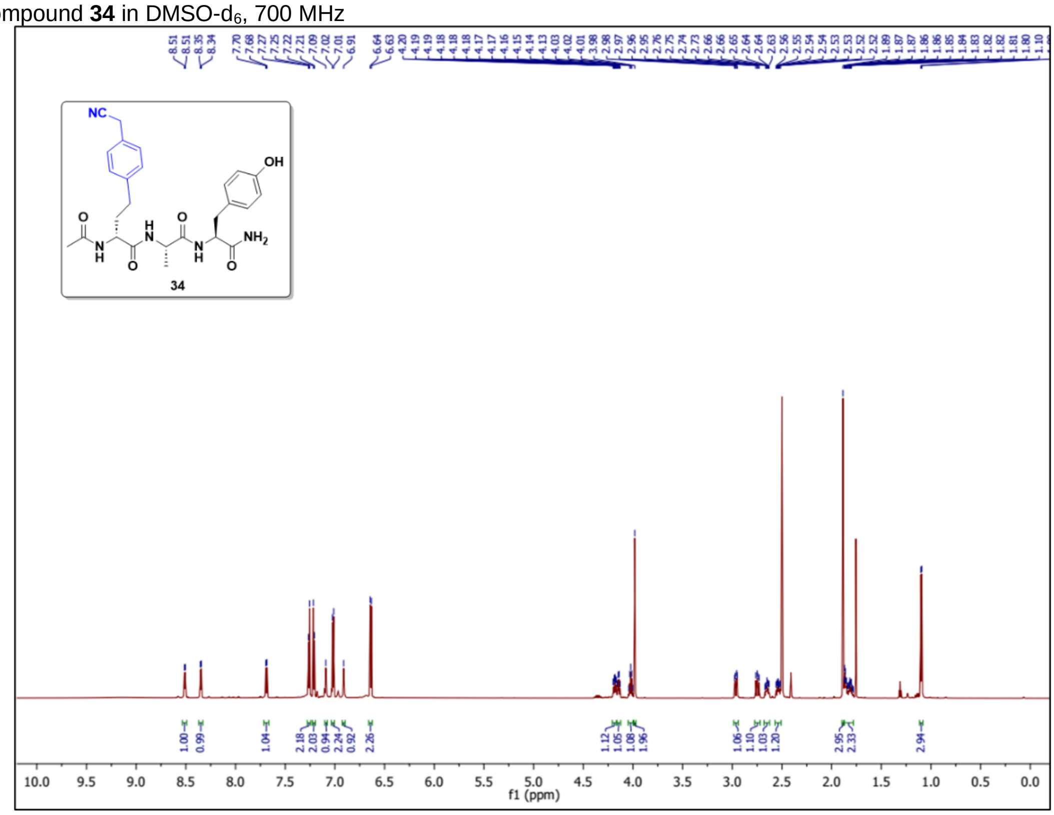

^{13}C NMR of compound **34** in DMSO-d$_6$, 176 MHz

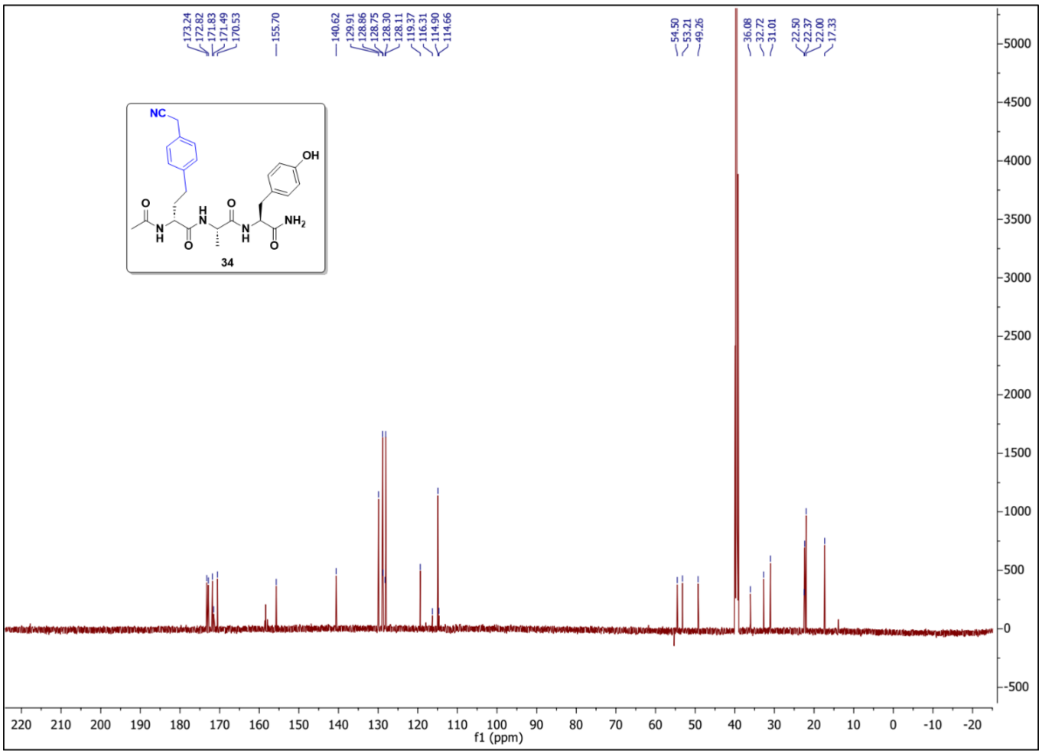

^{1}H NMR of compound **35** in DMSO-d_6, 700 MHz

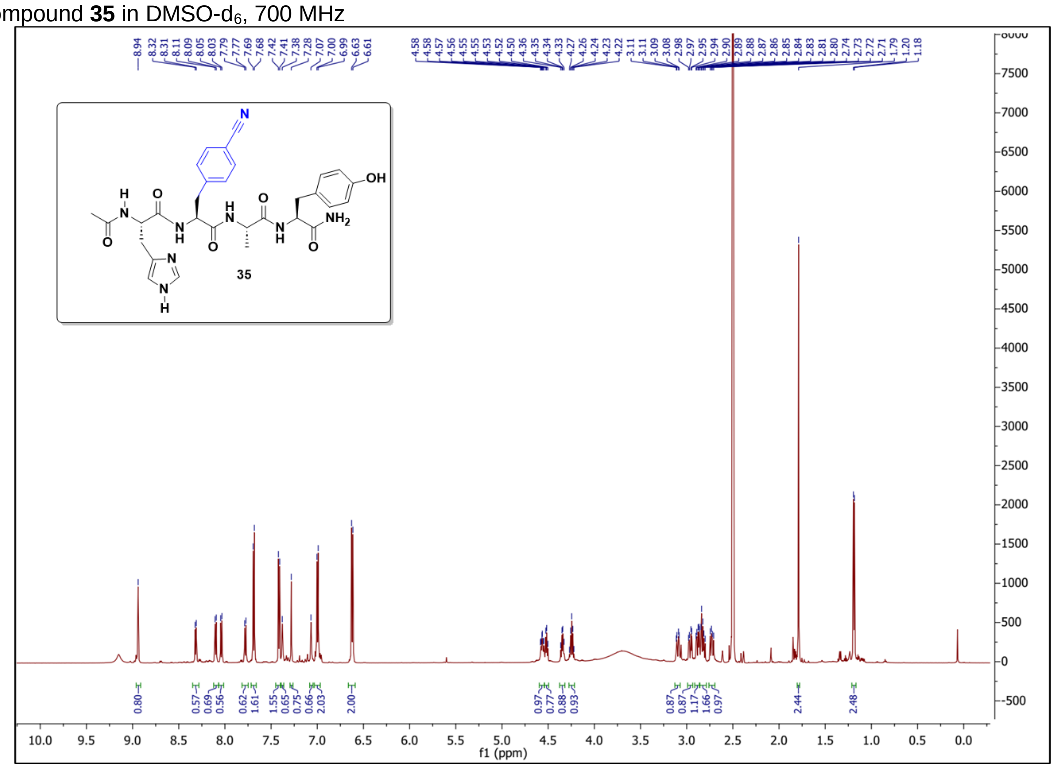

^{13}C NMR of compound **35** in DMSO-d_6, 176 MHz

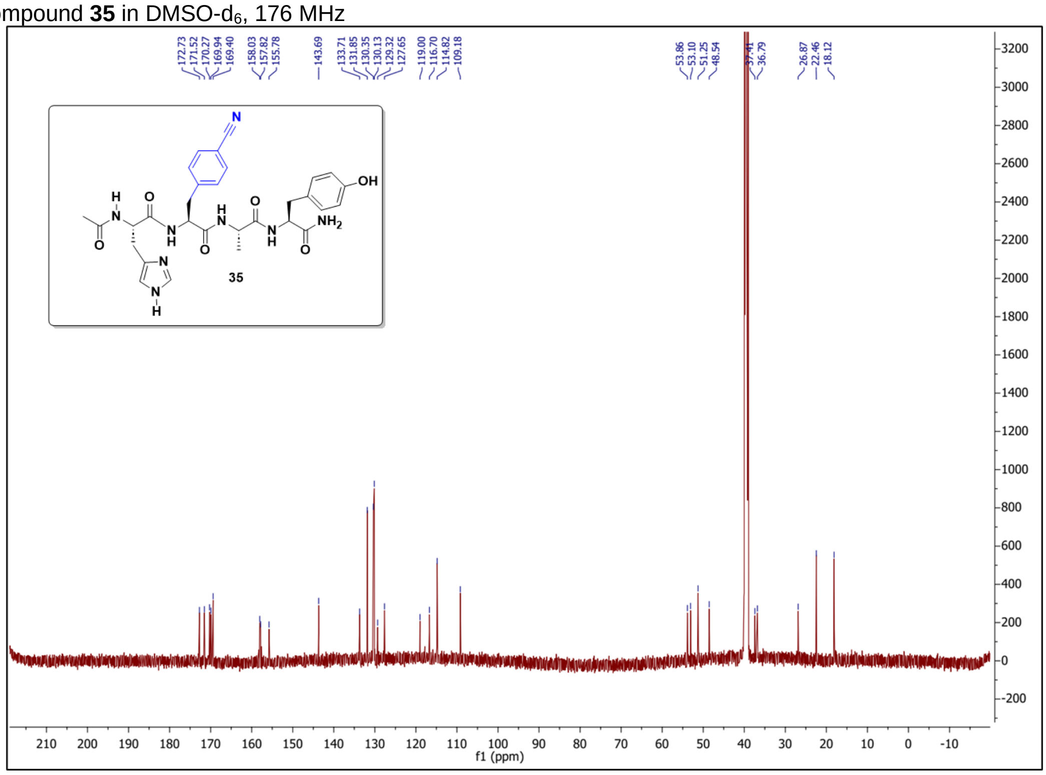

^{1}H NMR of compound **36** in DMSO-d$_6$, 700 MHz

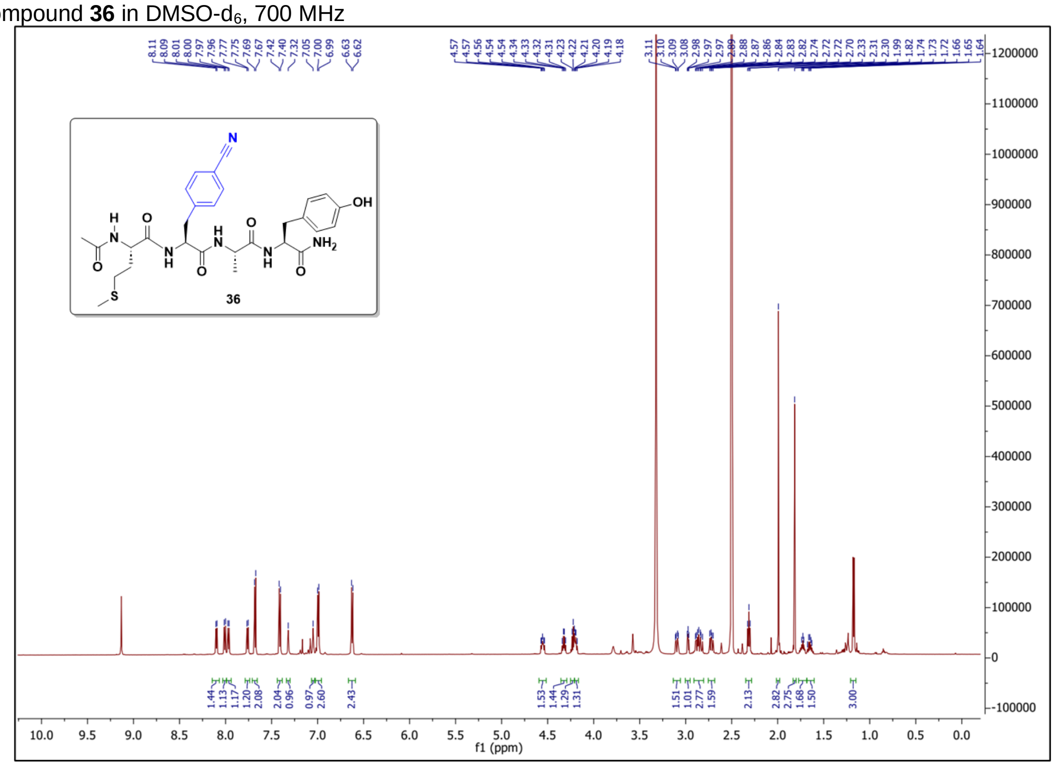

^{13}C NMR of compound **36** in DMSO-d$_6$, 176 MHz

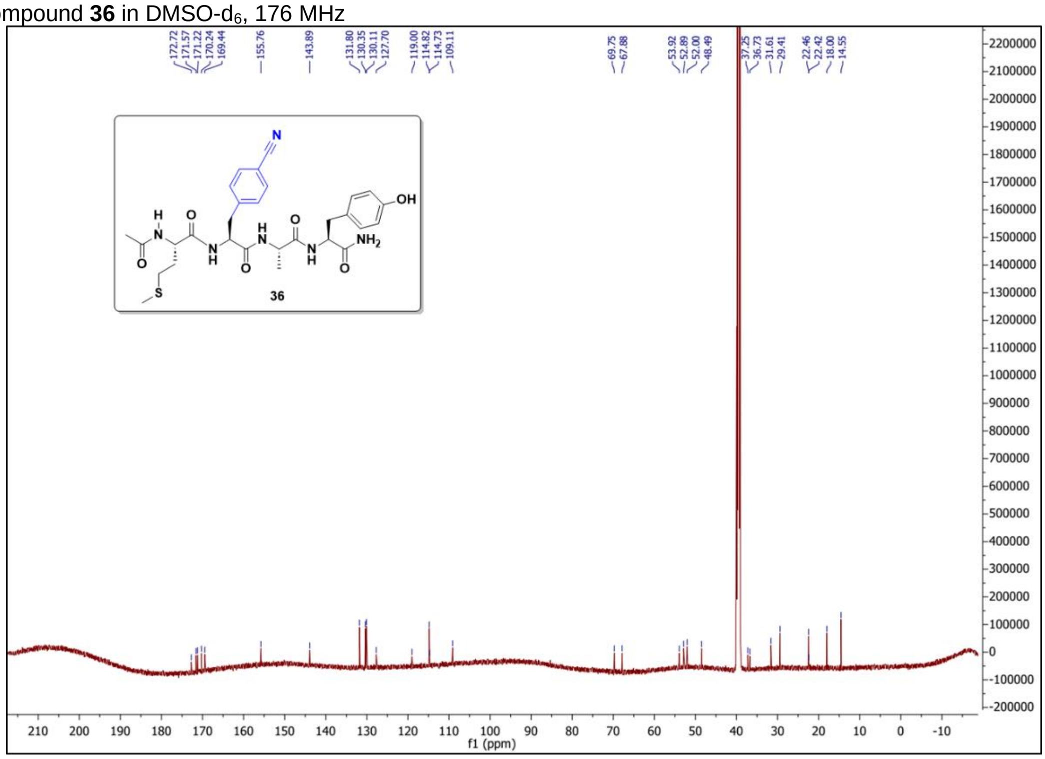

^{1}H NMR of compound **37** in DMSO-d_6, 700 MHz

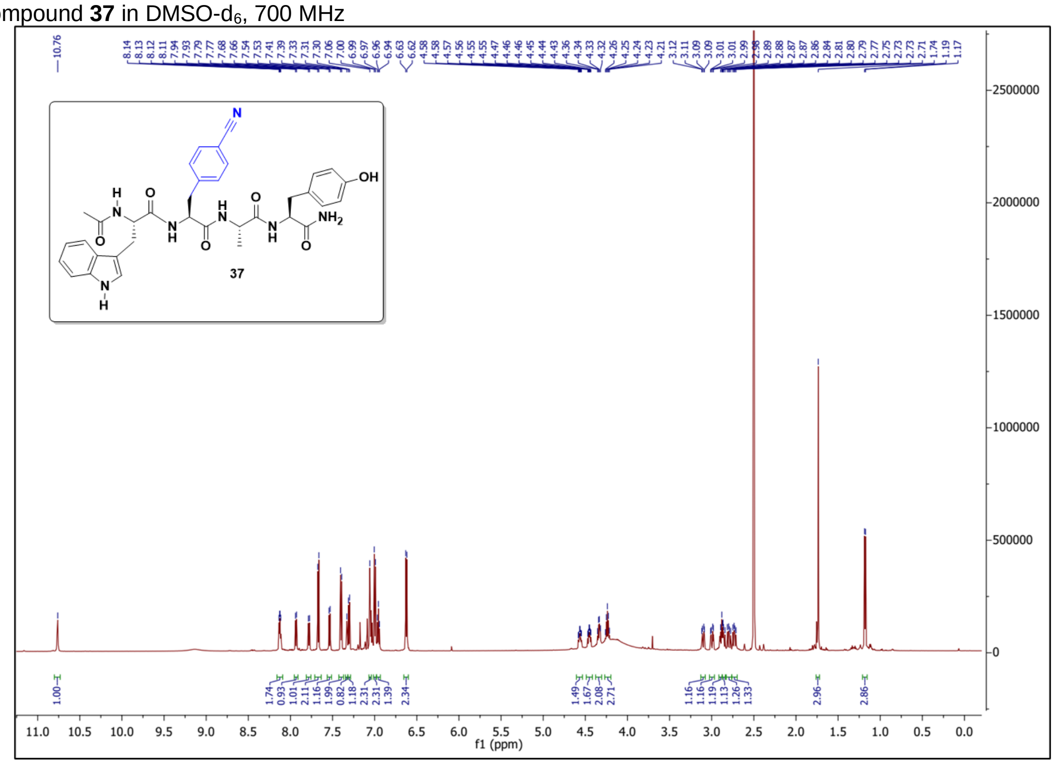

^{13}C NMR of compound **37** in DMSO-d_6, 176 MHz

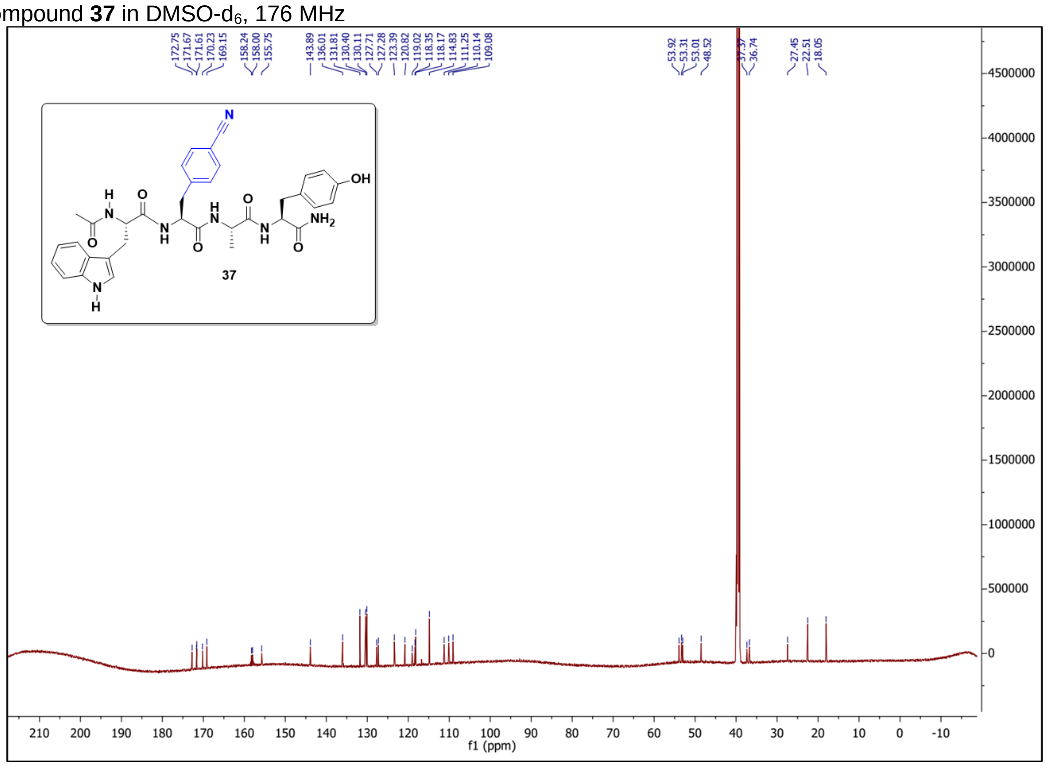

^{1}H NMR of compound **39** in DMSO-d$_6$, 700 MHz

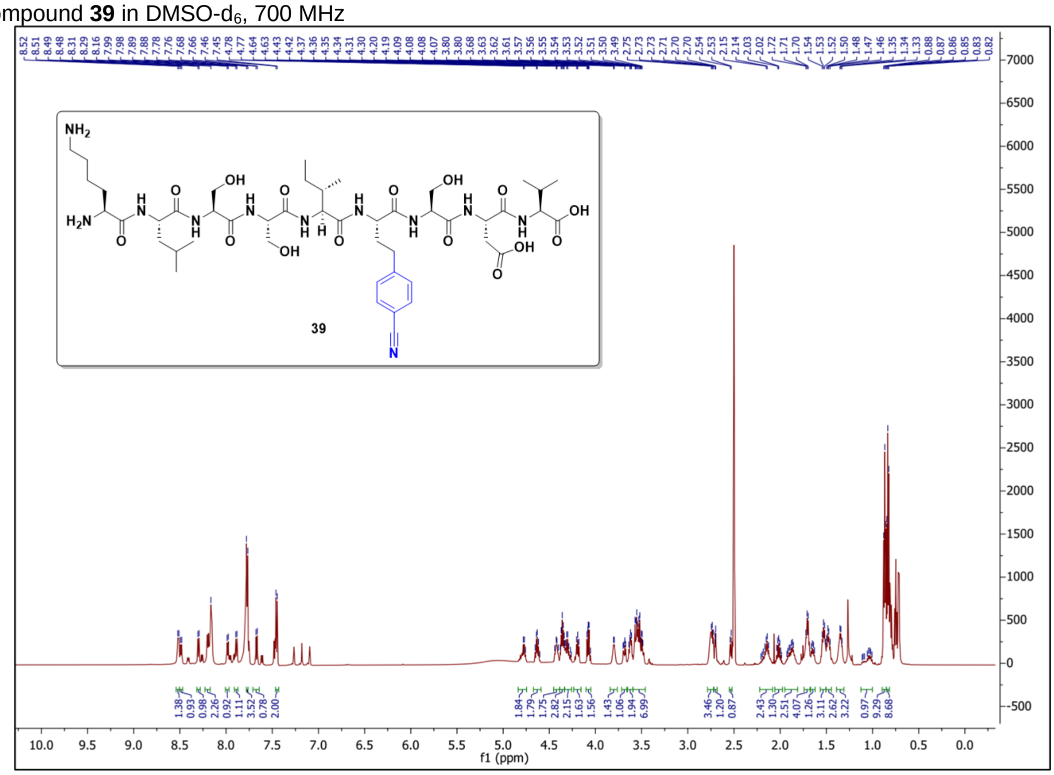

^{13}C NMR of compound **39** in DMSO-d$_6$, 176 MHz

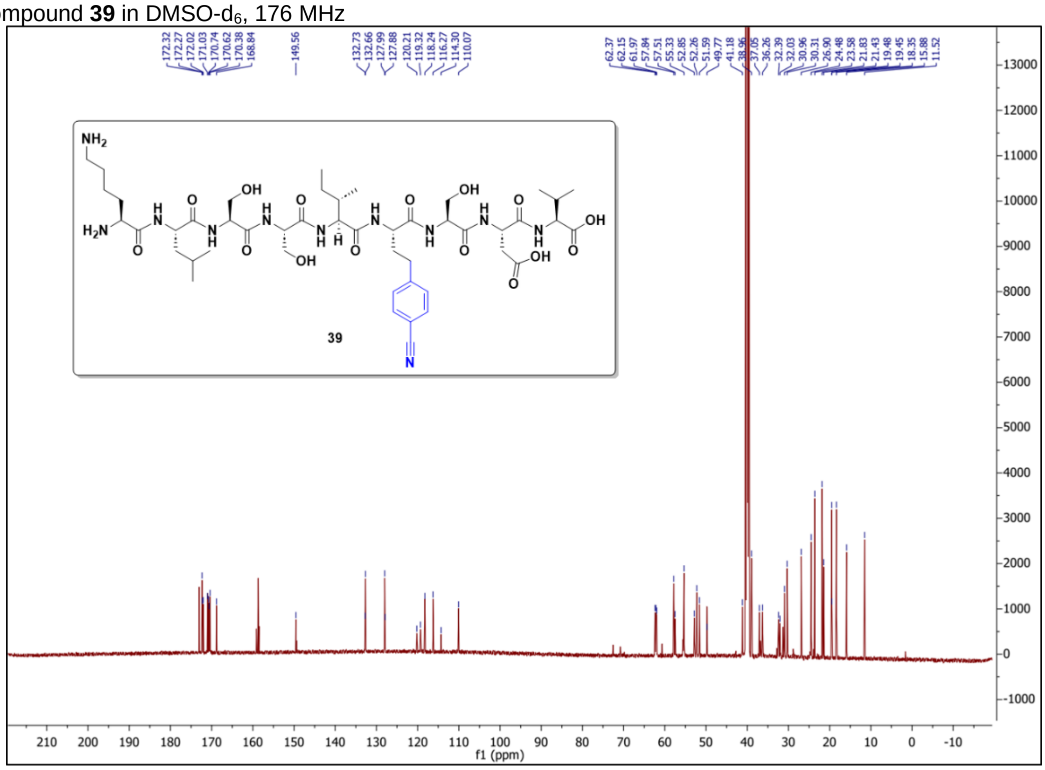

^{1}H NMR of compound **40** in DMSO-d$_6$, 700 MHz

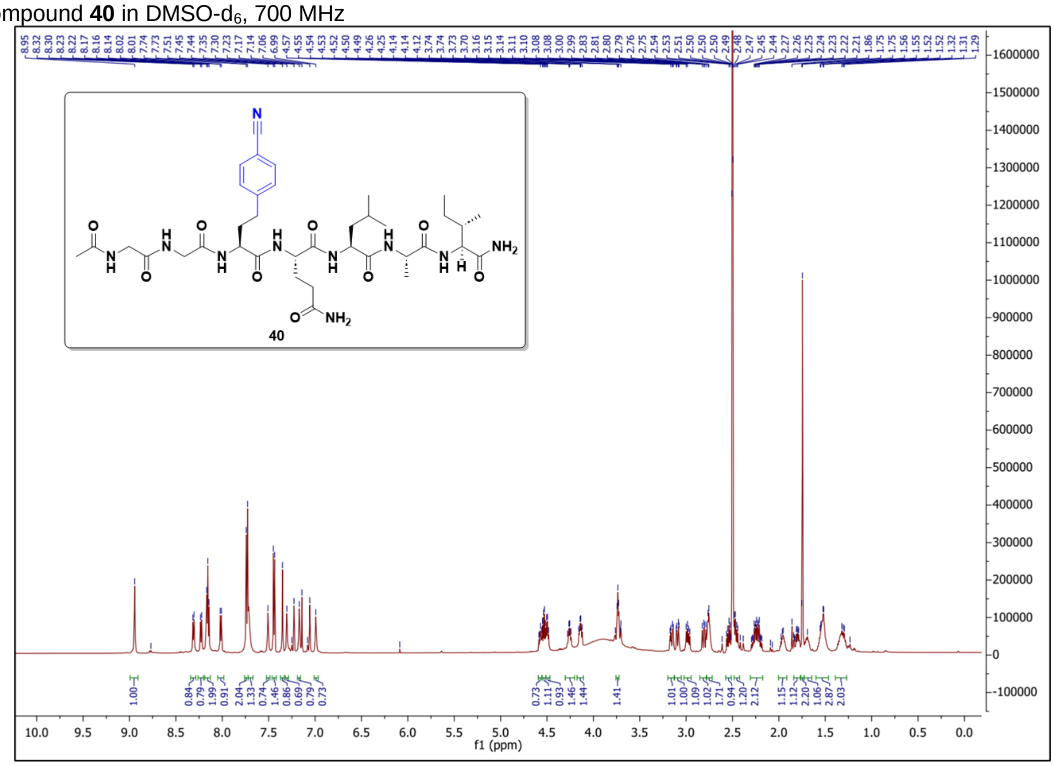

^{13}C NMR of compound **40** in DMSO-d$_6$, 176 MHz

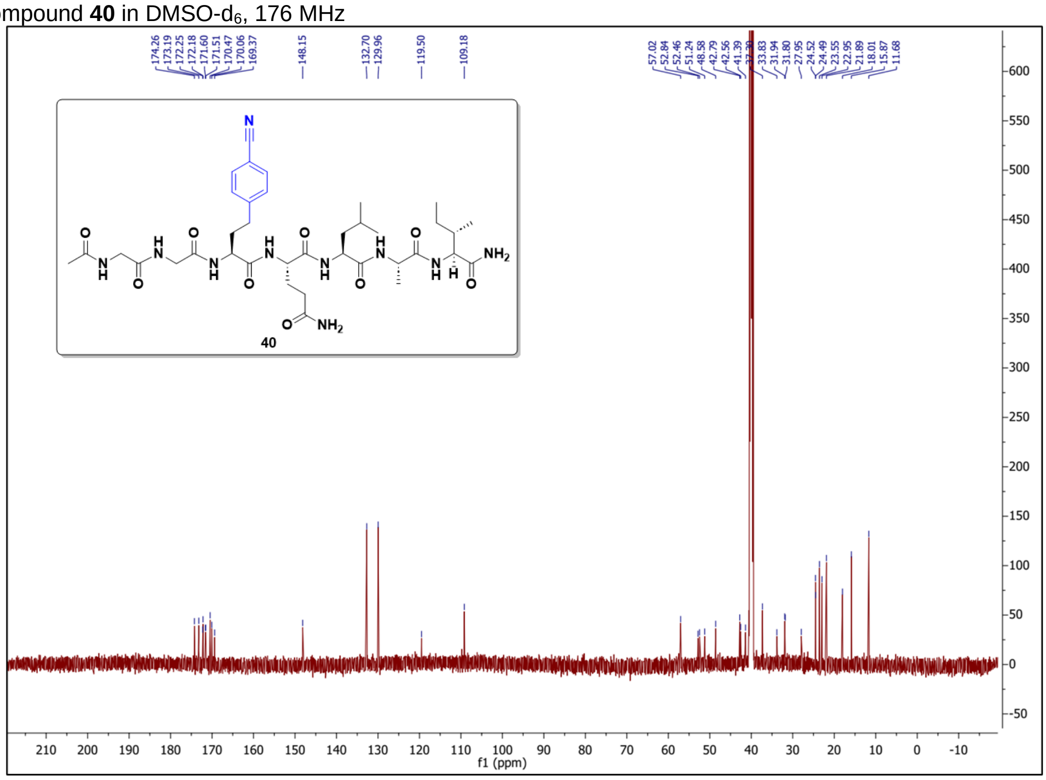

^{1}H NMR of compound **41** in DMSO-d_6, 700 MHz

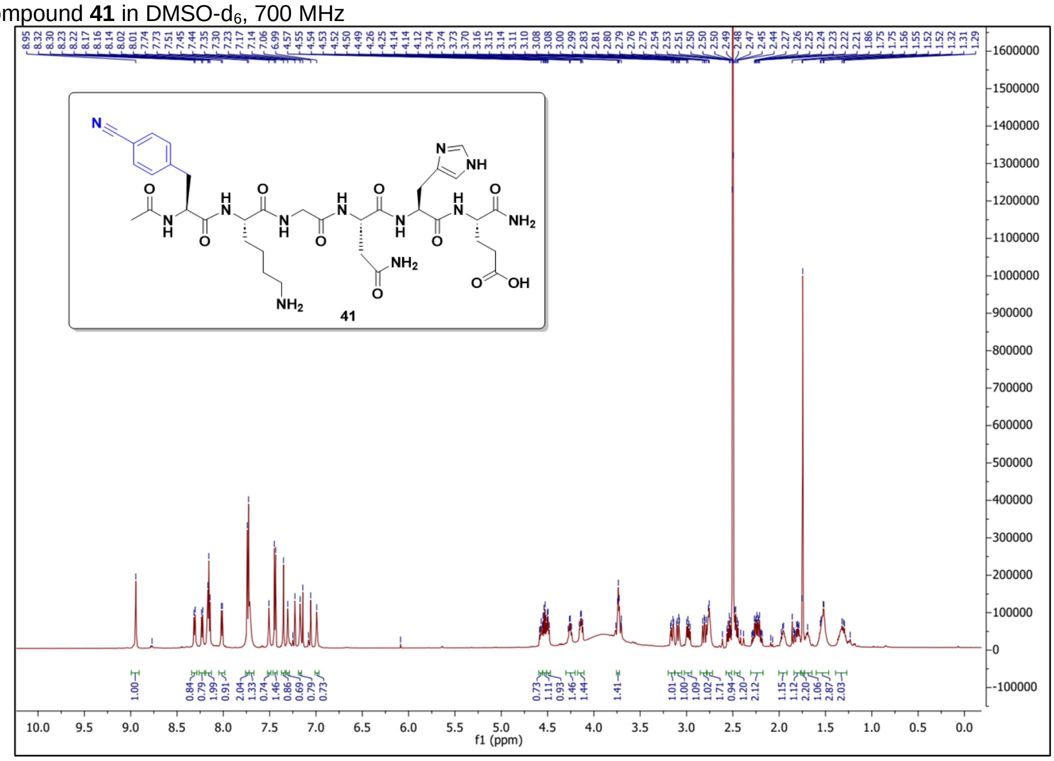

^{13}C NMR of compound **41** in DMSO-d_6, 176 MHz

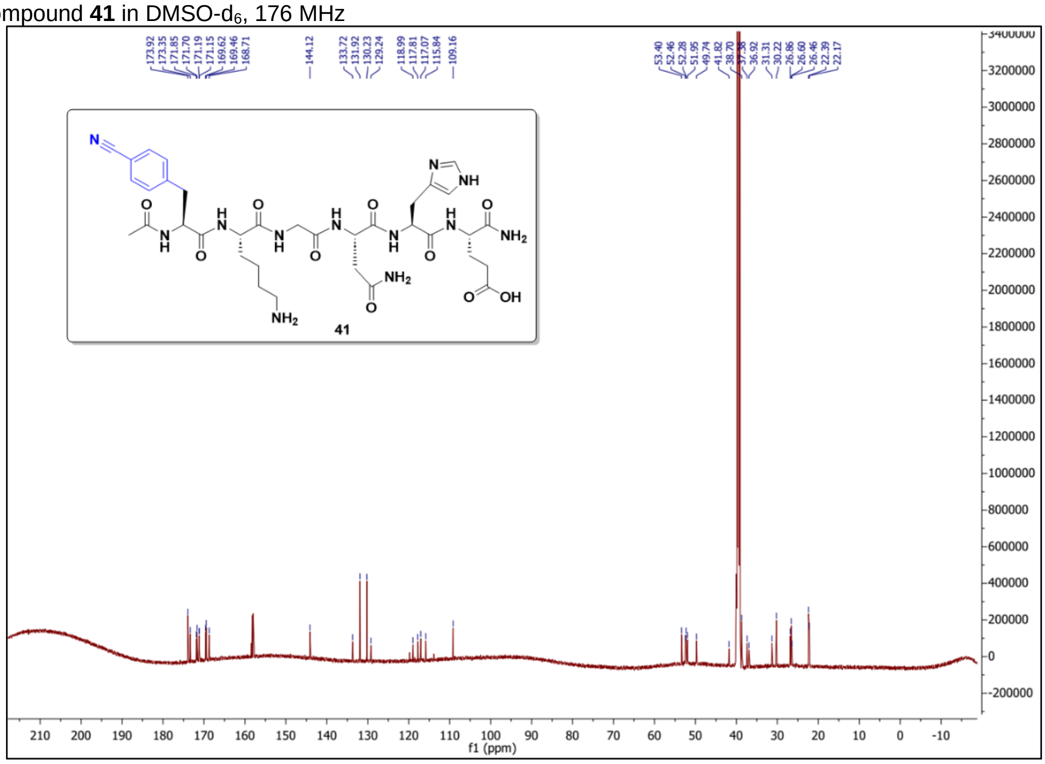

^{19}F NMR of compound **44** in DMSO-d$_6$, 470 MHz (* corresponds to residual trifluoroacetic acid)

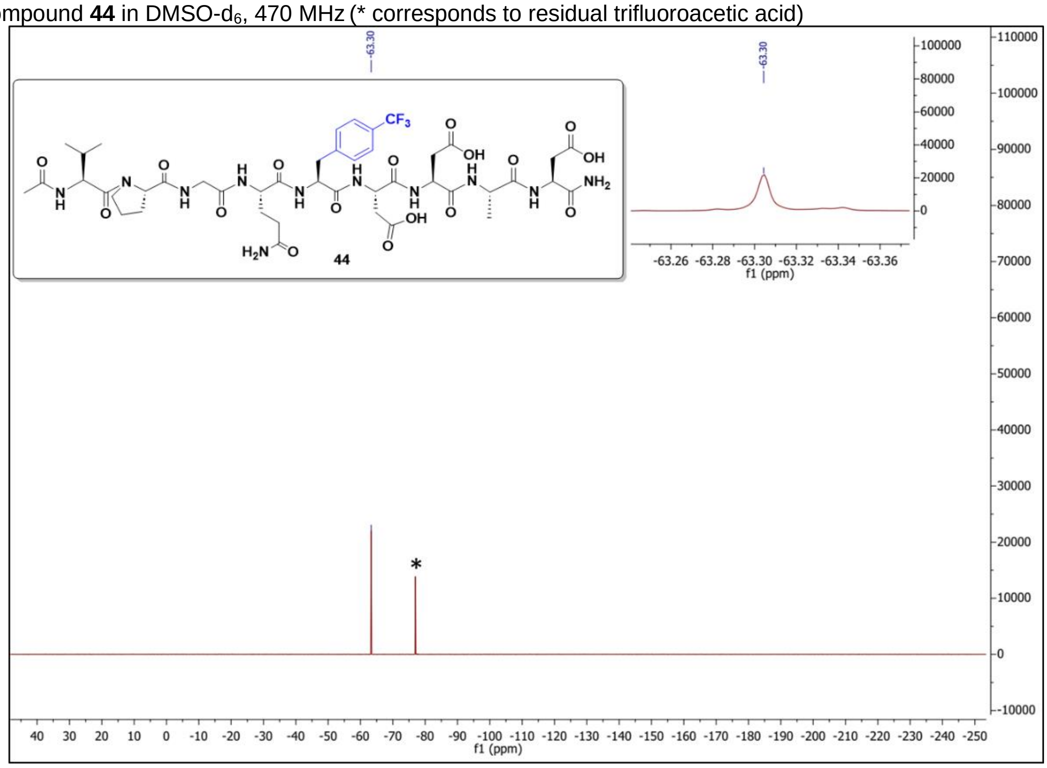

^{19}F NMR of compound **45** in DMSO-d$_6$, 470 MHz (* corresponds to residual trifluoroacetic acid)

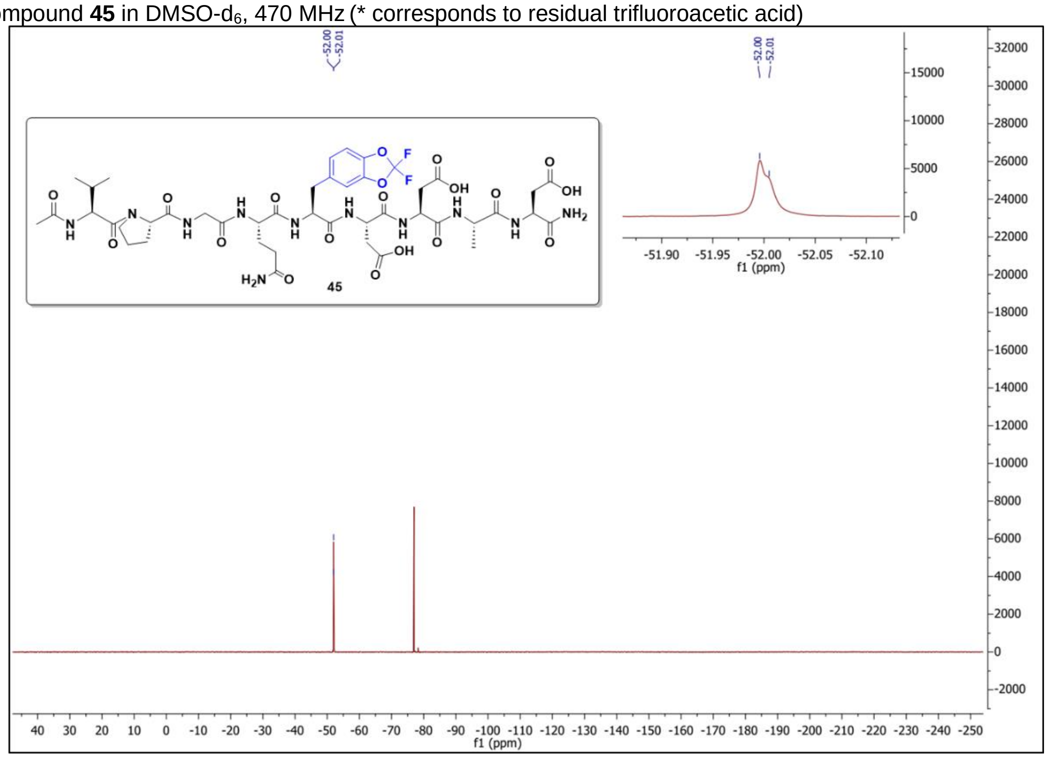

References

(1) Teixeira, A.; Benckhuijsen, W. E.; Koning, P. E. de; Valentijn, A. R. P. M.; Drijfhout, J. W. The Use of Dodt as a Non-Malodorous Scavenger in Fmoc-Based Peptide Synthesis. *Protein Pept. Lett.* **2002**, *9* (5), 379–385.

(2) Anka-Lufford, L. L.; Huihui, K. M. M.; Gower, N. J.; Ackerman, L. K. G.; Weix, D. J. Nickel-Catalyzed Cross-Electrophile Coupling with Organic Reductants in Non-Amide Solvents. *Chem. Eur. J.* **2016**, *22* (33), 11564–11567.

(3) Krzyzanowski, A.; Esser, L. M.; Willaume, A.; Prudent, R.; Peter, C.; 't Hart, P.; Waldmann, H. Development of Macrocyclic PRMT5–Adaptor Protein Interaction Inhibitors. *J. Med. Chem.* **2022**, *65* (22), 15300–15311.
